# Challenge *N*- versus *O*-six-membered annulation: FeCl_3_-catalyzed synthesis of heterocyclic *N*,*O*-aminals

**DOI:** 10.3762/bjoc.20.123

**Published:** 2024-06-26

**Authors:** Giacomo Mari, Lucia De Crescentini, Gianfranco Favi, Fabio Mantellini, Diego Olivieri, Stefania Santeusanio

**Affiliations:** 1 Department of Biomolecular Sciences, Section of Chemistry and Pharmaceutical Technologies, University “Carlo Bo” of Urbino, Via Ca’ le Suore 2-4, 61029, Urbino (PU), Italy

**Keywords:** α-aminoacetals, fused-ring systems, heterocyclic hemiaminals, heterocyclic *N,O*-aminals, multicomponent reactions

## Abstract

A new class of heterocyclic *N*,*O*-aminal and hemiaminal scaffolds was successfully obtained by means of a three-component reaction (3-CR) of 1,2-diaza-1,3-dienes (DDs), α-aminoacetals and iso(thio)cyanates. These stable imine surrogates are generated from key-substituted (thio)hydantoin intermediates through selective FeCl_3_-catalyzed intramolecular *N*-annulation.

## Introduction

**Figure 1:**
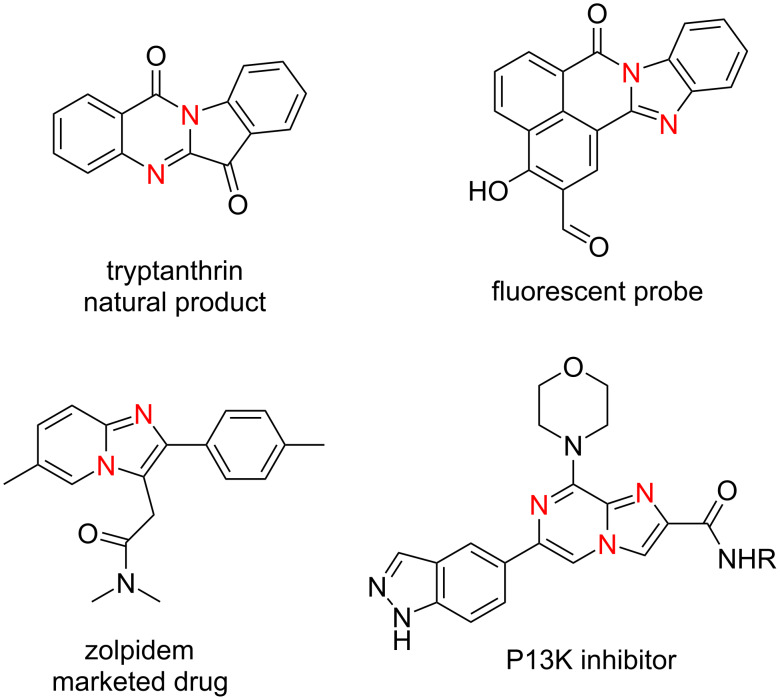
Representative examples of relevant *N*-fused heterocycles.

*N*-Fused heterocycles are ubiquitous within crucial molecules, including biologically active natural products, pharmaceuticals, and functional materials (Figure 1) [1–3].

It has been assessed that almost one-third of the best-selling therapeutics contains fused heterocyclic structures [4]. Among the *N*-heterocycles, imidazopyrazine structures [5–6], derived from amalgamation of privileged imidazole and pyrazine pharmacophores, are well represented in the area of medicinal chemistry since they possess pharmacological properties as mammalian target of rapamycin (mTOR) inhibitors [7], adenosine triphosphate (ATP) competitive inhibitors of the insuline-like growth factor 1 (IGF-1) receptor related to Ewing sarcoma [8], IGF-1 receptor inhibitors [9] or act as ligands on corticotropin releasing hormone (CRH) [10], γ-aminobutyric acid (GABA) [11] and melanocortin receptors [12].

Given the established potencies of this class of *N*-ring-fused compounds, planned syntheses that simplify their preparation by using small building blocks and that lead, through appropriate transformations, to a product that becomes a substrate for another complexity-generating reaction, merit investigation [13–15].

**Scheme 1:**
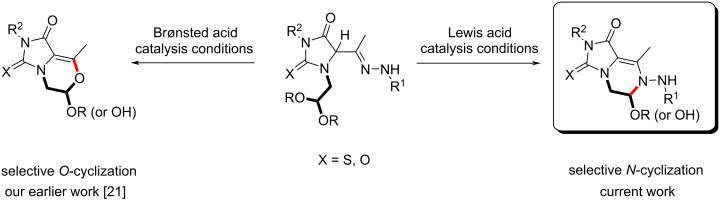
Different acid-catalyzed six-membered ring cyclizations.

Herein, we report a 3-CR-based synthesis of new properly decorated (thio)hydantoin framework able to afford, by a chemospecific Lewis acid-catalyzed ring-closure protocol, valuable heterocyclic *N*,*O*-aminals (Scheme 1).

## Results and Discussion

Since the direct functionalization of *N*-heterocycles offers an attractive entry to important molecular targets that might otherwise require lengthy synthetic procedures [16], our consolidated 3-CR strategy [17–19] implicates a careful selection of the starting components that ensures the installation of functionalities to be converged, by regioselective control, in different ring-closing processes [20–21].

With these considerations in mind and with the aim of diversity-oriented synthesis of *N*-heterocycles via sequential multicomponent approaches, we envisioned that α-aminoacetals could act as bifunctional building blocks along with 1,2-diaza-1,3-diene (DD) coupling partners [22–23], in obtaining functionalized *N*-aminohydrazones as key intermediates.

**Scheme 2:**
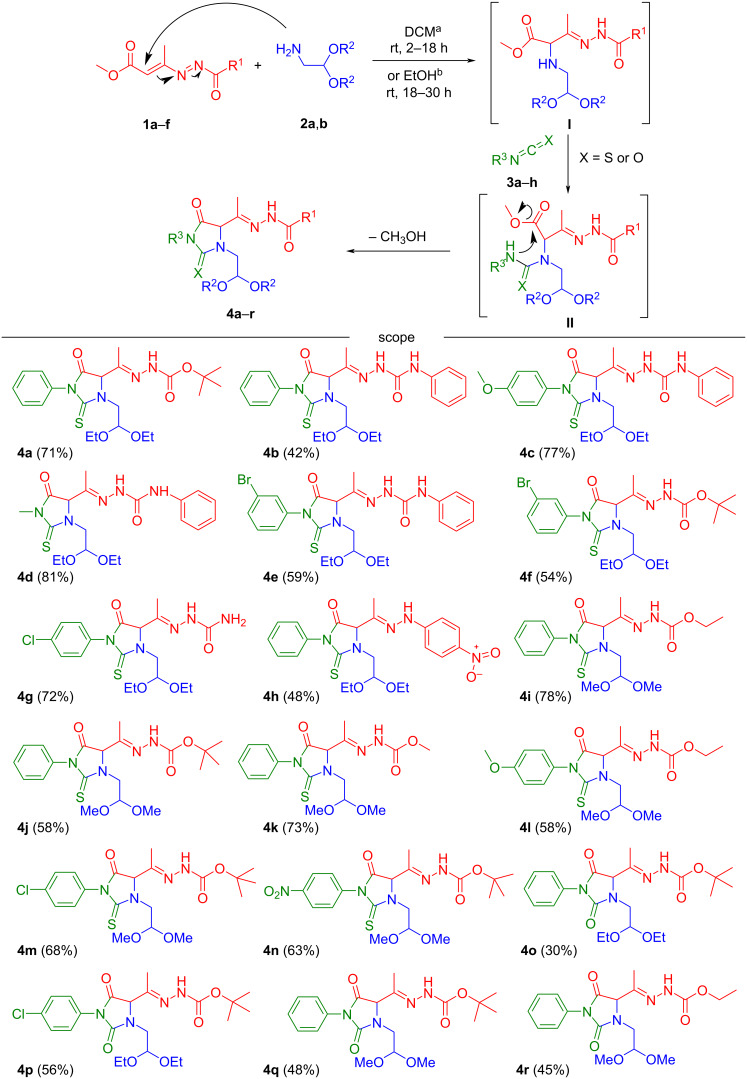
Substrate scope for the assembly of suitably *N*-3-functionalized (thio)hydantoins **4a–r**. ^a^DCM was utilized as solvent with isothiocyanates **3a**–**f**, while ^b^EtOH was utilized with isocyanates **3g**,**h**.

Based on our previous findings [17–19], the initial nucleophilic addition of α-aminoacetals **2a**,**b** as nitrogen source to the activated heterodiene system of 4-methoxycarbonyl-DDs **1a**–**f** in dichloromethane (DCM) or ethanol (EtOH) at room temperature affords *N*-aminohydrazone derivatives **I** (Scheme 2), whose sequential acylation process by iso(thio)cyanates **3a**–**h** gives rise to the asymmetric (thio)urea derivatives (intermediate **II**). The spontaneous nucleophilic attack of the (thio)amide nitrogen on the terminal methyl ester function at C-4 of the starting azo-ene system provides a regioselective heteroring closure, positioning appropriate functions both at N-3 and C-4 of the (thio)hydantoin frameworks **4a**–**r** (30–81%) broadening their usable decorations (Scheme 2).

Recently, we reported that compounds **4a**, **4f**, and **4m** undergo an intramolecular cyclization process through the involvement of the restored keto function of the hydrazone moiety and the open-chain hemiacetal or aldehyde hydrate in Brønsted acid medium to access 1*H*-imidazo[5,1-*c*][1,4]oxazine derivatives (Scheme 1) [21].

Considering that the hydrazone function at C-4 of **4a**–**r** may exist in a tautomeric equilibrium with the corresponding ene-hydrazino form [17,24–25], we conceived the idea of reversing the reactivity of **4a–r** in the six-membered cyclization process (*N*- vs *O*-annulation) through the generation of an electrophilic oxocarbenium [26–27] cation intermediate from the acetal residue at *N*-3 of the (thio)hydantoin core. To pursue our goal, different Lewis acids (10 mol %) such as Zn(OTf)_2_, CuCl_2_, and FeCl_3_ were screened at room temperature in different solvents, employing compound **4a** as the model substrate (Table 1).

**Table 1:** Optimization conditions for the Lewis acid-catalyzed intramolecular cyclization of **4a**.

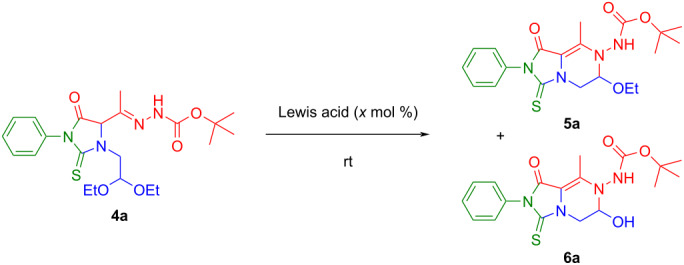					

Entry^a^	Lewis acid	Solvent	Time (h)	**5a** (yield %)^b^	**6a** (yield %)^b^

1	Zn(OTf)_2_ (10 mol %)	DCM	42	38	22
2	CuCl_2_ (10 mol %)	DCM	96	28	15
3	FeCl_3_ (10 mol %)	DCM	86	44	31
4	FeCl_3_ (20 mol %)	DCM	38	63	18
5	FeCl_3_ (30 mol %)	DCM	2	73	8
6	FeCl_3_ (30 mol %)	ACN	2	68	13
7	FeCl_3_ (30 mol %)	THF	2	62	11
8	FeCl_3_ (30 mol %)	EtOH	48	–^c^	–^c^

^a^The reactions were performed on a 0.5 mmol scale in 5 mL of solvent. ^b^Isolated yields of products **5a** and **6a** based on starting **4a**. ^c^Not detected by TLC analysis.

From the set of data collected, both the formation of *N,O*-aminal **5a** and corresponding hemiaminal **6a** were observed (entries 1–7, Table 1). Similarly to what was observed by Yu and co-workers for the intramolecular cyclization of alkynyl aldehyde acetals [28–29], it was found that the use of FeCl_3_ provided the better result in terms of overall yield (entry 3, Table 1). Moreover, the choice of iron(III) seemed to have remarkable advantages such as an environmentally benign alternative to traditional transition-metal catalysis, a low cost, nontoxicity, good stability, and easy handling [30–31]. Upon increasing the amount of FeCl_3_ to 20 mol %, the time of the reaction was reduced from 86 to 38 hours, and the yield of **5a** was incremented with respect to **6a** (entry 4, Table 1). Rising the amount of FeCl_3_ to 30 mol %, the reaction was complete in 2 hours, enhancing the yield of **5a** (73%) and minimizing the yield of **6a** (8%) (entry 5, Table 1). In reactions carried out in acetonitrile (ACN) or tetrahydrofuran (THF) the yield of **5a** decreased, while utilizing ethanol the reaction proceeded slowly and produced a complicated mixture in which both **5a** and **6a** were not detected (Table 1, entries 6–8).

**Scheme 3:**
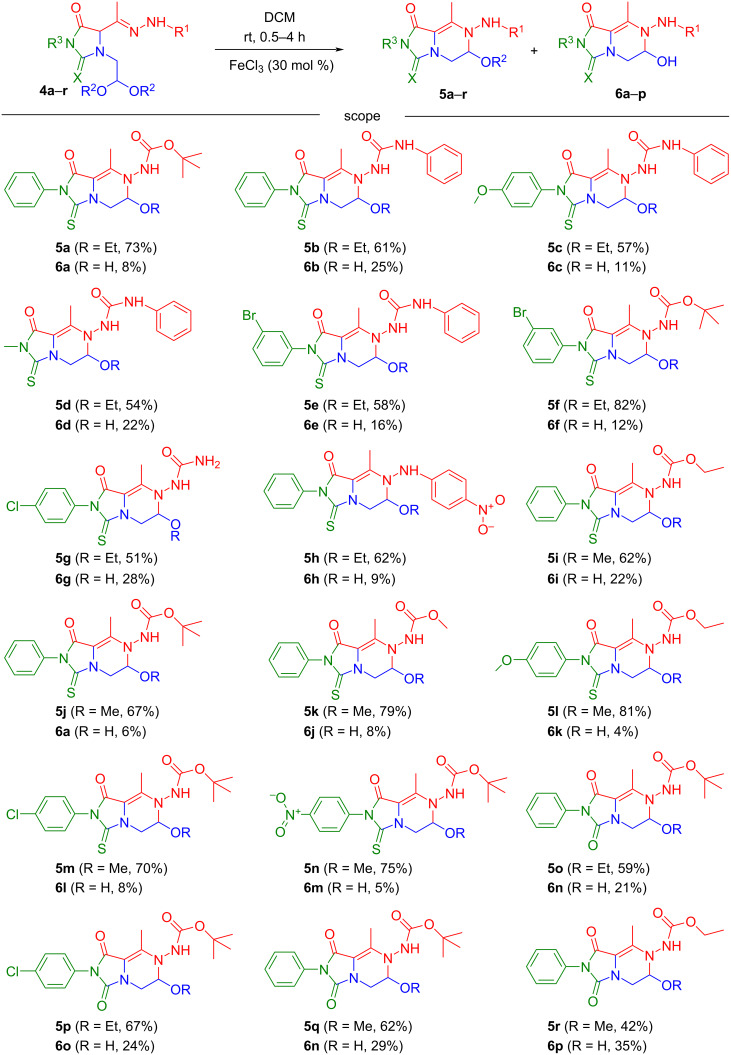
Substrate scope of the iron(III)-catalyzed synthesis of functionalized heterocyclic *N,O*-aminals **5a–r** and hemiaminals **6a–p**.

With the optimized conditions in hand (Table 1, entry 5), a selection of *N*-3-functionalized (thio)hydantoins (**4a**–**r**, 1 mmol) were dissolved in DCM (10 mL), FeCl_3_ (30 mol %) added and magnetically stirred at room temperature. Within 2–30 h, the reactions went to completion (TLC monitoring), affording, at last, *N*,*O*-aminals **5a**–**r** (42–82%) and the corresponding hemiaminals **6a**–**p** (4–35%) after column chromatography (Scheme 3).

An increased yield of **6** was observed alongside a decreased yield of **5**, in all those cases that required prolonged reaction times (24–30 h). This event led us to suppose the formation of carbinolamine **6** from *N*,*O*-aminal **5** owing to the nucleophilic attack of a water molecule, probably caused by the enriched moisture content of the reaction environment during the time.

**Scheme 4:**
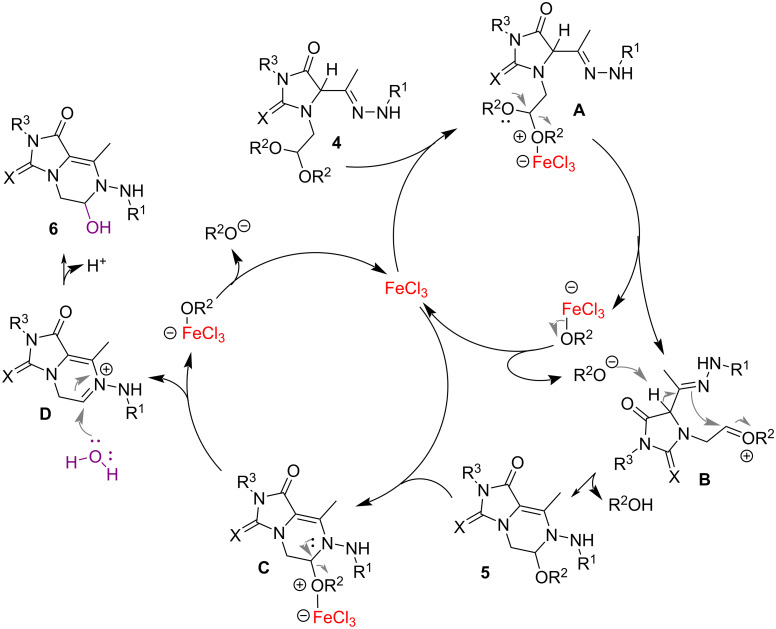
Proposed mechanism for the formation of *N,O*-aminals **5** and hemiaminals **6**.

Then, to explain the related formation of **5** and **6**, we hypothesized a plausible reaction mechanism in which iron is involved in two concomitant catalytic cycles (Scheme 4). Initially, FeCl_3_ forms an acid–base complex with one of the alkoxy groups of **4** providing intermediate **A**. The latter, by loss of a trichloro(alkoxy)ferrate(III) anion, generates a strong electrophile such as the oxocarbenium cation intermediate **B**. The released trichloro(alkoxy)ferrate(III) splits into FeCl_3_, which enters the catalytic cycle, and a free alkoxide, which acts as a base, promoting, via hydrazone–enamine tautomerization [17,24–25], the nucleophilic addition which concludes with the construction of the heterocyclic *N*,*O*-aminal **5** through the intramolecular N–C bond formation. The FeCl_3_ can also interact with the newly formed *N*,*O*-aminals **5**, giving rise to the second parallel catalytic cycle. Similar to what was previously observed, the elimination of the trichloro(alkoxy)ferrate(III) anion from intermediate **C** provides the iminium ion **D**, susceptible to nucleophilic attack by a water molecule present in the reaction medium, leading to the carbinolamines **6**. This latter synthesis represents an interesting example of auto-tandem catalysis in which FeCl_3_ promotes two subsequent reactions.

**Scheme 5:**
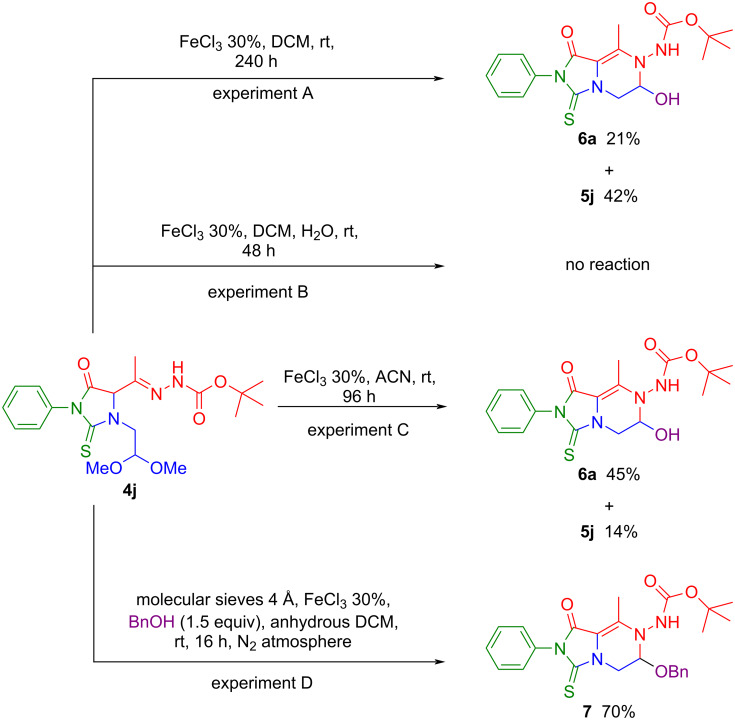
Control mechanistic experiments.

For further confirmation to support our mechanistic hypothesis and in an attempt to switch the reaction toward the formation of hemiaminal **6a**, we repeated the reaction of thiohydantoin **4j**, (chosen as representative substrate), under the same previously optimized conditions, but extending the reaction time to 240 hours (experiment A in Scheme 5). In this case, the yield of hemiaminal **6a** increased from 6% recorded after two hours at the complete conversion of **4j** (Scheme 3) to 21% (experiment A, Scheme 5), in line with the values found for the slower reactions previously described (compounds **6b**,**d**,**g**,**i**,**n–p**). By adding 500 μL of water to the medium the cyclization did not proceed, and the starting material **4j** was recovered unchanged (experiment B, Scheme 5). This observation seems to suggest that the presence of a high water amount results in catalyst deactivation.

Based on these results and what was observed in the optimization tests (Table 1, entry 6), we extended the reaction time but used ACN as solvent, which possesses a higher water content with respect to DCM (experiment C, Scheme 5). Gratifyingly, in this case, the formation of carbinolamine **6a** becomes predominant (45%), despite a small quantity of *N,O*-aminal **5j** (14%) is also produced, by virtue of the alcohol released from the starting acetal **4j**. Probably, the higher water concentration in acetonitrile shifts the equilibrium in favour of **6a** over the time (Scheme 4).

Within our proposed catalytic cycle, when compound **4j** is utilized, methanol is released, due to the presence of a dimethyl acetal residue. Therefore, using molecular sieves (MS 4 Å) little alcohol molecules, such as MeOH, can be potentially trapped, allowing the insertion of a more encumbered alcohol, such as benzyl alcohol, which is not sequestered by MS 4 Å. As a matter of fact, the new benzylated *N,O*-aminal **7** was successfully obtained in 70% isolated yield as the sole product (experiment D, Scheme 5). In this latter case, the benzyl alcohol presumably reacts with the iminium ion **D** formed in the second catalytic cycle (Scheme 4), and its sole formation is ascribable to the capability of molecular sieves of sequestering MeOH eventually formed, shifting the equilibrium towards **7**.

Noteworthy, in compounds **5a**–**r**, **6a**–**p** and **7**, the newly created heterocyclic nucleus represents a new example of cyclic *N,O*-aminals and carbinolamine derivatives, an interesting class of organic compounds that are common structural motifs embedded within diverse biologically important natural products and pharmaceuticals [32–37]. On the other hand, the *N,O*-aminals are stable and very practical synthetic intermediates commonly employed for the in situ generation of highly electrophilic iminium ions [38–41].

## Conclusion

In summary, we planned the synthesis of decorated imidazo skeletons accessible through a judicious choice of the starting components of a 3-CR process and developed a catalytic system-controlled selective intramolecular *N*-annulation process for ring-fused biheterocyclic *N*,*O*-aminal derivatives as stable imine equivalents and useful tools for new bond formation in view of further fused-heterocylization processes. Moreover, control experiments corroborate our mechanistic hypothesis related to the formation of both *N*,*O*-aminals and corresponding hemiaminals. In particular, the domino reaction that leads to the carbinolamines represents an interesting example of “auto-tandem catalysis” in which the FeCl_3_ catalyzes two different chemical transformations in a single reactor, reducing the number of steps and the amount of waste with consequent benefits of cost and environmental impact [42–43].

## Supporting Information

File 1 General experimental information, synthetic procedures, analytical data and NMR spectra for the reported compounds.

## Data Availability

All data that supports the findings of this study is available in the published article and/or the supporting information to this article.
