# Supplementary material for: Challenge N- versus O-six-membered annulation: FeCl3-catalyzed synthesis of heterocyclic N,O-aminals
Source: Beilstein J Org Chem. 2024 Jun 26;20:1412–20. doi: 10.3762/bjoc.20.123 (PMC11216082; doi:10.3762/bjoc.20.123)
Supplement: File 1 — General experimental information, synthetic procedures, analytical data and NMR spectra for the reported compounds. [file Beilstein_J_Org_Chem-20-1412-s001.pdf]

BEILSTEIN JOURNAL OF ORGANIC CHEMISTRY

Supporting Information

for

Challenge *N*- versus *O*-six-membered annulation: $FeCl_3$-catalyzed synthesis of heterocyclic *N*,*O*-aminals

Giacomo Mari, Lucia De Crescentini, Gianfranco Favi, Fabio Mantellini, Diego Olivieri and Stefania Santeusanio

Beilstein J. Org. Chem. **2024,** *20,* 1412–1420. doi:10.3762/bjoc.20.123

General experimental information, synthetic procedures, analytical data and NMR spectra for the reported compounds

License and Terms: This is a supporting information file under the terms of the Creative Commons Attribution License (https://creativecommons.org/licenses/by/4.0). Please note that the reuse, redistribution and reproduction in particular requires that the author(s) and source are credited and that individual graphics may be subject to special legal provisions.
The license is subject to the *Beilstein Journal of Organic Chemistry* terms and conditions: (https://www.beilstein-journals.org/bjoc/terms)

Table of contents

General information S2

Table S1: Substituted 1,2-diaza-1,3-dienes (DDs) **1a**–**f** employed S3

Table S2: Nucleophiles **2a**,**b** employed S3

Table S3: Substituted isothiocyanates **3a**–**f**, and isocyanates **3g**,**h** employed S3

General procedures S4

Characterization of compounds **4a**–**r**, **5a**–**r**, **6a**–**p**, and **7** S6

Copies of NMR spectra for compounds **4b**–**e**, **4g**–**l**, **4n**–**r**, **5a**–**r**, **6a**–**p**, and **7** S24

References S74

1. General information

All commercially available reagents and solvents were used without further purification. The control experiment to increase hemiaminal formation was conducted using ACN from Merck, ACS reagent purity grade (≥ 99.5%).The control experiment with benzyl alcohol was conducted using DCM distilled over CaH_2 and preserved on activated MS 4Å. 1,2-Diaza-1,3-dienes **1a**–**f** were synthesized as a mixture of *E*/*Z* isomers as previously reported.[1,2] Chromatographic purification of compounds was carried out on silica gel (60–200 μm). TLC analysis was performed on pre-loaded (0.25 mm) glass supported silica gel plates (Kieselgel 60); compounds were visualized by exposure to UV light and by dipping the plates in 1% $Ce(SO_4)\bullet 4H_2O$, 2.5% $(NH_4)_6Mo_7O_{24}\bullet 4H_2O$ in 10% sulphuric acid followed by heating on a hot plate. Melting points were determined in open capillary tubes with a Gallenkamp apparatus and are uncorrected. All ^1H NMR, ^{13}C NMR spectra and 2D experiments were recorded on a Bruker Avance at 400 and 100 MHz, respectively, using $[D_6]$DMSO as solvent. Chemical shifts (δ scale) are reported in parts per million (ppm) relative to the central peak of the solvent and are sorted in ascending order within each group. The following abbreviations are used to describe peak patterns where appropriate: s = singlet, d = doublet, dd = doublet of doublet, dt = doublet of triplet, t = triplet, q = quartet, m = multiplet and br = broad signal, Ar, aromatic hydrogen. All coupling constants (*J* value) are given in hertz [Hz]. All the OH, NH and NH_2 exchanged with D_2O. The multiplicities in ^{13}C NMR spectra were obtained using HMQC experiments to aid in assignment (q = methyl, t = methylene, d = methine, s = quaternary). In some spectra double peaks are due to *E*/*Z* isomerism or to a mixture of diastereomers. High- and low-resolution mass spectrometry was performed on a Micromass Q-ToF Micro mass spectrometer (Micromass, Manchester, UK) using an ESI source.

2 Table S1: Substituted 1,2-diaza-1,3-dienes (DDs) 1a–f employed.[1]

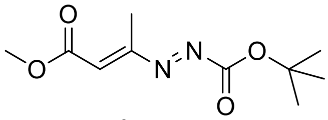

1a

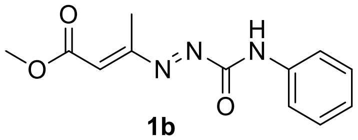

1b

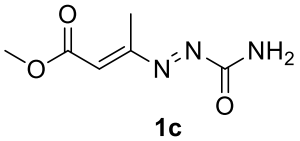

1c

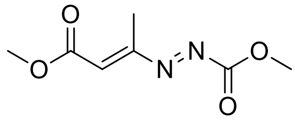

1d

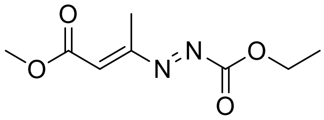

1e

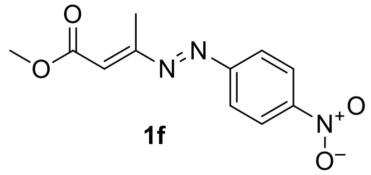

1f

3 Table S2: Nucleophiles 2a,b.

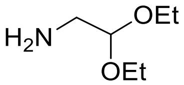

2a

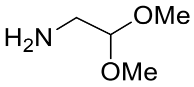

2b

4 Table S3: Substituted isothiocyanates 3a–f and isocyanates 3g,h employed.

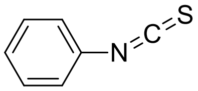

3a

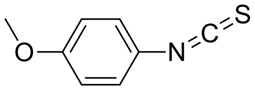

3b

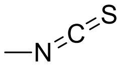

3c

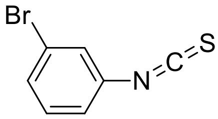

3d

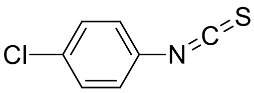

3e

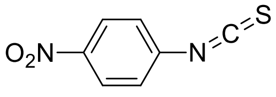

3f

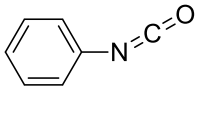

3g

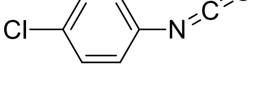

3h

5 General procedures

*Sequential MCR procedure for the synthesis of substituted 2-thiohydantoins **4a–n**.*

In a similar manner,[3] to a solution of DD **1a–f** (1.0 mmol) in DCM (10.0 mL) at room temperature, α-aminoacetal **2a**,**b** (1.0 mmol) was added and the reaction mixture was stirred at room temperature until the disappearance of the corresponding DD and the formation of the *N*-adduct (TLC control, 0.50–2.0 h). Directly, to the reaction medium, 1.0 equiv of isothiocyanate **3a**–**f** was then added, and the reaction was left at room temperature under magnetic stirring (2–18 h). After the removal of the reaction solvent under reduced pressure, derivatives **4a**,**d**,**f**,**h–j**,**l–n** were obtained by column chromatography (eluting with a mixture of cyclohexane/ethyl acetate, from 60:40 to pure ethyl acetate) and crystallized from appropriate solvents. In the cases of derivatives **4b**,**c**,**e**,**g**,**k**, the products precipitated directly from the reaction mixture and were collected by filtration in vacuo. Then, their mother liquors were dried over anhydrous Na_2SO_4, the solvent removed under reduced pressure and the crudes chromatographed on silica gel column (eluting with a mixture of cyclohexane/ethyl acetate, from 60:40 to pure ethyl acetate). The pure products **4a–n**, were then crystallized from appropriate solvents.

*Sequential MCR procedure for the synthesis of substituted hydantoins **4o**–**r***

In a similar manner,[3] to a solution of DD **1a**,**e** (1.0 mmol) in EtOH (10.0 mL) at room temperature, α-aminoacetal **2a**,**b** (1.0 mmol) was added and the reaction mixture was stirred at room temperature until the disappearance of the corresponding DD and the formation of the *N*-adduct (TLC control, 0.50–2.0 h). Directly to the reaction medium, 1.0 equiv of isocyanate **3g,h** was then added, and the reaction was left at room temperature under magnetic stirring (18–30 h). Then, the solvent was removed under reduced pressure and the crude reaction mixture was chromatographed on silica gel column (eluting with a mixture of cyclohexane/ethyl acetate, from 60:40 to pure ethyl acetate). The obtained products **4o**–**r** were precipitated from ethyl acetate/light petroleum ether and recrystallized from appropriate solvents.

Procedure for the synthesis of substituted heterocyclic N,O-aminals 5a–r and hemiaminals 6a–p.

Derivative **4a–r** (1.0 mmol) was dissolved in DCM (10 mL) under magnetic stirring at room temperature. Then, $FeCl_3$ (0.3 mmol) was added and the reaction mixture was stirred until the disappearance of **4a**–**r** (0.5–4.0 h; TLC monitoring). After the removal of DCM under reduced pressure, the crude reaction mixture was chromatographed on silica gel column

(eluting with a mixture of cyclohexane/ethyl acetate, from 70:30 to 30:70) to separate **5a**–**r** from **6a**–**p** that were subjected to crystallization from appropriate solvents.

Procedure for the conversion of 2-thiohydantoin ***4j*** *into hemiaminal* ***6a*** *and N,O-aminal* ***5j****.*
Derivative **4j** (0.5 mmol, 218 mg) was dissolved in ACN (5 mL) under magnetic stirring at room temperature. Then, $FeCl_3$ (0.15 mmol, 24 mg) was added, and the reaction mixture was stirred for 96 h. After the removal of the solvent under reduced pressure, the crude reaction mixture was chromatographed on silica gel column (eluting with a mixture of cyclohexane/ethyl acetate, from 70:30 to 30:70) to separate the prevalent **6a** from **5j** that were subjected to crystallization from appropriate solvents.

Conversion of ***4j*** *into* ***7*** *under nitrogen atmosphere.*
In a flame-dried Schlenk flask under nitrogen, containing a magnetic stirring bar and activated MS 4Å (330 mg), derivative **4j** (0.15 mmol, 66 mg), $FeCl_3$ (0.045 mmol, 7.3 mg), benzyl alcohol (0.225 mmol, 24 μL) and DCM (1.5 mL) were added and the reaction mixture was stirred under nitrogen for 24 h. The crude reaction mixture was filtered through a plug of silica gel, washing with ethyl acetate/cyclohexane (v:v; 60:40) and the resulting filtrate was evaporated under reduced pressure. The mixture was chromatographed on silica gel column, eluting with dichloromethane until all the remaining benzyl alcohol had been removed (TLC check). Then, DCM/Et_2O (v:v; 90:10) was utilized, to obtain the pure product **7** as a yellow solid in 70% isolated yield.

6 Characterization of compounds 4a–r, 5a–r, 6a–p, and 7

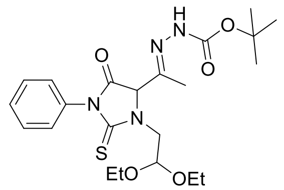

***tert*-Butyl 2-(1-(3-(2,2-diethoxyethyl)-5-oxo-1-phenyl-2-thioxoimidazolidin-4-yl)ethylidene)hydrazinecarboxylate (4a)**[3]:

The characterization data of **4a** are available at: https://chemistry-europe.onlinelibrary.wiley.com/action/downloadSupplement?doi=10.1002%2Fejoc.202201053&file=ejoc202201053-sup-0001-misc_information.pdf.

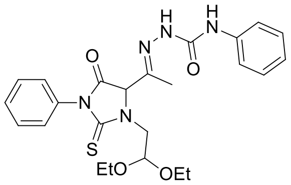

2-(1-(3-(2,2-Diethoxyethyl)-5-oxo-1-phenyl-2-thioxoimidazolidin-4-yl)ethylidene)-*N*-phenylhydrazinecarboxamide (4b): reaction time: 8 h; yield 42%, (203.1 mg); white powder from DCM; m.p. 194-195 °C; ^{1}H NMR (400 MHz, DMSO-d_6): δ 1.10-1.17 (m, 6H, 2 x OCH_2CH_3), 1.85 (s, 3H, CH_3), 3.48-3.69 (m, 5H, 2 x OCH_2CH_3 and NCH_aH_b), 4.11 (dd, ^{2}J = 14.4 Hz, ^{3}J = 4.8 Hz, 1H, NCH_aH_b), 4.88 (t, J = 5.2 Hz, 1H, CH), 5.24 (s, 1H, CH), 7.02 (t, J = 7.6 Hz, 1H, Ar), 7.29-7.37 (m, 4H, Ar), 7.47- 7.58 (m, 5H, Ar), 8.87 (s, 1H, NH, D_2O exch.), 10.12 (s, 1H, NH, D_2O exch.); ^{13}C NMR (100 MHz, DMSO-d_6): δ 12.3, 15.1, 15.2, 48.1, 61.7, 62.1, 69.9, 98.8, 119.2, 122.5, 128.6, 128.7, 128.9, 133.5, 138.8, 140.1, 152.8, 170.0, 182.8; HRMS (ESI) calcd. for $C_{24}H_{30}N_5O_4S$ [M + H$^+$] 484.2013; found 484.2005.

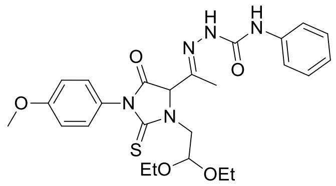

2-(1-(3-(2,2-Diethoxyethyl)-1-(4-methoxyphenyl)-5-oxo-2 thioxo-imidazolidin-4-yl)ethylidene)-*N*-phenylhydrazinecarboxamide (4c): reaction time: 15 h; yield 77%, (395.5 mg); white powder from DCM; m.p. 188-189 °C; ^{1}H NMR (400 MHz, DMSO-d_6): δ 1.10-1.16 (m, 6H, 2 x OCH_2CH_3), 1.83 (s, 3H, CH_3), 3.47-3.68 (m, 5H, 2 x OCH_2CH_3 and NCH_aH_b), 3.81 (s, 3H, OCH_3), 4.09 (dd, ^{2}J = 14.4 Hz, ^{3}J = 4.8 Hz, 1H, NCH_aH_b), 4.87 (t, J = 5.2 Hz, 1H, CH), 5.20 (s, 1H, CH), 7.00-7.06 (m, 3H, Ar), 7.24-7.33 (m, 4H, Ar), 7.57 (d, J = 8.8 Hz, 2H, Ar), 8.88 (s, 1H, NH, D_2O exch.), 10.13 (s, 1H, NH, D_2O exch.); ^{13}C NMR (100 MHz, DMSO-d_6): δ 12.3, 15.1, 15.2, 48.1, 55.4, 61.7, 62.0, 69.8, 98.8, 114.1, 119.2, 122.5, 126.0, 128.6, 129.8, 138.8, 140.2, 152.9, 159.4, 170.2, 183.3; HRMS (ESI) calcd. for $C_{25}H_{32}N_5O_5S$ [M + H$^+$] 514.2119 found 514.2125.

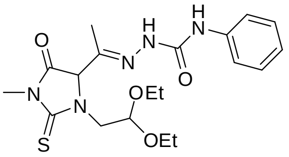

2-(1-(3-(2,2-Diethoxyethyl)-1-methyl-5-oxo-2-thioxoimidazolidin-4-yl)ethylidene)-*N*-phenylhydrazinecarboxamide (4d):

reaction time: 18 h; yield 81%, (341.4 mg); white powder from cyclohexane/EtOAc; m.p. 140-141 °C; ^{1}H NMR (400 MHz, DMSO-d_6): δ 1.07-1.13 (m, 6H, 2 x OCH_2CH_3), 1.70 (s, 3H, CH_3), 3.18 (s, 3H, CH_3), 3.41-3.64 (m, 5H, 2 x OCH_2CH_3 and NCH_aH_b), 4.06 (dd, ^{2}J = 14.4 Hz, ^{3}J = 4.8 Hz, 1H, NCH_aH_b), 4.80 (t, J = 5.2 Hz, 1H, CH), 5.04 (s, 1H, CH), 7.01 (t, J = 7.2 Hz, 1H, Ar), 7.30 (t, J = 7.2 Hz, 2H, Ar), 7.56 (d, J = 6.4 Hz, 2H, Ar), 8.85 (s, 1H, NH, D_2O exch.), 10.08 (s, 1H, NH, D_2O exch.); ^{13}C NMR (100 MHz, DMSO-d_6): δ 12.1, 15.0, 15.1, 28.2, 61.6, 62.0, 69.2, 98.7, 119.1, 122.4, 128.5, 138.6, 140.1, 152.7, 170.4, 183.3; HRMS (ESI) calcd. for $C_{19}H_{27}N_5O_4SNa$ [M + Na^+]: 444.1676; found: 444.1686.

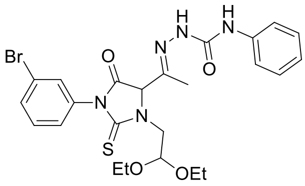

2-(1-(1-(3-Bromophenyl)-3-(2,2-diethoxyethyl)-5-oxo-2-thioxoimidazolidin-4-yl)ethylidene)-*N*-phenyl-hydrazinecarboxamide (4e):

reaction time: 16.5 h; yield 59%, (331.8 mg); white powder from DCM; m.p. 180-182 °C; ^{1}H NMR (400 MHz, DMSO-d_6): δ 1.11-1.18 (m, 6H, 2 x OCH_2CH_3), 1.86 (s, 3H, CH_3), 3.48-3.69 (m, 5H, 2 x OCH_2CH_3 and NCH_aH_b), 4.10 (dd, ^{2}J = 14.4 Hz, ^{3}J = 4.8 Hz, 1H, NCH_aH_b), 4.86 (t, J = 5.2 Hz, 1H, CH), 5.23 (s, 1H, CH), 7.02 (t, J = 7.6 Hz, 1H, Ar), 7.31 (t, J = 7.6 Hz, 2H, Ar), 7.41-7.44 (m, 1H, Ar), 7.50 (t, J = 8.4 Hz, 1H, Ar), 7.58 (d, J = 7.6 Hz, 2H, Ar), 7.68-7.70 (m, 2H, Ar), 8.87 (s, 1H, NH, D_2O exch.), 10.12 (s, 1H, NH, D_2O exch.); ^{13}C NMR (100 MHz, DMSO-d_6): δ 12.4, 15.1, 15.2, 48.2, 61.7, 62.0, 70.0, 98.8, 119.2, 121.1, 122.5, 128.1, 128.6, 130.7, 131.5, 132.0, 134.9, 138.8, 140.0, 152.8, 169.8, 182.4; HRMS (ESI) calcd. for $C_{24}H_{29}BrN_5O_4S$ [M + H^+]: 562.1118; found: 562.1126.

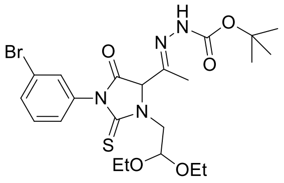

***tert*-Butyl 2-(1-(1-(3-bromophenyl)-3-(2,2-diethoxyethyl)-5-oxo-2-thioxoimidazolidin-4-yl)ethylidene)hydrazinecarboxylate (4f)**[3]:

The characterization data of **4f** are available at: https://chemistry-europe.onlinelibrary.wiley.com/action/downloadSupplement?doi=10.1002%2Fejoc.202201053&file=ejoc202201053-sup-0001-misc_information.pdf.

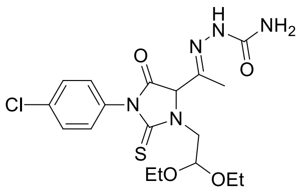

2-{1-[1-(4-Chlorophenyl)-3-(2,2-diethoxyethyl)-5-oxo-2-thioxoimidazolidin-4-yl]ethylidene}hydrazine carboxamide (4g): reaction time: 12.5 h; yield 72%, (318.2 mg); white powder from EtOAc/MeOH/Et_2O; m.p. 126-128 °C; ^{1}H NMR (400 MHz, DMSO-d_6): δ 1.02-1.16 (m, 6H, 2 x OCH_2CH_3), 1.77 (s, 3H, CH_3), 3.44-3.67 (m, 5H, 2 x OCH_2CH_3 and NCH_aH_b), 4.08 (dd, ^2J = 14.4 Hz, ^3J = 4.8 Hz, 1H, NCH_aH_b), 4.82 (t, J = 5.2 Hz, 1H, CH), 5.07 (s, 1H, CH), 6.45 (brs, 2H, NH_2, D_2O exch.), 7.40 (d, J = 8.4 Hz, 2H, Ar), 7.59 (d, J = 8.4 Hz, 2H, Ar), 9.69 (s, 1H, NH, D_2O exch.); ^{13}C NMR (100 MHz, DMSO-d_6): δ 12.3, 15.1, 15.2, 48.0, 61.7, 62.1, 64.9, 70.0, 98.8, 129.0, 130.6, 132.3, 133.6, 138.4, 156.6, 169.9, 183.4; HRMS (ESI) calcd. for $C_{18}H_{25}ClN_5O_4S$ [M + H$^+$]: 442.1310; found: 442.1329.

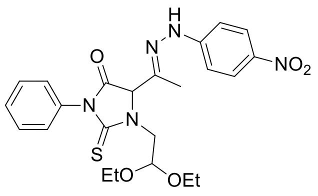

1-(2,2-Diethoxyethyl)-5-(1-(2-(4-nitrophenyl)hydrazono)ethyl)-3-phenyl-2-thioxoimidazolidin-4-one (4h): reaction time: 10 h; yield 48%, (233.0 mg); light orange powder from EtOAc/Et_2O; m.p. 129-130 °C; ^{1}H NMR (400 MHz, DMSO-d_6): δ 1.11 (t, J = 7.2 Hz, OCH_2CH_3), 1.18 (t, J = 7.2 Hz, OCH_2CH_3), 1.96 (s, 3H, CH_3), 3.46-3.70 (m, 5H, 2 x OCH_2CH_3 and NCH_aH_b), 4.21 (dd, ^2J = 14.0 Hz, ^3J = 4.0 Hz, 1H, NCH_aH_b), 4.82-4.85 (m, 1H, CH), 5.23 (s, 1H, CH), 7.28 (d, J = 9.2 Hz, 2H, Ar), 7.35 (d, J = 7.2 Hz, 2H, Ar), 7.45-7.54 (m, 3H, Ar), 8.17 (d, J = 9.2 Hz, 2H, Ar), 10.35 (s, 1H, NH, D_2O exch.); ^{13}C NMR (100 MHz, DMSO-d_6): δ 12.8, 15.1, 15.2, 47.8, 61.8, 62.1, 69.8, 99.1, 111.9, 125.8, 128.6, 128.9, 129.0, 133.5, 139.1, 140.6, 150.7, 170.1, 182.6; HRMS (ESI) calcd for $C_{23}H_{28}N_5O_5S$: [M + H$^+$]: 486.1806; found: 486.1815.

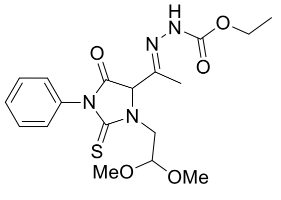

Ethyl 2-(1-(3-(2,2-dimethoxyethyl)-1-(4-methoxyphenyl)-5-oxo-2-thioxoimidazolidin-4-yl)ethylidene)hydrazinecarboxylate (4i): reaction time: 6 h; yield 78%, (318.6 mg); white solid from DCM; m.p 150 °C; ^{1}H NMR (400 MHz, , DMSO-d_6): δ 1.25 (t, J = 7.2 Hz, 3H, OCH_2CH_3), 1.82 (s, 3H, CH_3), 3.29 (s, 3H, OCH_3), 3.33 (s, 3H, OCH_3), 3.60 (dd, ^2J = 14.0 Hz, ^3J = 5.6 Hz, 1H, NCH_aH_b), 4.03 (dd, ^2J = 14.4 Hz, ^3J = 4.8 Hz, 1H, NCH_aH_b), 4.17 (q, J = 7.2 Hz, 3H, OCH_2CH_3), 4.69 (t, J = 5.2 Hz, 1H, CH), 5.12 (s, 1H, CH), 7.34 (d, J = 8.4 Hz, 2H, Ar), 7.46-7.53 (m, 3H, Ar), 10.37 (s, 1H, NH, D_2O exch.); ^{13}C NMR (100 MHz, DMSO-d_6): δ 12.7, 14.5, 47.3, 53.7, 53.8, 60.7, 69.8, 100.8, 128.6, 129.8, 129.0, 133.4, 143.9, 153.9, 169.9, 182.6; HRMS (ESI) calcd. for $C_{18}H_{25}N_4O_5S$: [M + H$^+$]: 409.1540; found: 409.1527.

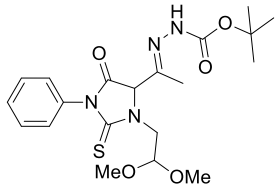

***tert*-Butyl 2-(1-(3-(2,2-dimethoxyethyl)-5-oxo-1-phenyl-2-thioxoimidazolidin-4-yl)ethylidene)hydrazinecarboxylate (4j)**: reaction time: 13.5 h; yield 58%, (253.2 mg); white powder from DCM; m.p. 163-164 °C; ^{1}H NMR (400 MHz, DMSO-d_6): δ 1.47 (s, 9H, Bu^t), 1.81 (s, 3H, CH_3), 3.30 (s, 3H, OCH_3), 3.33 (s, 3H, OCH_3), 3.55 (dd, ^2J = 14.4 Hz, ^3J = 6.0 Hz, 1H, NCH_aH_b), 4.07 (dd, ^2J = 14.4 Hz, ^3J = 4.4 Hz, 1H, NCH_aH_b,), 4.69 (t, 1H, *J*= 5.2 Hz, CH), 5.10 (s, 1H, CH), 7.33 (d, *J* = 7.2 Hz, 2H, Ar), 7.44-7.53 (m, 3H, Ar), 10.08 (brs, 1H, NH, D_2O exch.); ^{13}C NMR (100 MHz, DMSO-d_6): δ 12.7, 28.0, 47.1, 53.7, 53.8, 69.8, 79.8, 100.8, 128.6, 128.8, 128.9, 133.5, 143.1, 152.7, 169.9, 182.7; HRMS (ESI) calcd. for $C_{20}H_{29}N_4O_5S$ [M + H^+]: 437.1853; found: 437.1865.

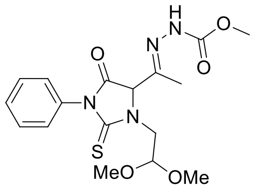

Methyl 2-(1-(3-(2,2-dimethoxyethyl)-5-oxo-1-phenyl-2-thioxoimidazolidin-4-yl)ethylidene)hydrazinecarboxylate (4k): reaction time: 2 h; yield 73%, (287.9 mg); white solid from DCM; m.p 125-126 °C; ^{1}H NMR (400 MHz, , DMSO-d_6): δ 1.81 (s, 3H, CH_3), 3.29 (s, 3H, OCH_3), 3.33 (s, 3H, OCH_3), 3.60 (dd, ^2J = 14.4 Hz, ^3J = 5.6 Hz, 1H, NCH_aH_b), 3.70 (s, 3H, OCH_3), 4.00 (dd, ^2J = 14.4 Hz, ^3J = 4.8 Hz, 1H, NCH_aH_b), 4.69 (t, *J* = 5.2 Hz, 1H, CH), 5.10 (s, 1H, CH), 7.33 (d, *J* = 6.8 Hz, 2H, Ar), 7.45-7.54 (m, 3H, Ar), 10.41 (s, 1H, NH, D_2O exch.); ^{13}C NMR (100 MHz, DMSO-d_6): δ 12.6, 47.3, 52.1, 53.7, 53.9, 69.9, 100.8, 128.6, 129.9, 129.0, 133.5, 144.0, 154.4, 169.9, 182.9; HRMS (ESI) calcd. for $C_{17}H_{23}N_4O_5S$ [M + H^+]: 395.1384; found: 395.1391.

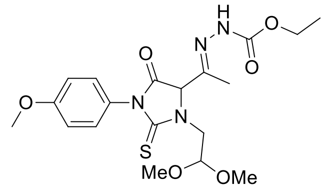

Ethyl 2-(1-(3-(2,2-dimethoxyethyl)-1-(4-methoxyphenyl)-5-oxo-2-thioxoimidazolidin-4-yl)ethylidene)hydrazinecarboxylate (4l): reaction time: 8 h; yield 58%, (254.3 mg); white solid from DCM; m.p 155 °C; ^{1}H NMR (400 MHz, , DMSO-d_6): δ 1.24 (t, *J* = 7.2 Hz, 3H, OCH_2CH_3), 1.80 (s, 3H, CH_3), 3.28 (s, 3H, OCH_3), 3.32 (s, 3H, OCH_3), 3.57 (dd, ^2J = 14.0 Hz, ^3J = 5.6 Hz, 1H, NCH_aH_b), 3.80 (s, 3H, OCH_3) 4.01 (dd, ^2J = 14.4 Hz, ^3J = 4.8 Hz, 1H, NCH_aH_b), 4.16 (q, *J* = 7.2 Hz, 3H, OCH_2CH_3), 4.68 (t, *J* = 5.2 Hz, 1H, CH), 5.07 (s, 1H, CH), 7.03 (d, *J* = 9.2 Hz, 2H, Ar), 7.23 (d, *J* = 8.8 Hz, 2H, Ar), 10.35 (s, 1H, NH, D_2O exch.); ^{13}C NMR (100 MHz, DMSO-d_6): δ 12.6, 14.5, 47.3, 53.7, 53.8, 55.4, 60.7, 69.7, 100.8, 114.1, 126.0, 129.8, 143.9, 153.6, 159.4, 170.1, 183.3; HRMS (ESI) calcd. for $C_{19}H_{27}N_4O_6S$ [M + H^+]: 439.1646; found: 439.1631.

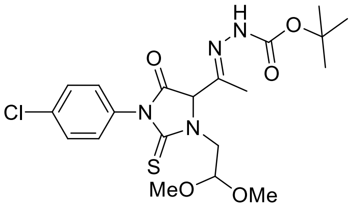

***tert*-Butyl 2-(1-(1-(4-chlorophenyl)-3-(2,2-dimethoxyethyl)-5-oxo-2-thioxoimidazolidin-4-yl)ethylidene)hydrazine-carboxylate (4m)**[3]:

The characterization data of **4m** are available at: https://chemistry-europe.onlinelibrary.wiley.com/action/downloadSupplement?doi=10.1002%2Fejoc.202201053&file=ejoc202201053-sup-0001-misc_information.pdf.

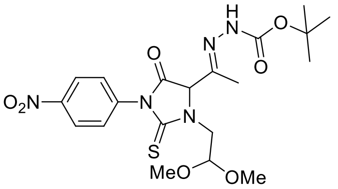

***tert*-Butyl 2-(1-(3-(2,2-dimethoxyethyl)-1-(4-nitrophenyl)-5-oxo-2-thioxoimidazolidin-4-yl)ethylidene)hydrazine-carboxylate (4n)**:

reaction time: 14.5 h; yield 63% (303.3 mg); beige powder from DCM/light petroleum ether; m.p. 150-154 °C dec.; ^{1}H NMR (400 MHz, DMSO-*d$_6$*): δ 1.45 and 1.47 (2s, 9H, Bu^t), 1.83 (s, 3H, CH_3), 3.31 (s, 3H, OCH_3), 3.34 (s, 3H, OCH_3), 3.56 (dd, ^2J = 14.4 Hz, ^3J = 6.4 Hz, 1H, NCH_aH_b), 4.10 (dd, ^2J = 14.4 Hz, ^3J = 4.4 Hz, 1H, NCH_aH_b,), 4.68 (t, *J* = 5.6 Hz, 1H, CH), 5.12 (s, 1H, CH), 7.71 (d, *J* = 9.2 Hz, 2H, Ar), 8.37 (d, *J* = 8.8 Hz, 2H, Ar), 10.09 (s, 1H, NH, D_2O exch.); ^{13}C NMR (100 MHz, DMSO-*d$_6$*): δ 12.9, 27.9, 28.0, 47.1, 53.8, 69.9, 79.8, 100.8, 124.0, 130.1, 138.9, 147.2, 152.7, 169.5 181.6; HRMS (ESI) calcd. for $C_{20}H_{28}N_5O_7S$ [M + H$^+$]: 482.1704; found: 482.1716.

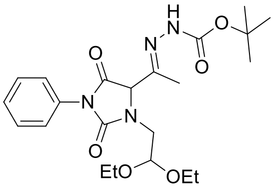

***tert*-Butyl 2-(1-(3-(2,2-diethoxyethyl)-2,5-dioxo-1-phenylimidazolidin-4-yl)ethylidene)hydrazine-carboxylate (4o)**:

reaction time: 25 h; yield 30%, (134.5 mg); white powder from Et_2O/light petroleum ether; m.p. 124-125 °C; ^{1}H NMR (400 MHz, DMSO-*d$_6$*): δ 1.09-1.14 (m, 6H, 2 x OCH_2CH_3), 1.46 (s, 9H, Bu^t), 1.80 (s, 3H, CH_3), 3.23 (dd, ^2J = 14.4 Hz, ^3J = 5.6 Hz, 1H, NCH_aH_b), 3.45-3.64 (m, 5H, 2 x OCH_2CH_3 and NCH_aH_b), 4.64 (t, 1H, *J* = 5.6 Hz, CH), 4.88 (s, 1H, CH),), 7.36-7.44 (m, 3H, Ar), 7.50 (t, *J* = 7.2 Hz, 2H, Ar), 9.99 (s, 1H, NH, D_2O exch.); ^{13}C NMR (100 MHz, DMSO-*d$_6$*): δ 12.4, 15.1, 15.2, 28.0, 44.0, 61.6, 61.7, 67.2, 79.6, 99.4, 126.4, 128.2, 128.8, 131.6, 144.0, 152.7, 155.0, 168.7; HRMS (ESI) calcd. for $C_{22}H_{32}N_4O_6Na$ [M + Na$^+$]; 471.2214; found: 471.2216.

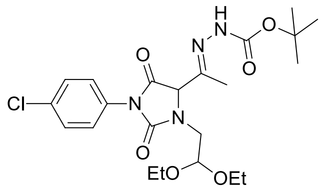

***tert*-Butyl 2-(1-(1-(4-chlorophenyl)-3-(2,2-diethoxyethyl)-2,5-dioxoimidazolidin-4-yl)ethylidene)hydrazinecarboxylate (4p)**: reaction time: 18 h; yield 56%, (270.5 mg); white powder from Et_2O /Petroleum ether; m.p. 147-148 °C; ^1H NMR (400 MHz, DMSO-d_6): δ 1.09-1.14 (m, 6H, 2 x OCH_2CH_3), 1.46 (s, 9H, Bu^t), 1.80 (s, 3H, CH_3), 3.23 (dd, ^2J = 14.8 Hz, ^3J = 5.6 Hz, 1H, NCH_aH_b,), 3.45-3.64 (m, 5H, 2 x OCH_2CH_3 and NCH_aH_b), 4.63 (t, 1H, J = 5.2 Hz, CH), 4.88 (s, 1H, CH),), 7.43 (d, J = 8.8 Hz, 2H, Ar), 7.58 (d, J = 8.8 Hz, 2H, Ar), 10.00 (s, 1H, NH, D_2O exch.); ^{13}C NMR (100 MHz, DMSO-d_6): δ 12.4, 15.1, 15.2, 28.0, 44.1, 61.5, 61.7, 67.1, 79.6, 99.4, 128.2, 128.9, 130.5, 132.5, 143.9, 152.7, 154.9, 168.6; HRMS (ESI) calcd. for $C_{22}H_{32}ClN_4O_6$ [M + H^+]: 483.2005; found: 483.2009.

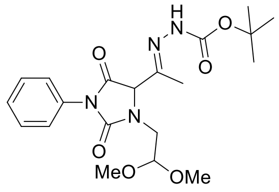

***tert*-Butyl 2-(1-(3-(2,2-dimethoxyethyl)-2,5-dioxo-1-phenylimidazolidin-4-yl)ethylidene)hydrazinecarboxylate (4q)**: reaction time: 22 h; yield 48%, (201.8 mg); white powder from Et_2O/Petroleum ether; m.p. 151-152 °C; ^1H NMR (400 MHz, DMSO-d_6): δ 1.48 (s, 9H, Bu^t), 1.82 (s, 3H, CH_3), 3.28 (s, 3H, OCH_3), 3.31 (s, 3H, OCH_3), 3.34 (d, J = 3.6 Hz, 1H, NCH_aH_b), 3.53 (dd, ^2J = 14.4 Hz, ^3J = 4.8 Hz, 1H, NCH_aH_b), 4.52 (t, 1H, J = 5.2 Hz, CH), 4.90 (s, 1H, CH), 7.38-7.45 (m, 3H, Ar), 7.51 (t, J = 7.2 Hz, 2H, Ar), 10.02 (s, 1H, NH, D_2O exch.); ^{13}C NMR (100 MHz, DMSO-d_6): δ 12.4, 28.0, 43.2, 53.3, 53.4, 67.2, 79.7, 100.2, 126.6, 128.2, 128.9, 131.8, 144.4, 152.8, 155.2, 168.7; HRMS (ESI) calcd. for $C_{20}H_{29}N_4O_6$ [M + H^+]: 421.2082; found: 421.2075.

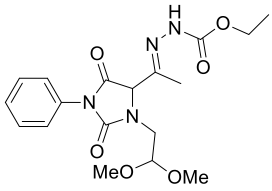

Ethyl 2-(1-(3-(2,2-dimethoxyethyl)-2,5-dioxo-1-phenylimidazolidin-4-yl)ethylidene)hydrazinecarboxylate (4r): reaction time: 30 h; yield 45% (176.6 mg); white powder from Et_2O/light petroleum ether; m.p 125-126 °C; ^1H NMR (400 MHz, DMSO-d_6): δ 1.24 (t, J = 7.2 Hz, 3H, OCH_2CH_3), 1.82 (s, 3H, CH_3), 3.26 (s, 3H, OCH_3), 3.29 (s, 3H, OCH_3), 3.33 (d, J = 6.0 Hz, 1H, NCH_aH_b), 3.48 (dd, ^2J = 14.8 Hz, ^3J = 5.2 Hz, 1H, NCH_aH_b), 4.16 (q, J = 7.2 Hz, 2H, OCH_2CH_3), 4.50 (t, J = 5.2 Hz, 1H, CH), 4.90 (s, 1H, CH), 7.37-7.44 (m, 3H, Ar), 7.50 (t, J = 7.2 Hz, 2H, Ar), 10.29 (s, 1H, NH, D_2O exch.); ^{13}C NMR (100 MHz, DMSO-d_6): δ 12.3, 14.5, 43.3, 53.3, 53.4, 60.7, 67.3, 101.1, 128.6, 128.2, 128.9, 131.7, 145.1, 153.9, 155.3, 168.7; HRMS (ESI) calcd. for $C_{18}H_{25}N_4O_6$ [M +H^+]: 393.1769; found: 393.1781.

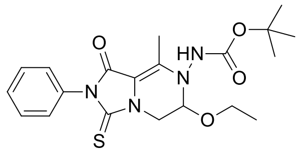

tert-Butyl (6-ethoxy-8-methyl-1-oxo-2-phenyl-3-thioxo-2,3,5,6-tetrahydroimidazo[1,5-a]pyrazin-7(1H)-yl)carbamate (5a): reaction time: 0.5 h; yield 73%, (305.5 mg); light yellow powder from EtOAc/cyclohexane; m.p 174-175 °C; ^{1}H NMR (400 MHz, DMSO-d_6): δ 1.14 (t, J = 7.2 Hz, 3H, OCH_2CH_3), 1.43 and 1.45 (2 s, 9H, Bu^t), 2.25 (s, 3H, CH_3), 3.57-3.67 (m, 2H, $OCH_aH_bCH_3$ and NCH_aH_b), 3.72-3.80 (m, 1H, $OCH_aH_bCH_3$), 4.50 (d, J = 13.2 Hz, 1H, NCH_aH_b), 5.04 (brs, 1H, CH), 7.33 (d, J = 6.8 Hz, 2H, Ar), 7.41-7.51 (m, 3H, Ar), 9.83 and 10.17 (2 brs, 1H, NH, D_2O exch.); ^{13}C NMR (100 MHz, DMSO-d_6): δ 11.4, 14.9, 27.9, 45.8, 63.7, 80.8, 86.9, 105.6, 128.3, 128.6, 128.7, 139.3, 154.9, 160.4, 168.8; HRMS (ESI) calcd. for $C_{20}H_{27}N_4O_4S$ [M + H^+]: 419.1748; found: 419.1747.

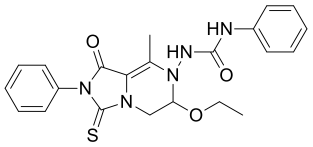

1-(6-Ethoxy-8-methyl-1-oxo-2-phenyl-3-thioxo-2,3,5,6-tetrahydroimi-dazo[1,5-a]pyrazin-7(1H)-yl)-3-phenylurea (5b): reaction time: 1.5 h; yield 61%, (266.9 mg); light yellow powder from EtOAc/cyclohexane; m.p. 152-153 °C; ^{1}H NMR (400 MHz, DMSO-d_6): δ 1.18 (t, J = 7.2 Hz, 3H, OCH_2CH_3), 2.34 (s, 3H, CH_3), 3.67-3.90 (m, 3H, OCH_2CH_3 and NCH_aH_b), 4.50 (d, J = 12.8 Hz, 1H, NCH_aH_b), 5.11 (t, J = 2.4 Hz, 1H, CH), 7.01 (t, J = 7.2 Hz, 1H, Ar), 7.27-7.34 (m, 4H, Ar), 7.42-7.52 (m, 5H, Ar), 8.90 (s, 1H, NH, D_2O exch.), 9.26 (brs, 1H, NH, D_2O exch.); ^{13}C NMR (100 MHz, DMSO-d_6): δ 11.6, 15.1, 45.7, 64.3, 86.9, 105.9, 119.2, 122.5, 128.4, 128.6, 128.7, 133.9, 139.0, 140.8, 154.5, 160.4, 168.6; HRMS (ESI) calcd. for $C_{22}H_{24}N_5O_3S$ [M +H^+]:438.1594; found: 438.1607.

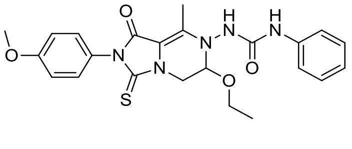

1-(6-Ethoxy-2-(4-methoxyphenyl)-8-methyl-1-oxo-3-thioxo-2,3,5,6-tetrahydroimidazo[1,5-a]pyrazin-7(1H)-yl)-3-phenylurea (5c): reaction time: 2.5 h; yield 57%, (266.5 mg); dark yellow powder from EtOAc/cyclohexane; m.p. 158-159 °C; ^{1}H NMR (400 MHz, DMSO-d_6): δ 1.17 (t, J = 6.8 Hz, 3H, OCH_2CH_3), 2.33 (s, 3H, CH_3), 3.64-3.71 (m, 1H, $OCH_aH_bCH_3$), 3.78-3.88 (m, 5H, OCH_3, $OCH_aH_bCH_3$ and NCH_aH_b), 4.48 (d, J = 13.2 Hz, 1H, NCH_aH_b), 5.10 (brs, 1H, CH), 6.99-7.04 (m, 3H, Ar), 7.22 (d, J = 8.8 Hz, 2H, Ar), 7.28 (t, J = 8.4 Hz, 2H, Ar), 7.48 (d, J = 8.0 Hz, 2H, Ar), 8.91 (s, 1H, NH, D_2O exch.), 9.27 (brs, 1H, NH, D_2O exch.); ^{13}C NMR (100 MHz, DMSO-d_6): δ 11.6, 15.1, 45.7, 52.3, 64.3, 86.9, 105.9, 113.9, 119.2, 122.5, 126.5, 128.6, 129.8 139.0, 140.5, 154.5, 159.0, 160.7, 169.0; HRMS (ESI) calcd. for $C_{23}H_{26}N_5O_4S$ [M +H^+]: 468.1700; found:468.1716.

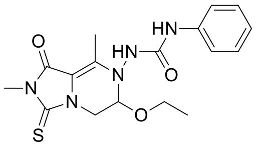

1-(6-Ethoxy-2,8-dimethyl-1-oxo-3-thioxo-2,3,5,6-tetrahydroimidazo[1,5-*a*]pyrazin-7(1*H*)-yl)-3-phenylurea (5d): reaction time: 4 h; yield 54%, (202.7 mg); yellow powder from EtOAc/cyclohexane; m.p. 194-195 °C; ^{1}H NMR (400 MHz, DMSO-d_6): δ 1.14 (t, *J* = 7.2 Hz, 3H, OCH$_2$CH_3), 2.31 (s, 3H, CH$_3$), 3.16 (s, 3H, CH$_3$), 3.59-3.67 (m, 1H, OCH_aH$_b$CH$_3$), 3.72-3.80 (m, 2H, OCH$_a$$H_bCH_3$ and NCH_aH$_b$), 4.41 (d, *J* = 12.8 Hz, 1H, NCH$_a$$H_b$), 5.04 (t, *J* = 2.4 Hz, 1H, CH), 6.99 (t, *J* = 7.6 Hz, 1H, Ar), 7.27 (t, *J* = 7.6 Hz, 2H, Ar), 7.46 (d, *J* = 7.6 Hz, 2H, Ar), 8.86 (s, 1H, NH, D$_2$O exch.), 9.19 (brs, 1H, NH, D$_2$O exch.); ^{13}C NMR (100 MHz, DMSO-d_6): δ 11.6, 15.0, 27.3, 45.4, 64.1, 86.8, 106.0, 119.1, 122.5, 128.6, 139.0, 140.2, 154.5, 160.8, 168.9; HRMS (ESI) calcd. for $C_{17}H_{22}N_5O_3S$ [M + H$^+$]: 376.1438; found: 376.1447.

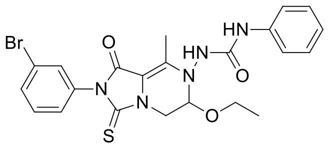

2-(2-(3-Bromophenyl)-6-ethoxy-8-methyl-1-oxo-3-thioxo-2,3,5,6-tetrahydroimidazo[1,5-*a*]pyrazin-7(1*H*)-yl)-*N*-phenylacetamide (5e): reaction time: 4 h; yield 58%, (299.5 mg); white powder from EtOAc/cyclohexane; m.p. 119-120 °C; ^{1}H NMR (400 MHz, DMSO-d_6): δ 1.18 (t, *J* = 6.8 Hz, 3H, OCH$_2$CH_3), 2.34 (s, 3H, CH$_3$), 3.65-3.72 (m, 1H, OCH_aH$_b$CH$_3$), 3.78-3.88 (m, 2H, OCH$_a$$H_bCH_3$ and NCH_aH$_b$), 4.49 (d, *J* = 12.8 Hz, 1H, NCH$_a$$H_b$), 5.11 (brs, 1H, CH), 7.01 (t, *J* = 7.2 Hz, 1H, Ar), 7.29 (t, *J* = 7.6 Hz, 2H, Ar), 7.39 (d, *J* = 8.0 Hz, 1H, Ar), 7.47 (t, *J* = 8.0 Hz, 3H, Ar), 7.62 (s, 1H, Ar), 7.65 (d, *J* = 8.0 Hz, 1H, Ar), 8.91 (s, 1H, NH, D$_2$O exch.), 9.27 (brs, 1H, NH, D$_2$O exch.); ^{13}C NMR (100 MHz, DMSO-d_6): δ 11.7, 15.1, 45.7, 64.3, 86.9, 105.6, 119.1, 120.9, 122.5, 128.0, 128.6, 130.5, 131.3, 131.4, 135.3, 139.0, 141.2, 154.4, 160.1, 168.1; HRMS (ESI) calcd. for $C_{22}H_{23}BrN_5O_3S$ [M + H$^+$]: 516.0699; found: 516.0712.

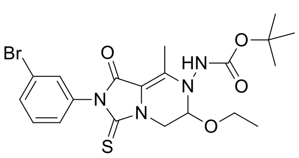

***tert*-Butyl (2-(3-bromophenyl)-6-ethoxy-8-methyl-1-oxo-3-thioxo-2,3,5,6-tetrahydroimidazo[1,5-*a*]pyrazin-7(1*H*)-yl)carbamate (5f)**[4]: reaction time: 2.5 h; yield 82%, (407.9 mg); white powder from EtOAc/cyclohexane; m.p. 180-181 °C; ^{1}H NMR (400 MHz, DMSO-d_6): δ 1.14 (t, *J* = 7.2 Hz, 3H, OCH$_2$CH_3), 1.45 (s, 9H, Bu^t), 2.25 (s, 3H, CH$_3$), 3.59-3.67 (m, 2H, OCH_aH$_b$CH$_3$ and NCH_aH$_b$), 3.72-3.79 (m, 1H, OCH$_a$$H_bCH_3$), 4.49 (d, *J* = 13.2 Hz, 1H, NCH$_a$$H_b$), 5.04 (brs, 1H, CH), 7.39 (d, *J* = 8.0 Hz, 1H, Ar), 7.46 (t, *J* = 8.0 Hz, 1H, Ar), 7.62-7.65 (m, 2H, Ar), 9.82 and 10.15 (2 brs, 1H, NH, D$_2$O exch.); ^{13}C NMR (100 MHz, DMSO-d_6): δ 11.5, 14.9, 27.9, 45.9, 63.8, 81.9, 86.9, 105.5, 120.9, 128.0, 130.5, 131.3, 131.5, 135.3, 139.8, 155.0, 160.1, 168.4; HRMS (ESI) calcd. for $C_{20}H_{26}BrN_4O_4S$ [M + H$^+$]: 497.0853; C; found: 497.0841.

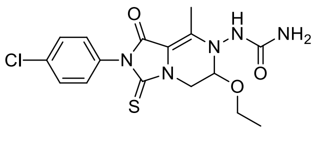

1-(6-Ethoxy-2-(4-chlorophenyl)-8-methyl-1-oxo-3-thioxo-2,3,5,6-tetrahydroimidazo[1,5-*a*]pyrazin-7(1*H*)-yl)urea (5g): reaction time: 3.5 h; yield 51.0%, (201.9 mg); light yellow powder from EtOAc/cyclohexane; m.p. 188-189 °C; ^{1}H NMR (400 MHz, DMSO-d_6): δ 1.15 (t, J = 7.2 Hz, 3H, OCH$_2$CH_3), 2.28 (s, 3H, CH$_3$), 3.60-3.68 (m, 1H, OCH_aH$_b$CH$_3$), 3.74-3.82 (m, 1H, OCH$_a$$H_bCH_3$), 3.86 (d, J = 12.0 Hz, 1H, NCH_aH$_b$), 4.44 (d, J = 13.2 Hz, 1H, NCH$_a$$H_b$), 5.00 (brs, 1H, CH), 6.33 (brs, 2H, NH$_2$, D$_2$O exch.), 7.37 (d, J = 8.8 Hz, 2H, Ar), 7.56 (d, J = 8.8 Hz, 2H, Ar), 9.00 (brs, 1H, NH, D$_2$O exch.); ^{13}C NMR (100 MHz, DMSO-d_6): δ 11.6, 15.1, 45.9, 64.4, 86.8, 105.4, 128.8, 130.5, 132.7, 132.9, 141.4, 157.7, 160.1, 167.9; HRMS (ESI) calcd. for $C_{16}H_{19}ClN_5O_3S$ [M + H$^+$]: 396.0892; found: 396.0905.

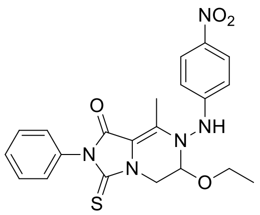

6-Ethoxy-8-methyl-7-((4-nitrophenyl)amino)-2-phenyl-3-thioxo-2,3,6,7-tetrahydroimidazo[1,5-*a*]pyrazin-1(5*H*)-one (5h): reaction time: 3.0 h; 62 %, (272.5 mg); dark orange powder from EtOAc/light petroleum ether; m.p 184-187 °C dec.; ^{1}H NMR (400 MHz, , DMSO-d_6): δ 1.19 (t, J = 6.8 Hz, 3H, OCH$_2$CH_3), 2.27 (s, 3H, CH$_3$), 3.65-3.73 (m, 1H, OCH_aH$_b$CH$_3$), 3.77-3.85 (m, 1H, OCH$_a$$H_bCH_3$), 4.05 (d, J = 14.4 Hz, 1H, NCH_aH$_b$), 4.61 (d, J = 13.6 Hz, 1H, NCH$_a$$H_b$), 5.10 (s, 1H, CH), 6.97 (d, J = 9.2 Hz, 2H, Ar), 7.33 (d, J = 7.2 Hz, 2H, Ar), 7.44 (t, J = 7.2 Hz, 1H, Ar), 7.51 (t, J = 7.6 Hz, 2H, Ar), 8.13 (d, J = 8.8 Hz, 2H, Ar), 9.82 (s, 1H, NH, D$_2$O exch.); ^{13}C NMR (100 MHz, DMSO-d_6): δ 11.3, 15.1, 45.8, 64.4, 85.1, 106.2, 110.9, 126.2, 128.4, 128.6, 128.7, 133.8, 139.3, 140.1, 152.7, 160.3, 168.8; HRMS (ESI) calcd. for $C_{21}H_{22}N_5O_4S$ [M + H$^+$]: 440.1387; found: 440.1398.

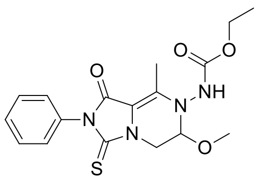

Ethyl (6-methoxy-8-methyl-1-oxo-2-phenyl-3-thioxo-2,3,5,6-tetrahydroimidazo[1,5-*a*]pyrazin-7(1*H*)-yl)carbamate (5i): reaction time: 0.5 h; yield 62%, (233.4 mg); orange powder from EtOAc/cyclohexane; m.p 144-145 °C; ^{1}H NMR (400 MHz, , DMSO-d_6): δ 1.24 (t, J = 6.8 Hz, 3H, OCH$_2$CH_3), 2.27 (s, 3H, CH$_3$), 3.41 (s, 3H, OCH$_3$), 3.61 (d, J = 12.4 Hz, 1H, NCH_aH$_b$), 4.15 (q, J = 7.2 Hz, 2H, OCH_2CH$_3$), 4.58 (d, J = 12.8 Hz, 1H, NCH$_a$$H_b$), 5.00 (brs, 1H, CH), 7.33 (d, J = 8.4 Hz, 2H, Ar), 7.41-7.51 (m, 3H, Ar), 10.42 (brs, 1H, NH, D$_2$O exch.); ^{13}C NMR (100 MHz, DMSO-d_6): δ 11.4, 14.3, 45.4, 55.7, 61.5, 88.4, 105.8, 128.3, 128.6, 128.7, 133.8, 138.8, 155.9, 160.4, 169.1; HRMS (ESI) calcd. for $C_{17}H_{21}N_4O_4S$ [M + H$^+$]: 377.1278; found: 377.1294.

tert-Butyl (6-methoxy-8-methyl-1-oxo-2-phenyl-3-thioxo-2,3,5,6-tetrahydroimidazo[1,5-_a_]pyrazin-7(1_H_)-yl)carbamate (5j)[4]:

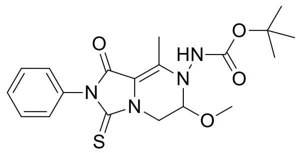

reaction time: 0.5 h; yield 67%, (271.0 mg); yellow powder from EtOAc/Et_2O; m.p. 187-188 °C; ^{1}H NMR (400 MHz, DMSO-d_6): δ 1.46 (s, 9H, Bu^t), 2.26 (s, 3H, CH_3), 3.40 (s, 3H, OCH_3), 3.61 (d, *J* = 12.0 Hz, 1H, NC$H_a$$H_b$), 4.56 (d, *J* = 13.2 Hz, 1H, NC$H_a$$H_b$), 4.96 (brs, 1H, CH), 7.32 (d, *J* = 8.4 Hz, 2H, Ar), 7.40-7.51 (m, 3H, Ar), 9.85 and 10.20 (2 brs, 1H, NH, D_2O exch.); ^{13}C NMR (100 MHz, DMSO-d_6): δ 11.4, 27.9, 45.5, 55.7, 80.9, 88.6, 105.8, 128.3, 128.6, 128.7, 133.8, 138.9, 155.0, 160.4, 169.0; HRMS (ESI) calcd. for $C_{19}H_{25}N_4O_4S$ [M + H^+]: 405.1591; found: 405.1607.

Methyl (6-methoxy-8-methyl-1-oxo-2-phenyl-3-thioxo-2,3,5,6-tetrahydroimidazo[1,5-_a_]pyrazin-7(1_H_)-yl)carbamate (5k)[4]:

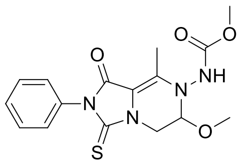

reaction time: 1.5 h; yield 79%, (286.3 mg); yellow powder from EtOAc/cyclohexane; m.p 165-166 °C; ^{1}H NMR (400 MHz, , DMSO-d_6): δ 2.27 (s, 3H, CH_3), 3.41 (s, 3H, OCH_3), 3.62 (d, *J* = 13.2 Hz, 1H, NC$H_a$$H_b$), 3.70 (s, 3H, OCH_3), 4.58 (d, *J* = 13.2 Hz, 1H, NC$H_a$$H_b$), 5.01 (brs, 1H, CH), 7.32 (d, *J* = 7.2 Hz, 2H, Ar), 7.41-7.51 (m, 3H, Ar), 10.15 and 10.45 (2 brs, 1H, NH, D_2O exch.); ^{13}C NMR (100 MHz, DMSO-d_6): δ 11.6, 45.4, 52.8, 55.8, 88.6 105.8, 128.4, 128.7, 128.8, 133.8, 139.1, 156.6, 160.4, 169.1; HRMS (ESI) calcd. for $C_{16}H_{19}N_4O_4S$ [M + H^+]: 363.1122; found: 363.1106.

Ethyl (6-methoxy-2-(4-methoxyphenyl)-8-methyl-1-oxo-3-thioxo-2,3,5,6-tetrahydroimidazo[1,5-_a_]pyrazin-7(1_H_)-yl)carbamate (5l)[4]:

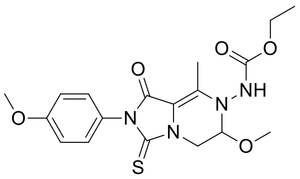

reaction time: 2.5 h; yield 81.0%, (329.2 mg); pale yellow powder from EtOAc/cyclohexane; m.p 154-155 °C; ^{1}H NMR (400 MHz, , DMSO-d_6): δ 1.23 (t, *J* = 6.8 Hz, 3H, OCH_2CH_3), 2.26 (s, 3H, CH_3), 3.40 (s, 3H, OCH_3), 3.60 (d, *J* = 12.0 Hz, 1H, NC$H_a$$H_b$), 3.80 (s, 3H, OCH_3) 4.14 (q, *J* = 7.2 Hz, 3H, OC$H_2$$CH_3$), 4.57 (d, *J* = 12.8 Hz, 1H, NC$H_a$$H_b$), 4.99 (brs, 1H, CH), 7.01 (d, *J* = 9.2 Hz, 2H, Ar), 7.22 (d, *J* = 8.8 Hz, 2H, Ar), 10.40 (brs, 1H, NH, D_2O exch.); ^{13}C NMR (100 MHz, DMSO-d_6): δ 11.4, 14.4, 45.4, 55.3, 55.7, 61.6, 88.4, 105.9, 113.9, 126.4, 129.8, 138.6, 155.9, 159.0, 160.6, 169.5; HRMS (ESI) calcd. for $C_{18}H_{23}N_4O_5S$ [M + H^+]: 407.1384; found: 407.1397.

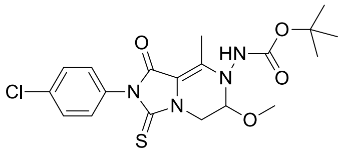

tert-Butyl (2-(4-chlorophenyl)-6-methoxy-8-methyl-1-oxo-3-thioxo-2,3,5,6-tetrahydroimidazo[1,5-_a_]pyrazin-7(1_H_)-yl)carbamate (5m)[4]:
reaction time: 1 h; yield 70%, (307.2 mg); yellow powder from EtOAc/Et_2O m.p. 184-188 °C dec.; ^{1}H NMR (400 MHz, DMSO-d_6): δ 1.39 and 1.45 (2 s, 9H, Bu^t), 2.26 (s, 3H, CH_3), 3.40 (s, 3H, OCH_3), 3.62 (d, J = 12.8 Hz, 1H, NCH_aH$_b$), 4.57 (d, J = 12.8 Hz, 1H, NCH$_a$$H_b$), 4.96 (s, 1H, CH), 7.39 (d, J = 8.8 Hz, 2H, Ar), 7.55 (d, J = 8.8 Hz, 2H, Ar), 9.87 and 10.20 (2 brs, 1H, NH, D_2O exch.); ^{13}C NMR (100 MHz, DMSO-d_6): δ 11.5, 27.9, 45.5, 55.7, 80.9, 88.6, 105.7, 128.7, 130.5, 132.7, 132.9, 139.3, 155.0, 160.2, 168.6; HRMS (ESI) calcd. for $C_{19}H_{24}ClN_4O_4S$ [M + H$^+$]: 439.1201; found: 439.1211.

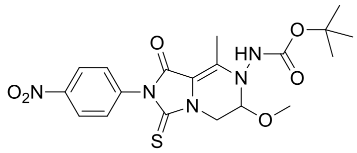

tert-Butyl (6-methoxy-8-methyl-2-(4-nitrophenyl)-1-oxo-3-thioxo-2,3,5,6-tetrahydroimidazo[1,5-_a_]pyrazin-7(1_H_)-yl)carbamate (5n)[4]:
reaction time: 3 h; yield 75%, (337.1 mg); orange powder from DCM/light petroleum ether; m.p. 190-193 °C dec.; ^{1}H NMR (400 MHz, DMSO-d_6): δ 1.40 and 1.46 (s, 9H, Bu^t), 2.27 (s, 3H, CH_3), 3.41 (s, 3H, OCH_3), 3.64 (d, J = 14.4 Hz, 1H, NCH_aH$_b$), 4.58 (d, J = 12.8 Hz, 1H, NCH$_a$$H_b$), 4.98 (s, 1H, CH), 7.72 (d, J = 9.2 Hz, 2H, Ar), 8.34 (d, J = 9.2 Hz, 2H, Ar), 9.90 and 10.24 (2 brs, 1H, NH, D_2O exch.); ^{13}C NMR (100 MHz, DMSO-d_6): δ 11.5, 26.3, 27.9, 45.5, 55.8, 81.0, 88.5, 105.5, 123.8, 129.9, 139.4, 139.9, 146.7, 154.9, 159.8, 167.8; HRMS (ESI) calcd. for $C_{19}H_{25}N_5O_6S$ [M + H$^+$]: 450.1442; found: 450.1445.

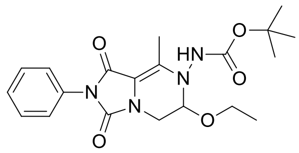

tert-Butyl (6-ethoxy-8-methyl-1,3-dioxo-2-phenyl-2,3,5,6-tetrahydroimidazo[1,5-_a_]pyrazin-7(1_H_)-yl)carbamate (5o)[4]:
reaction time: 3.5 h; yield 59%, (237.4 mg); orange powder from EtOAc/DCM/Et_2O; m.p. 114-115 °C; ^{1}H NMR (400 MHz, DMSO-d_6): δ 1.14 (t, J = 7.2 Hz, 3H, OCH$_2$CH_3), 1.44 (s, 9H, Bu^t), 2.21 (s, 3H, CH_3), 3.47 (d, J = 11.2 Hz, 1H, NCH_aH$_b$), 3.54-3.61 (m, 1H, OCH_aH$_b$CH$_3$), 3.71-3.78 (m, 1H, OCH$_a$$H_bCH_3$), 4.02 (d, J = 12.0 Hz, 1H, NCH$_a$$H_b$), 4.89 (brs, 1H, CH), 7.35-7.39 (m, 3H, Ar), 7.47 (t, J = 8.4 Hz, 2H, Ar) 9.59 and 9.94 (2 brs, 1H, NH, D_2O exch.); ^{13}C NMR (100 MHz, DMSO-d_6): δ 11.7 14.9, 27.9, 42.3, 63.2, 80.4, 87.2, 104.3, 126.7, 127.4, 128.6, 132.4, 135.4, 149.9, 155.2, 159.9; HRMS (ESI) calcd. for $C_{20}H_{27}N_4O_5$ [M + H$^+$]: 403.1976; found: 403.1990 .

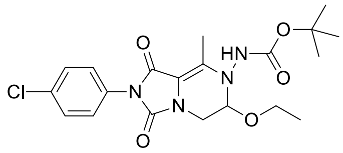

tert-Butyl (2-(4-chlorophenyl)-6-ethoxy-8-methyl-1,3-dioxo-2,3,5,6-tetrahydroimidazo[1,5-*a*]pyrazin-7(1*H*)-yl)carbamate (5p)[4]: reaction time: 2.5 h; yield 67%, (292.7 mg); yellow powder from EtOAc/cyclohexane; m.p. 139-140 °C; ^{1}H NMR (400 MHz, DMSO-d_6): δ 1.14 (t, J = 6.8 Hz, 3H, OCH$_2$CH_3), 1.45 (s, 9H, Bu^t), 2.21 (s, 3H, CH$_3$), 3.47 (d, J = 11.2 Hz, 1H, NCH_aH$_b$), 3.54-3.61 (m, 1H, OCH_aH$_b$CH$_3$), 3.70-3.78 (m, 1H, OCH$_a$$H_bCH_3$), 4.01 (d, J = 11.6 Hz, 1H, NCH$_a$$H_b$), 4.89 (brs, 1H, CH), 7.45 (d, J = 8.8 Hz, 2H, Ar), 7.54 (d, J = 8.4 Hz, 2H, Ar), 9.61 and 9.96 (2 brs, 1H, NH, D$_2$O exch.); ^{13}C NMR (100 MHz, DMSO-d_6): δ 11.7 14.9, 27.9, 42.3, 63.2, 80.5, 87.2, 104.1, 128.3, 128.7, 131.3, 131.8, 135.7, 149.7, 155.2, 159.7; HRMS (ESI) calcd. for $C_{20}H_{26}ClN_4O_5$ [M + H $^+$]: 437.1586; found: 437.1591.

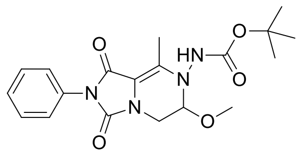

tert-Butyl (6-methoxy-8-methyl-1,3-dioxo-2-phenyl-2,3,5,6-tetrahydroimidazo[1,5-*a*]pyrazin-7(1*H*)-yl)carbamate (5q)[4]: reaction time: 1.5 h; yield 62%, (240.8 mg); yellow powder from DCM/light petroleum ether; m.p. 139-140 °C; ^{1}H NMR (400 MHz, DMSO-d_6): δ 1.44 (s, 9H, Bu^t), 2.21 (s, 3H, CH$_3$), 3.37 (s, 3H, OCH$_3$), 3.43 (d, J = 12.0 Hz, 1H, NCH_aH$_b$), 4.05 (d, J = 12.0 Hz, 1H, NCH$_a$$H_b$), 4.80 (s, 1H, CH), 7.36-7.39 (m, 3H, Ar), 7.47 (t, J = 7.6 Hz, 2H, Ar) 9.63 and 9.98 (2 brs, 1H, NH, D$_2$O exch.); ^{13}C NMR (100 MHz, DMSO-d_6): δ 11.7, 28.0, 42.0, 55.3, 80.6, 88.7, 104.4, 126.8, 127.6, 128.7, 132.3, 135.1, 149.9, 155.3, 160.0; HRMS (ESI) calcd. for $C_{19}H_{25}N_4O_5$ [M + H$^+$]: 389.1819; found: 389.1822.

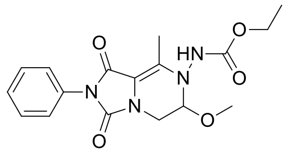

Ethyl (6-methoxy-8-methyl-1,3-dioxo-2-phenyl-2,3,5,6-tetrahydroimidazo[1,5-*a*]pyrazin-7(1*H*)-yl)carbamate (5r)[4]: reaction time: 2 h; yield 42%, (151.3 mg); white powder from EtOAc/Et$_2$O; m.p. 158-160 °C; ^{1}H NMR (400 MHz, DMSO-d_6): δ 1.23 (t, J = 8.0 Hz, 3H, OCH$_2$CH_3), 2.23 (s, 3H, CH$_3$), 3.38 (s, 3H, OCH$_3$), 3.44 (d, J = 11.6 Hz, 1H, NCH_aH$_b$), 4.11-4.13 (m, 3H, NCH$_a$$H_b$ and OCH_2CH$_3$), 4.85 (brs, 1H, CH), 7.36-7.39 (m, 3H, Ar), 7.47 (t, J = 7.6 Hz, 3H, 2H, Ar) 9.88 and 10.20 (2 brs, 1H, NH, D$_2$O exch.); ^{13}C NMR (100 MHz, DMSO-d_6): δ 11.7, 14.4, 41.9, 55.3, 61.3, 88.6, 104.5, 126.8, 127.5, 128.7, 132.3, 134.8, 149.9, 156.2, 160.0; HRMS (ESI) calcd. for $C_{17}H_{21}N_4O_5$ [M + H$^+$]: 361.1506; found: 361.1491.

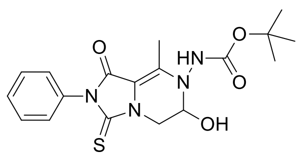

***tert*-Butyl (6-hydroxy-8-methyl-1-oxo-2-phenyl-3-thioxo-2,3,5,6-tetrahydroimidazo[1,5-*a*]pyrazin-7(1*H*)-yl)carbamate (6a)**: reaction time: 0.5 h; yield 8%, (31.2 mg) from **4a**; reaction time: 0.5 h; yield 6%, (23.4 mg) from **5j**; ocher powder from EtOAc/Et_2O; m.p. 168-169 °C; ^{1}H NMR (400 MHz, DMSO-*d*$_6$): δ 1.45 (s, 9H, Bu^t), 2.25 (s, 3H, CH_3), 3.86 (d, *J* = 12.8 Hz, 1H, NCH_aH_b), 4.25 (d, *J* = 12.0 Hz, 1H, NCH_aH_b), 5.09 (t, *J* = 3.2 Hz,, 1H, CH), 7.02 (brs, 1H, OH, D_2O exch.) 7.31 (d, *J* = 7.6 Hz, 2H, Ar), 7.42 (t, *J* = 7.2 Hz, 1H, Ar), 7.49 (t, *J* = 7.2 Hz, 2H, Ar), 9.80 (brs, 1H, NH, D_2O exch.); ^{13}C NMR (100 MHz, DMSO-*d*$_6$): δ 11.4, 27.9, 47.5, 80.5, 104.9, 128.2, 128.6, 128.7,134.1 140.8, 154.7, 160.3, 168.2; HRMS (ESI) calcd. for $C_{18}H_{23}N_4O_4S$ [M + H$^+$]: 391.1435; found: 391.1422.

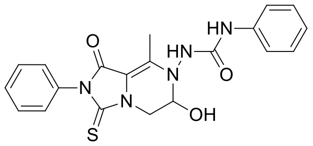

1-(6-Hydroxy-8-methyl-1-oxo-2-phenyl-3-thioxo-2,3,5,6-tetrahydro-imidazo[1,5-*a*]pyrazin-7(1*H*)-yl)-3-phenylurea (6b): reaction time: 1.5 h; yield 25%, (102.3 mg); orange powder from EtOAc/light petroleun ether; m.p. 149-150 °C; ^{1}H NMR (400 MHz, DMSO-*d*$_6$): δ 2.34 (s, 3H, CH_3), 4.07 (d, *J* = 14.4 Hz, 1H, NCH_aH_b), 4.27 (d, *J* = 12.0 Hz, 1H, NCH_aH_b), 5.17 (t, *J* = 2.8 0 Hz, 1H, CH), 7.01 (t, *J* = 7.2 Hz, 1H, Ar), 7.07 (brs, 1H, OH, D_2O exch.), 7.27-7.33 (m, 4H, Ar), 7.43 (t, *J* = 7.2 Hz, 1H, Ar), 7.49-7.52 (m, 4H, Ar), 8.88 (brs, 1H, NH, D_2O exch.), 8.94 (brs, 1H, NH, D_2O exch.); ^{13}C NMR (100 MHz, DMSO-*d*$_6$): δ 11.7, 47.5, 79.5, 105.6, 119.2, 122.5, 128.3, 128.6, 128.7, 134.0, 139.0, 141.6, 154.5, 160.4, 168.1; HRMS (ESI) calcd. for $C_{20}H_{20}N_5O_3S$ [M + H$^+$]: 410.1281; found: 410.1271.

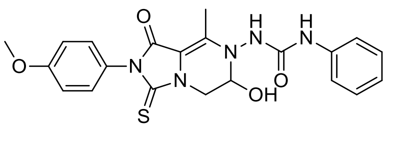

1-(6-Hydroxy-2-(4-methoxyphenyl)-8-methyl-1-oxo-3-thioxo-2,3,5,6-tetrahydroimidazo[1,5-*a*]pyrazin-7(1*H*)-yl)-3-phenylurea (6c): reaction time: 2.5 h; yield 11%, (48.3 mg); light yellow powder from EtOAc/Et_2O; m.p. 159-160°C; ^{1}H NMR (400 MHz, DMSO-*d*$_6$): δ 2.32 (s, 3H, CH_3), 3.80 (s, 3H, OCH_3), 4.05 (d, *J* = 12.0 Hz, 1H, NCH_aH_b), 4.24 (d, *J* = 12.8 Hz, 1H, NCH_aH_b), 5.15 (t, *J* = 2.8 Hz, 1H, CH), 6.98-7.05 (m, 3H, Ar and OH), 7.21 (d, *J* = 9.2 Hz, 2H, Ar), 7.28 (t, *J* = 7.2 Hz, 2H, Ar), 7.50 (d, *J* = 7.6 Hz, 2H, Ar), 8.87 (brs, 1H, NH, D_2O exch.), 8.93 (brs, 1H, NH, D_2O exch.); ^{13}C NMR (100 MHz, DMSO-*d*$_6$): δ 11.7, 47.5, 55.4, 79.5, 105.6, 113.9, 119.2, 122.5, 126.6, 128.6, 129.8 139.1, 141.4, 154.3, 158.9, 160.7, 168.5; HRMS (ESI) calcd. for $C_{21}H_{22}N_5O_4S$ [M + H$^+$]: 440.1387; found: 440.1379.

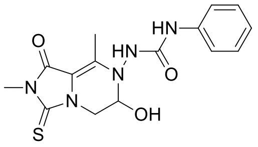

1-(6-Hydroxy-2,8-dimethyl-1-oxo-3-thioxo-2,3,5,6-tetrahydroimidazo[1,5-*a*]pyrazin-7(1*H*)-yl)-3-phenylurea (6d): reaction time: 4 h; yield 22%, (76.4 mg); beige powder from EtOAc/cyclohexane; m.p. 188-192 °C dec.; ^{1}H NMR (400 MHz, DMSO-*d$_6$*): δ 2.30 (s, 3H, CH_3), 3.17 (s, 3H, CH_3), 3.97 (d, J = 11.2 Hz, 1H, $NC\textit{H}_{a}H_{b}$), 4.17 (d, J = 12.8 Hz, 1H, $NCH_{a}\textit{H}_{b}$), 5.08-5.11 (m, 1H, CH), 6.94 (brs, 1H, OH, D_2O exch.), 6.99 (t, J = 7.2 Hz, 1H, Ar), 7.27 (t, J = 7.6 Hz, 2H, Ar), 7.48 (d, J = 7.6 Hz, 2H, Ar), 8.78 (s, 1H, NH, D_2O exch.), 8.89 (brs, 1H, NH, D_2O exch.); ^{13}C NMR (100 MHz, DMSO-*d$_6$*): δ 11.6, 27.3, 47.3, 79.5, 105.8, 119.1, 122.4, 128.6, 139.0, 141.0, 154.6, 160.8, 168.5; HRMS (ESI) calcd. for $C_{15}H_{18}N_5O_3S$ [M + H^+]: 348.1125; found: 348.1134.

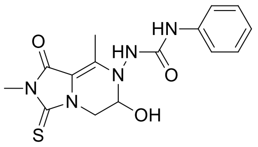

1-(2-(3-Bromophenyl)-6-hydroxy-8-methyl-1-oxo-3-thioxo-2,3,5,6-tetrahydroimidazo[1,5-*a*]pyrazin-7(1*H*)-yl)-3-phenylurea (6e): reaction time: 4 h; yield 16%, (78.1 mg); white powder from EtOAc/light petroleun ether; m.p. 217-218 °C; ^{1}H NMR (400 MHz, DMSO-*d$_6$*): δ 2.33 (s, 3H, CH_3), 4.05 (d, J = 14.0 Hz, 1H, $NC\textit{H}_{a}H_{b}$), 4.24 (d, J = 12.0 Hz, 1H, $NCH_{a}\textit{H}_{b}$), 5.17 (t, J = 2.8 Hz 1H, CH), 7.00 (t, J = 7.2 Hz, 1H, Ar), 7.06 (brs, 1H, OH, D_2O exch.), 7.28 (t, J = 7.6 Hz, 2H, Ar), 7.37 (d, J = 8.0 Hz, 1H, Ar), 7.46-7.50 (m, 3H, Ar), 7.58 (s, 1H, Ar), 7.65 (d, J = 8.4 Hz, 1H, Ar), 8.88 (brs, 1H, NH, D_2O exch.), 8.95 (brs, 1H, NH, D_2O exch.); ^{13}C NMR (100 MHz, DMSO-*d$_6$*): δ 11.6, 47.4, 79.5, 105.4, 119.1, 120.9, 122.5, 128.0, 128.6, 130.6, 131.3, 131.4, 135.4, 138.9, 142.0, 154.4, 160.1, 167.6; HRMS (ESI) calcd. for $C_{20}H_{19}BrN_5O_3S$ [M + H^+]: 488.0386; found: 488.0398.

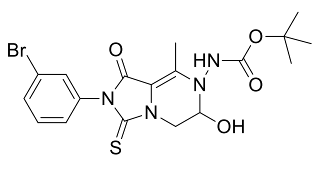

***tert*-Butyl (2-(3-bromophenyl)-6-hydroxy-8-methyl-1-oxo-3-thioxo-2,3,5,6-tetrahydroimidazo[1,5-*a*]pyrazin-7(1*H*)-yl)carbamate (6f)**: reaction time: 2.5 h; yield 12%, (56.3 mg); yellow powder from DCM/light petroleum ether; m.p. 128-129 °C; ^{1}H NMR (400 MHz, DMSO-*d$_6$*): δ 1.45 (s, 9H, Bu^t), 2.26 (s, 3H, CH_3), 3.87 (d, J = 12.8 Hz, 1H, $NCH_{a}\textit{H}_{b}$), 4.25 (d, J = 12.4 Hz, 1H, $NCH_{a}\textit{H}_{b}$), 5.10 (t, J = 3.2 Hz, 1H, CH), 7.02 (brs, 1H, OH, D_2O exch.) 7.38 (d, J = 8.0 Hz, 1H, Ar), 7.47 (t, J = 8.0 Hz, 1H, Ar), 7.59 (s, 1H, Ar), 7.65 (d, J = 8.0 Hz, 1H, Ar), 9.83 (brs, 1H, NH, D_2O exch.); ^{13}C NMR (100 MHz, DMSO-*d$_6$*): δ 11.6, 27.9, 47.4, 79.2, 80.5, 104.8, 120.8, 128.0, 130.5, 131.2, 131.5, 135.5, 141.1, 154.5, 160.1, 167.7; HRMS (ESI) calcd. for $C_{18}H_{22}BrN_4O_4S$ [M + H^+]: 469.0540; found: 469.0527.

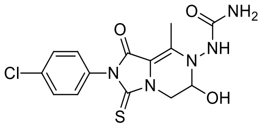

1-(2-(4-Chlorophenyl)-6-hydroxy-8-methyl-1-oxo-3-thioxo-2,3,5,6-tetrahydroimidazo[1,5-*a*]pyrazin-7(1*H*)-yl)urea (6g): reaction time: 3.5 h; yield 28%, (103.0 mg); light yellow powder from EtOAc/Et_2O; m.p. 187-188 °C; ^{1}H NMR (400 MHz, DMSO-*d*$_6$): δ 2.28 (s, 3H, CH_3), 3.97 (d, *J* = 12.8 Hz, 1H, NCH_aH$_b$), 4.27 (d, *J* = 12.4 Hz, 1H, NCH$_a$$H_b$), 5.07 (t, *J* = 2.4 Hz, 1H, CH), 6.39 (brs, 2H, NH_2, D_2O exch.), 6.96 (brs, 1H, OH, D_2O exch), 7.36 (d, *J* = 8.4 Hz, 2H, Ar), 7.56 (d, *J* = 8.4 Hz, 2H, Ar), 8.65 (s, 1H, NH, D_2O exch); ^{13}C NMR (100 MHz, DMSO-*d*$_6$): δ 11.6, 47.5, 79.7, 105.2, 128.7, 130.5, 132.8, 132.9, 142.1, 157.9, 160.1, 167.5; HRMS (ESI) calcd. for $C_{14}H_{15}ClN_5O_3S$ [M + H$^+$]: 368.0579; found: 368.0565.

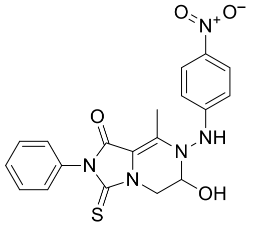

6-Hydroxy-8-methyl-7-((4-nitrophenyl)amino)-2-phenyl-3-thioxo-2,3,6,7-tetrahydroimidazo[1,5-*a*]pyrazin-1(5*H*)-one (6h): reaction time: 3 h; 9%, (39.5 mg); red powder from EtOAc/Et_2O; m.p 184-188 °C dec.; ^{1}H NMR (400 MHz, DMSO-*d*$_6$): δ 2.27 (s, 3H, CH_3), 4.09 (d, *J* = 11.2 Hz, 1H, NCH_aH$_b$), 4.47 (d, *J* = 13.2 Hz, 1H, NCH$_a$$H_b$), 5.11 (d, *J* = 6.0 Hz, 1H, CH), 6.96 (d, *J* = 8.8 Hz, 2H, Ar), 7.16 (d, *J* = 6.4 Hz, 1H, OH, D_2O exch.), 7.32 (d, *J* = 8.0 Hz, 2H, Ar), 7.44 (t, *J* = 7.6 Hz, 1H, Ar), 7.51 (t, *J* = 7.2 Hz, 2H, Ar), 8.12 (d, *J* = 9.2 Hz, 2H, Ar), 9.71 (s, 1H, NH, D_2O exch.); ^{13}C NMR (100 MHz, DMSO-*d*$_6$): δ 11.4, 14.3, 45.4, 55.7, 61.5, 88.4, 105.8, 128.3, 128.6, 128.7, 133.8, 138.8, 155.9, 160.4, 169.1; HRMS (ESI) calcd. for $C_{19}H_{18}N_5O_4S$ [M + H$^+$]: 412.1074; found: 412.1066.

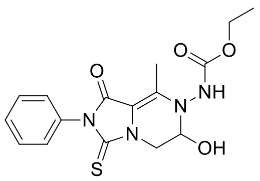

Ethyl (6-hydroxy-8-methyl-1-oxo-2-phenyl-3-thioxo-2,3,5,6-tetrahydroimidazo[1,5-*a*]pyrazin-7(1*H*)-yl)carbamate (6i): reaction time: 0.5 h; yield 22.0%, (79.7 mg); orange powder from EtOAc/Et_2O; m.p 140-141 °C; ^{1}H NMR (400 MHz, DMSO-*d*$_6$): δ 1.22 (t, *J* = 7.2 Hz, 3H, OCH$_2$CH_3), 2.26 (s, 3H, CH_3), 3.89 (d, *J* = 12.8 Hz, 1H, NCH_aH$_b$), 4.13 (q, *J* = 7.2 Hz, 2H, OCH_2CH$_3$), 4.24 (d, *J* = 13.6 Hz, 1H, NCH$_a$$H_b$), 5.12 (t, *J* = 3.2 Hz,, 1H, CH), 7.06 (brs, 1H, OH, D_2O exch.), 7.31 (d, *J* = 8.0 Hz, 2H, Ar), 7.40-7.51 (m, 3H, Ar), 10.04 (brs, 1H, NH, D_2O exch.); ^{13}C NMR (100 MHz, DMSO-*d*$_6$): δ 11.4, 14.4, 47.11, 61.4, 79.01, 105.0, 128.3, 128.6, 128.7, 133.9, 140.5, 155.7, 160.4, 168.4; HRMS (ESI) calcd. for $C_{16}H_{19}N_4O_4S$ [M + H$^+$]: 363.1122; found: 363.1105.

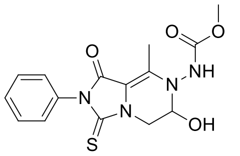

Methyl (6-hydroxy-8-methyl-1-oxo-2-phenyl-3-thioxo-2,3,5,6-tetrahydroimidazo[1,5-*a*]pyrazin-7(1*H*)-yl)carbamate (6j): reaction time: 1.5 h; yield 8%, (27.8 mg); yellow powder from EtOAc/Et_2O; m.p. 173-174 °C; ^{1}H NMR (400 MHz, , DMSO-*d*$_6$): δ 2.26 (s, 3H, CH_3), 3.68 (s, 3H, OCH_3), 3.91 (d, *J* = 12.0 Hz, 1H, NCH_aH_b), 4.24 (d, *J* = 12.8 Hz, 1H, NCH_aH_b), 5.11-5.14 (m, 1H, CH), 7.07 (d, *J* = 5.6 Hz, 1H, OH, D_2O exch.), 7.31 (d, *J* = 7.2 Hz, 2H, Ar), 7.40-7.51 (m, 3H, Ar), 10.08 (brs, 1H, NH, D_2O exch.); ^{13}C NMR (100 MHz, DMSO-*d*$_6$): δ 11.4, 47.4, 52.5, 79.0, 105.0, 128.2, 128.6, 128.7, 134.0, 140.5, 156.1, 160.4, 168.4; IR (Nujol, ν, cm^{-1}): 3719, 3293, 1749, 1699, 1627; HRMS (ESI) calcd. for $C_{15}H_{17}N_4O_4S$ [M + H$^+$]: 349.0965; found: 349.0978.

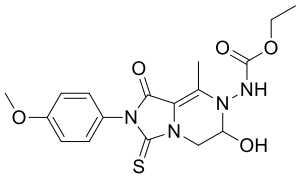

Ethyl (6-hydroxy-2-(4-methoxyphenyl)-8-methyl-1-oxo-3-thioxo-2,3,5,6-tetrahydroimidazo[1,5-*a*]pyrazin-7(1*H*)-yl)carbamate (6k): reaction time: 2.5 h; yield 4.0%, (15.7 mg); orange oil; ^{1}H NMR (400 MHz, , DMSO-*d*$_6$): δ 1.22 (t, *J* = 7.2 Hz, 3H, OCH_2CH_3), 2.26 (s, 3H, CH_3), 3.80 (s, 3H, OCH_3), 3.88 (d, *J* = 12.0 Hz, 1H, NCH_aH_b), 4.13 (q, *J* = 7.2 Hz, 3H, OCH_2CH_3), 4.23 (d, *J* = 12.8 Hz, 1H, NCH_aH_b), 5.10-5.13 (m, 1H, CH), 7.01-7.04 (m, 3H, Ar and OH, D_2O exch.), 7.21 (d, *J* = 8.8 Hz, 2H, Ar), 10.02 (brs, 1H, NH, D_2O exch.); ^{13}C NMR (100 MHz, DMSO-*d*$_6$): δ 11.4, 14.4, 47.5, 55.3, 61.3, 79.0, 105.0, 113.9, 126.6, 129.7, 140.3, 155.6, 158.9, 160.6, 168.8; HRMS (ESI) calcd. for $C_{17}H_{21}N_4O_5S$ [M + H$^+$]: 393.1227; found: 393.1218.

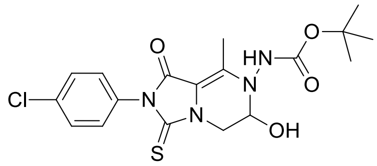

***tert*-Butyl (2-(4-chlorophenyl)-6-hydroxy-8-methyl-1-oxo-3-thioxo-2,3,5,6-tetrahydroimidazo[1,5-*a*]pyrazin-7(1*H*)-yl)carbamate (6l)**: reaction time: 1 h; yield 8%, (34.0 mg); light orange powder from EtOAc/Et_2O; m.p. 138-141 °C dec.; ^{1}H NMR (400 MHz, DMSO-*d*$_6$): δ 1.40 and 1.44 (2 s, 9H, Bu^t), 2.25 (s, 3H, CH_3), 3.86 (d, *J* = 12.4 Hz, 1H, NCH_aH_b), 4.25 (d, *J* = 12.8 Hz, 1H, NCH_aH_b), 5.09 (t, *J* = 2.8 Hz, 1H, CH), 7.00 (brs, 1H, OH, D_2O exch), 7.37 (d, *J* = 8.8 Hz, 2H, Ar), 7.56 (d, *J* = 8.8 Hz, 2H, Ar), 9.51 and 9.80 (2 brs, 1H, NH, D_2O exch.); ^{13}C NMR (100 MHz, DMSO-*d*$_6$): δ 11.6, 27.9, 47.7, 79.5, 80.7, 104.9, 128.7, 130.5, 132.8, 132.9, 141.2, 154.7, 160.1, 167.8; HRMS (ESI) calcd. for $C_{18}H_{22}ClN_4O_4S$ [M + H$^+$]: 425.1045; found: 425.1058.

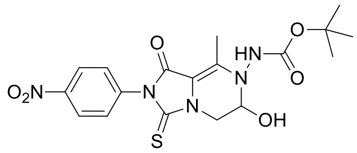

tert-Butyl (6-hydroxy-8-methyl-2-(4-nitrophenyl)-1-oxo-3-thioxo-2,3,5,6-tetrahydroimidazo[1,5-*a*]pyrazin-7(1*H*)-yl)carbamate (6m): reaction time: 3 h; yield 5%, (21.7 mg); orange powder from DCM/Et_2O; m.p. 179-182 °C dec.; ^{1}H NMR (400 MHz, DMSO-*d$_6$*): δ 1.45 (s, 9H, Bu^t), 2.27 (s, 3H, CH_3), 3.88 (d, J = 12.4 Hz, 1H, NCH_aH$_b$), 4.28 (d, J = 12.0 Hz, 1H, NCH$_a$$H_b$), 5.09-5.12 (m, 1H, CH), 7.04 (brs, 1H, OH, D_2O exch.), 7.70 (d, J = 9.2 Hz, 2H, Ar), 8.35 (d, J = 8.8 Hz, 2H, Ar), 9.84 (brs, 1H, NH, D_2O exch.); ^{13}C NMR (100 MHz, DMSO-*d$_6$*): δ 11.5, 27.9, 47.5, 79.2, 80.6, 104.6, 123.8, 129.8, 139.7, 141.7, 146.7, 154.5, 159.7, 167.1; HRMS (ESI) calcd. for $C_{18}H_{22}N_5O_6S$ [M + H$^+$]: 436.1285; found: 436.1273.

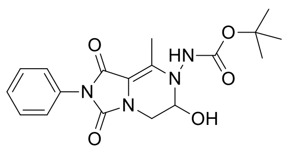

tert-Butyl (6-hydroxy-8-methyl-1,3-dioxo-2-phenyl-3-2,3,5,6-tetrahydroimidazo[1,5-*a*]pyrazin-7(1*H*)-yl)carbamate (6n): reaction time: 3.5 h; yield 21%, (78.6 mg) from **5o**; reaction time: 1.5 h yield 29%, (108.5 mg) from **5q**; light yellow powder from DCM/EtOAc//Et_2O; m.p. 140-141 °C; ^{1}H NMR (400 MHz, DMSO-*d$_6$*): δ 1.44 (s, 9H, Bu^t), 2.21 (s, 3H, CH_3), 3.64 (d, J = 11.2 Hz, 1H, NCH_aH$_b$), 3.80 (d, J = 11.6 Hz, 1H, NCH$_a$$H_b$), 4.94-4.97 (m, 1H, CH), 6.71 (brs, 1H, OH, D_2O exch.) 7.36-7.39 (m, 3H, Ar), 7.47 (t, J = 8.0 Hz, 2H, Ar), 9.55 (brs, 1H, NH, D_2O exch.); ^{13}C NMR (100 MHz, DMSO-*d$_6$*): δ 11.6, 28.0, 44.2, 78.9, 80.1, 103.4, 126.7, 127.4, 128.6, 132.6, 137.0, 150.0, 159.9; HRMS (ESI) calcd. for $C_{18}H_{23}N_4O_5$ [M + H$^+$]: 375.1663; found: 375.1662.

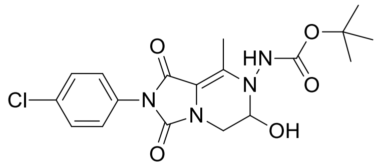

tert-Butyl (2-(4-chlorophenyl)-6-hydroxy-8-methyl-1,3-dioxo-2,3,5,6-tetrahydroimidazo[1,5-*a*]pyrazin-7(1*H*)-yl)carbamate (6o): reaction time: 2.5 h; yield 24%, (98.1 mg); light yellow powder from EtOAc/Et_2O; m.p. 144-145 °C; ^{1}H NMR (400 MHz, DMSO-*d$_6$*): δ 1.43 (s, 9H, Bu^t), 2.21 (s, 3H, CH_3), 3.64 (d, J = 11.6 Hz, 1H, NCH_aH$_b$), 3.79 (d, J = 12.8 Hz, 1H, NCH$_a$$H_b$), 4.94-4.97 (m, 1H, CH), 6.71 (brs, 1H, OH, D_2O exch.), 7.43 (d, J = 8.8 Hz, 2H, Ar), 7.53 (d, J = 8.8 Hz, 2H, Ar), 9.55 (brs, 1H, NH, D_2O exch.); ^{13}C NMR (100 MHz, DMSO-*d$_6$*): δ 11.6, 27.9, 44.2, 78.8, 80.2, 103.2, 128.2, 128.6, 131.5, 131.6, 137.4, 149.6, 154.9, 159.6; HRMS (ESI) calcd. for $C_{18}H_{22}ClN_4O_5$ [M + H$^+$]: 409.1273; found: 409.1257.

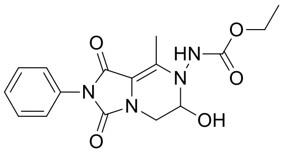

Ethyl (6-hydroxy-8-methyl-1,3-dioxo-2-phenyl-2,3,5,6-tetrahydroimidazo[1,5-*a*]pyrazin-7(1*H*)-yl)carbamate (6p): reaction time: 2 h; yield 35%, (121.2 mg); white powder from EtOAc/Et_2O; m.p. 170-171°C; ^{1}H NMR (400 MHz, DMSO-d_6): δ 1.21 (t, J = 6.8Hz, 3H, OCH_2CH_3), 2.21 (s, 3H, CH_3), 3.67 (d, J = 11.6 Hz, 1H, NCH_aH_b), 3.80 (d, J = 11.2 Hz, 1H, NCH_aH_b), 4.11 (q, J = 7.2 Hz, 2H, OCH_2CH_3), 4.96-4.99 (m, 1H, CH), 6.76 (m, 1H, CH), 7.35-7.39 (m, 3H, Ar), 7.47 (t, J = 7.6 Hz, 2H, Ar) 9.78 (brs, 1H, NH, D_2O exch.); ^{13}C NMR (100 MHz, DMSO-d_6): δ 11.5, 14.4, 41.2, 55.3, 61.0, 78.6, 103.5, 126.7, 127.4, 128.6, 132.6, 136.7, 149.9, 156.0, 159.9; HRMS (ESI) calcd. for $C_{16}H_{19}N_4O_5$ [M + H$^+$]: 347.1350; found: 347.1358.

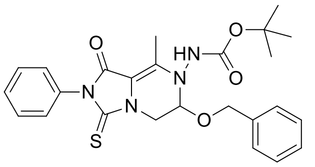

7

***tert*-Butyl (6-(benzyoxy)-8-methyl-1-oxo-2-phenyl-3-thioxo-2,3,5,6-tetrahydroimidazo[1,5-*a*]pyrazin-7(1*H*)-yl)carbamate (7)**: reaction time: 16 h; yield 70%, (50.5 mg); pale orange powder from DCM/Et_2O; m.p. 138-141 °C dec.; ^{1}H NMR (400 MHz, DMSO-d_6): δ 1.45 (s, 9H, Bu^t), 2.28 (s, 3H, CH_3), 3.68 (d, J = 12.4 Hz, 1H, NCH_aH_b), 4.60 (d, J = 13.2 Hz, 1H, NCH_aH_b), 4.67 (d, J = 12.0 Hz, 1H, OCH_aH_b), 4.77 (d, J = 11.6 Hz, 1H, OCH_aH_b), 5.12 (brs, 1H, CH), 7.30-7.50 (m, 10H, Ar), 9.90 and 10.21 (2 brs, 1H, NH, D_2O exch.); ^{13}C NMR (100 MHz, DMSO-d_6): δ 11.4, 27.9, 45.6, 69.5, 80.9, 86.5, 105.9, 127.8, 127.9, 128.3, 128.6, 128.7, 133.8, 137.2, 139.1, 154.9, 160.4, 169.1; HRMS (ESI) calcd. for $C_{25}H_{29}N_4O_4S$ [M + H$^+$]: 481.1904; found: 481.1915.

7 Copies of NMR spectra for compounds 4b–e, 4g–l, 4n–r, 5a–r, 6a–p, and 7

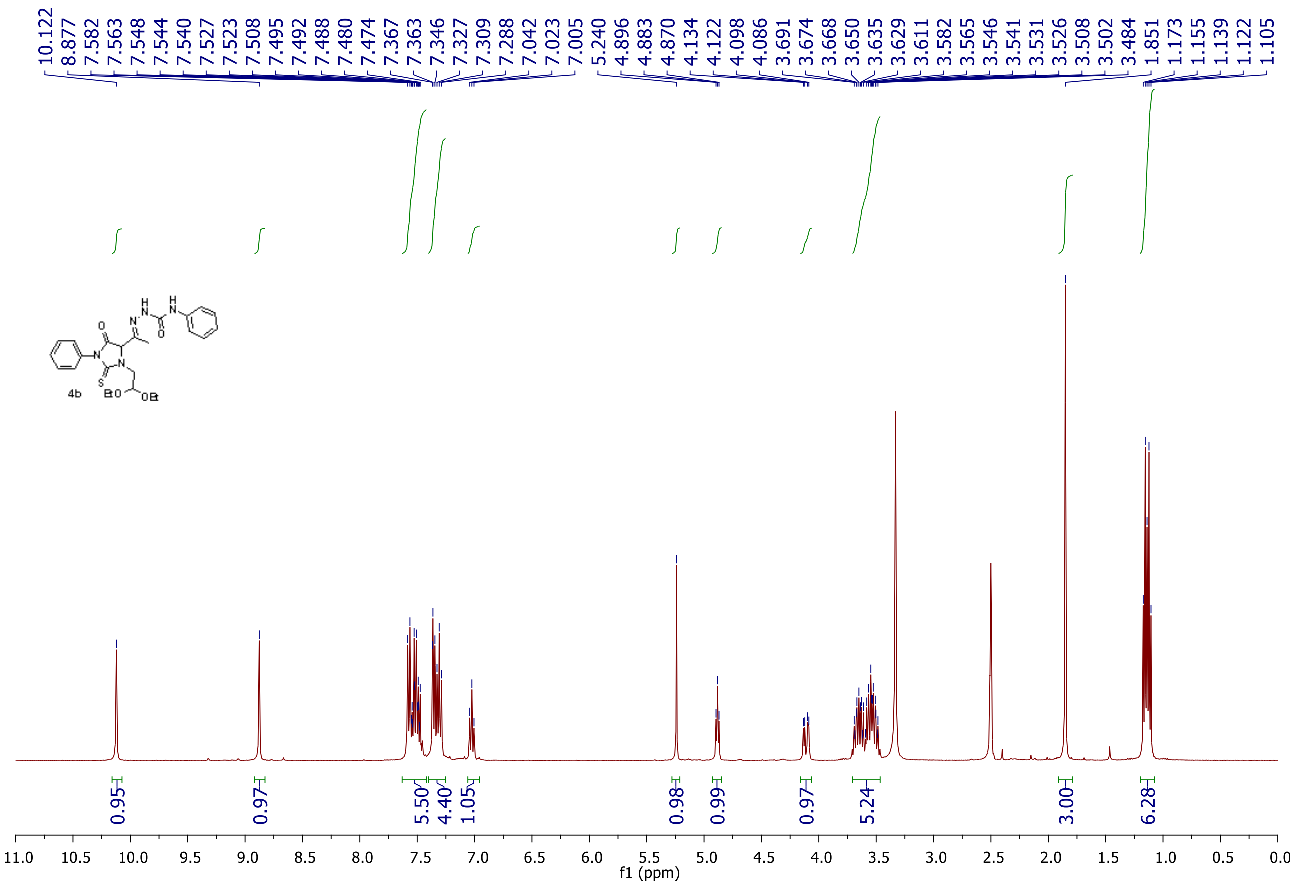

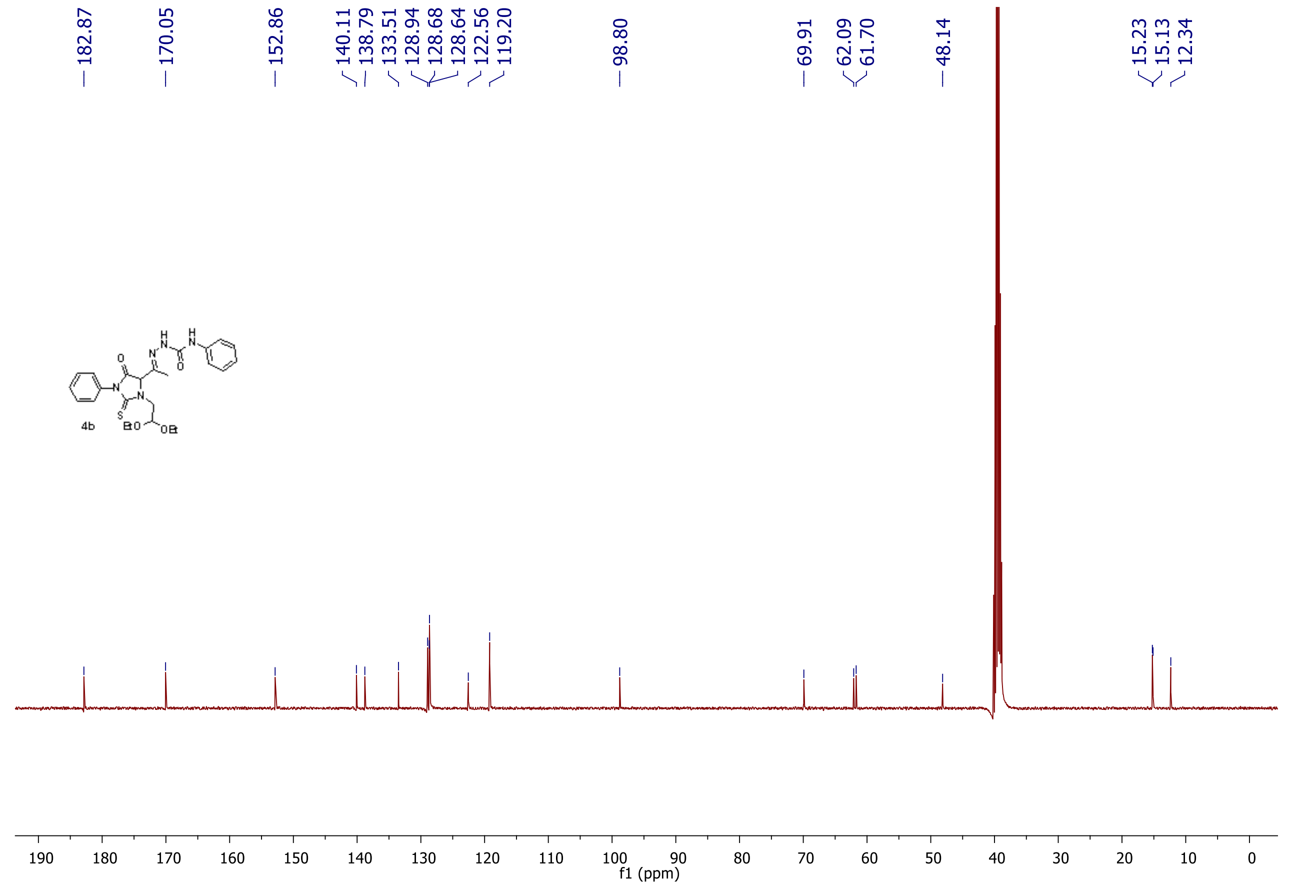

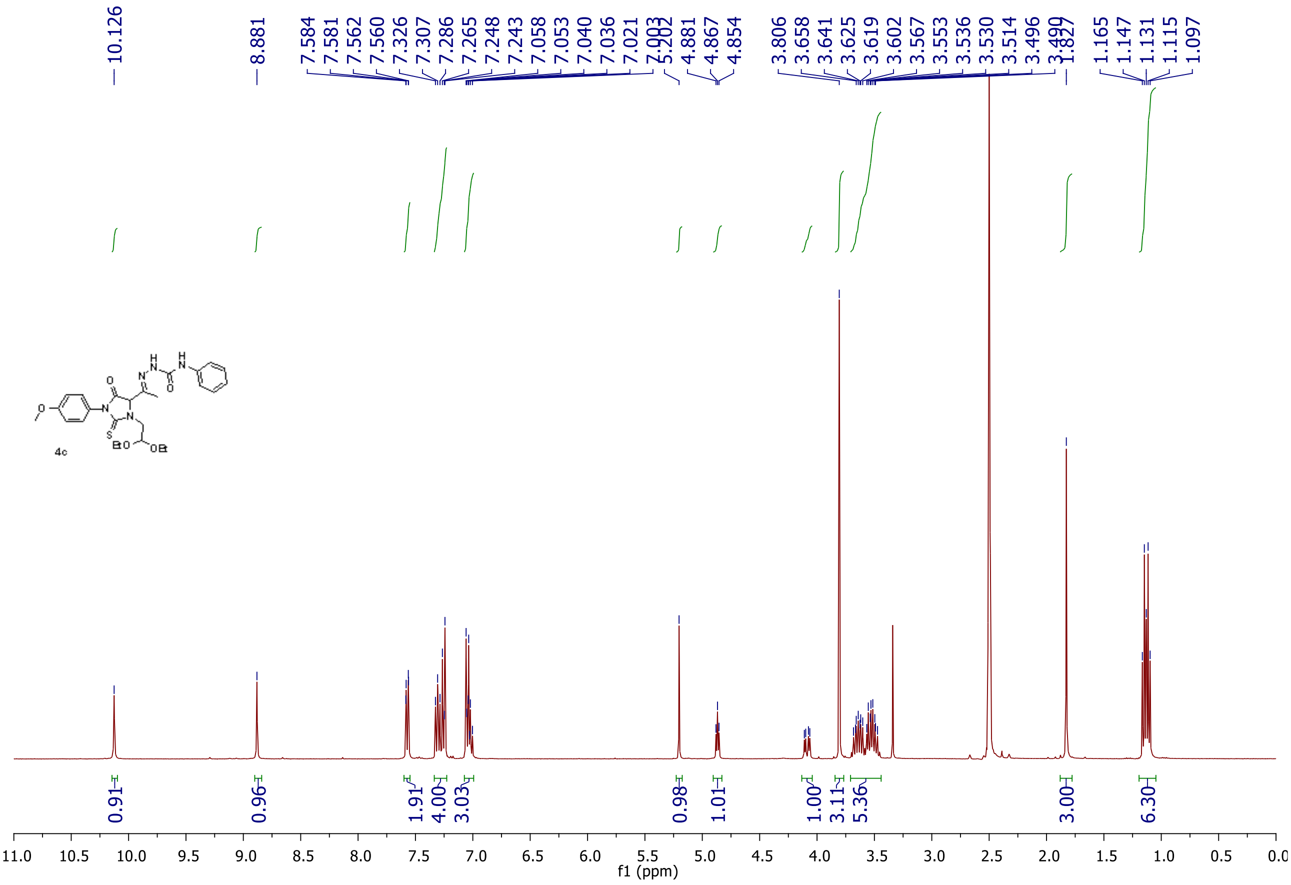
10.126
8.881
7.584
7.581
7.562
7.560
7.326
7.307
7.286
7.265
7.248
7.243
7.058
7.053
7.040
7.036
7.021
5.202
4.881
4.867
4.854
3.806
3.658
3.641
3.625
3.619
3.602
3.567
3.553
3.536
3.530
3.514
3.496
1.827
1.165
1.147
1.131
1.115
1.097
4c
0.91
0.96
1.91
4.00
3.03
0.98
1.01
1.00
3.11
5.36
3.00
6.30
f1 (ppm)

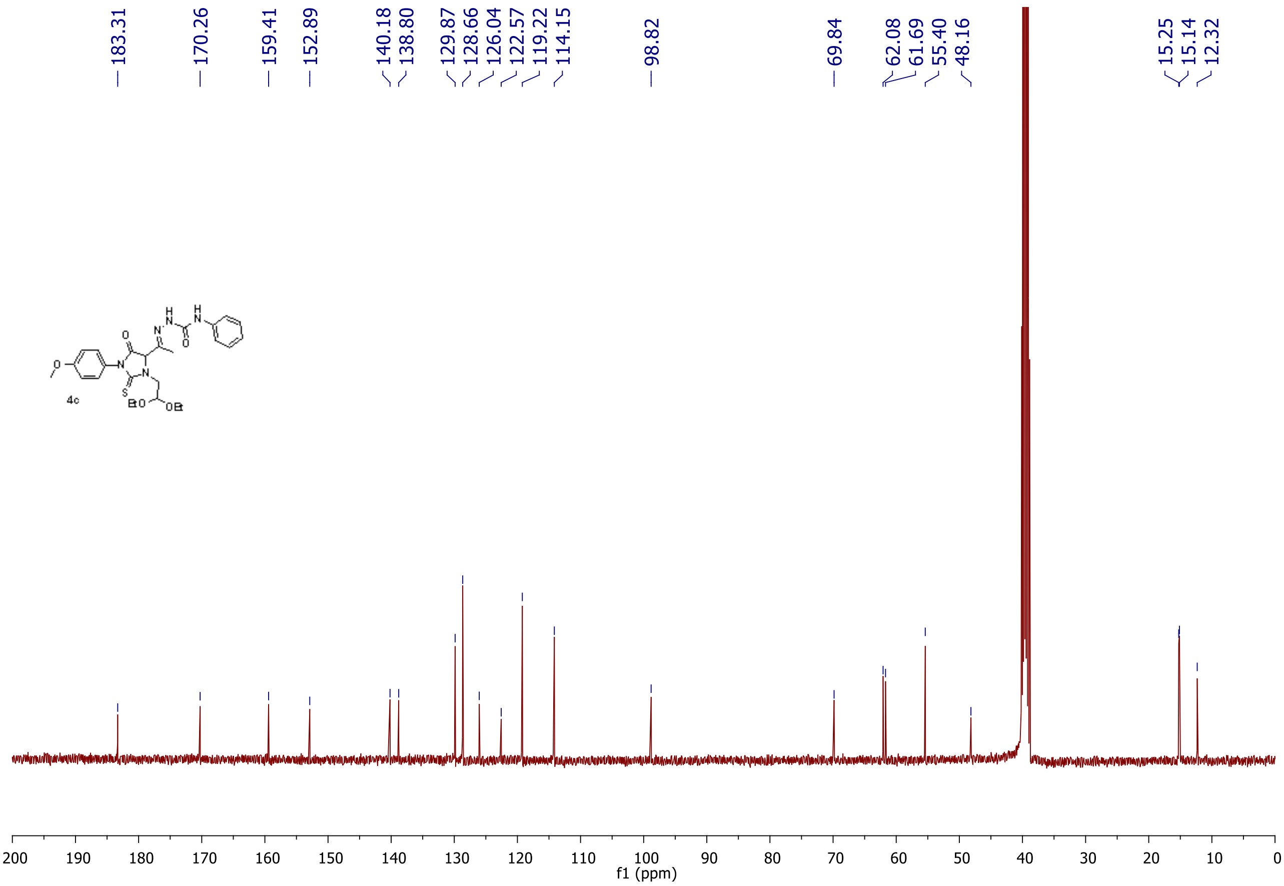
183.31
170.26
159.41
152.89
140.18
138.80
129.87
128.66
126.04
122.57
119.22
114.15
98.82
69.84
62.08
61.69
55.40
48.16
15.25
15.14
12.32
4c
f1 (ppm)

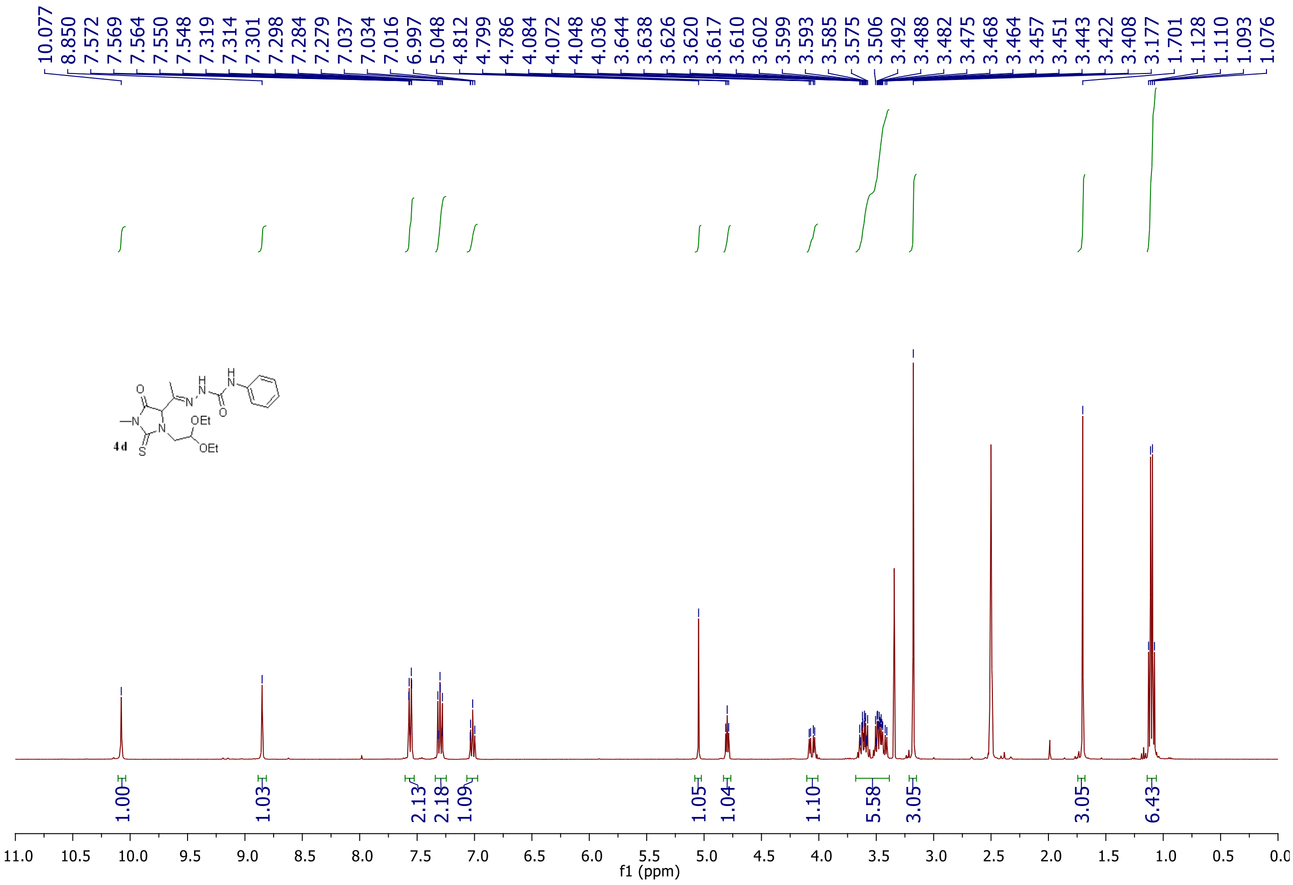

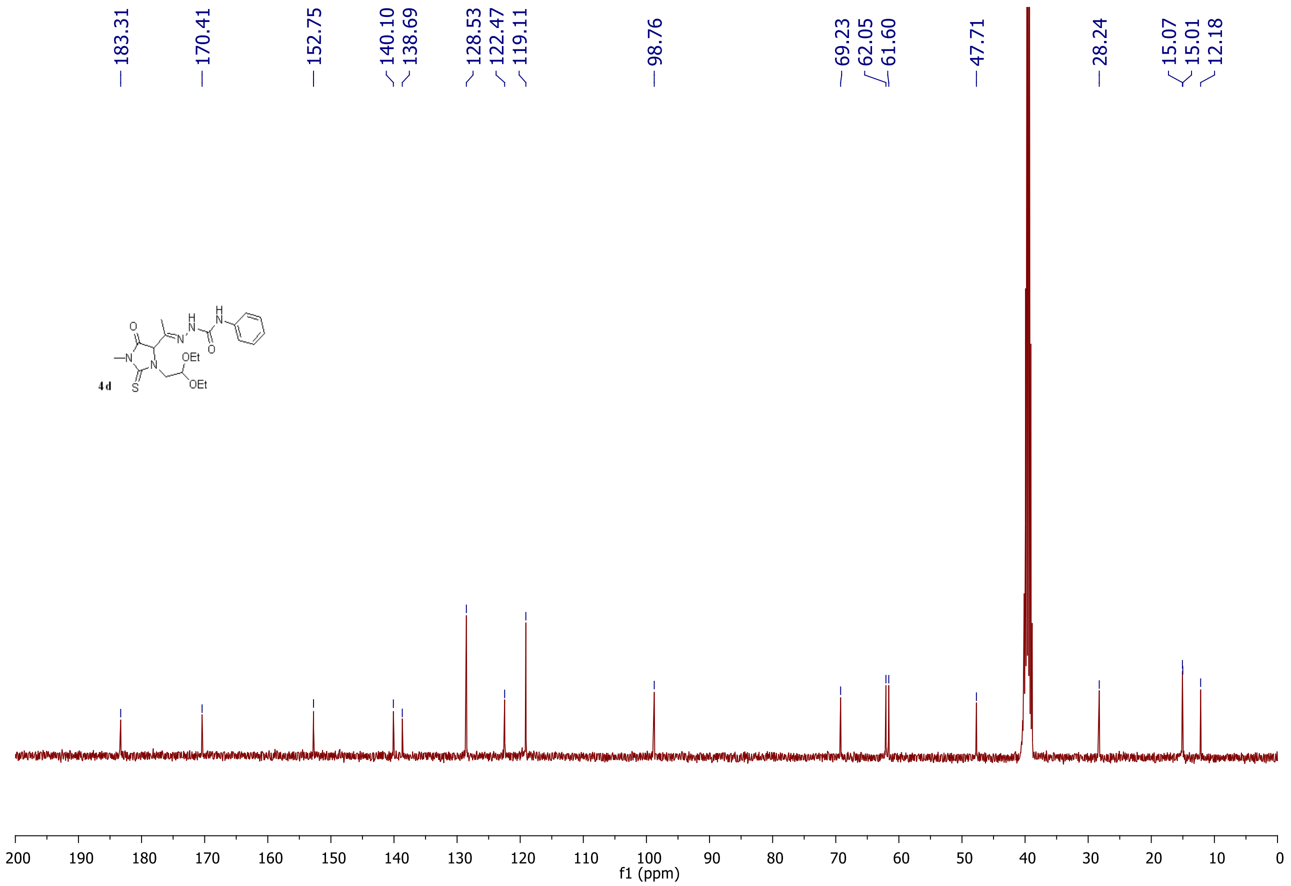

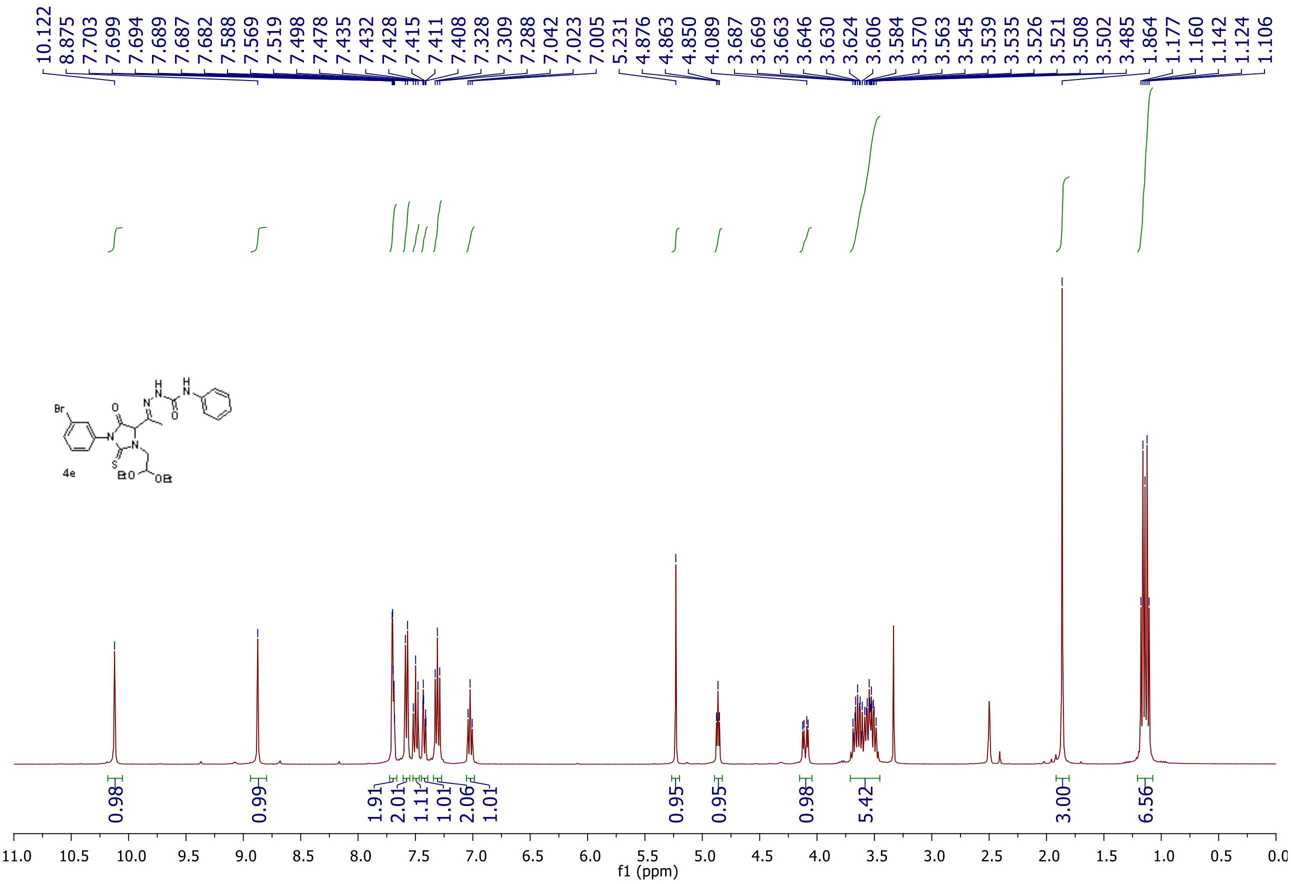
10.122 8.875 7.703 7.699 7.694 7.689 7.687 7.682 7.588 7.569 7.519 7.498 7.478 7.435 7.432 7.428 7.415 7.411 7.408 7.328 7.309 7.288 7.042 7.023 7.005 5.231 4.876 4.863 4.850 4.089 3.687 3.669 3.663 3.646 3.630 3.624 3.606 3.584 3.570 3.563 3.545 3.539 3.535 3.526 3.521 3.508 3.502 3.485 1.864 1.177 1.160 1.142 1.124 1.106
Br
O
N
H
S
4e
EtO
OEt
0.98 0.99 1.91 2.01 1.11 1.01 2.06 1.01 0.95 0.95 0.98 5.42 3.00 6.56
f1 (ppm)

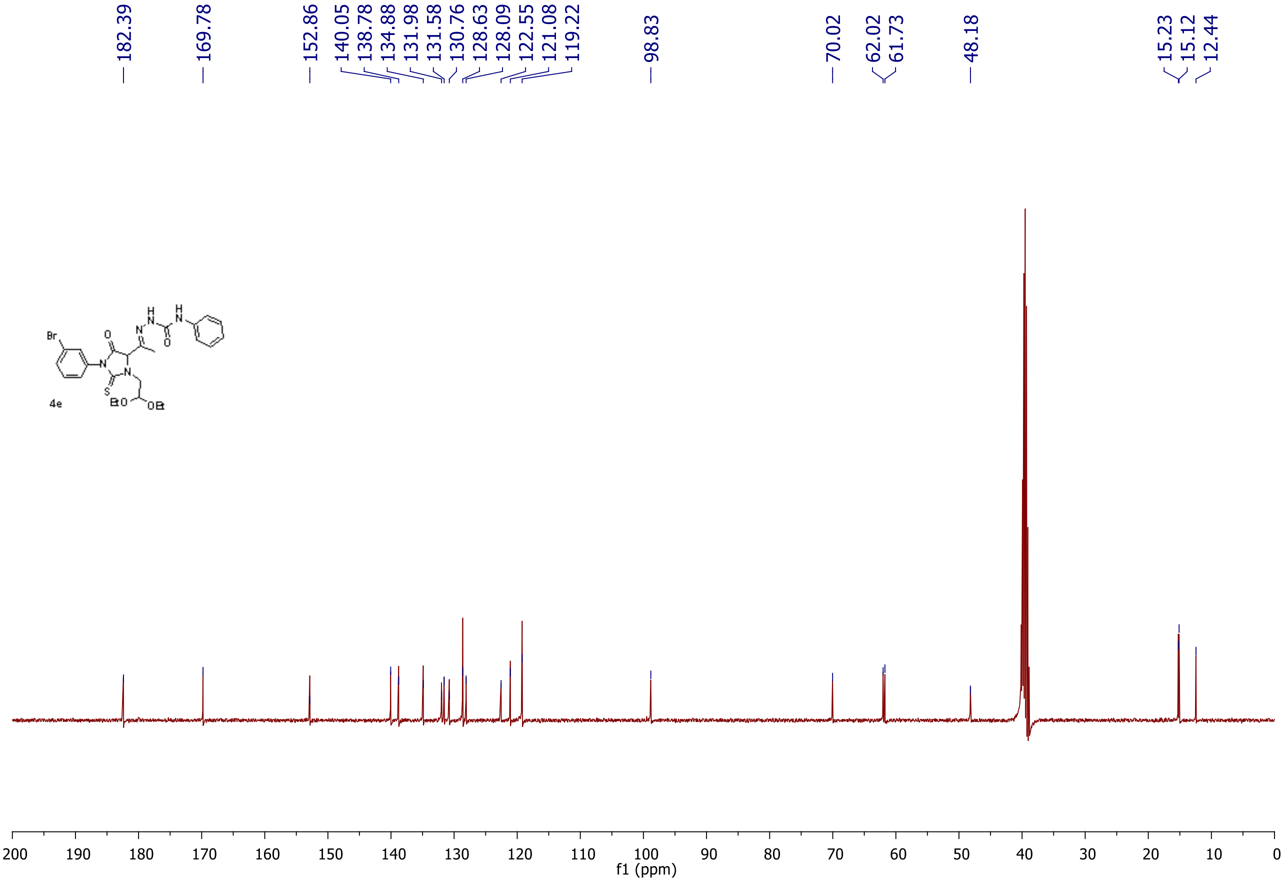
182.39 169.78 152.86 140.05 138.78 134.88 131.98 131.58 130.76 128.63 128.09 122.55 121.08 119.22 98.83 70.02 62.02 61.73 48.18 15.23 15.12 12.44
Br
O
N
H
S
4e
EtO
OEt
f1 (ppm)

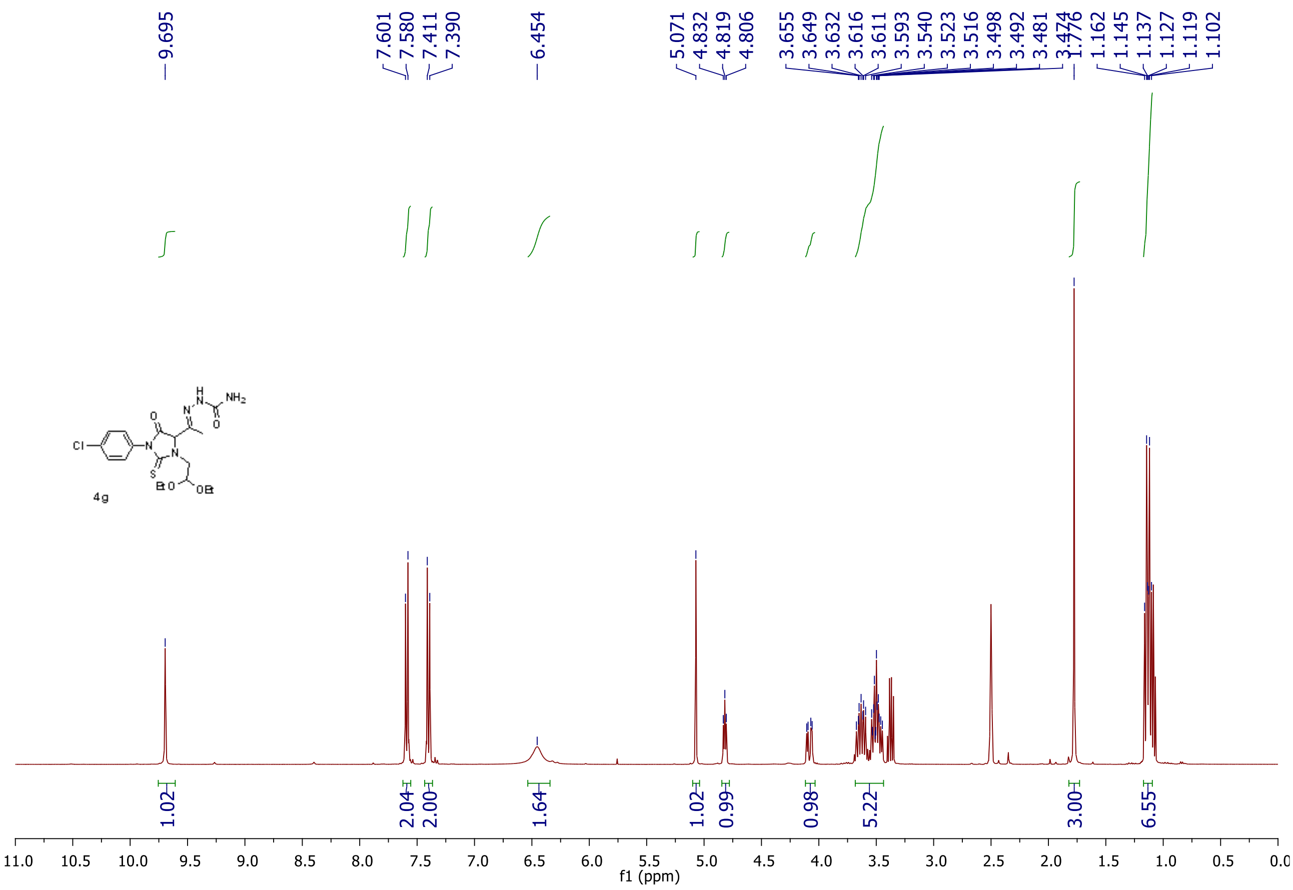

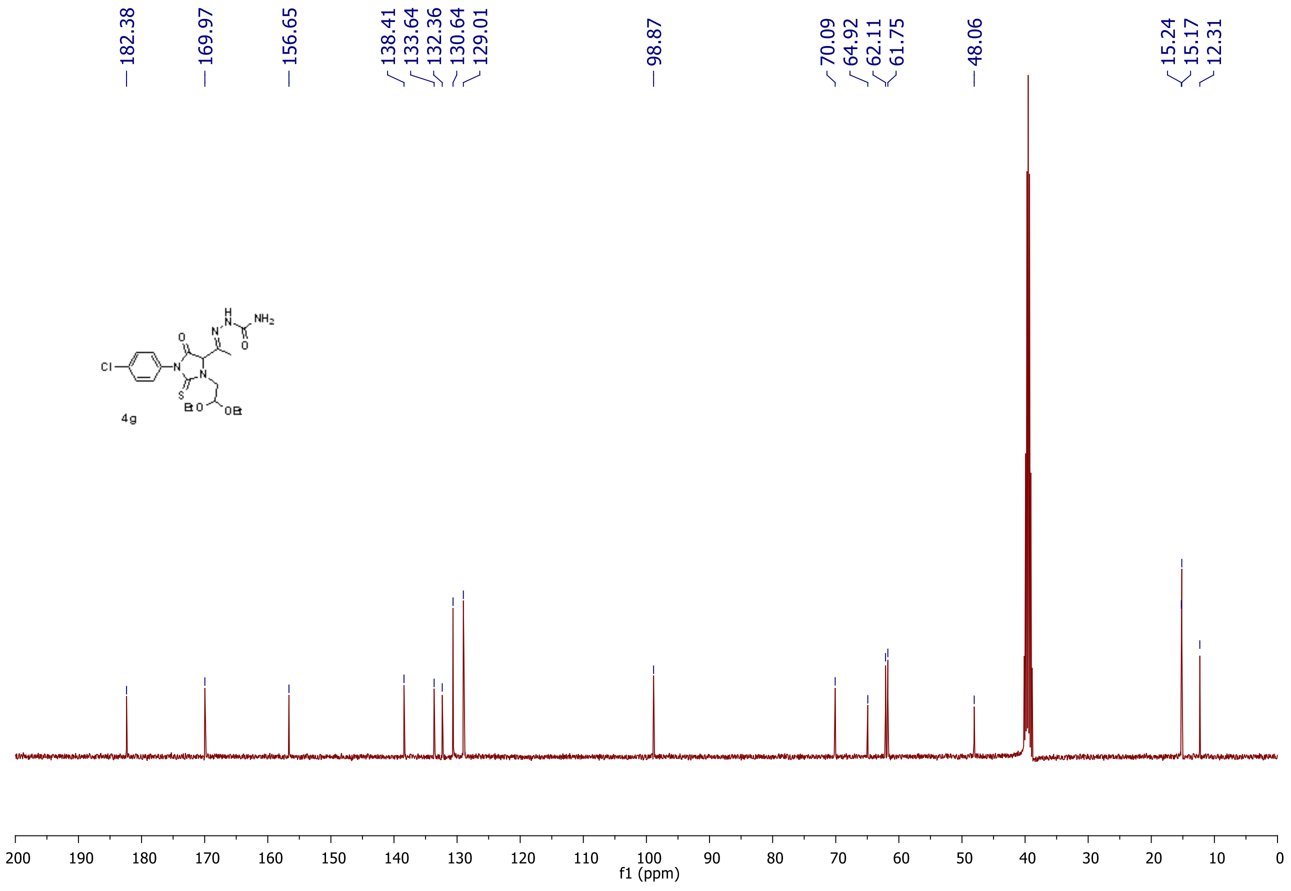

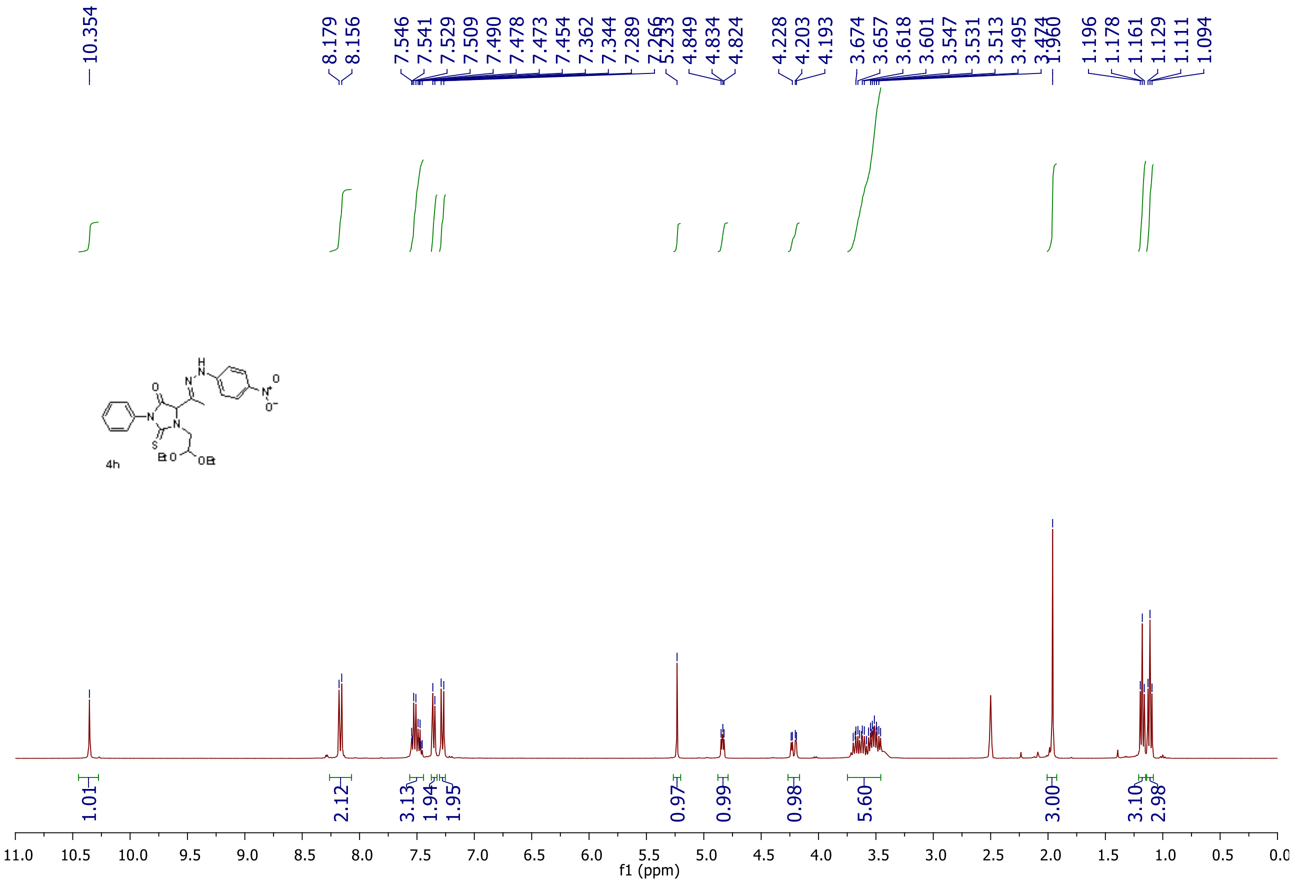
10.354
8.179
8.156
7.546
7.541
7.529
7.509
7.490
7.478
7.473
7.454
7.362
7.344
7.289
7.266
5.233
4.849
4.834
4.824
4.228
4.203
4.193
3.674
3.657
3.618
3.601
3.547
3.531
3.513
3.495
3.474
1.960
1.196
1.178
1.161
1.129
1.111
1.094
4h
1.01
2.12
3.13
1.94
1.95
0.97
0.99
0.98
5.60
3.00
3.10
2.98
f1 (ppm)

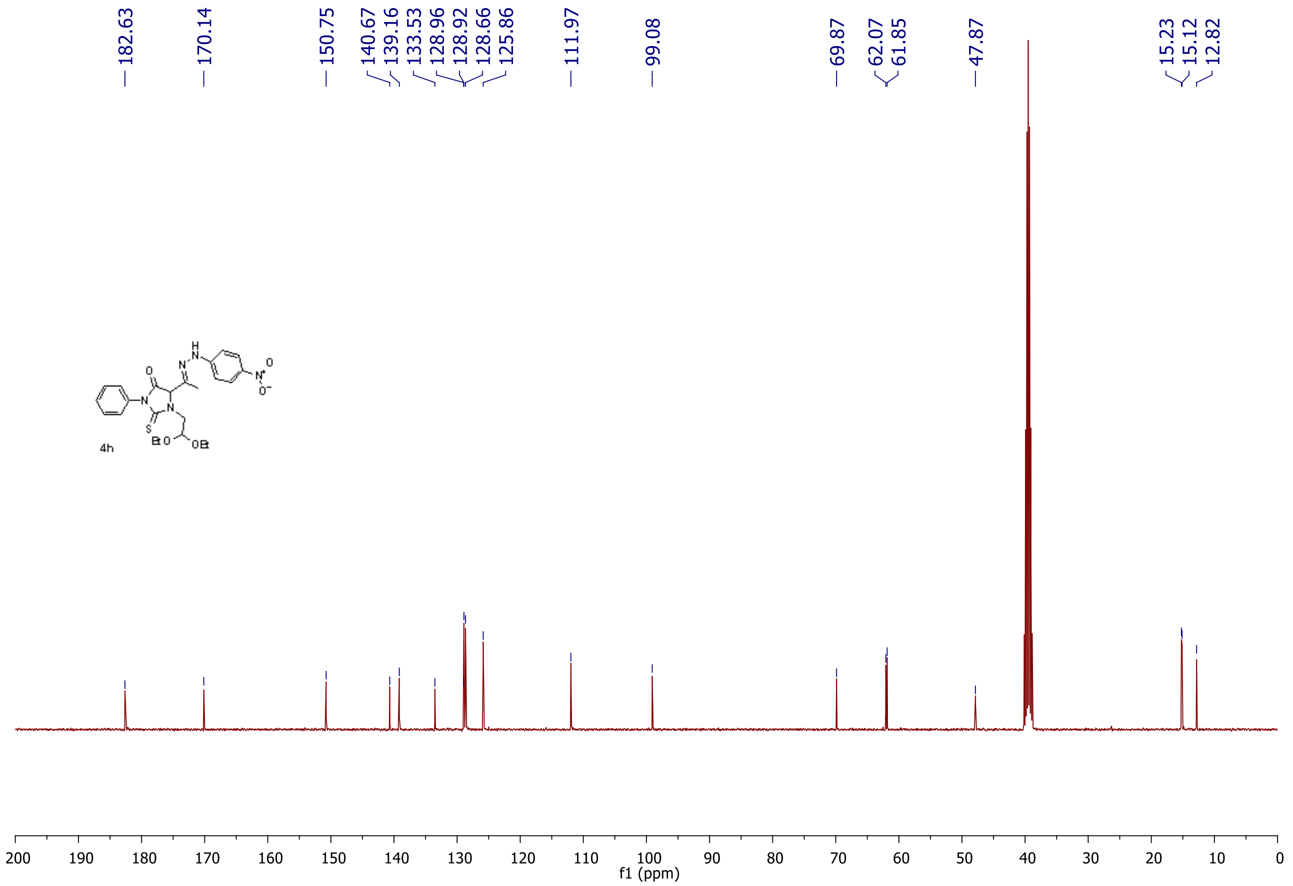
182.63
170.14
150.75
140.67
139.16
133.53
128.96
128.92
128.66
125.86
111.97
99.08
69.87
62.07
61.85
47.87
15.23
15.12
12.82
4h
f1 (ppm)

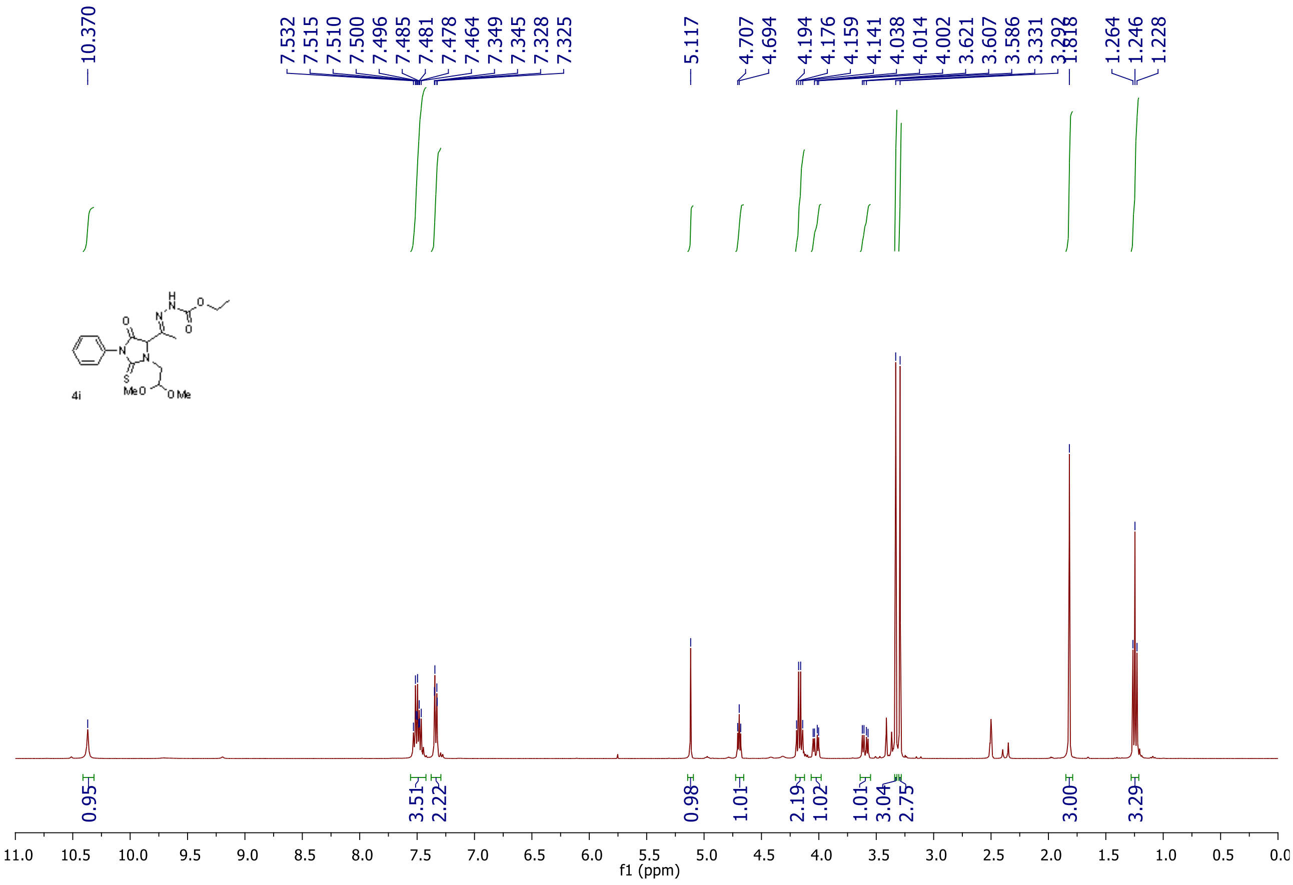
10.370
7.532
7.515
7.510
7.500
7.496
7.485
7.481
7.478
7.464
7.349
7.345
7.328
7.325
5.117
4.707
4.694
4.194
4.176
4.159
4.141
4.038
4.014
4.002
3.621
3.607
3.586
3.331
3.292
1.818
1.264
1.246
1.228
4i
0.95
3.51
2.22
0.98
1.01
2.19
1.02
1.01
3.04
2.75
3.00
3.29
f1 (ppm)

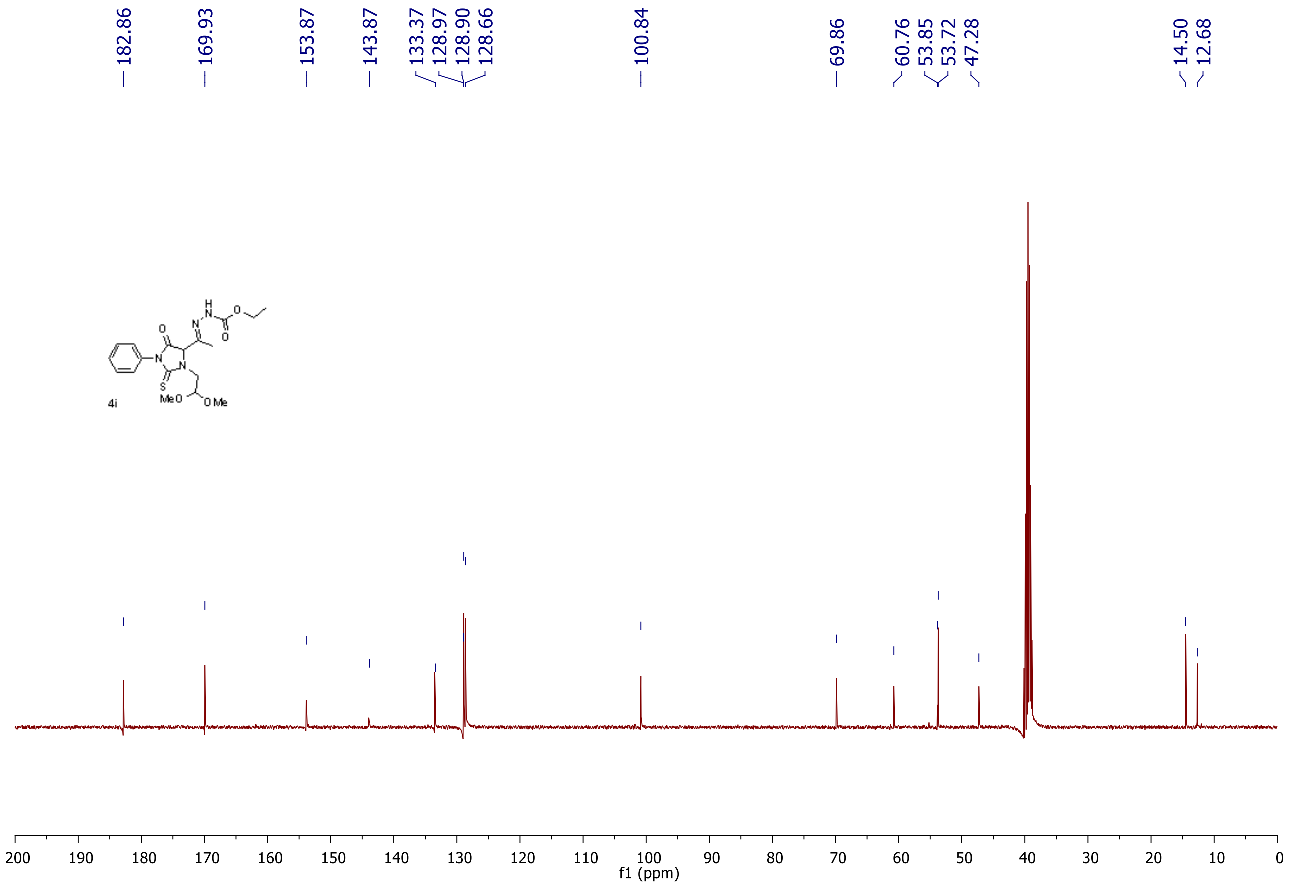
182.86
169.93
153.87
143.87
133.37
128.97
128.90
128.66
100.84
69.86
60.76
53.85
53.72
47.28
14.50
12.68
4i
f1 (ppm)

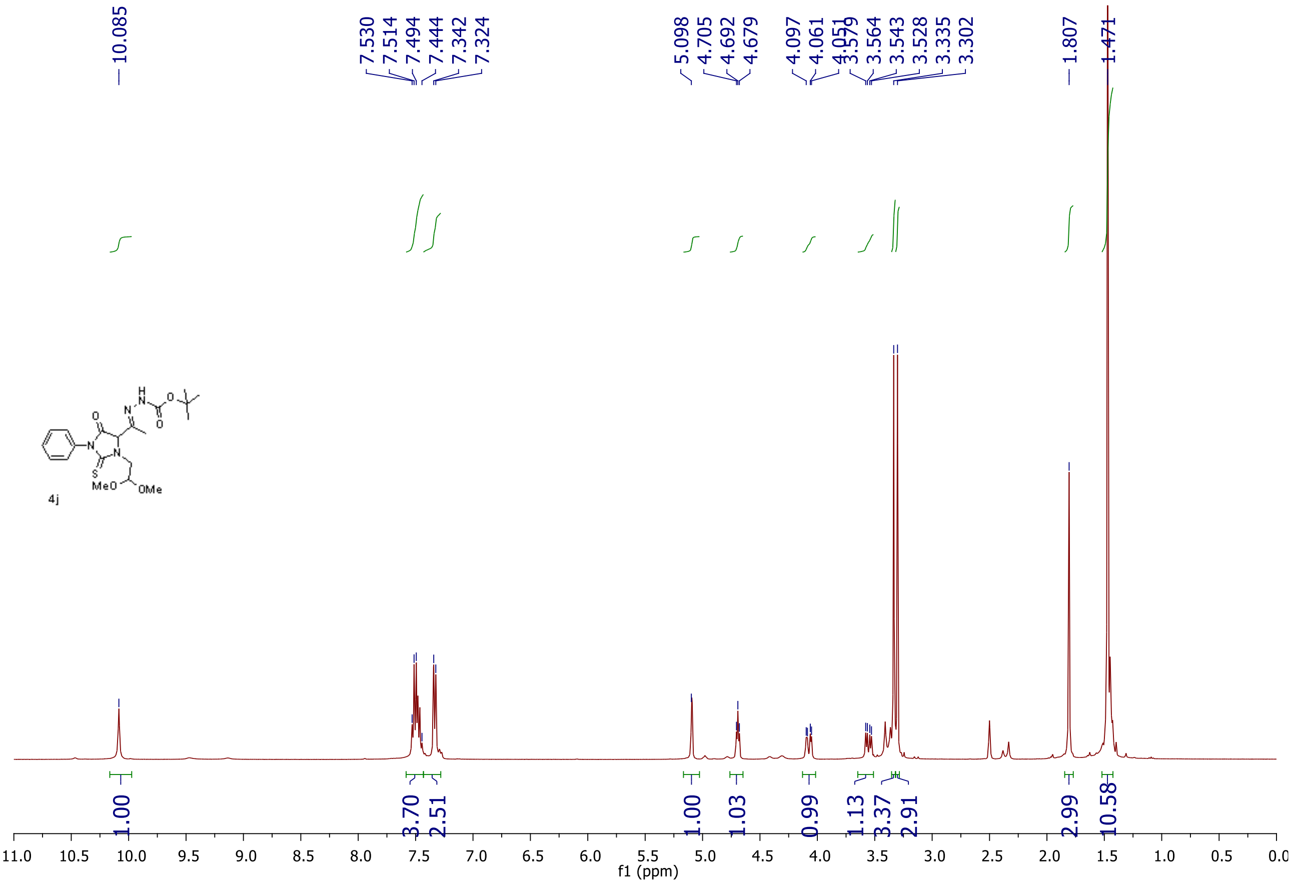
10.085
7.530 7.514 7.494 7.444 7.342 7.324
5.098 4.705 4.692 4.679
4.097 4.061 4.051 3.579 3.564 3.543 3.528 3.335 3.302
1.807 1.471
4j
1.00 3.70 2.51 1.00 1.03 0.99 1.13 3.37 2.91 2.99 10.58
f1 (ppm)

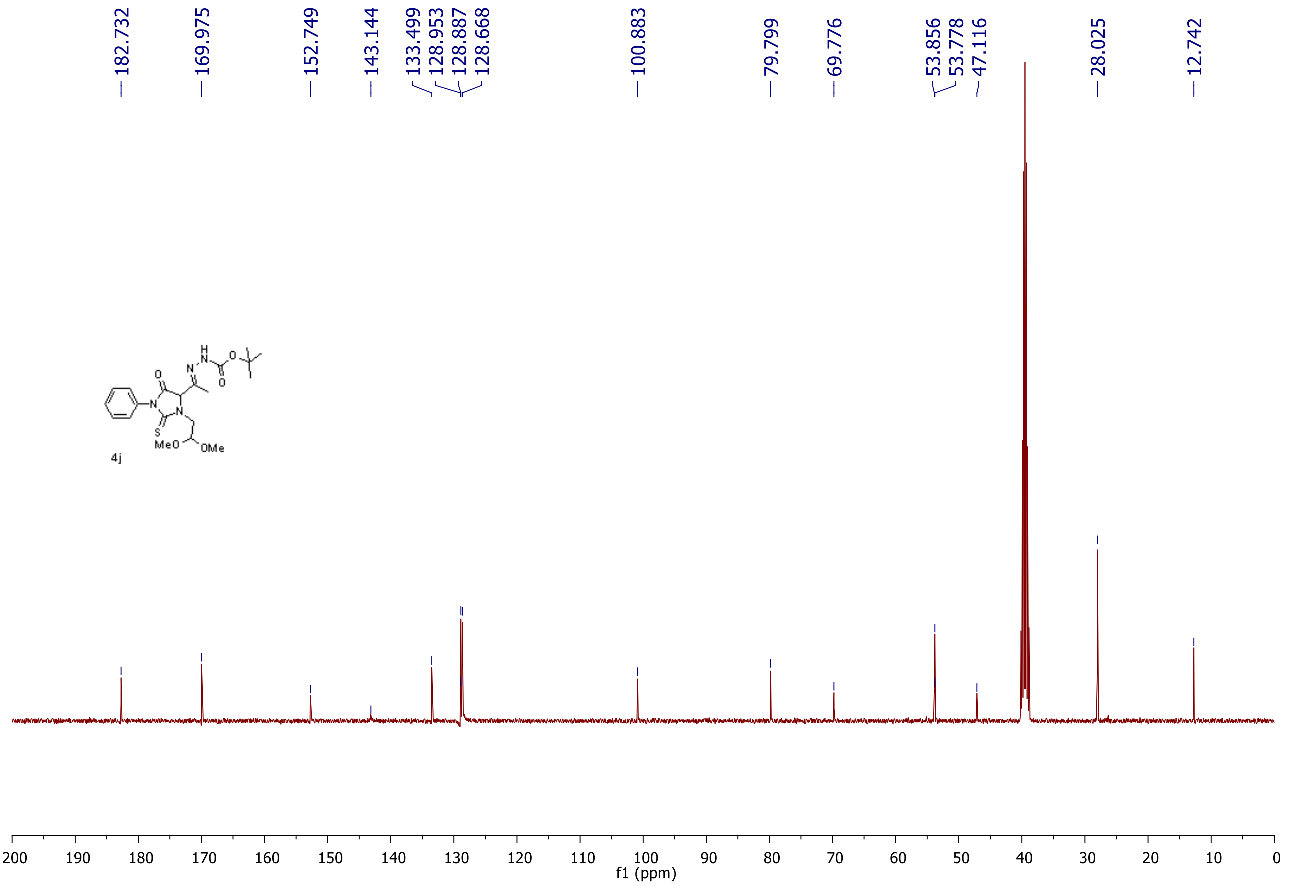
182.732
169.975
152.749
143.144
133.499 128.953 128.887 128.668
100.883
79.799
69.776
53.856 53.778 47.116
28.025
12.742
4j
f1 (ppm)

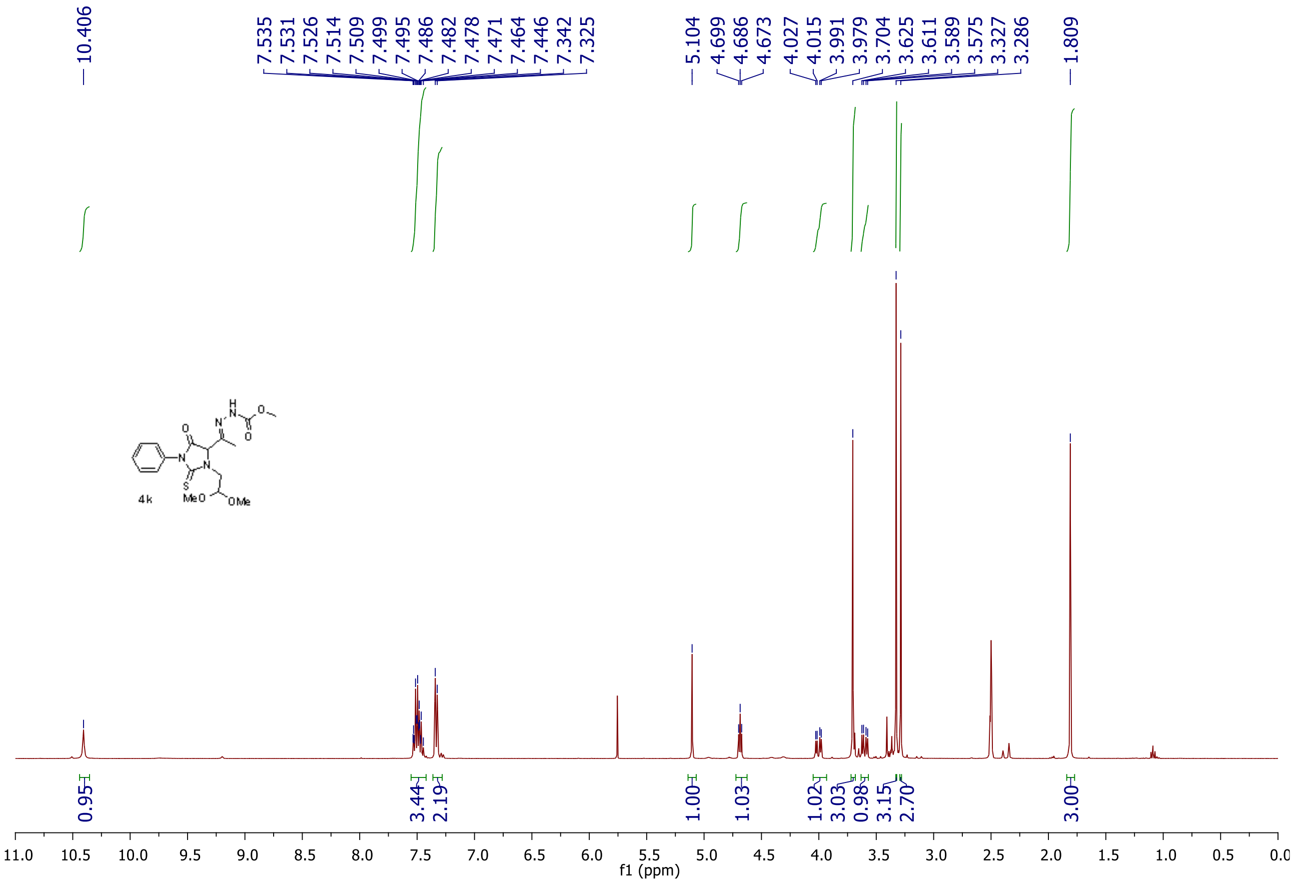

10.406
7.535 7.531 7.526 7.514 7.509 7.499 7.495 7.486 7.482 7.478 7.471 7.464 7.446 7.342 7.325
5.104 4.699 4.686 4.673 4.027 4.015 3.991 3.979 3.704 3.625 3.611 3.589 3.575 3.327 3.286
1.809
4k
0.95 3.44 2.19 1.00 1.03 1.02 3.03 0.98 3.15 2.70 3.00
f1 (ppm)

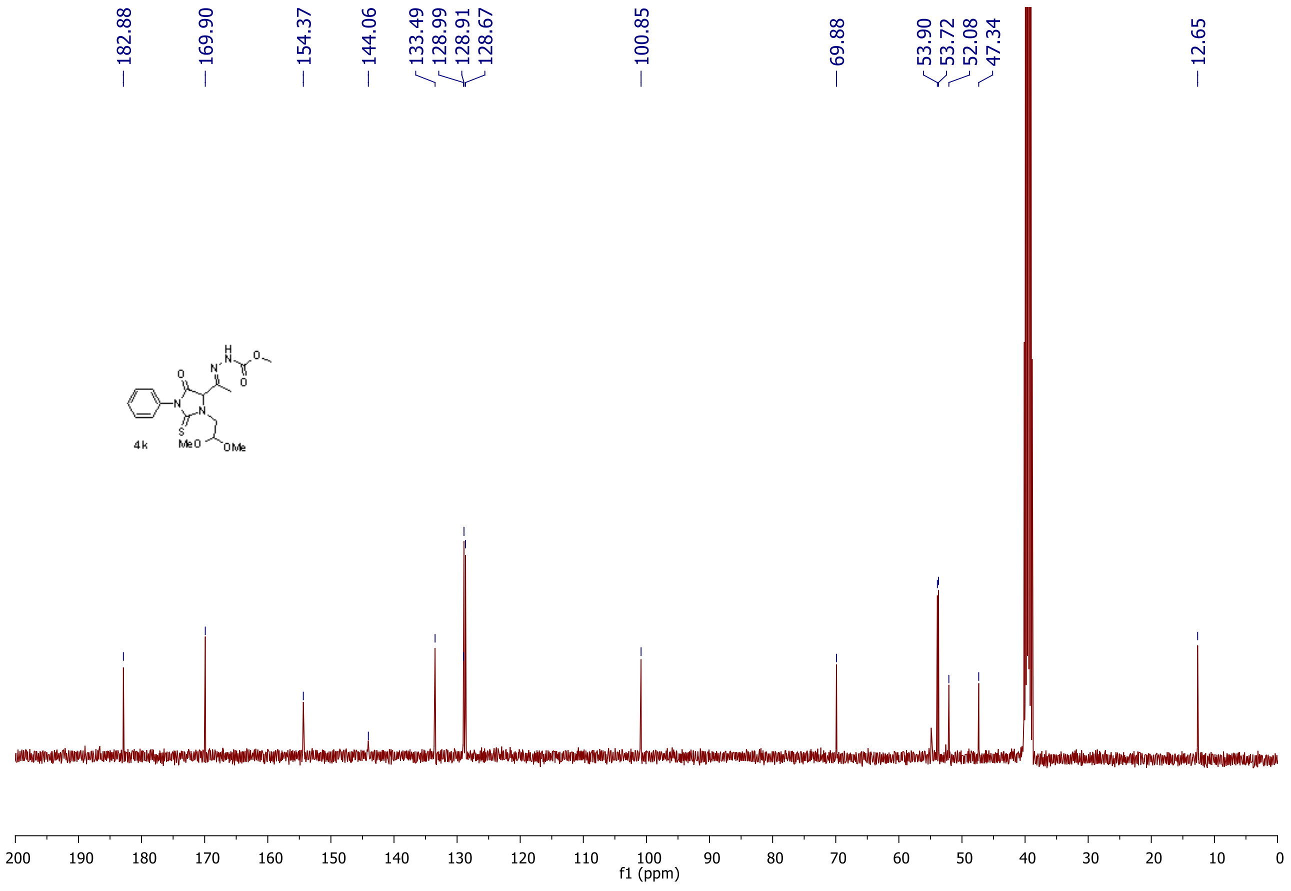

182.88 169.90 154.37 144.06 133.49 128.99 128.91 128.67 100.85 69.88 53.90 53.72 52.08 47.34 12.65
4k
f1 (ppm)

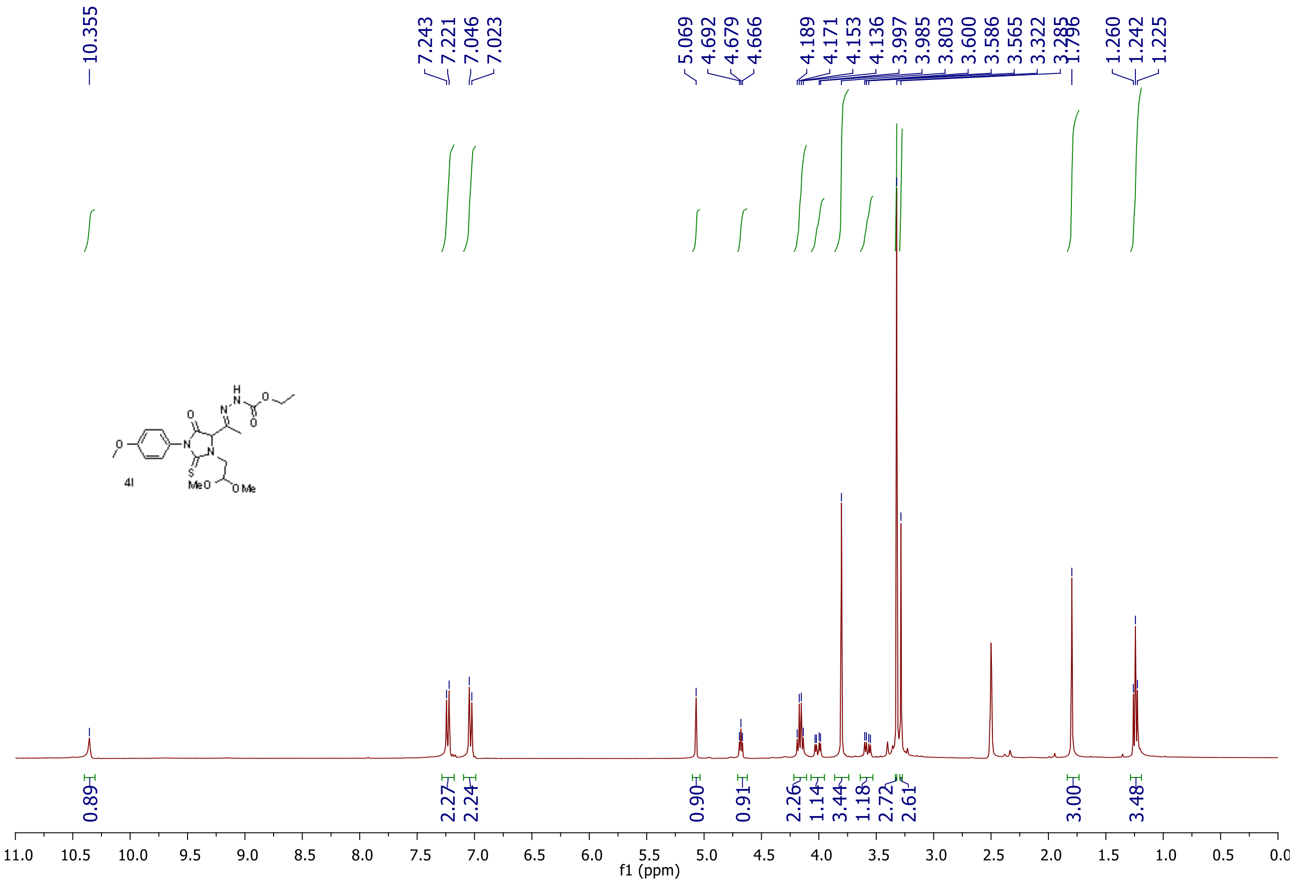
10.355
7.243
7.221
7.046
7.023
5.069
4.692
4.679
4.666
4.189
4.171
4.153
4.136
3.997
3.985
3.803
3.600
3.586
3.565
3.322
3.285
1.796
1.260
1.242
1.225
4l
0.89
2.27
2.24
0.90
0.91
2.26
1.14
3.44
1.18
2.72
2.61
3.00
3.48
f1 (ppm)

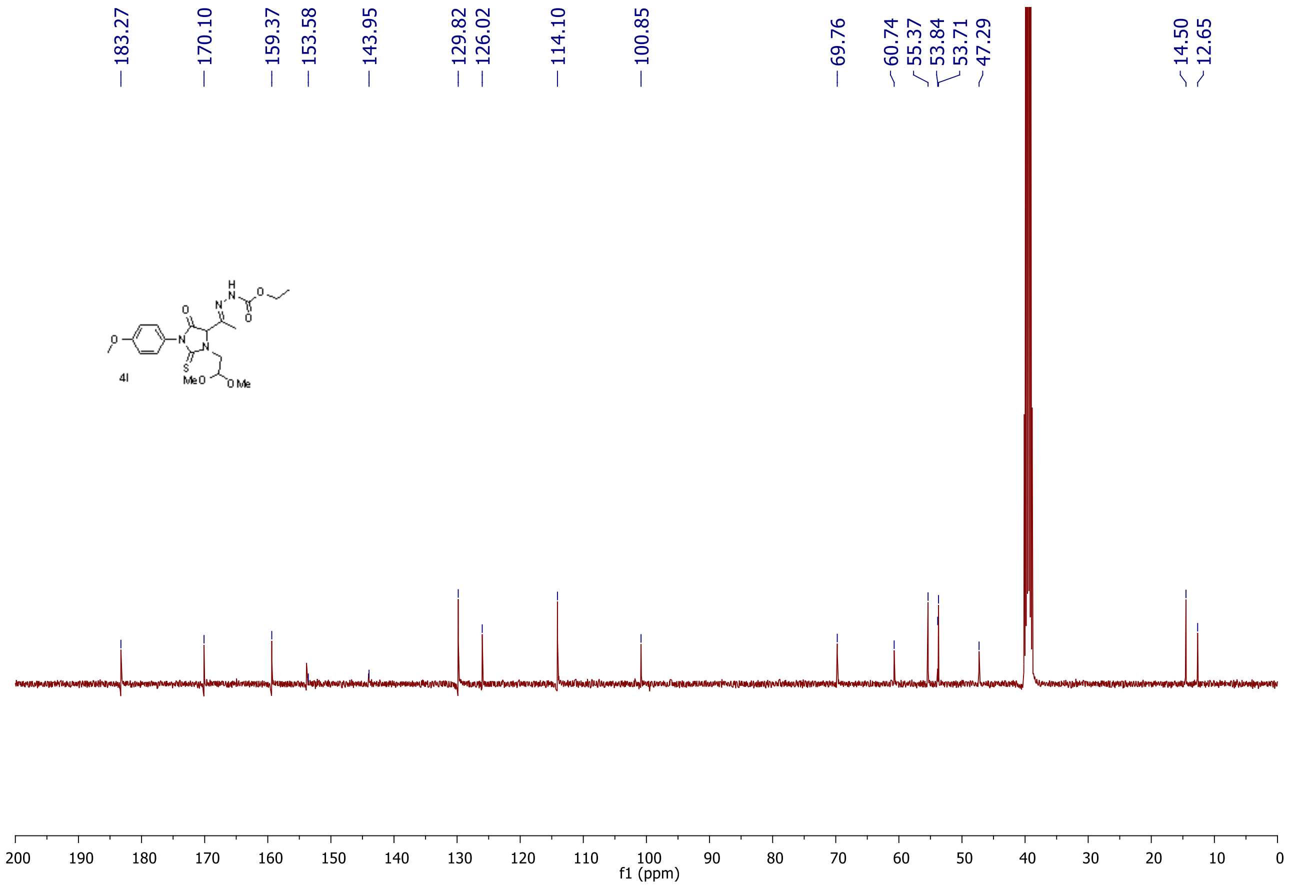
183.27
170.10
159.37
153.58
143.95
129.82
126.02
114.10
100.85
69.76
60.74
55.37
53.84
53.71
47.29
14.50
12.65
4l
f1 (ppm)

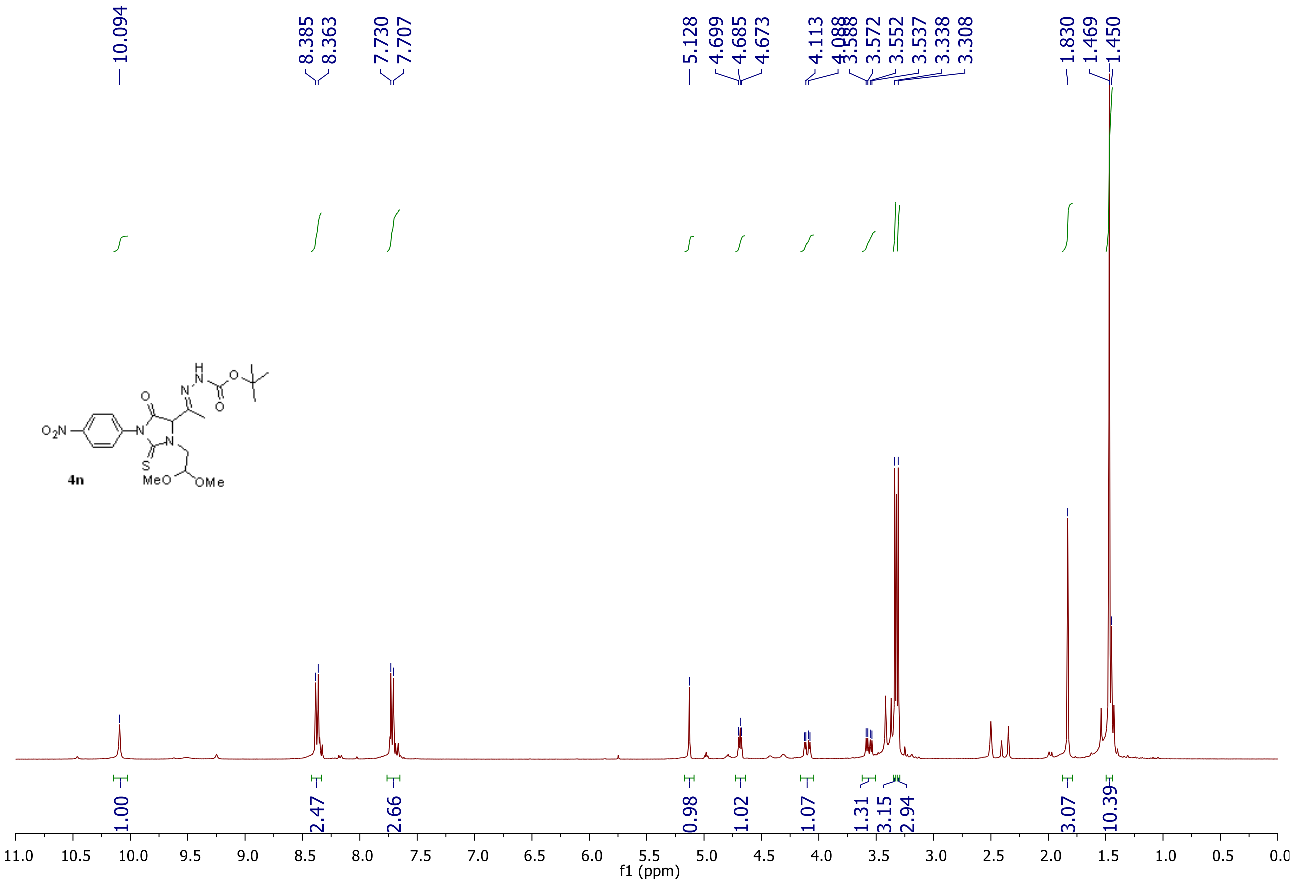
10.094
8.385
8.363
7.730
7.707
5.128
4.699
4.685
4.673
4.113
4.088
3.588
3.572
3.552
3.537
3.338
3.308
1.830
1.469
1.450
4n
1.00
2.47
2.66
0.98
1.02
1.07
1.31
3.15
2.94
3.07
10.39
f1 (ppm)

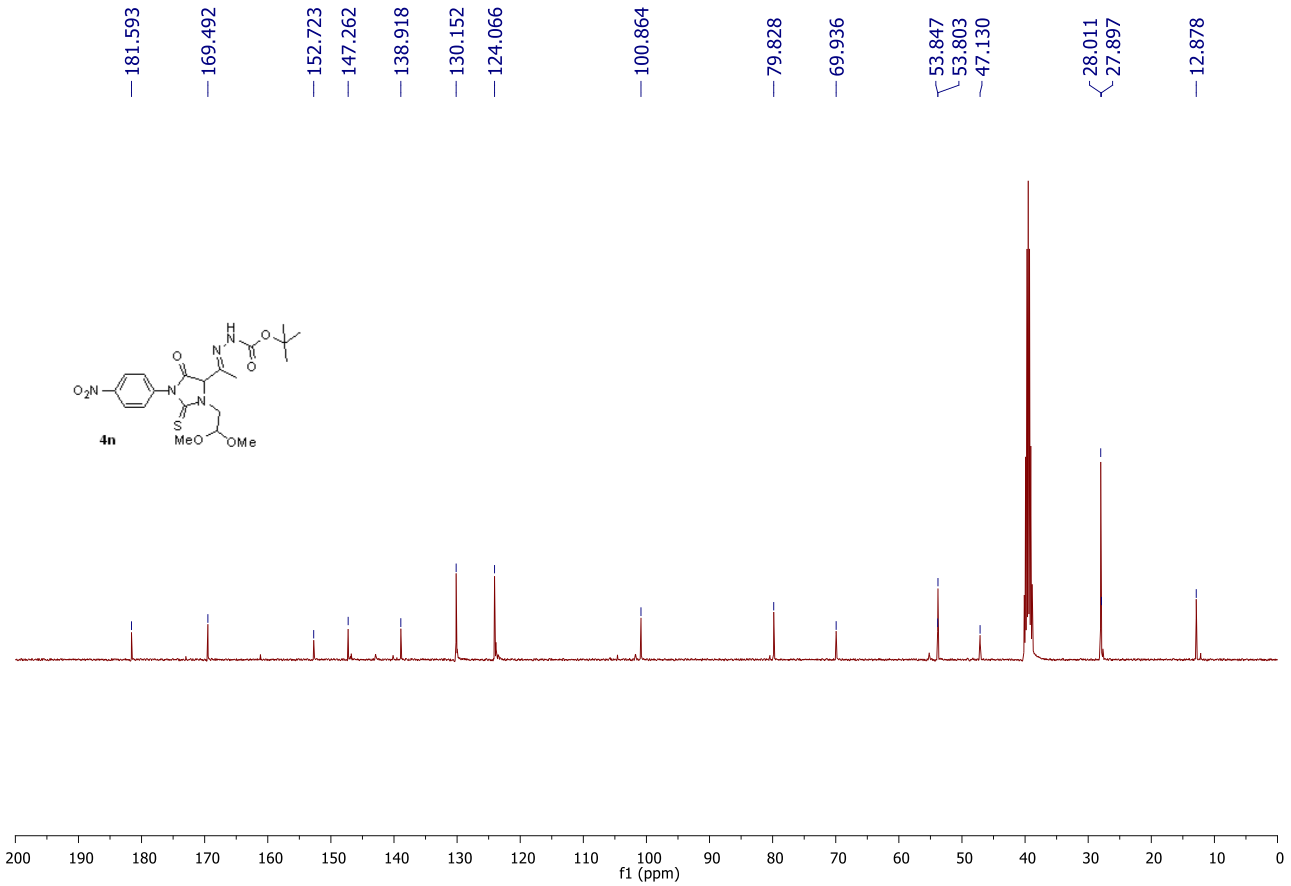
181.593
169.492
152.723
147.262
138.918
130.152
124.066
100.864
79.828
69.936
53.847
53.803
47.130
28.011
27.897
12.878
4n
f1 (ppm)

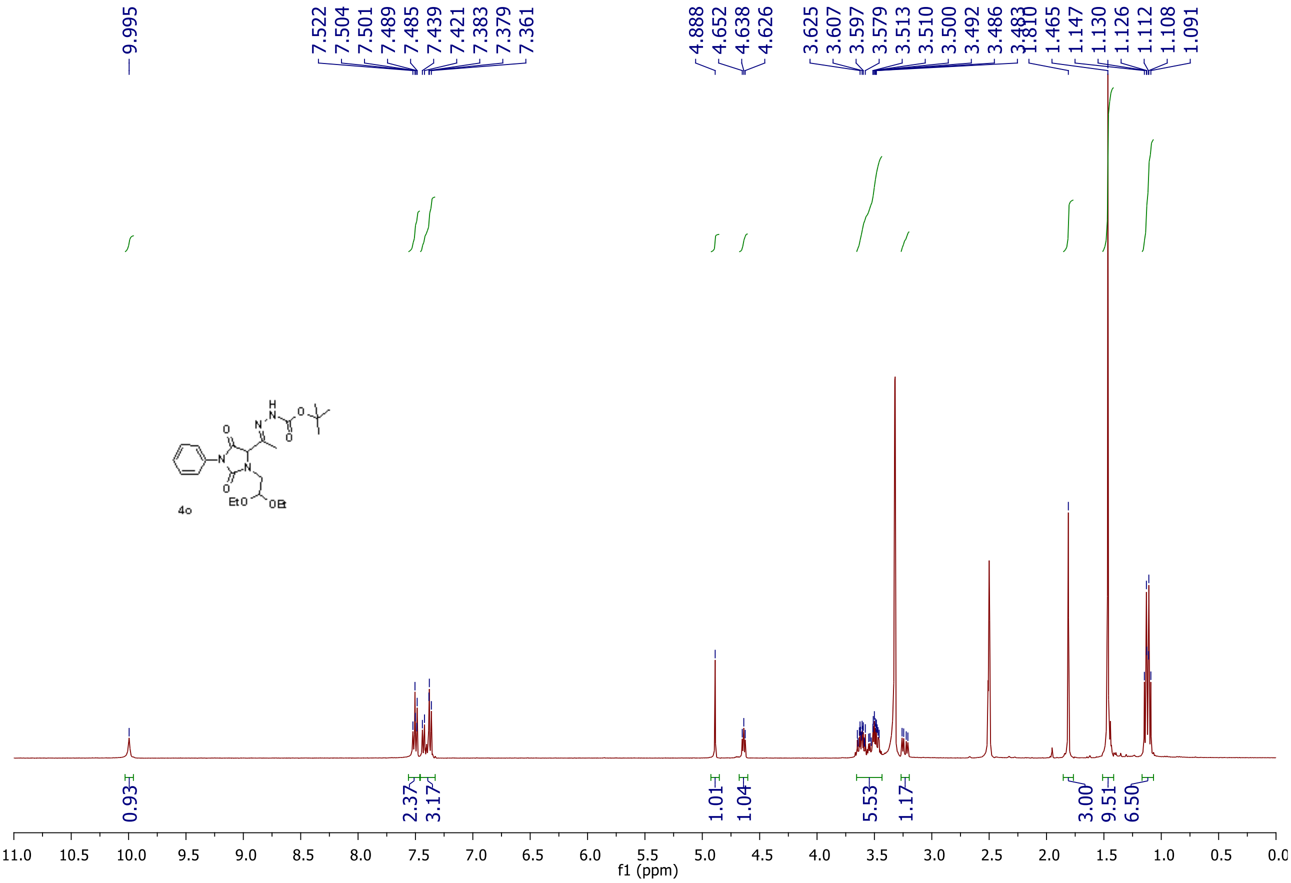
9.995
7.522
7.504
7.501
7.489
7.485
7.439
7.421
7.383
7.379
7.361
4.888
4.652
4.638
4.626
3.625
3.607
3.597
3.579
3.513
3.510
3.500
3.492
3.486
3.483
1.810
1.465
1.147
1.130
1.126
1.112
1.108
1.091
4o
0.93
2.37
3.17
1.01
1.04
5.53
1.17
3.00
9.51
6.50
f1 (ppm)

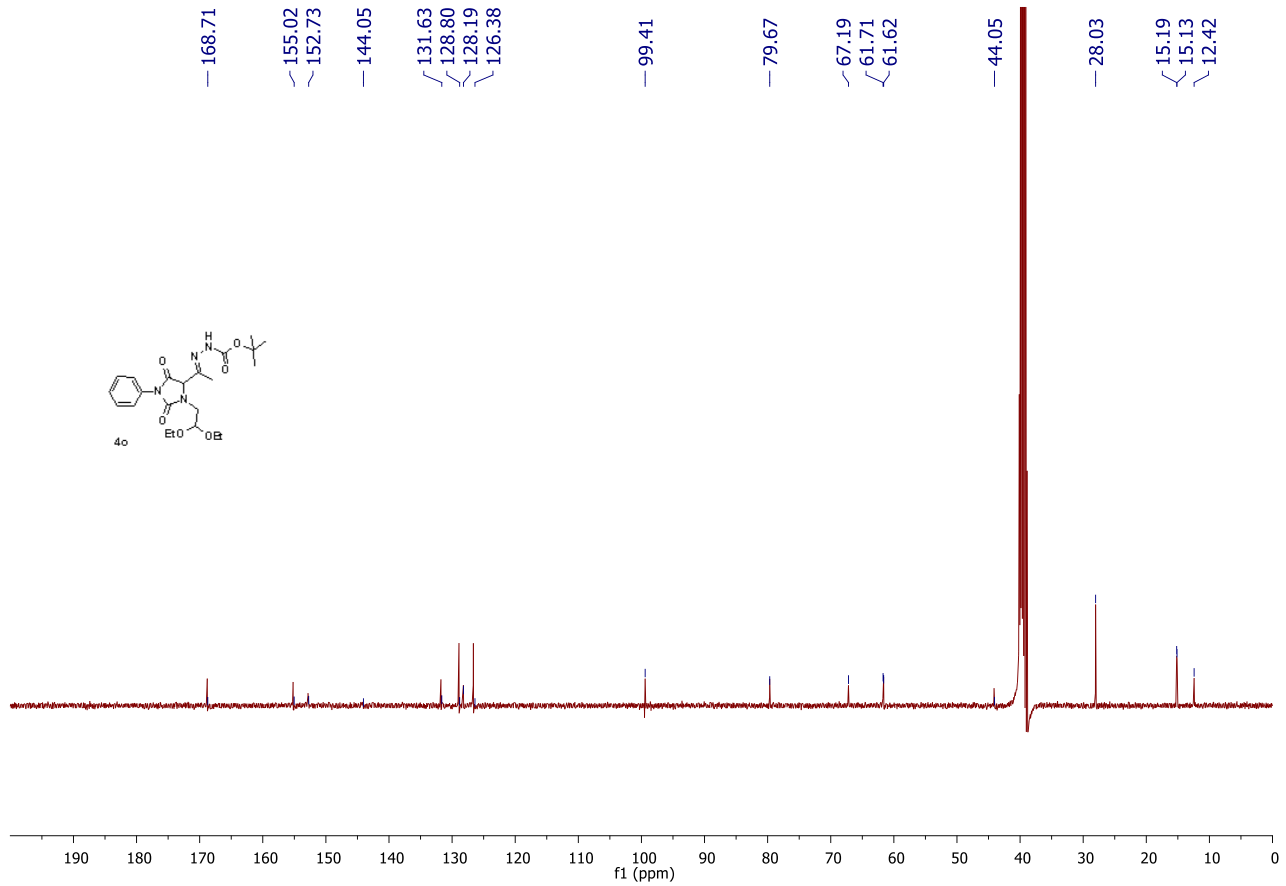
168.71
155.02
152.73
144.05
131.63
128.80
128.19
126.38
99.41
79.67
67.19
61.71
61.62
44.05
28.03
15.19
15.13
12.42
4o
f1 (ppm)

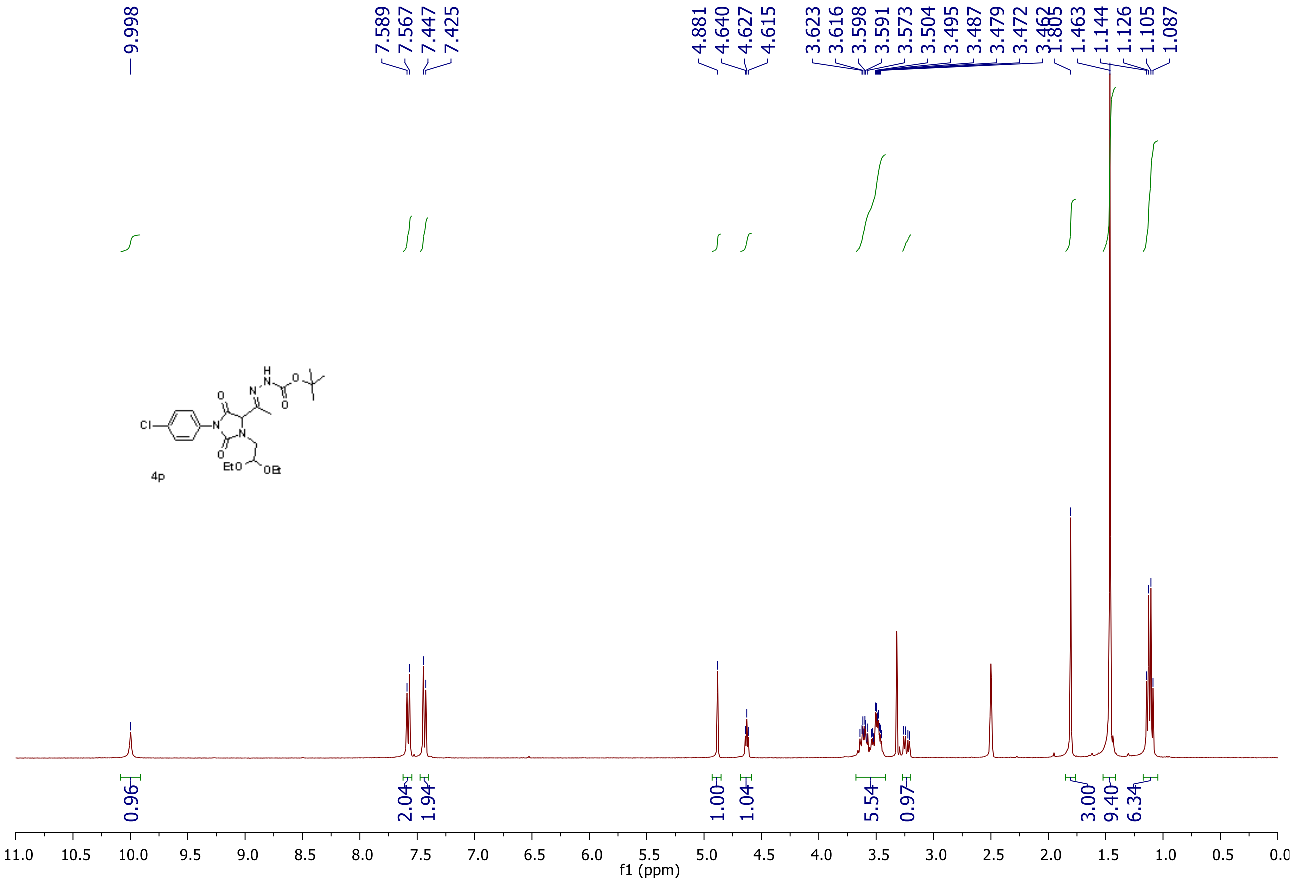
9.998
7.589
7.567
7.447
7.425
4.881
4.640
4.627
4.615
3.623
3.616
3.598
3.591
3.573
3.504
3.495
3.487
3.479
3.472
3.462
1.805
1.463
1.144
1.126
1.105
1.087
4p
0.96
2.04
1.94
1.00
1.04
5.54
0.97
3.00
9.40
6.34
f1 (ppm)

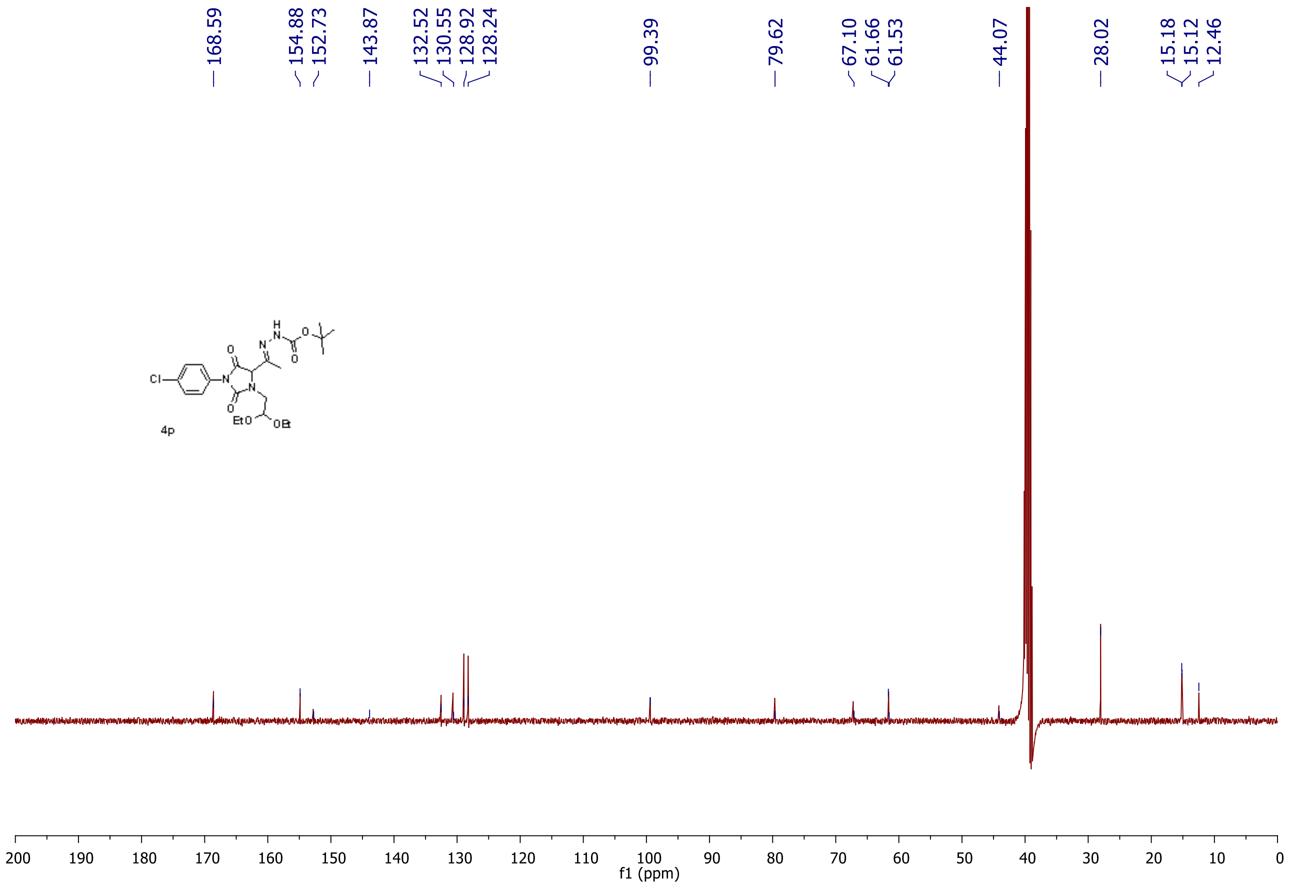
168.59
154.88
152.73
143.87
132.52
130.55
128.92
128.24
99.39
79.62
67.10
61.66
61.53
44.07
28.02
15.18
15.12
12.46
4p
f1 (ppm)

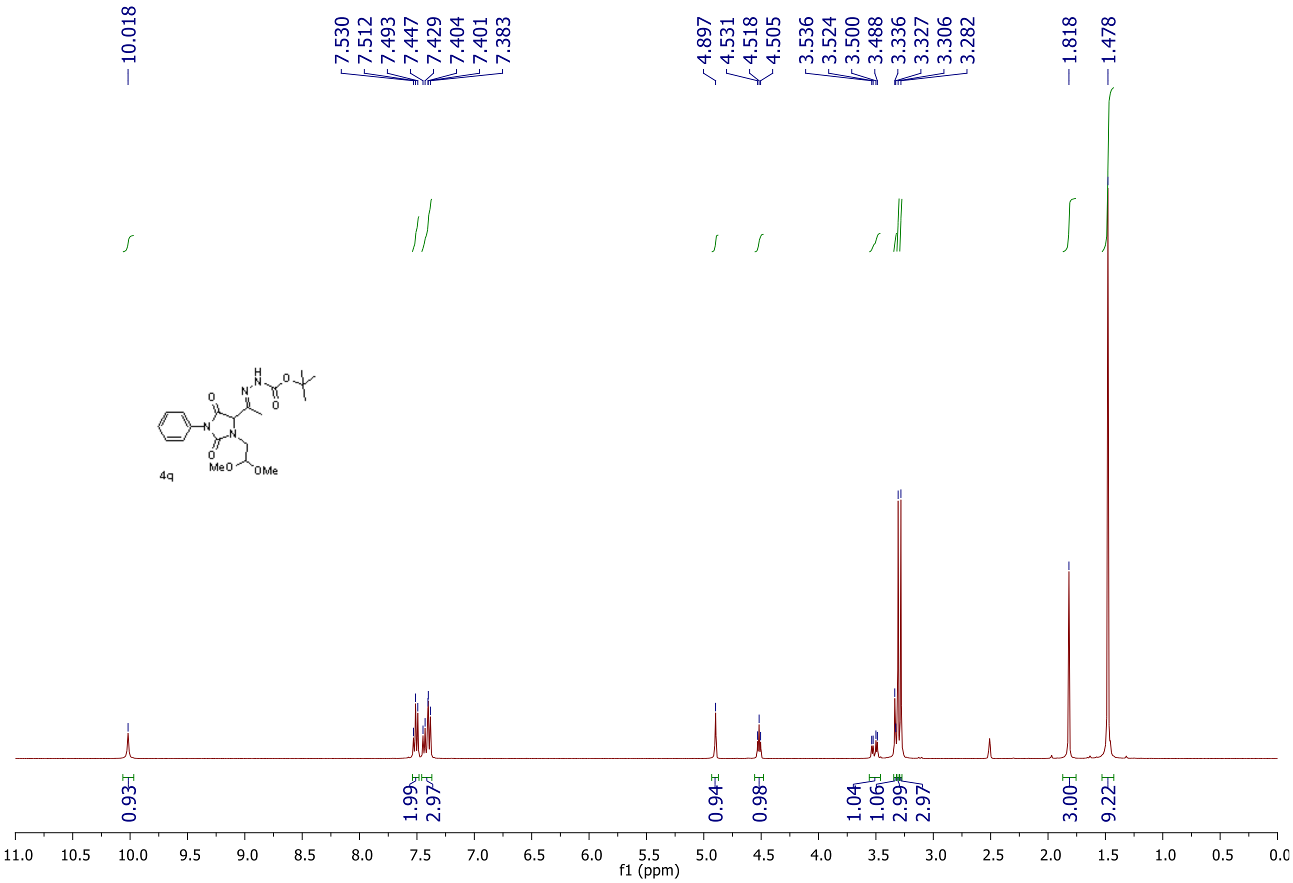
10.018
7.530 7.512 7.493 7.447 7.429 7.404 7.401 7.383
4.897 4.531 4.518 4.505
3.536 3.524 3.500 3.488 3.336 3.327 3.306 3.282
1.818
1.478
0.93 1.99 2.97 0.94 0.98 1.04 1.06 2.99 2.97 3.00 9.22
4q
MeO
OMe
11.0 10.5 10.0 9.5 9.0 8.5 8.0 7.5 7.0 6.5 6.0 5.5 5.0 4.5 4.0 3.5 3.0 2.5 2.0 1.5 1.0 0.5 0.0
f1 (ppm)

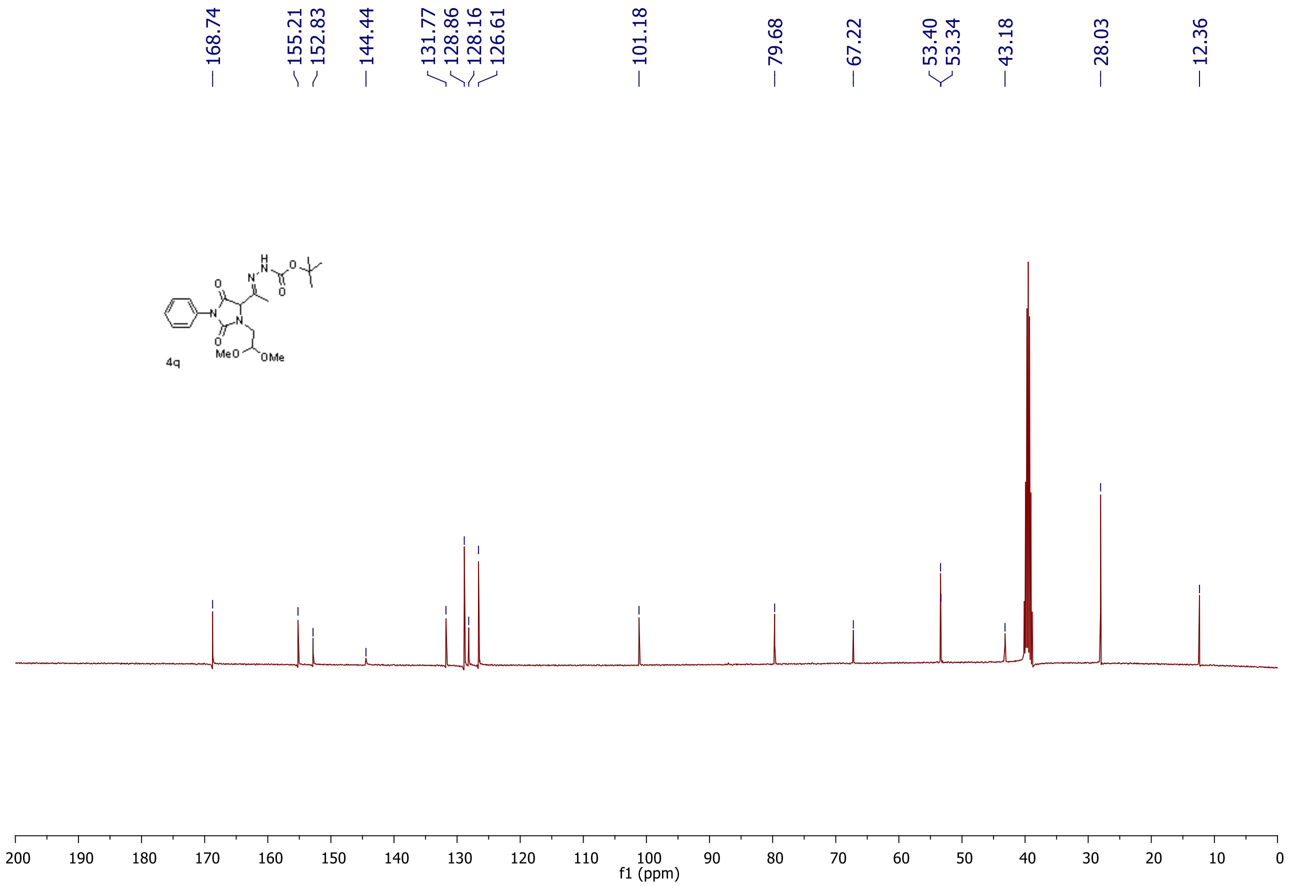
168.74
155.21 152.83
144.44
131.77 128.86 128.16 126.61
101.18
79.68
67.22
53.40 53.34
43.18
28.03
12.36
4q
MeO
OMe
200 190 180 170 160 150 140 130 120 110 100 90 80 70 60 50 40 30 20 10 0
f1 (ppm)

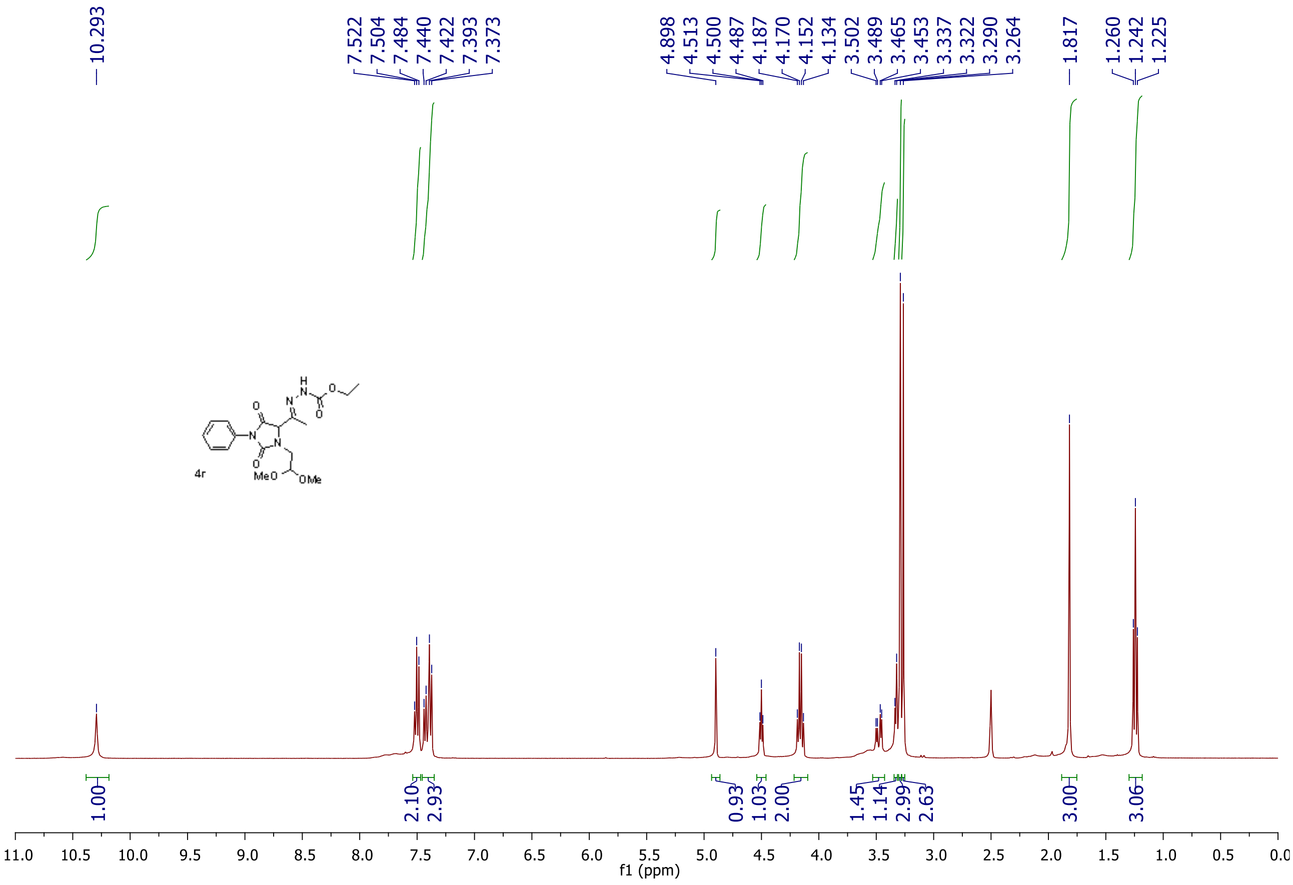
10.293
7.522
7.504
7.484
7.440
7.422
7.393
7.373
4.898
4.513
4.500
4.487
4.187
4.170
4.152
4.134
3.502
3.489
3.465
3.453
3.337
3.322
3.290
3.264
1.817
1.260
1.242
1.225
1.00
2.10
2.93
0.93
1.03
2.00
1.45
1.14
2.99
2.63
3.00
3.06
4r
f1 (ppm)

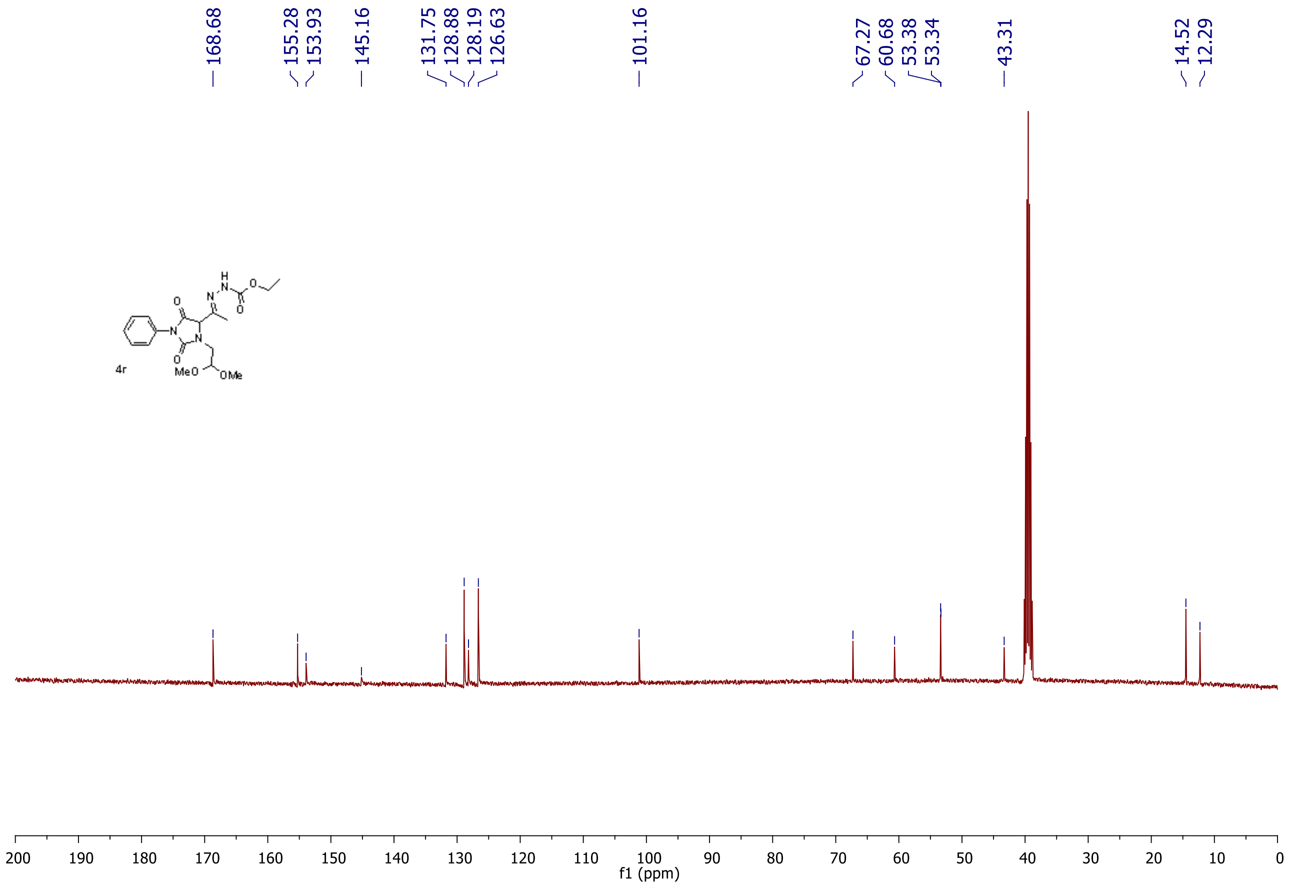
168.68
155.28
153.93
145.16
131.75
128.88
128.19
126.63
101.16
67.27
60.68
53.38
53.34
43.31
14.52
12.29
4r
f1 (ppm)

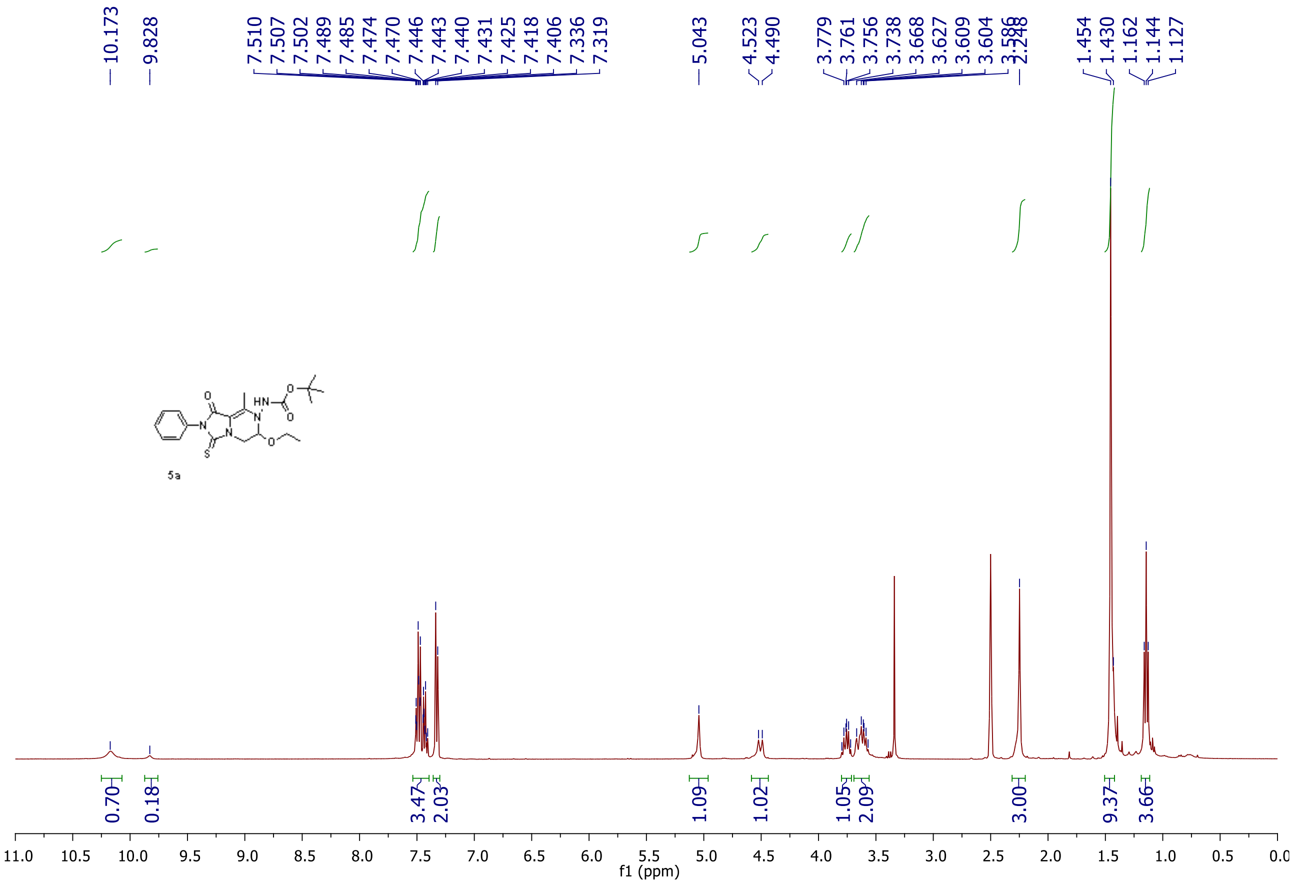
10.173
9.828
7.510
7.507
7.502
7.489
7.485
7.474
7.470
7.446
7.443
7.440
7.431
7.425
7.418
7.406
7.336
7.319
5.043
4.523
4.490
3.779
3.761
3.756
3.738
3.668
3.627
3.609
3.604
2.248
1.454
1.430
1.162
1.144
1.127
5a
0.70
0.18
3.47
2.03
1.09
1.02
1.05
2.09
3.00
9.37
3.66
f1 (ppm)

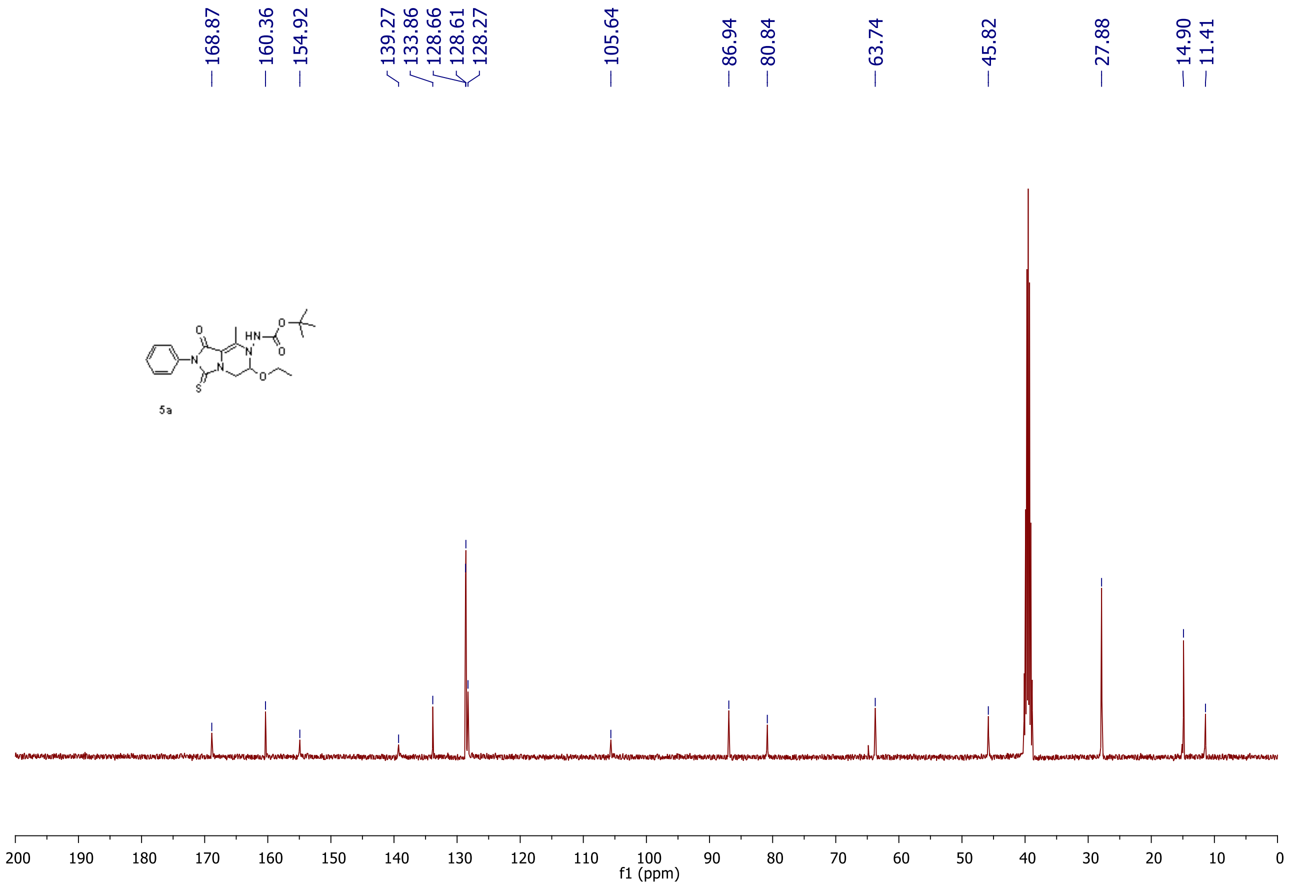
168.87
160.36
154.92
139.27
133.86
128.66
128.61
128.27
105.64
86.94
80.84
63.74
45.82
27.88
14.90
11.41
5a
f1 (ppm)

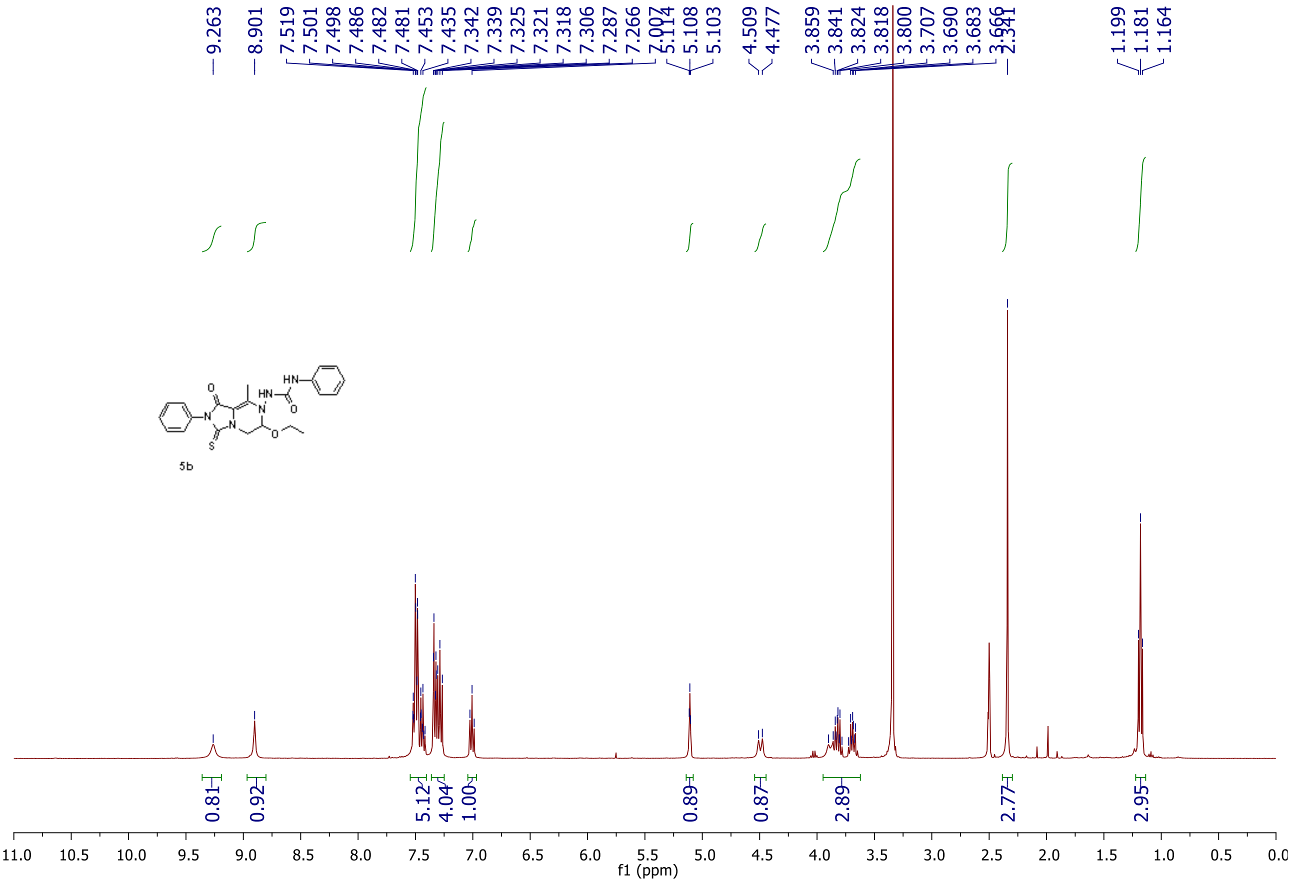
9.263
8.901
7.519
7.501
7.498
7.486
7.482
7.481
7.453
7.435
7.342
7.339
7.325
7.321
7.318
7.306
7.287
7.266
7.007
5.114
5.108
5.103
4.509
4.477
3.859
3.841
3.824
3.818
3.800
3.707
3.690
3.683
3.666
2.341
1.199
1.181
1.164
HN
HN
O
N
N
N
O
O
S
5b
0.81
0.92
5.12
4.04
1.00
0.89
0.87
2.89
2.77
2.95
f1 (ppm)

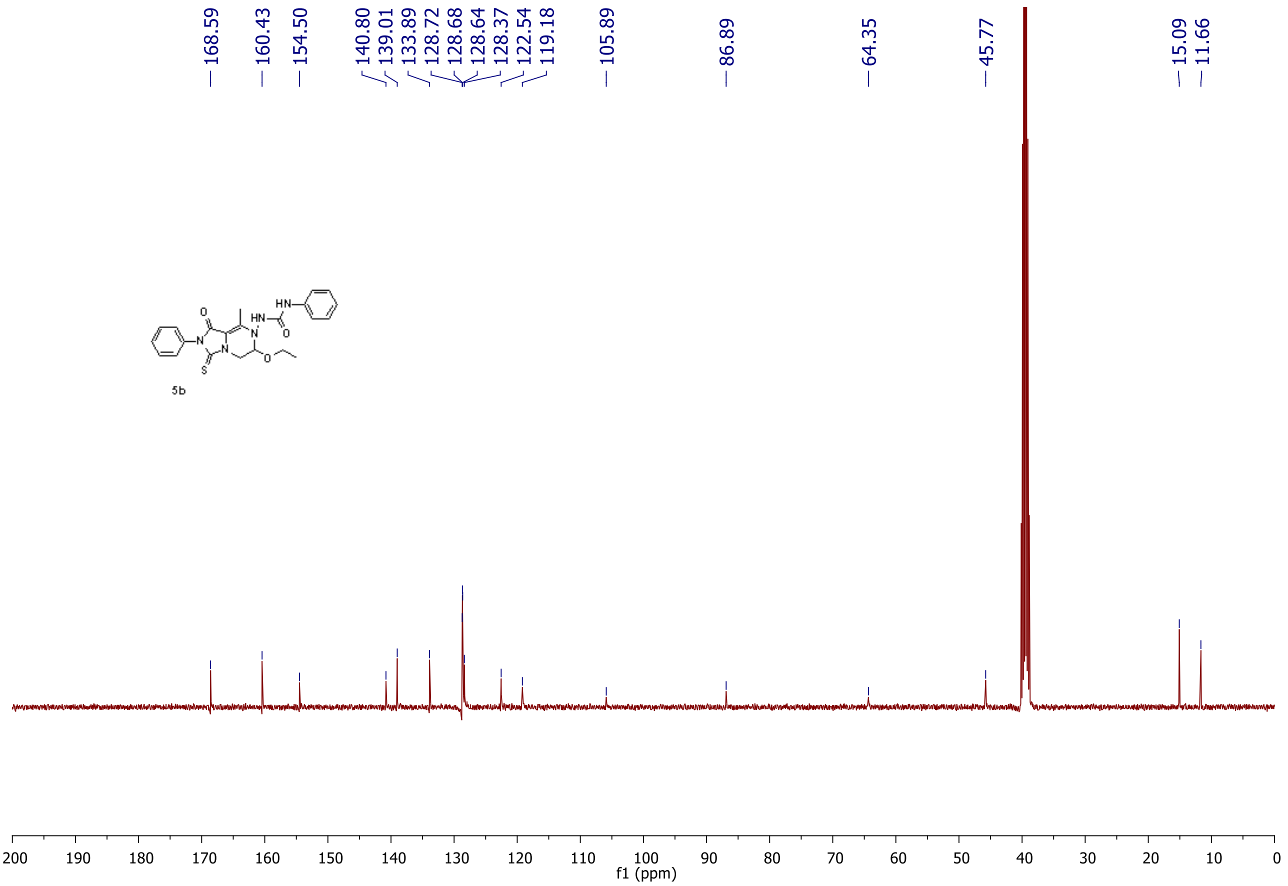
168.59
160.43
154.50
140.80
139.01
133.89
128.72
128.68
128.64
128.37
122.54
119.18
105.89
86.89
64.35
45.77
15.09
11.66
HN
HN
O
N
N
N
O
O
S
5b
f1 (ppm)

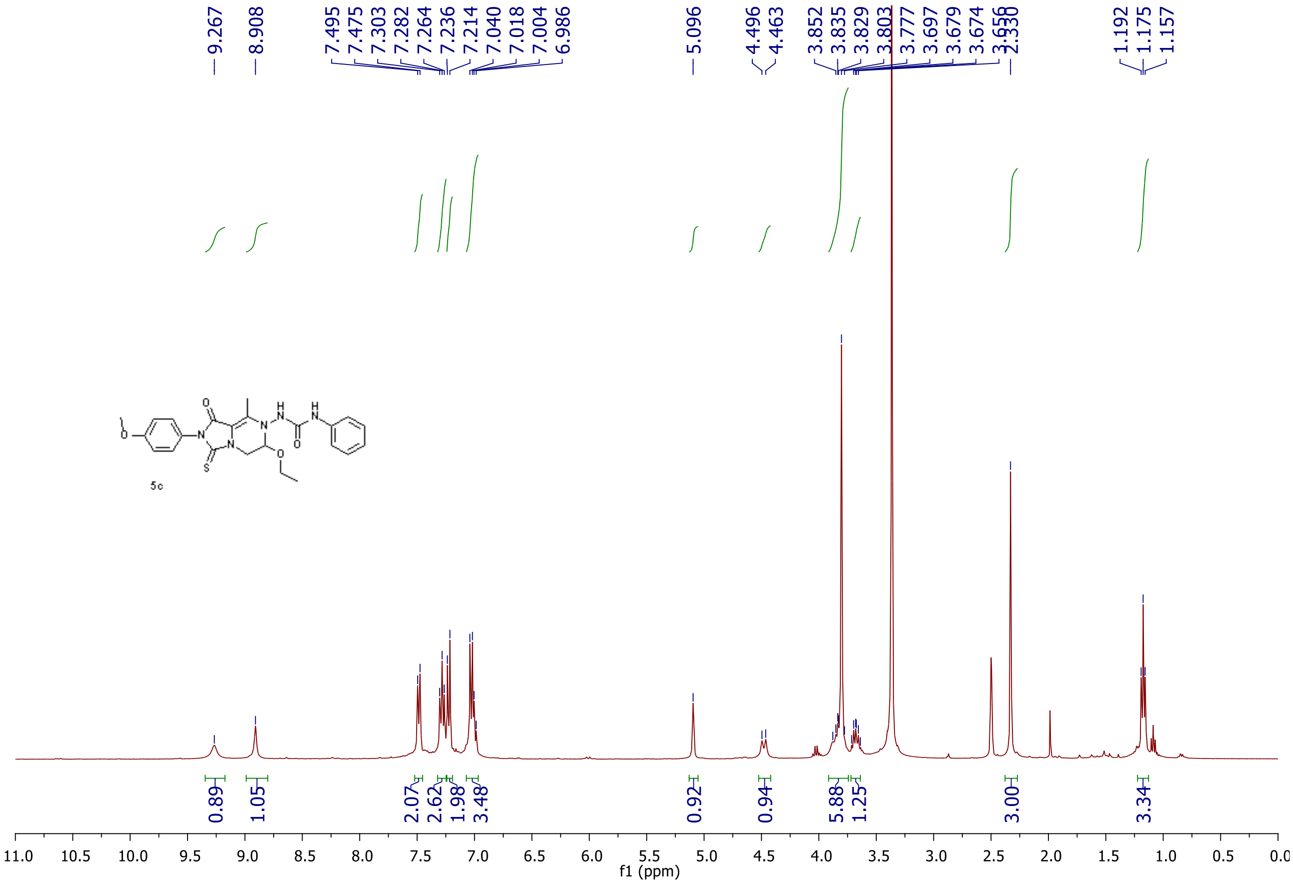
9.267
8.908
7.495
7.475
7.303
7.282
7.264
7.236
7.214
7.040
7.018
7.004
6.986
5.096
4.496
4.463
3.852
3.835
3.829
3.803
3.777
3.697
3.679
3.674
3.656
2.330
1.192
1.175
1.157
5c
0.89
1.05
2.07
2.62
1.98
3.48
0.92
0.94
5.88
1.25
3.00
3.34
f1 (ppm)

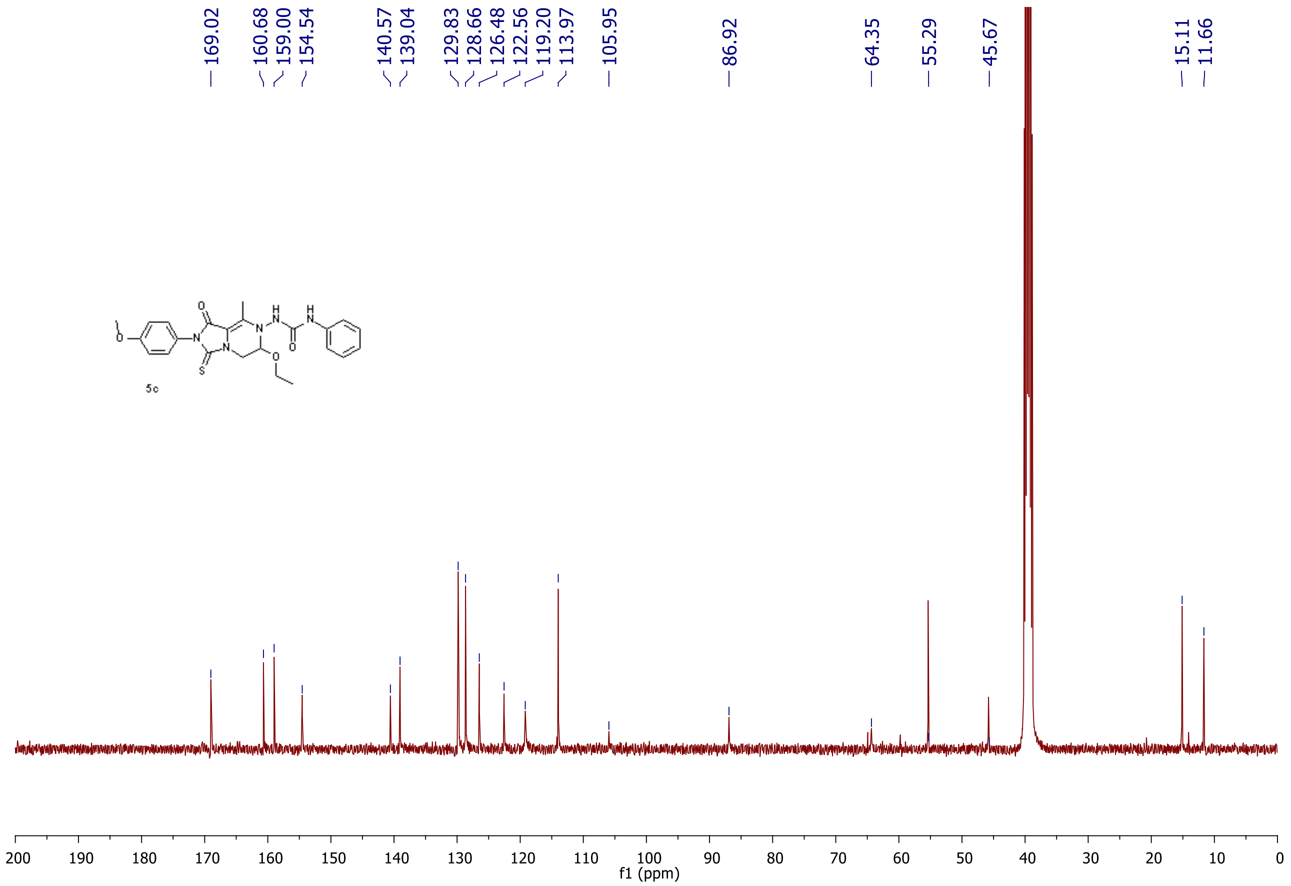
169.02
160.68
159.00
154.54
140.57
139.04
129.83
128.66
126.48
122.56
119.20
113.97
105.95
86.92
64.35
55.29
45.67
15.11
11.66
5c
f1 (ppm)

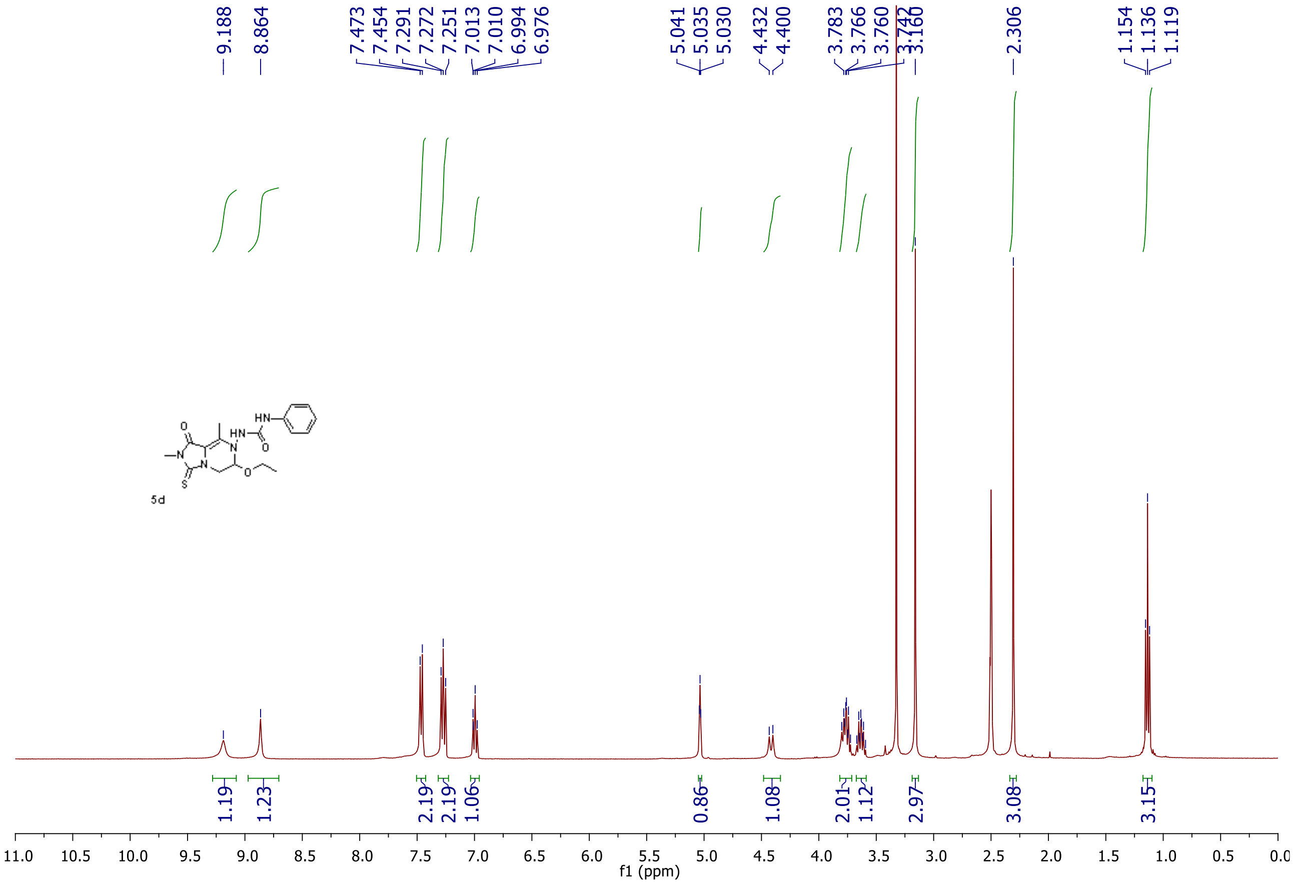
9.188
8.864
7.473
7.454
7.291
7.272
7.251
7.013
7.010
6.994
6.976
5.041
5.035
5.030
4.432
4.400
3.783
3.766
3.760
3.742
3.160
2.306
1.154
1.136
1.119
1.19
1.23
2.19
2.19
1.06
0.86
1.08
2.01
1.12
2.97
3.08
3.15
5d
f1 (ppm)

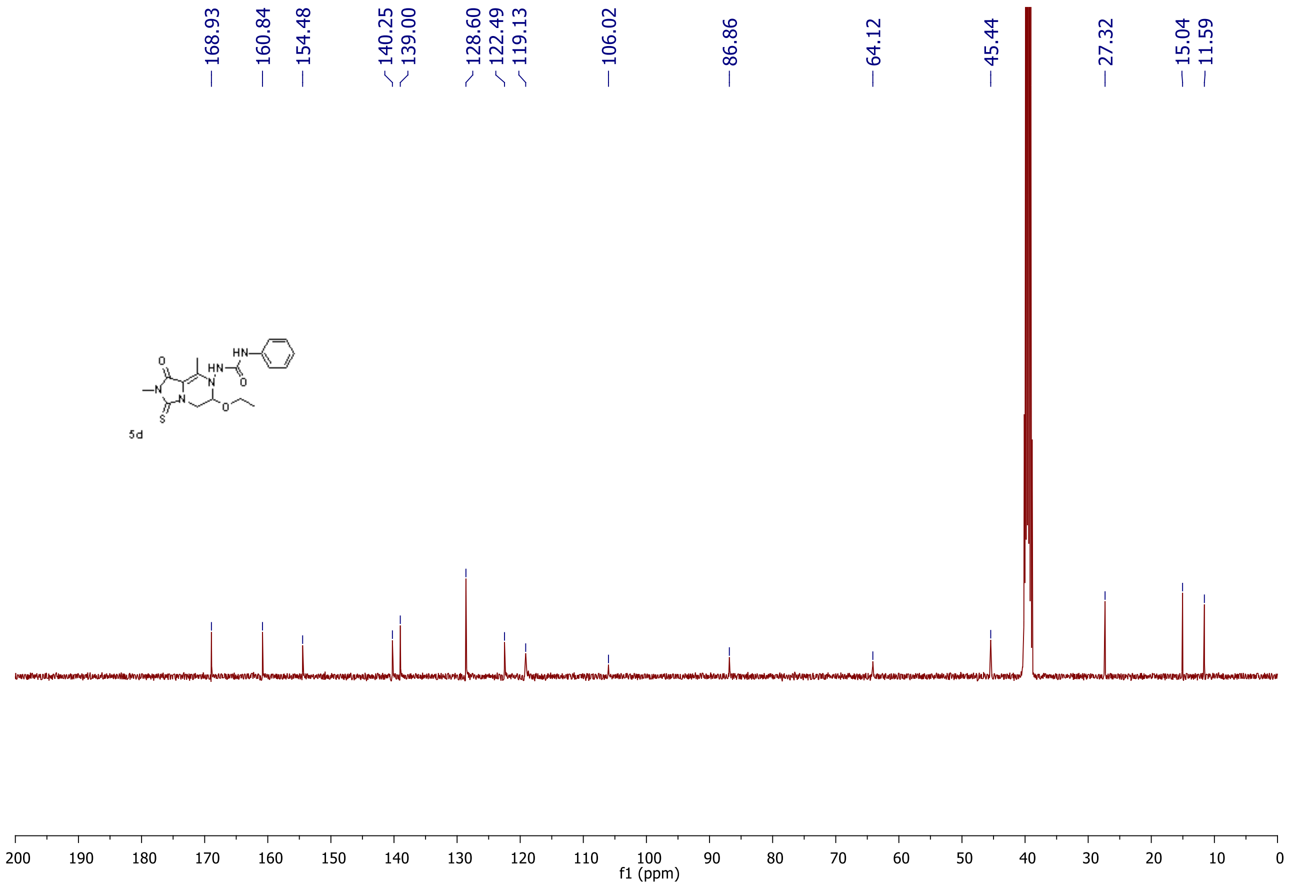
168.93
160.84
154.48
140.25
139.00
128.60
122.49
119.13
106.02
86.86
64.12
45.44
27.32
15.04
11.59
5d
f1 (ppm)

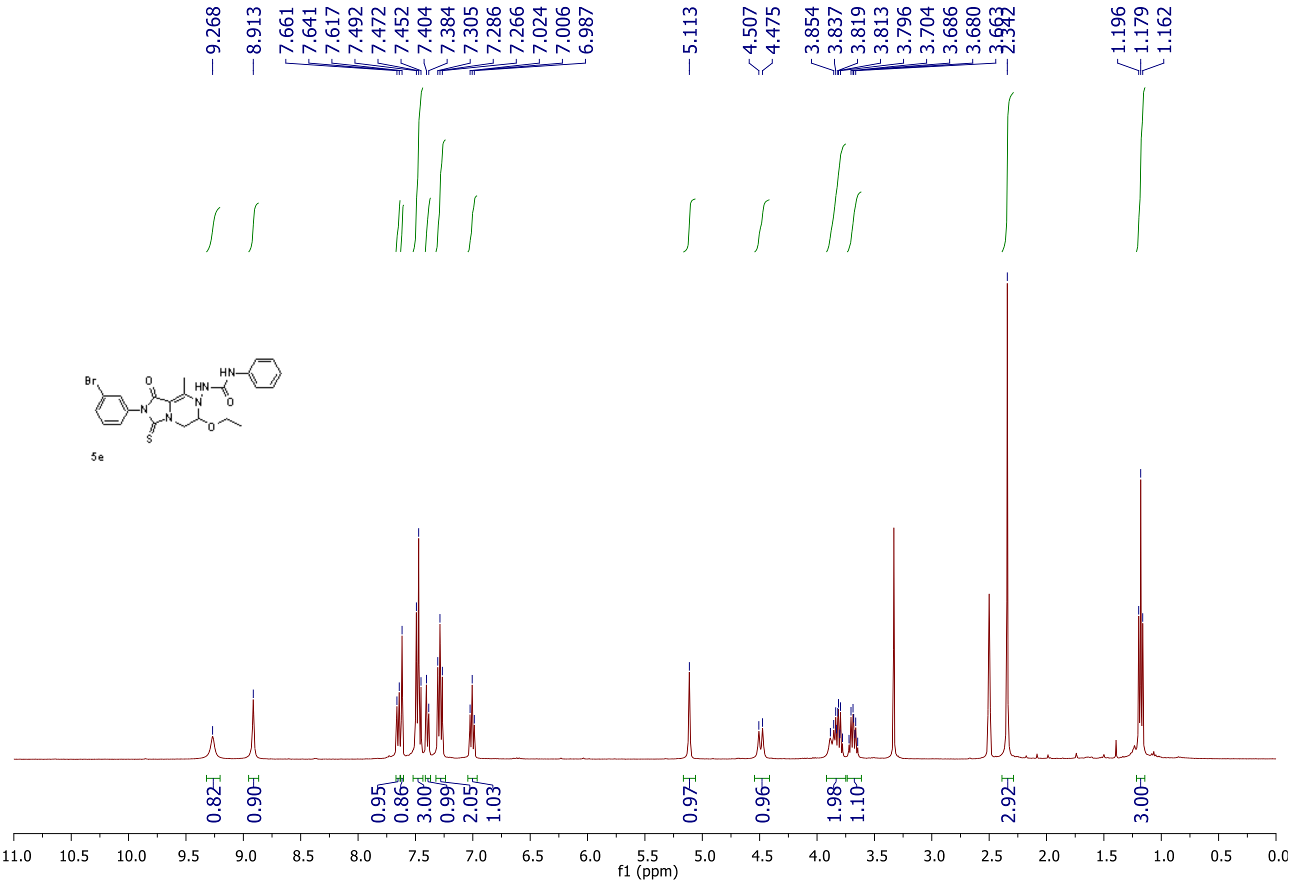
9.268
8.913
7.661
7.641
7.617
7.492
7.472
7.452
7.404
7.384
7.305
7.286
7.266
7.024
7.006
6.987
5.113
4.507
4.475
3.854
3.837
3.819
3.813
3.796
3.704
3.686
3.680
3.663
2.342
1.196
1.179
1.162
Br
O
HN
HN
N
N
N
O
O
S
5e
0.82
0.90
0.95
0.86
3.00
0.99
2.05
1.03
0.97
0.96
1.98
1.10
2.92
3.00
11.0
10.5
10.0
9.5
9.0
8.5
8.0
7.5
7.0
6.5
6.0
5.5
5.0
4.5
4.0
3.5
3.0
2.5
2.0
1.5
1.0
0.5
0.0
f1 (ppm)

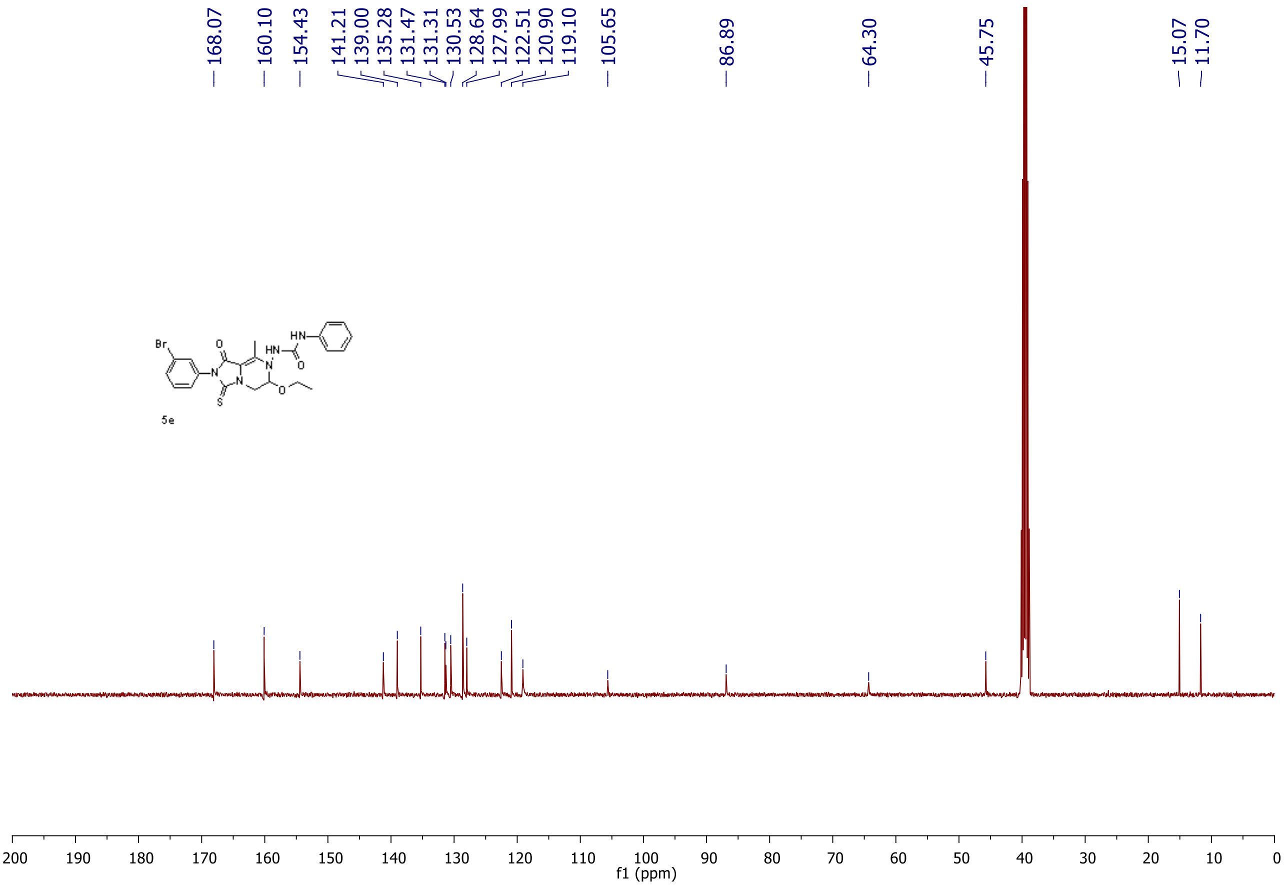
168.07
160.10
154.43
141.21
139.00
135.28
131.47
131.31
130.53
128.64
127.99
122.51
120.90
119.10
105.65
86.89
64.30
45.75
15.07
11.70
Br
O
HN
HN
N
N
N
O
O
S
5e
200
190
180
170
160
150
140
130
120
110
100
90
80
70
60
50
40
30
20
10
0
f1 (ppm)

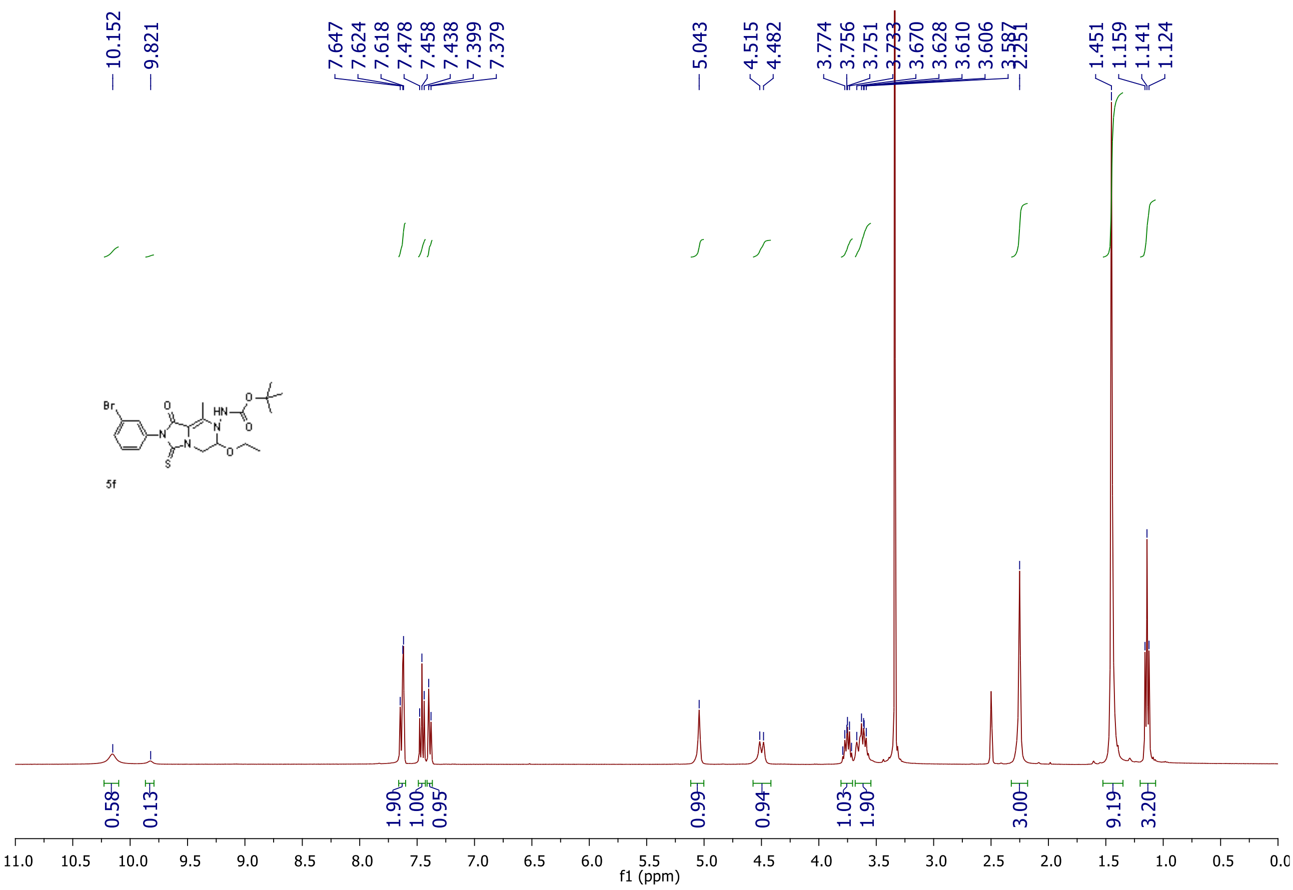

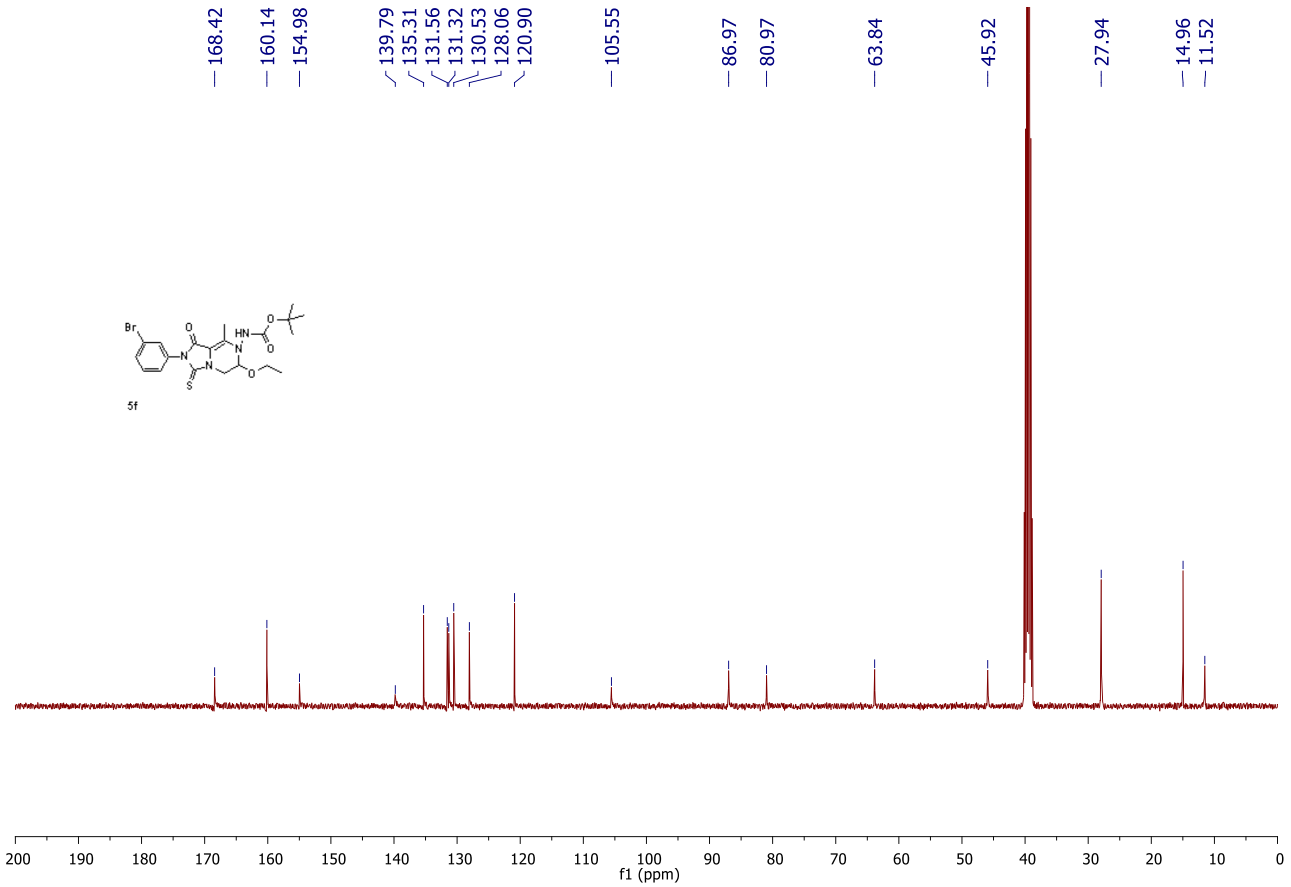

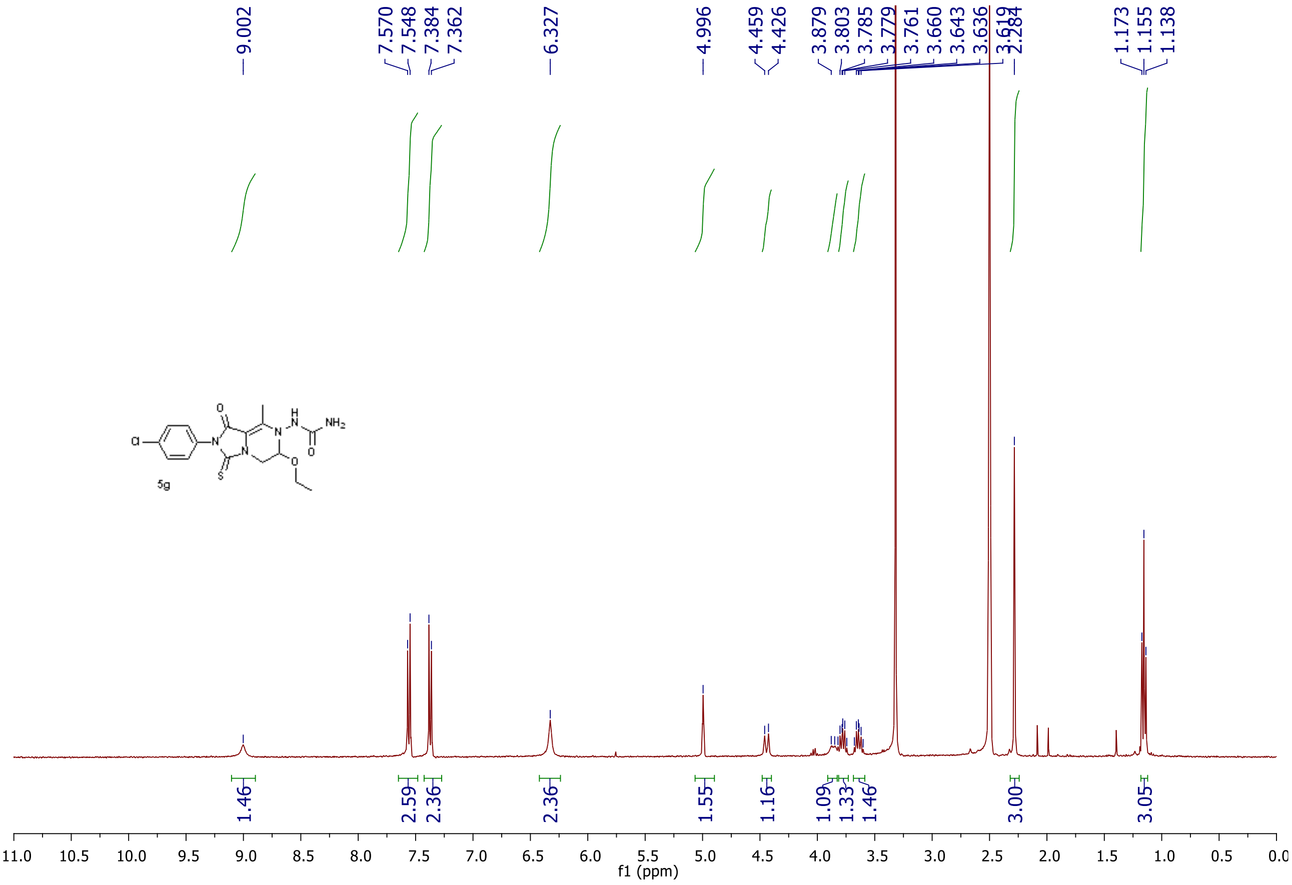
9.002
7.570
7.548
7.384
7.362
6.327
4.996
4.459
4.426
3.879
3.803
3.785
3.779
3.761
3.660
3.643
3.636
3.619
2.284
1.173
1.155
1.138
5g
1.46
2.59
2.36
2.36
1.55
1.16
1.09
1.33
1.46
3.00
3.05
f1 (ppm)

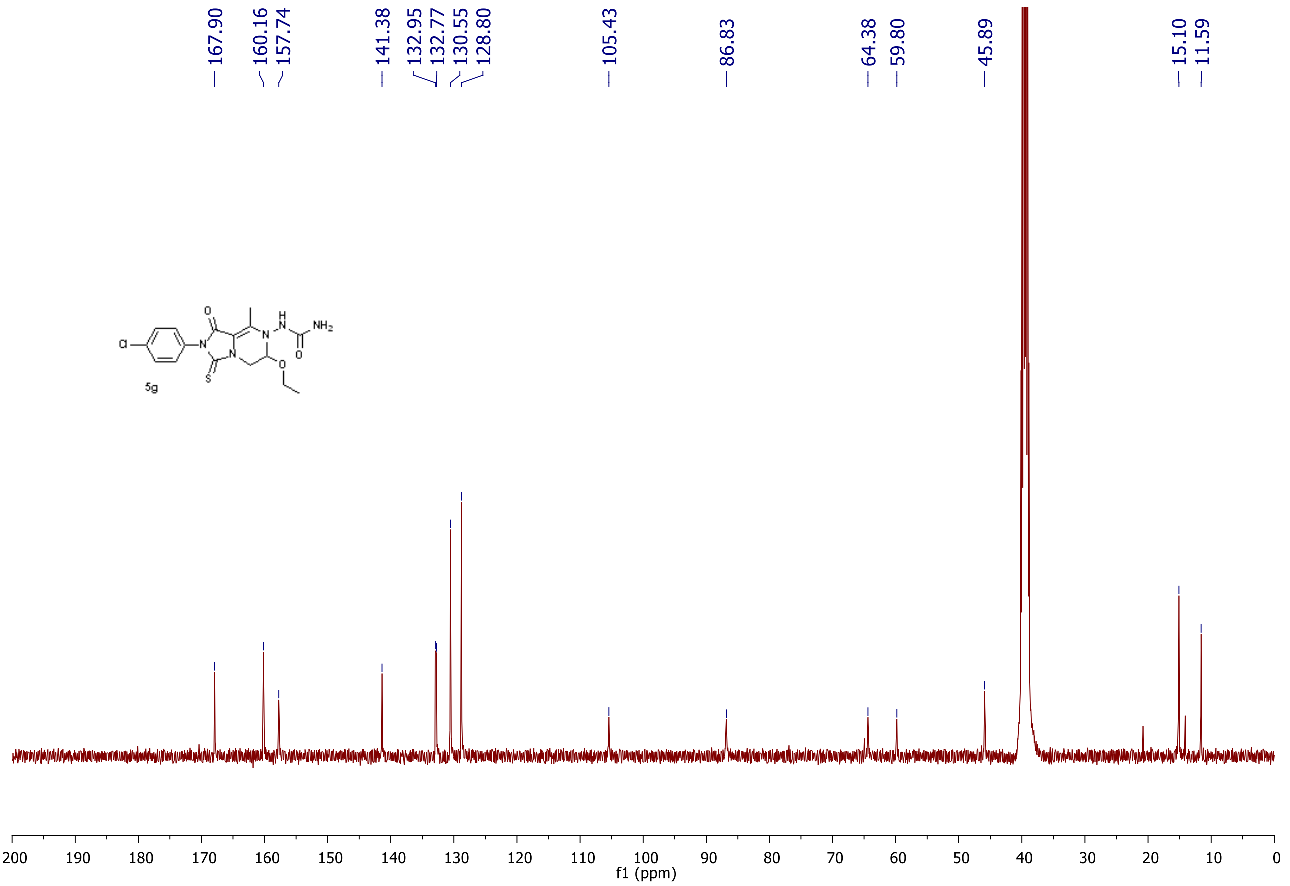
167.90
160.16
157.74
141.38
132.95
132.77
130.55
128.80
105.43
86.83
64.38
59.80
45.89
15.10
11.59
5g
f1 (ppm)

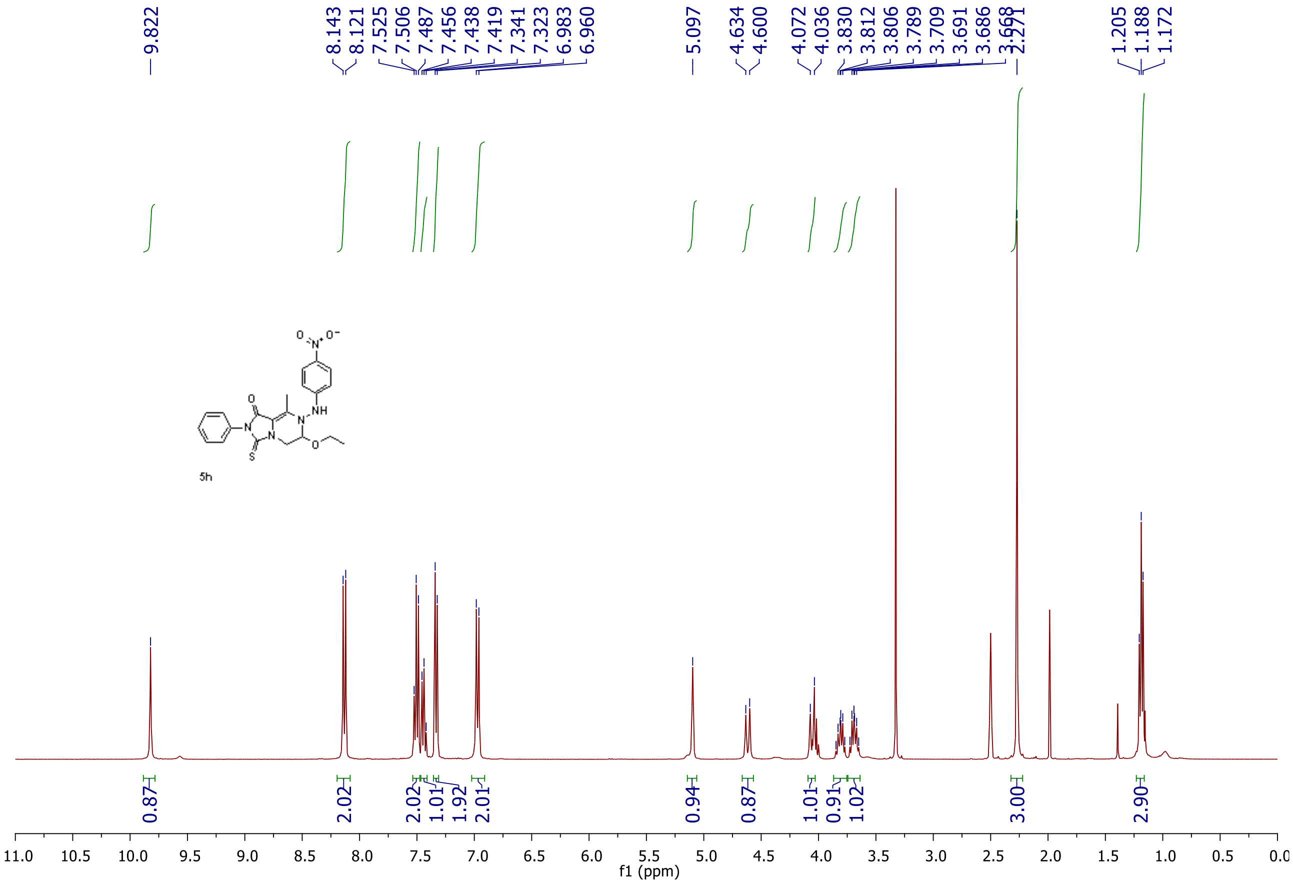
9.822
8.143
8.121
7.525
7.506
7.487
7.456
7.438
7.419
7.341
7.323
6.983
6.960
5.097
4.634
4.600
4.072
4.036
3.830
3.812
3.806
3.789
3.709
3.691
3.686
2.271
1.205
1.188
1.172
5h
0.87
2.02
2.02
1.01
1.92
2.01
0.94
0.87
1.01
0.91
1.02
3.00
2.90
f1 (ppm)

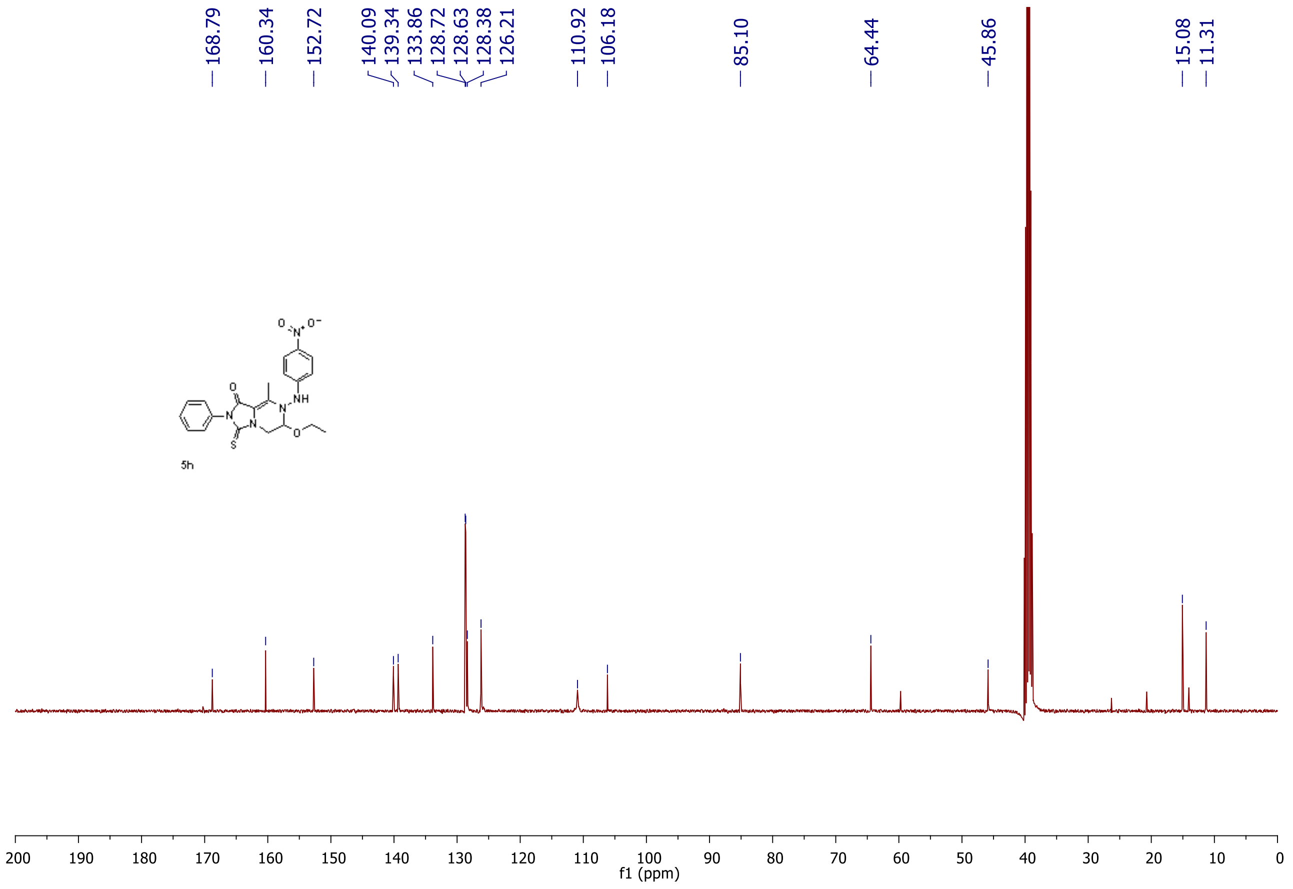
168.79
160.34
152.72
140.09
139.34
133.86
128.72
128.63
128.38
126.21
110.92
106.18
85.10
64.44
45.86
15.08
11.31
5h
f1 (ppm)

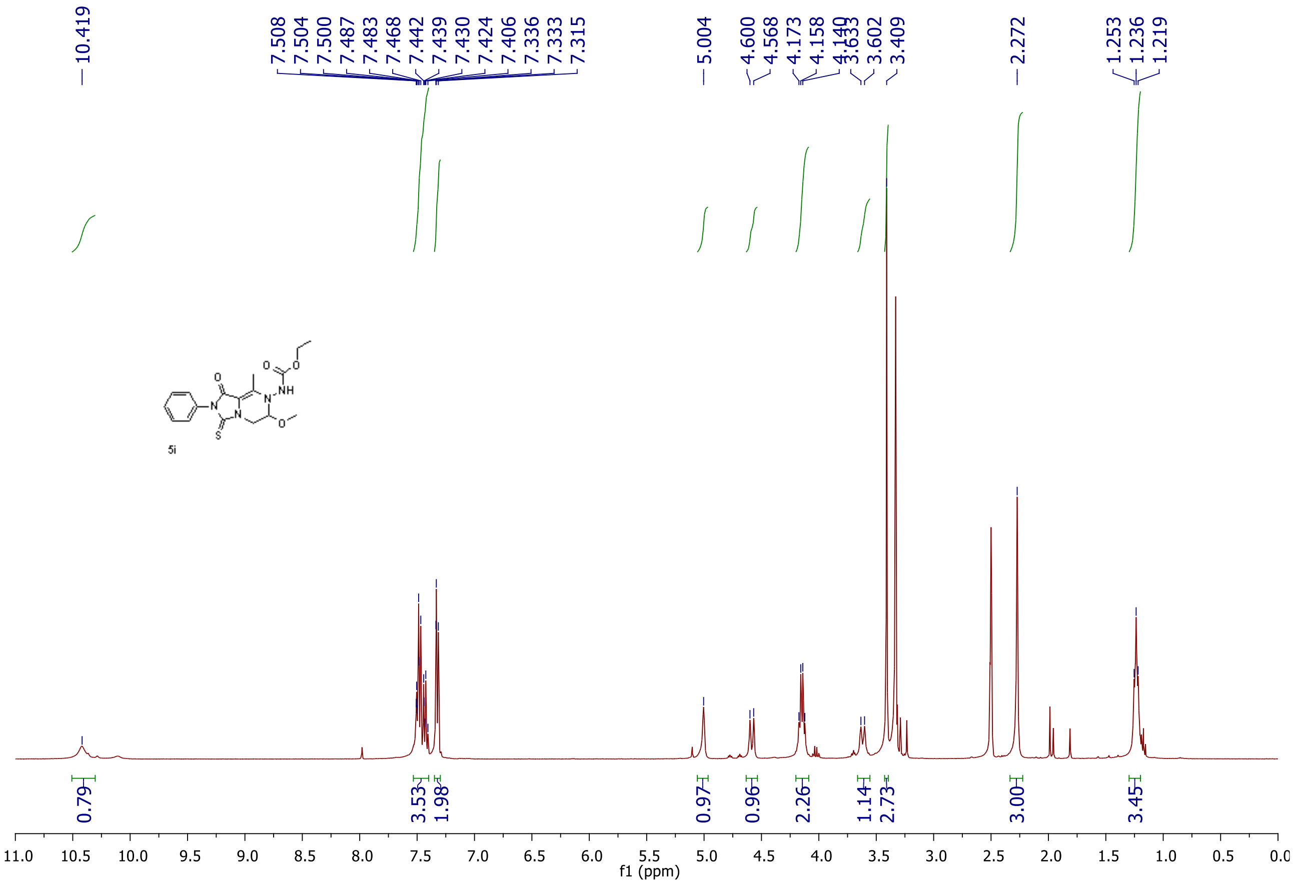

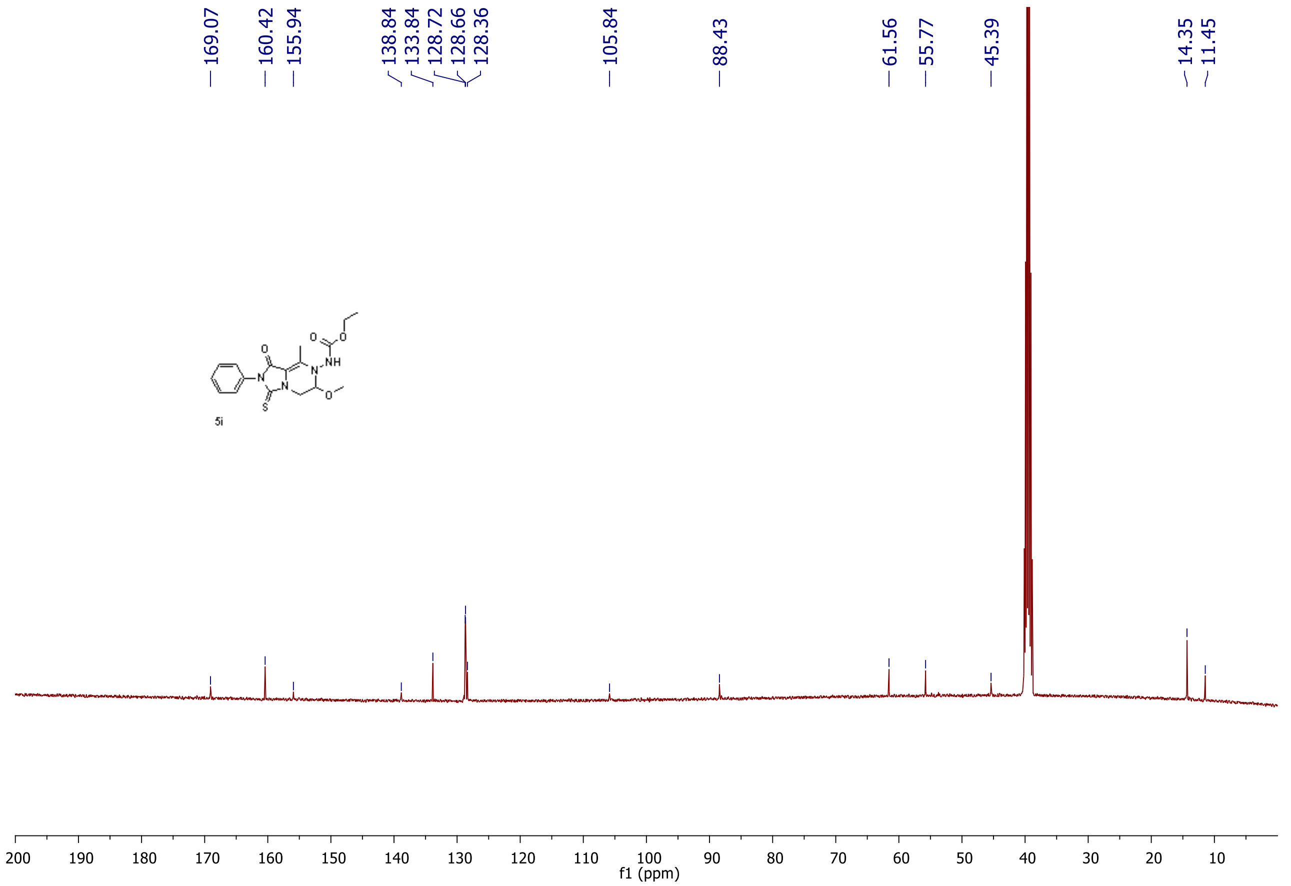

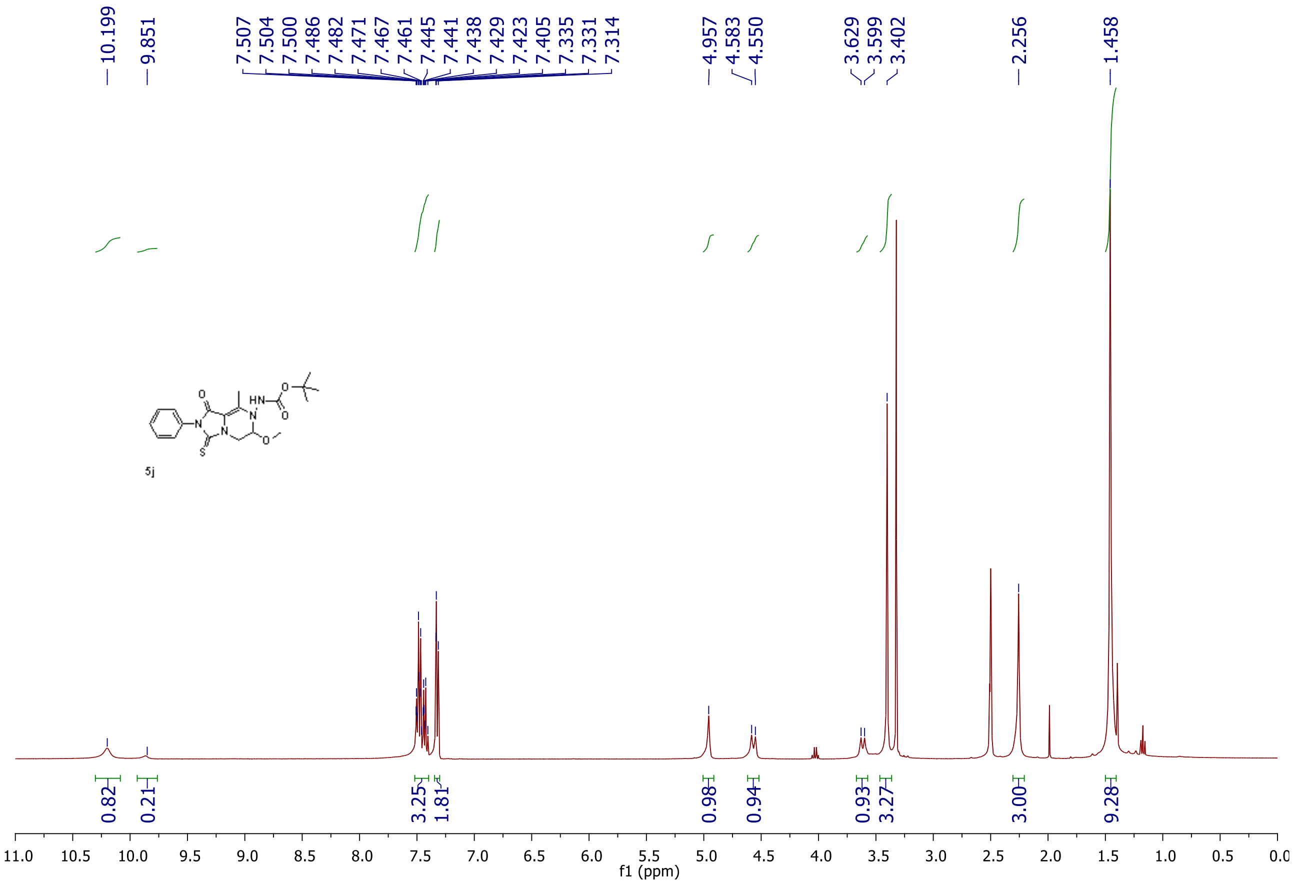
10.199
9.851
7.507
7.504
7.500
7.486
7.482
7.471
7.467
7.461
7.445
7.441
7.438
7.429
7.423
7.405
7.335
7.331
7.314
4.957
4.583
4.550
3.629
3.599
3.402
2.256
1.458
5j
0.82
0.21
3.25
1.81
0.98
0.94
0.93
3.27
3.00
9.28
f1 (ppm)

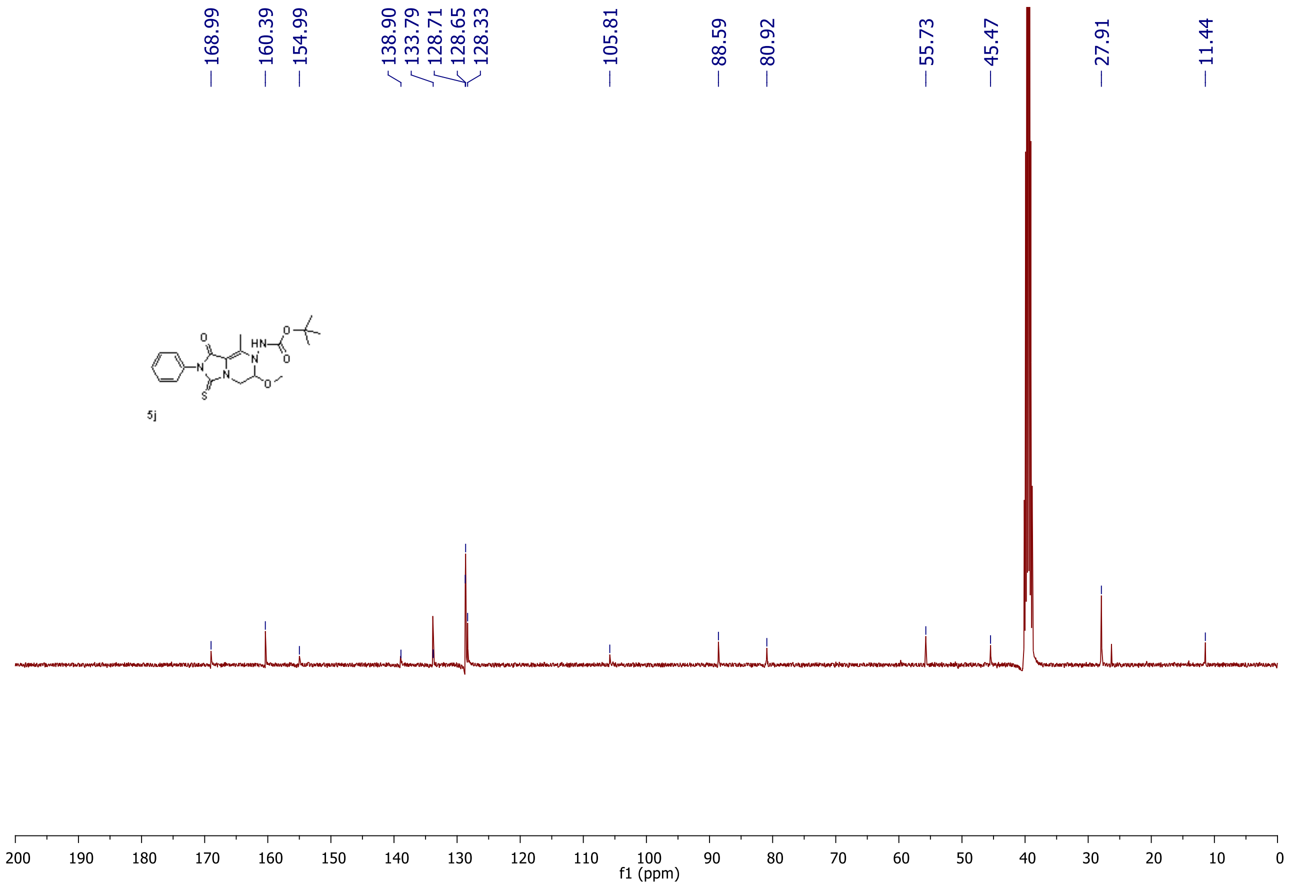
168.99
160.39
154.99
138.90
133.79
128.71
128.65
128.33
105.81
88.59
80.92
55.73
45.47
27.91
11.44
5j
f1 (ppm)

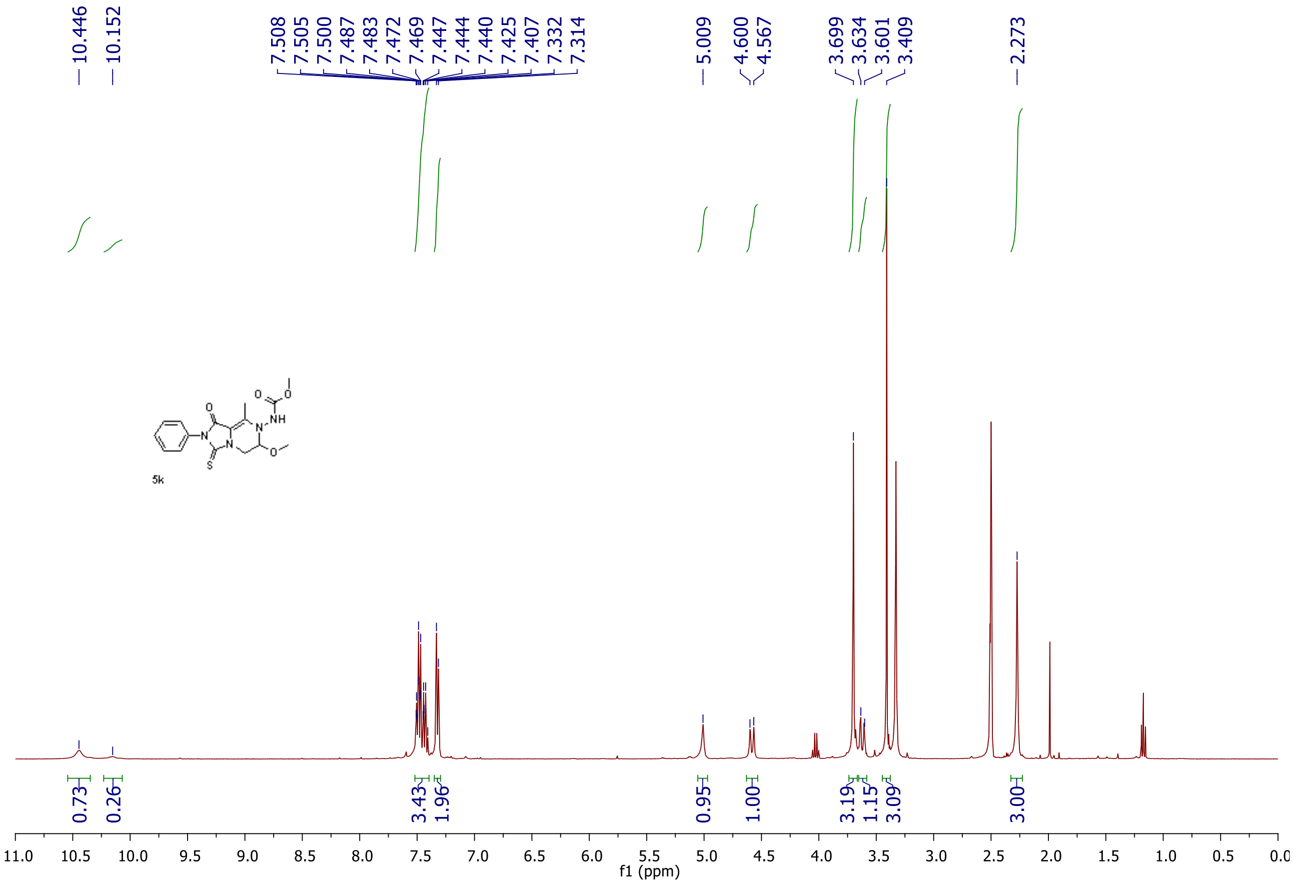
10.446
10.152
7.508
7.505
7.500
7.487
7.483
7.472
7.469
7.447
7.444
7.440
7.425
7.407
7.332
7.314
5.009
4.600
4.567
3.699
3.634
3.601
3.409
2.273
5k
0.73
0.26
3.43
1.96
0.95
1.00
3.19
1.15
3.09
3.00
f1 (ppm)

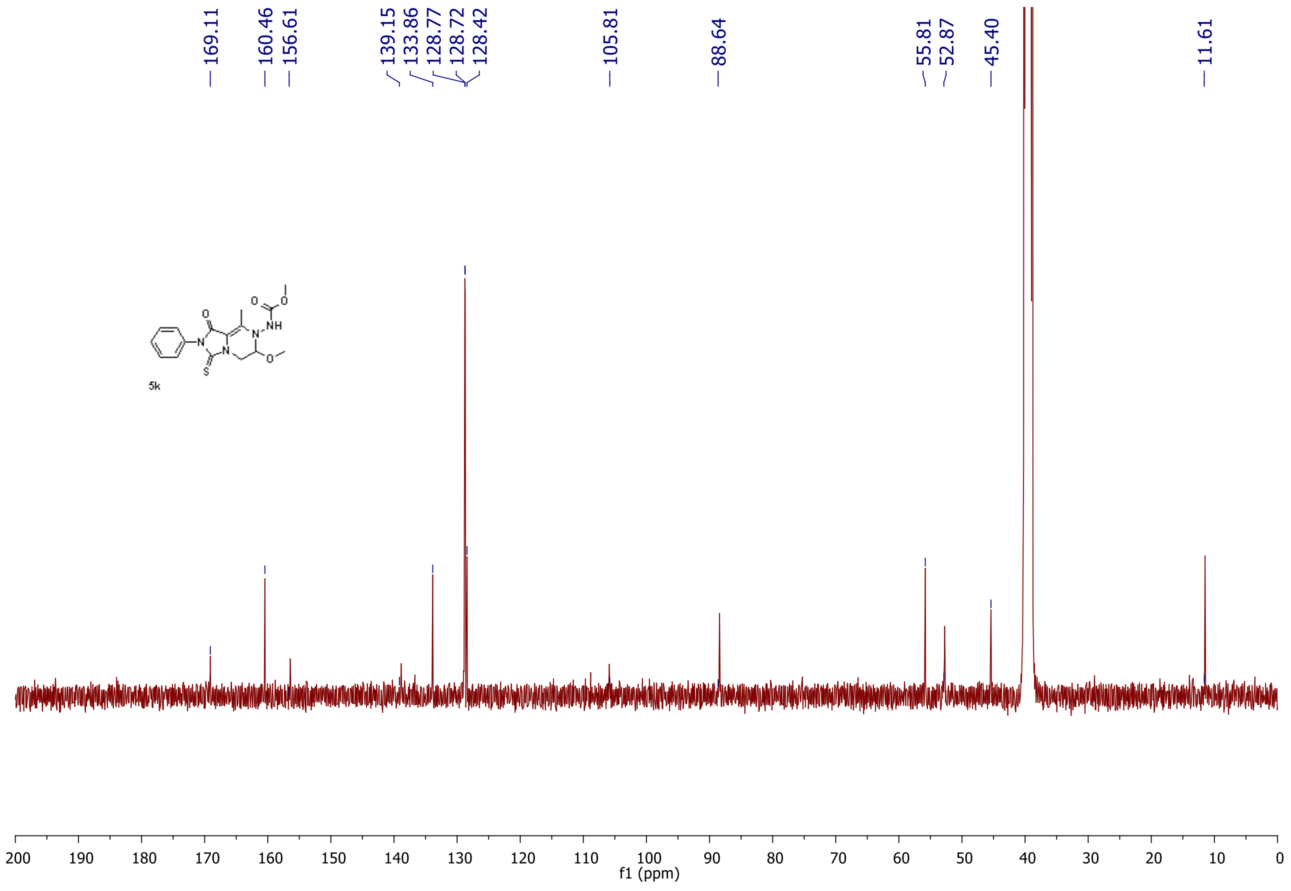
169.11
160.46
156.61
139.15
133.86
128.77
128.72
128.42
105.81
88.64
55.81
52.87
45.40
11.61
5k
f1 (ppm)

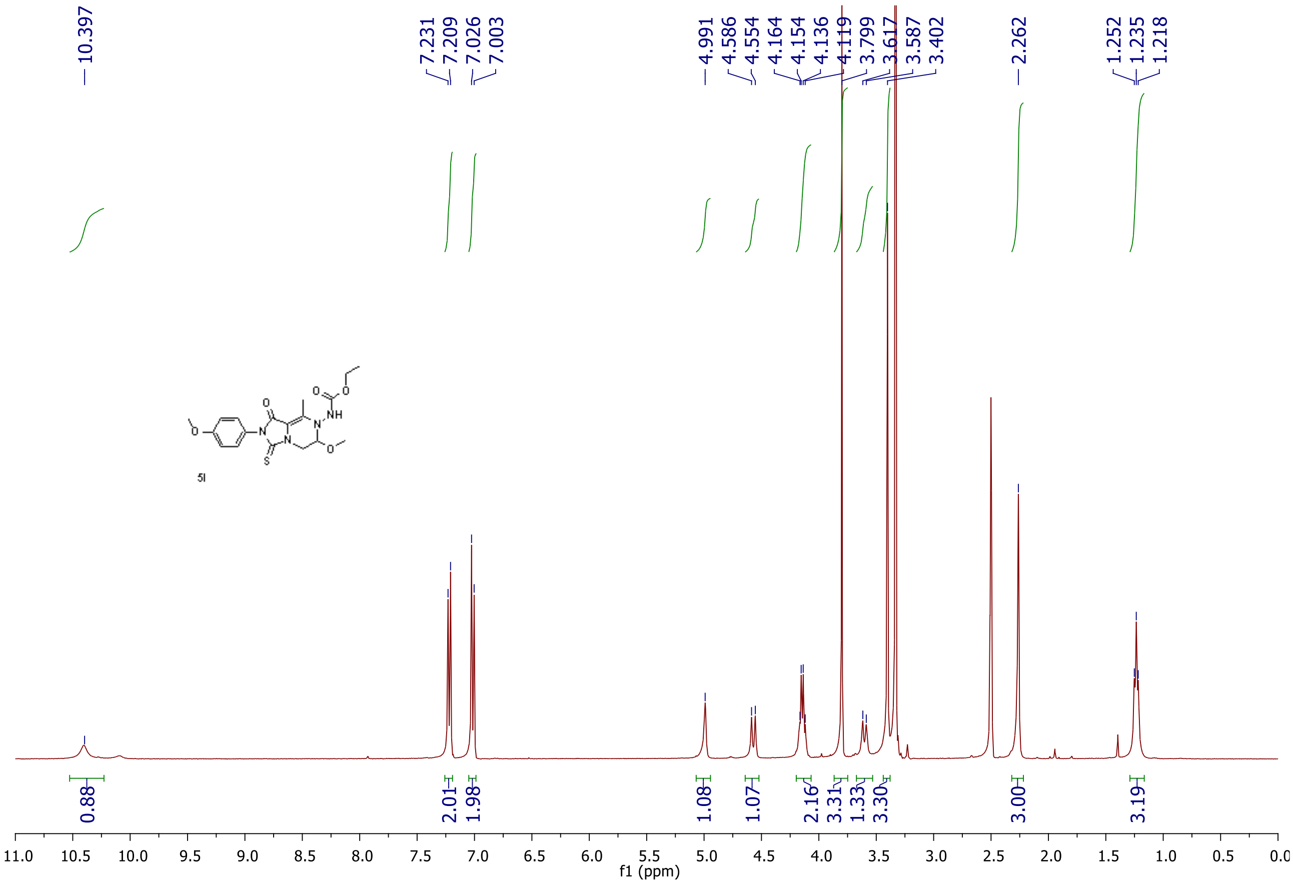
10.397
7.231
7.209
7.026
7.003
4.991
4.586
4.554
4.164
4.154
4.136
4.119
3.799
3.617
3.587
3.402
2.262
1.252
1.235
1.218
0.88
2.01
1.98
1.08
1.07
2.16
3.31
1.33
3.30
3.00
3.19
5l
f1 (ppm)

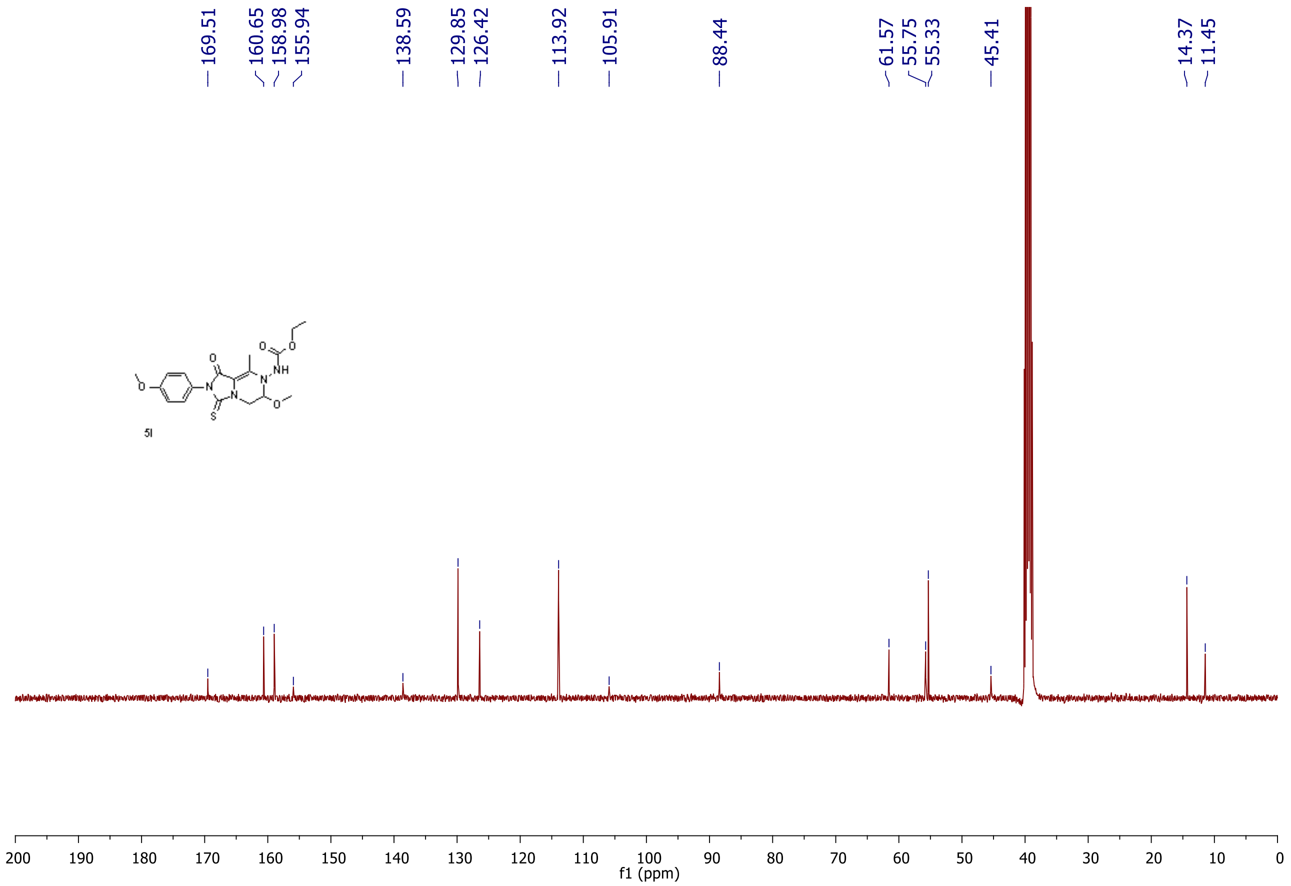
169.51
160.65
158.98
155.94
138.59
129.85
126.42
113.92
105.91
88.44
61.57
55.75
55.33
45.41
14.37
11.45
5l
f1 (ppm)

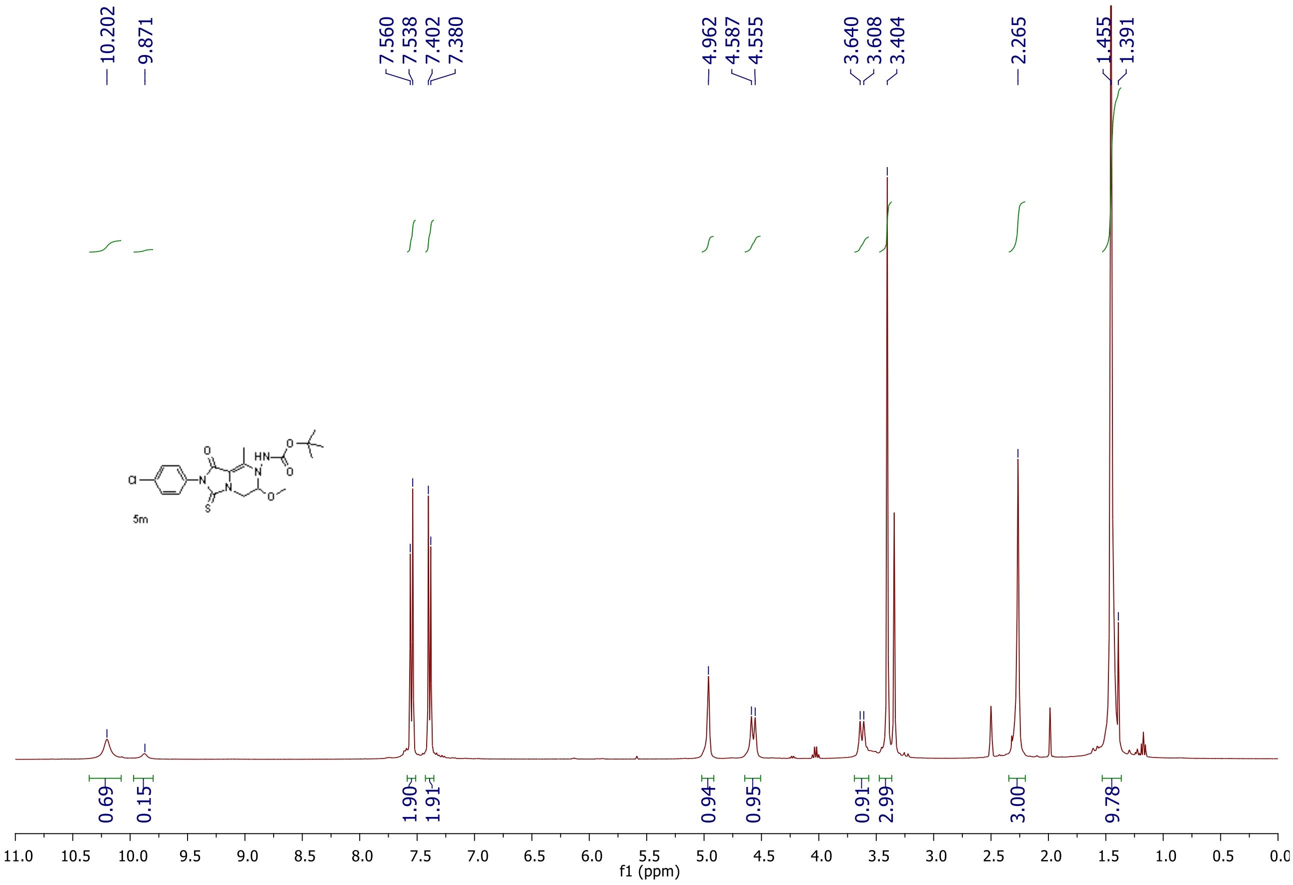
10.202
9.871
7.560
7.538
7.402
7.380
4.962
4.587
4.555
3.640
3.608
3.404
2.265
1.455
1.391
5m
0.69
0.15
1.90
1.91
0.94
0.95
0.91
2.99
3.00
9.78
11.0 10.5 10.0 9.5 9.0 8.5 8.0 7.5 7.0 6.5 6.0 5.5 5.0 4.5 4.0 3.5 3.0 2.5 2.0 1.5 1.0 0.5 0.0
f1 (ppm)

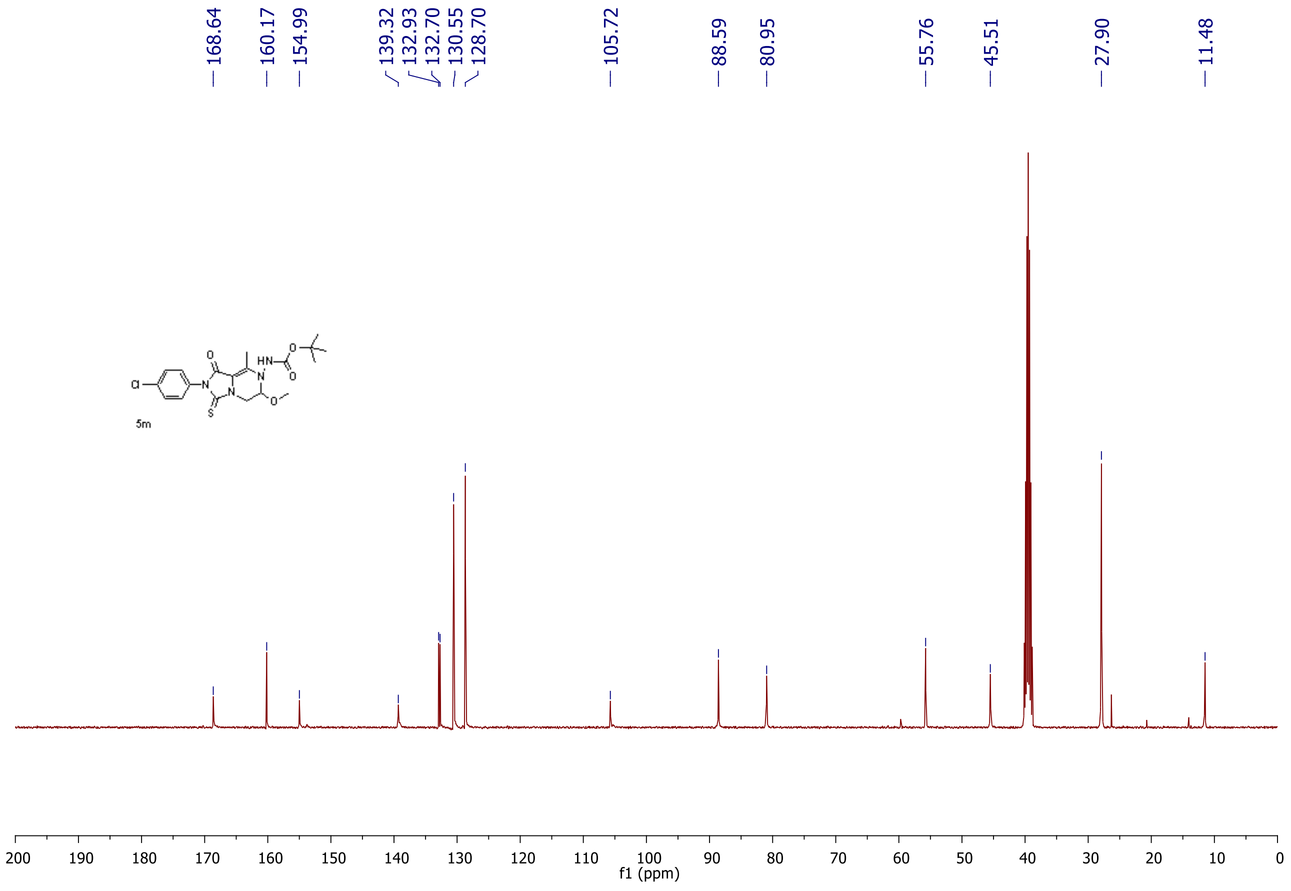
168.64
160.17
154.99
139.32
132.93
132.70
130.55
128.70
105.72
88.59
80.95
55.76
45.51
27.90
11.48
5m
200 190 180 170 160 150 140 130 120 110 100 90 80 70 60 50 40 30 20 10 0
f1 (ppm)

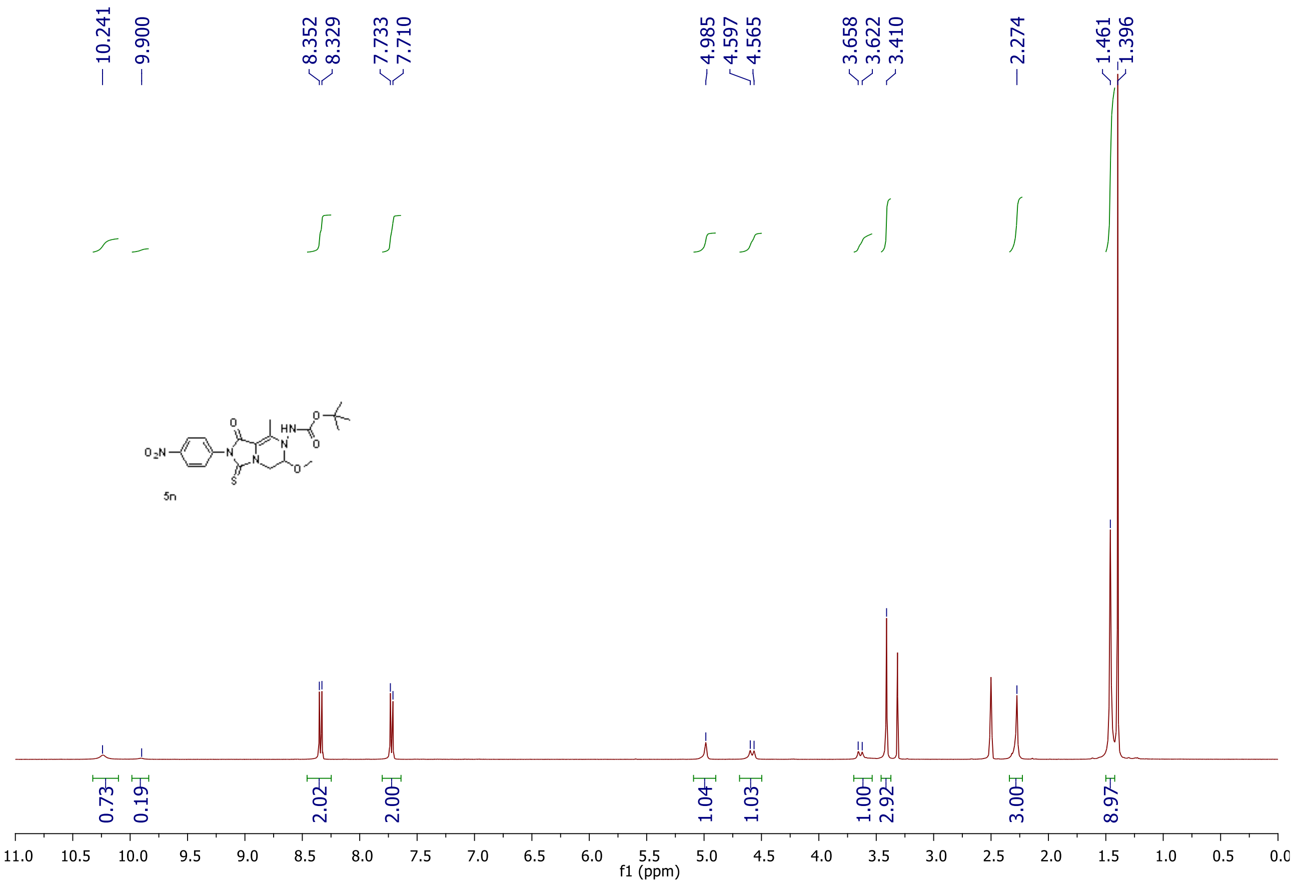
10.241
9.900
8.352
8.329
7.733
7.710
4.985
4.597
4.565
3.658
3.622
3.410
2.274
1.461
1.396
O$_2$N
HN
N
O
S
5n
0.73
0.19
2.02
2.00
1.04
1.03
1.00
2.92
3.00
8.97
f1 (ppm)

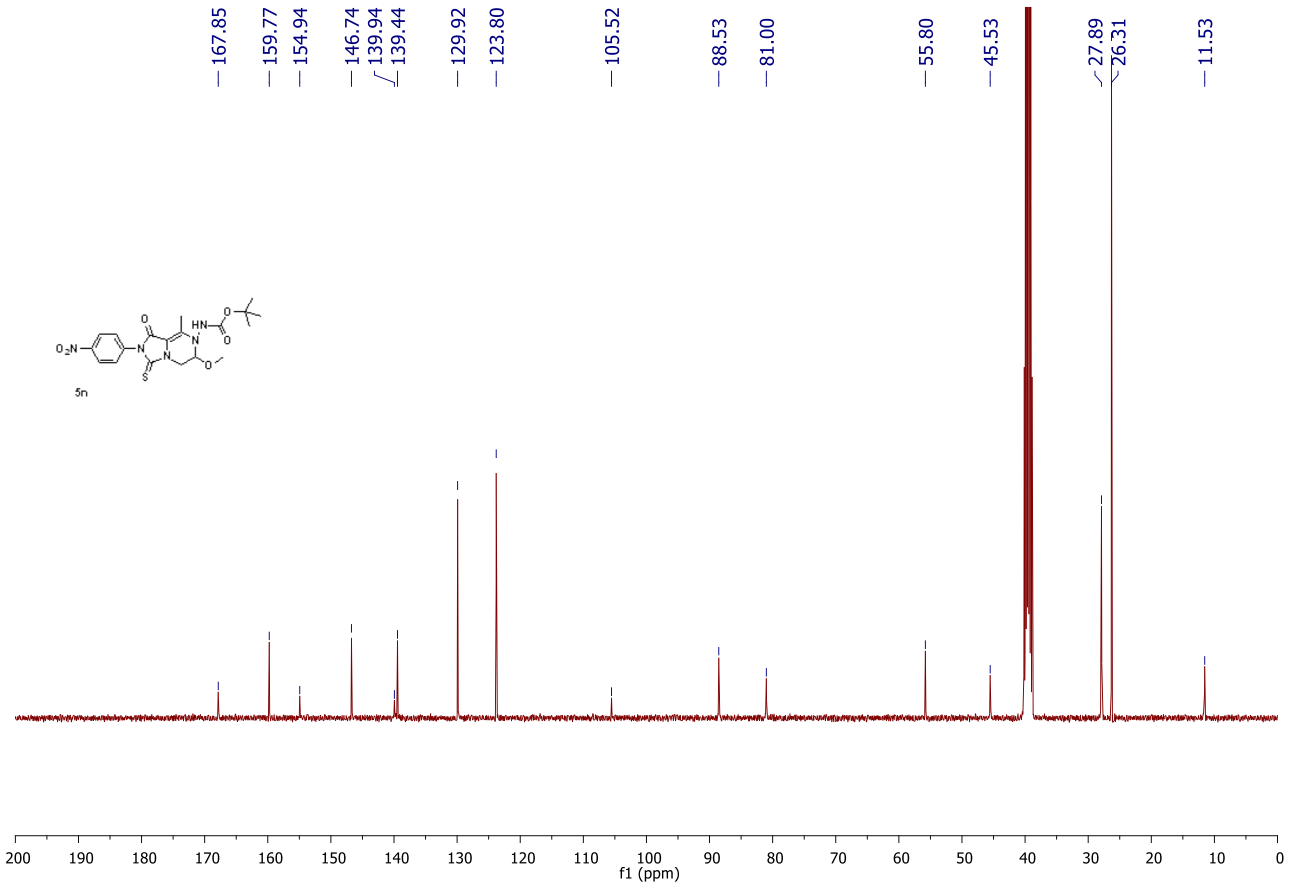
167.85
159.77
154.94
146.74
139.94
139.44
129.92
123.80
105.52
88.53
81.00
55.80
45.53
27.89
26.31
11.53
O$_2$N
HN
N
O
S
5n
f1 (ppm)

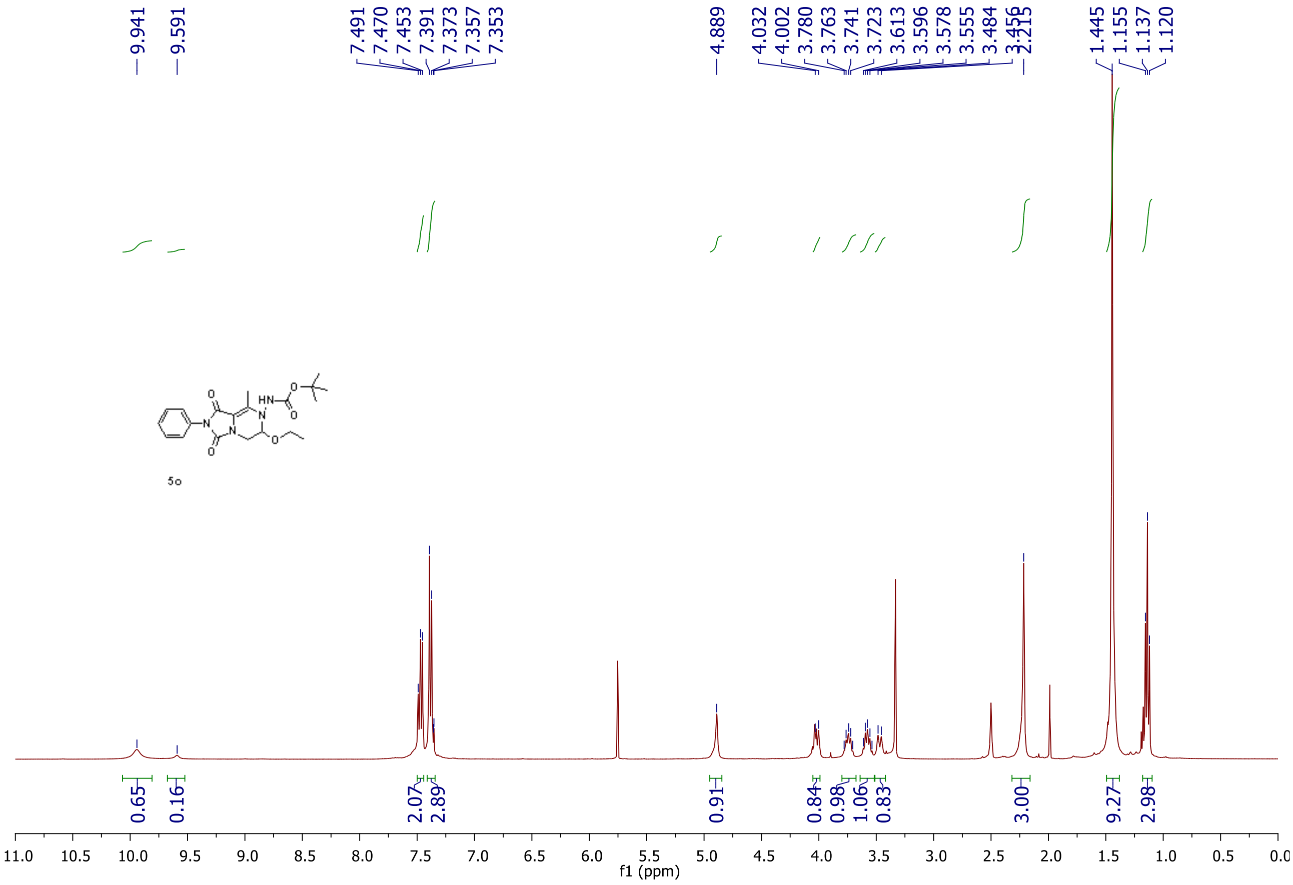
9.941
9.591
7.491
7.470
7.453
7.391
7.373
7.357
7.353
4.889
4.032
4.002
3.780
3.763
3.741
3.723
3.613
3.596
3.578
3.555
3.484
3.456
2.215
1.445
1.155
1.137
1.120
HN
O
O
N
N
N
O
O
O
5o
0.65
0.16
2.07
2.89
0.91
0.84
0.98
1.06
0.83
3.00
9.27
2.98
11.0 10.5 10.0 9.5 9.0 8.5 8.0 7.5 7.0 6.5 6.0 5.5 5.0 4.5 4.0 3.5 3.0 2.5 2.0 1.5 1.0 0.5 0.0
f1 (ppm)

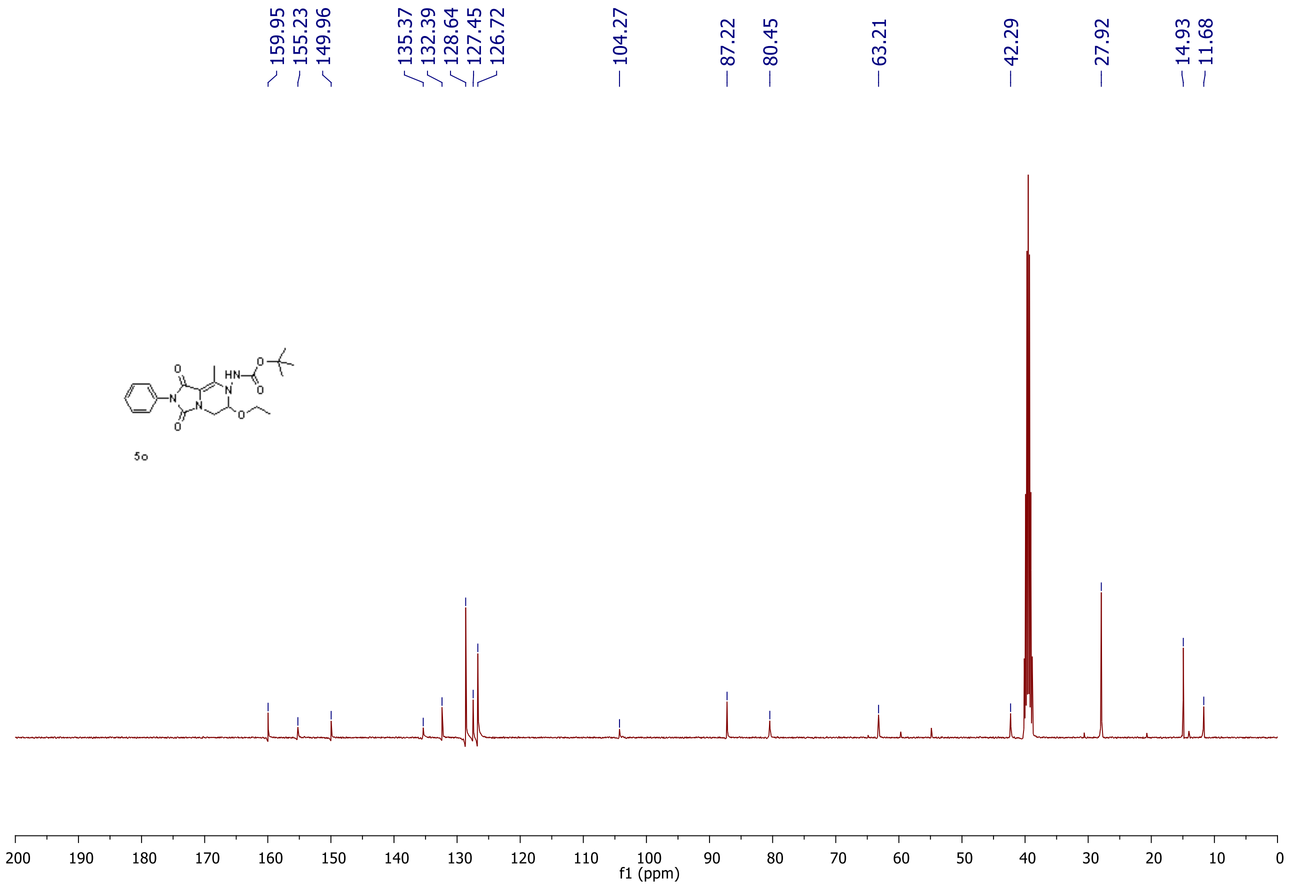
159.95
155.23
149.96
135.37
132.39
128.64
127.45
126.72
104.27
87.22
80.45
63.21
42.29
27.92
14.93
11.68
HN
O
O
N
N
N
O
O
O
5o
200 190 180 170 160 150 140 130 120 110 100 90 80 70 60 50 40 30 20 10 0
f1 (ppm)

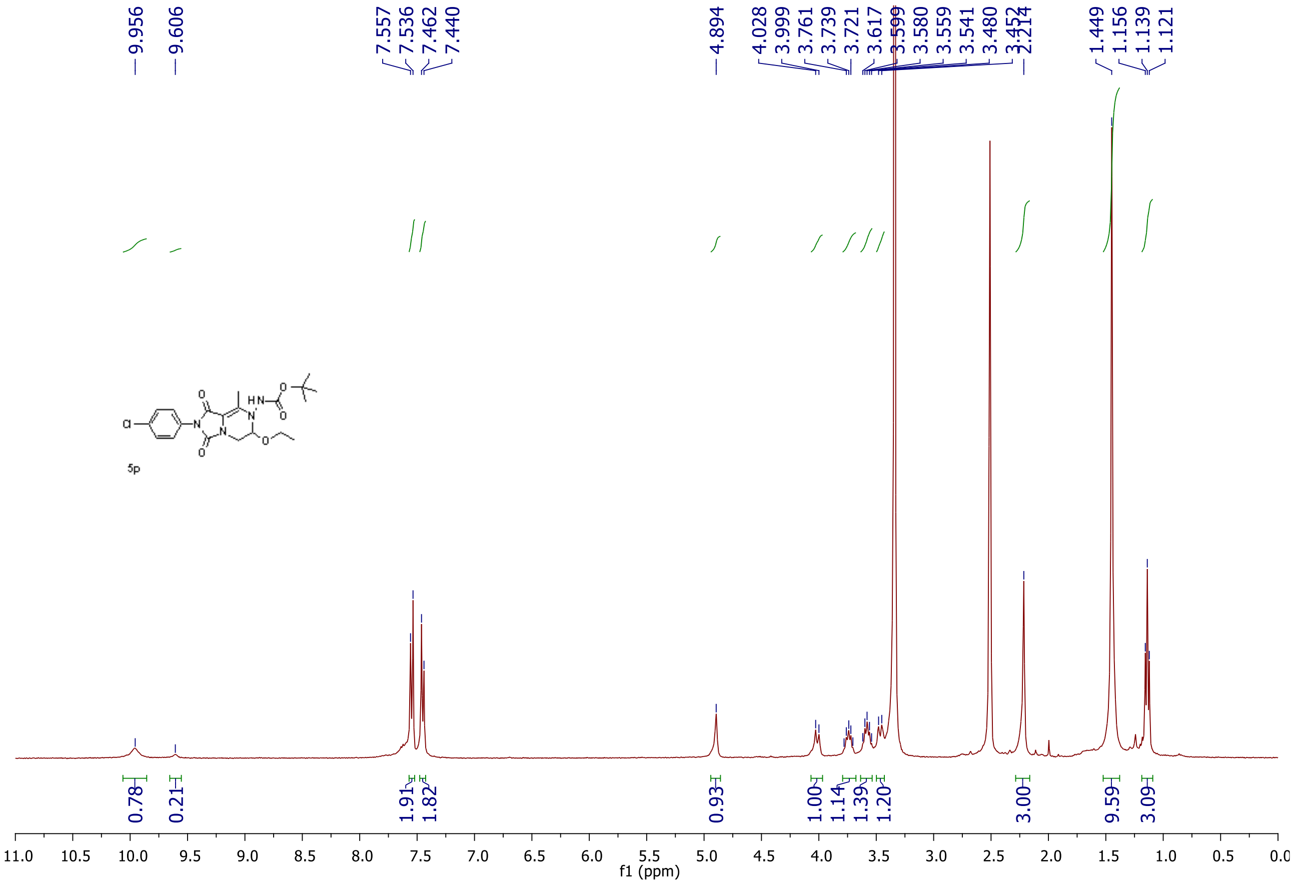
9.956
9.606
7.557
7.536
7.462
7.440
4.894
4.028
3.999
3.761
3.739
3.721
3.617
3.599
3.580
3.559
3.541
3.480
3.452
2.214
1.449
1.156
1.139
1.121
5p
0.78
0.21
1.91
1.82
0.93
1.00
1.14
1.39
1.20
3.00
9.59
3.09
f1 (ppm)

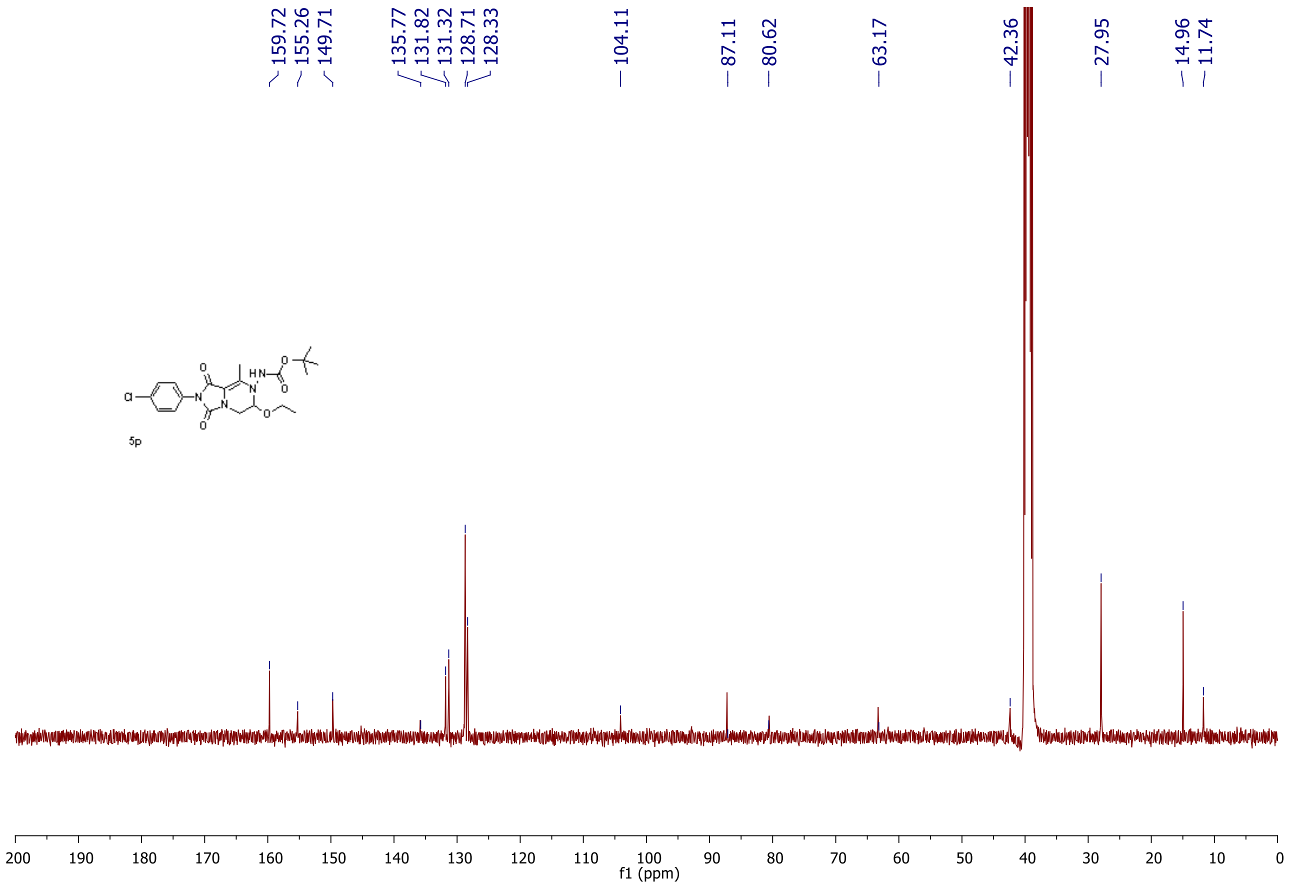
159.72
155.26
149.71
135.77
131.82
131.32
128.71
128.33
104.11
87.11
80.62
63.17
42.36
27.95
14.96
11.74
5p
f1 (ppm)

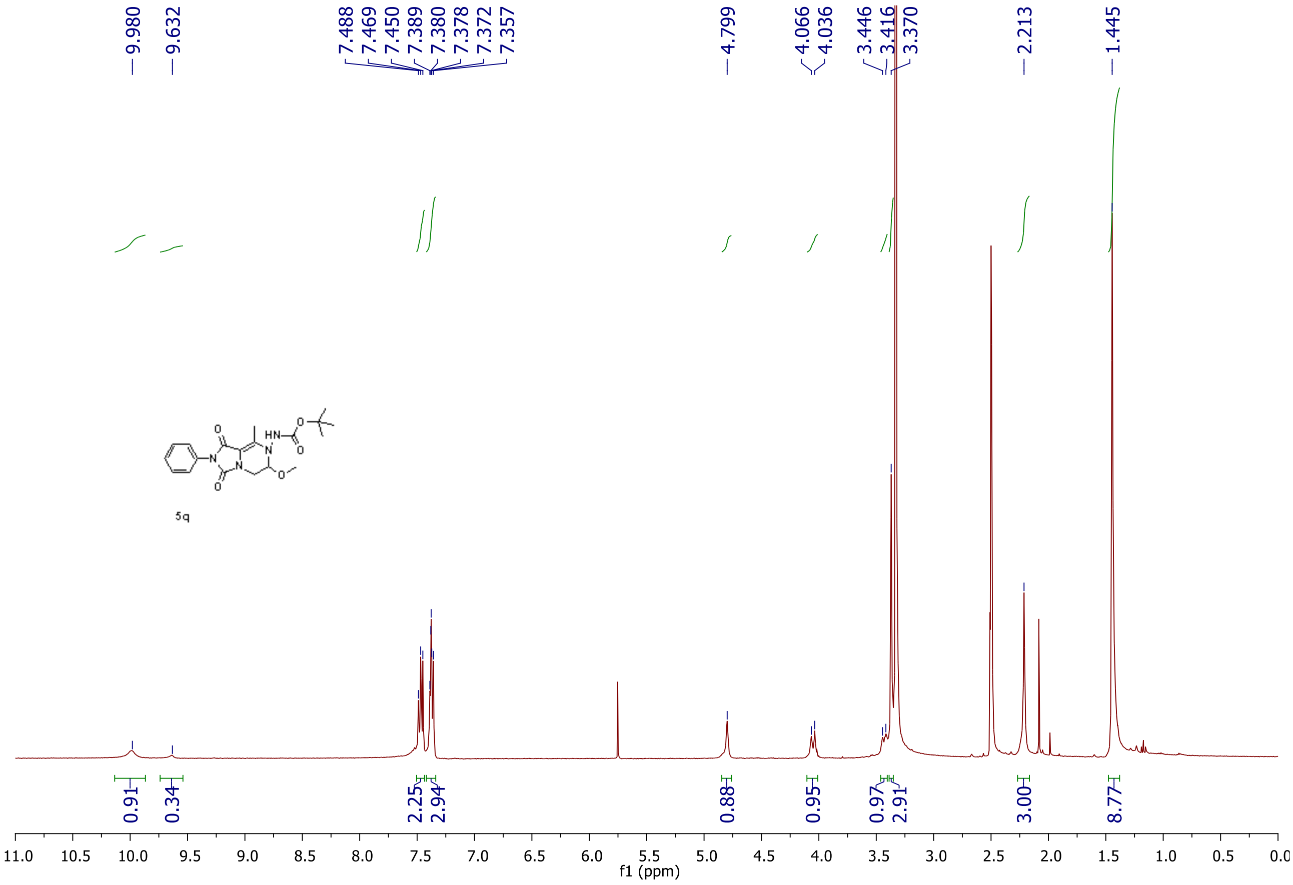

9.980
9.632
7.488
7.469
7.450
7.389
7.380
7.378
7.372
7.357
4.799
4.066
4.036
3.446
3.416
3.370
2.213
1.445
0.91
0.34
2.25
2.94
0.88
0.95
0.97
2.91
3.00
8.77
f1 (ppm)

5q

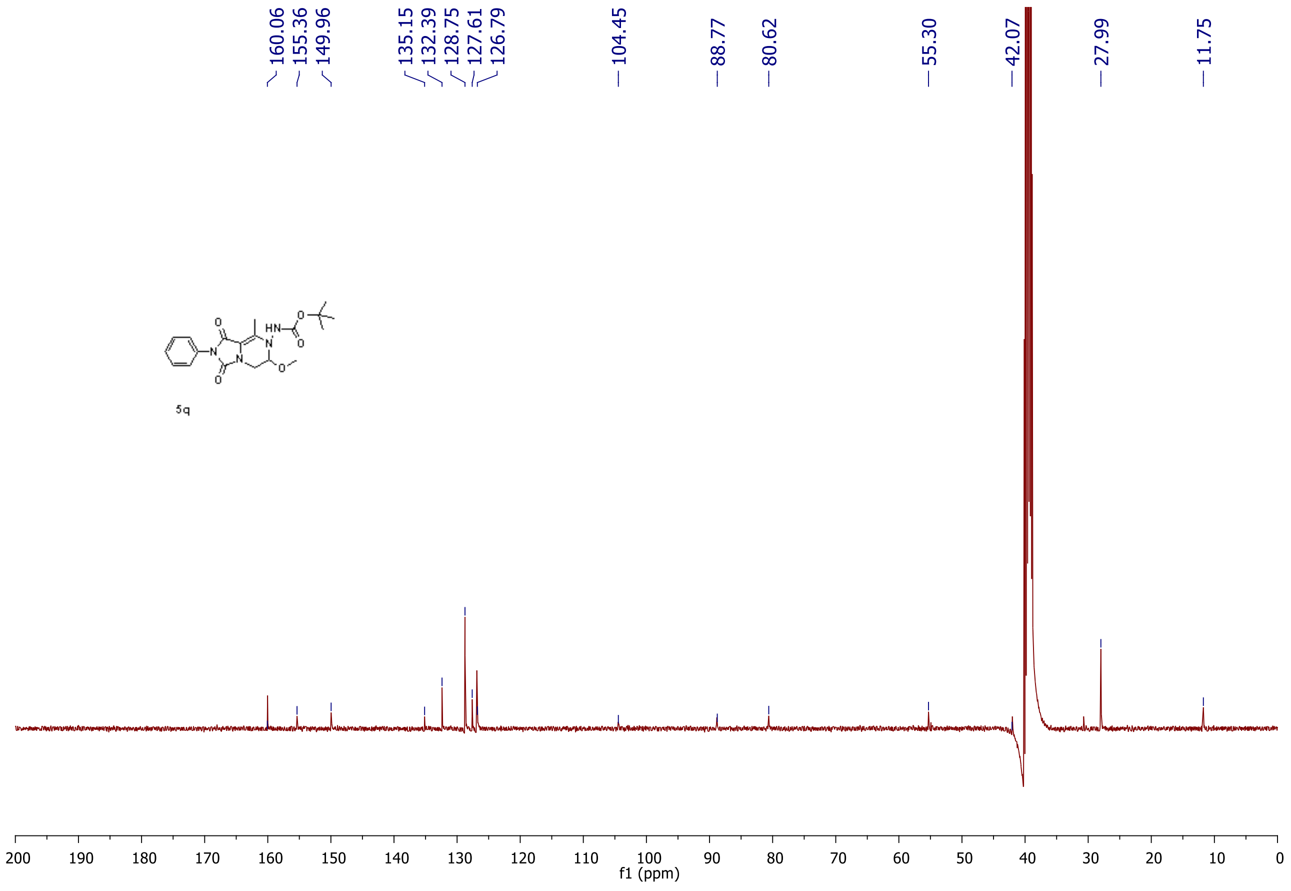

160.06
155.36
149.96
135.15
132.39
128.75
127.61
126.79
104.45
88.77
80.62
55.30
42.07
27.99
11.75
f1 (ppm)

5q

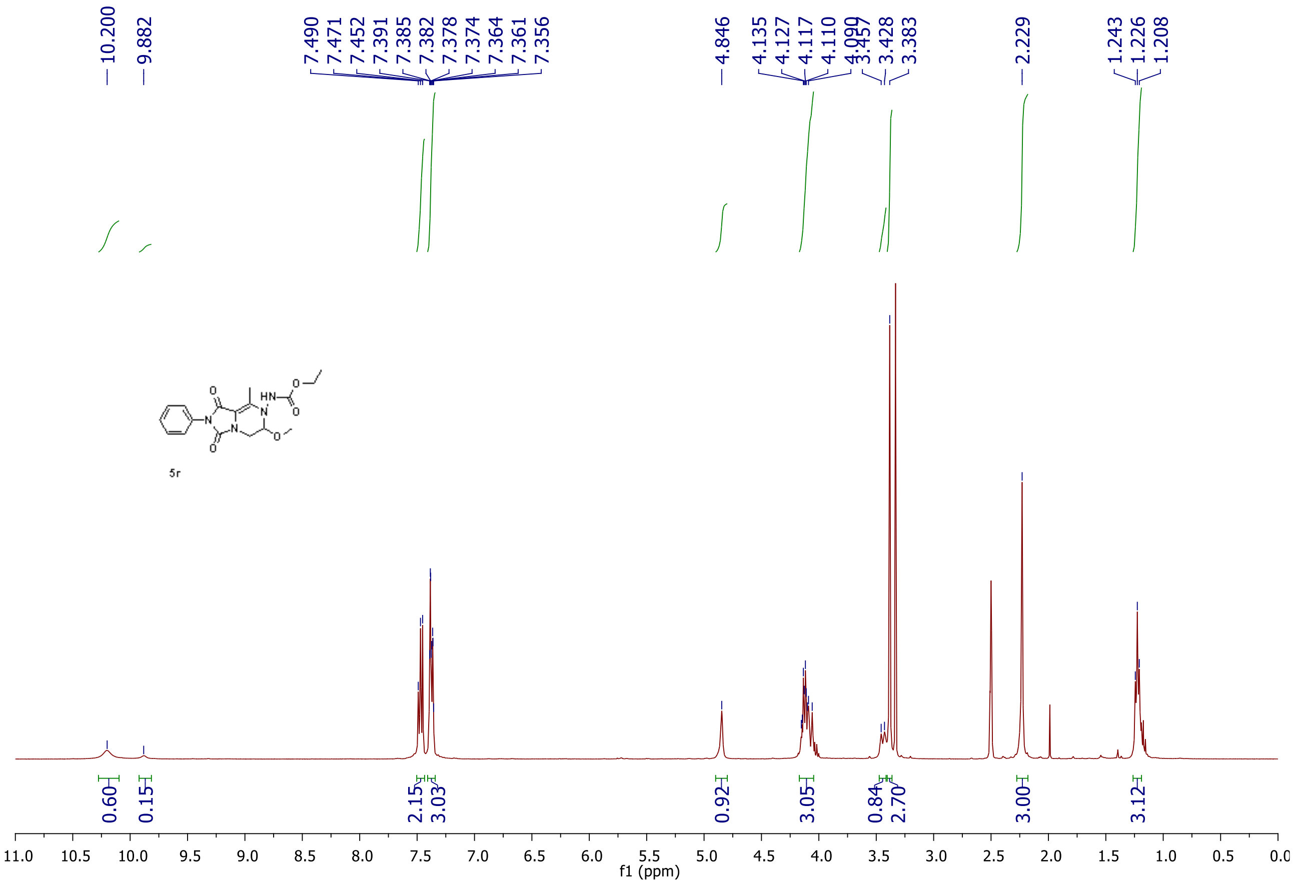

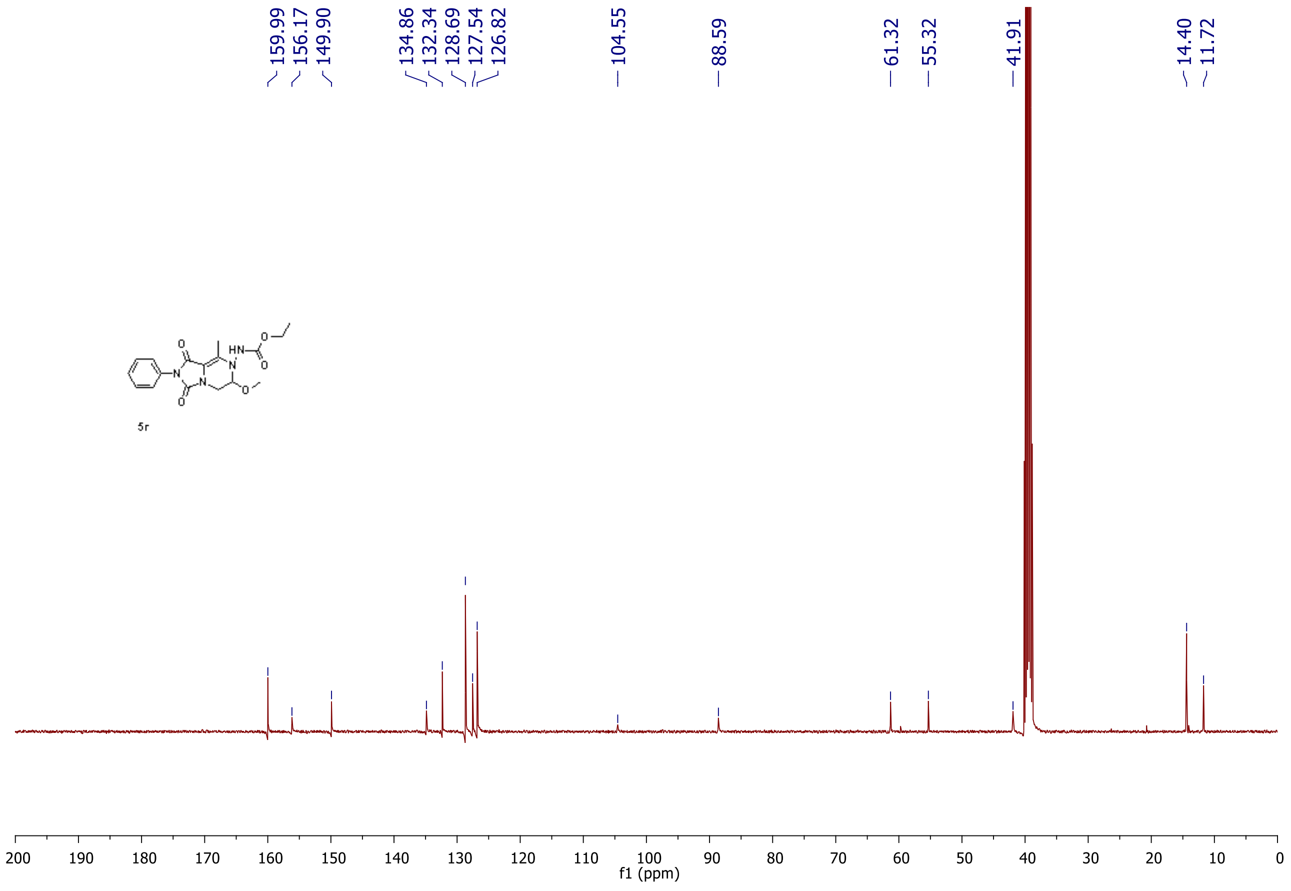

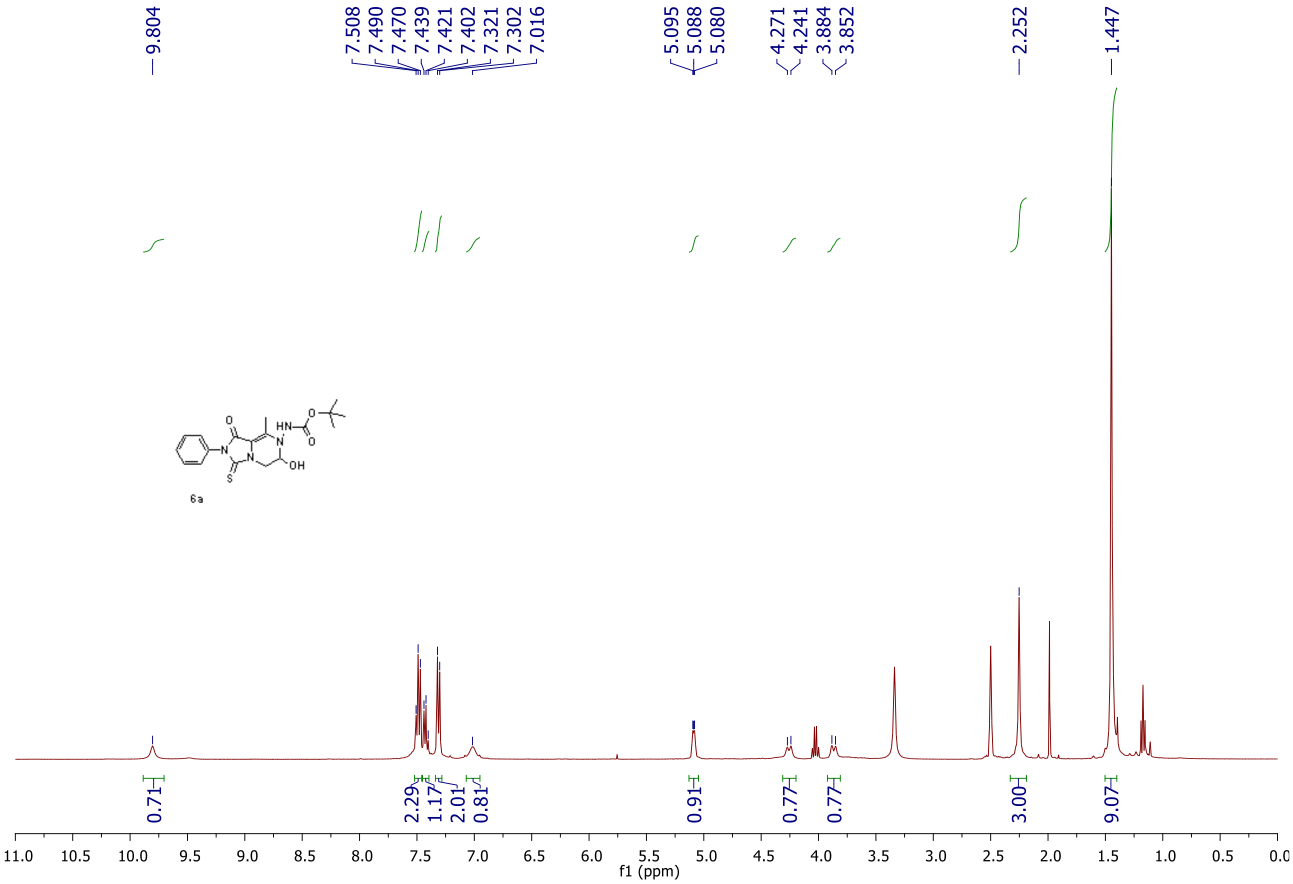
9.804
7.508
7.490
7.470
7.439
7.421
7.402
7.321
7.302
7.016
5.095
5.088
5.080
4.271
4.241
3.884
3.852
2.252
1.447
0.71
2.29
1.17
2.01
0.81
0.91
0.77
0.77
3.00
9.07
6a
11.0 10.5 10.0 9.5 9.0 8.5 8.0 7.5 7.0 6.5 6.0 5.5 5.0 4.5 4.0 3.5 3.0 2.5 2.0 1.5 1.0 0.5 0.0
f1 (ppm)

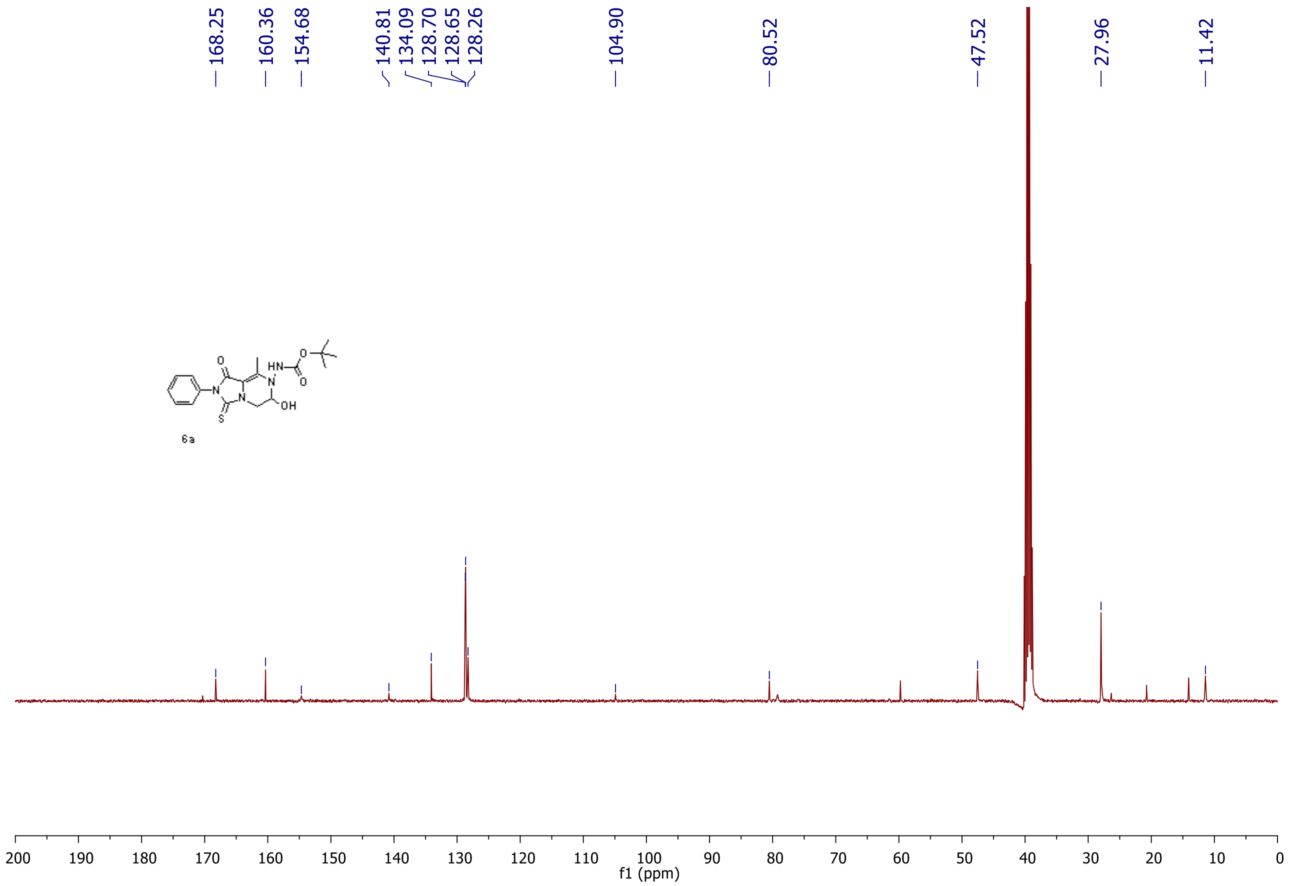
168.25
160.36
154.68
140.81
134.09
128.70
128.65
128.26
104.90
80.52
47.52
27.96
11.42
6a
200 190 180 170 160 150 140 130 120 110 100 90 80 70 60 50 40 30 20 10 0
f1 (ppm)

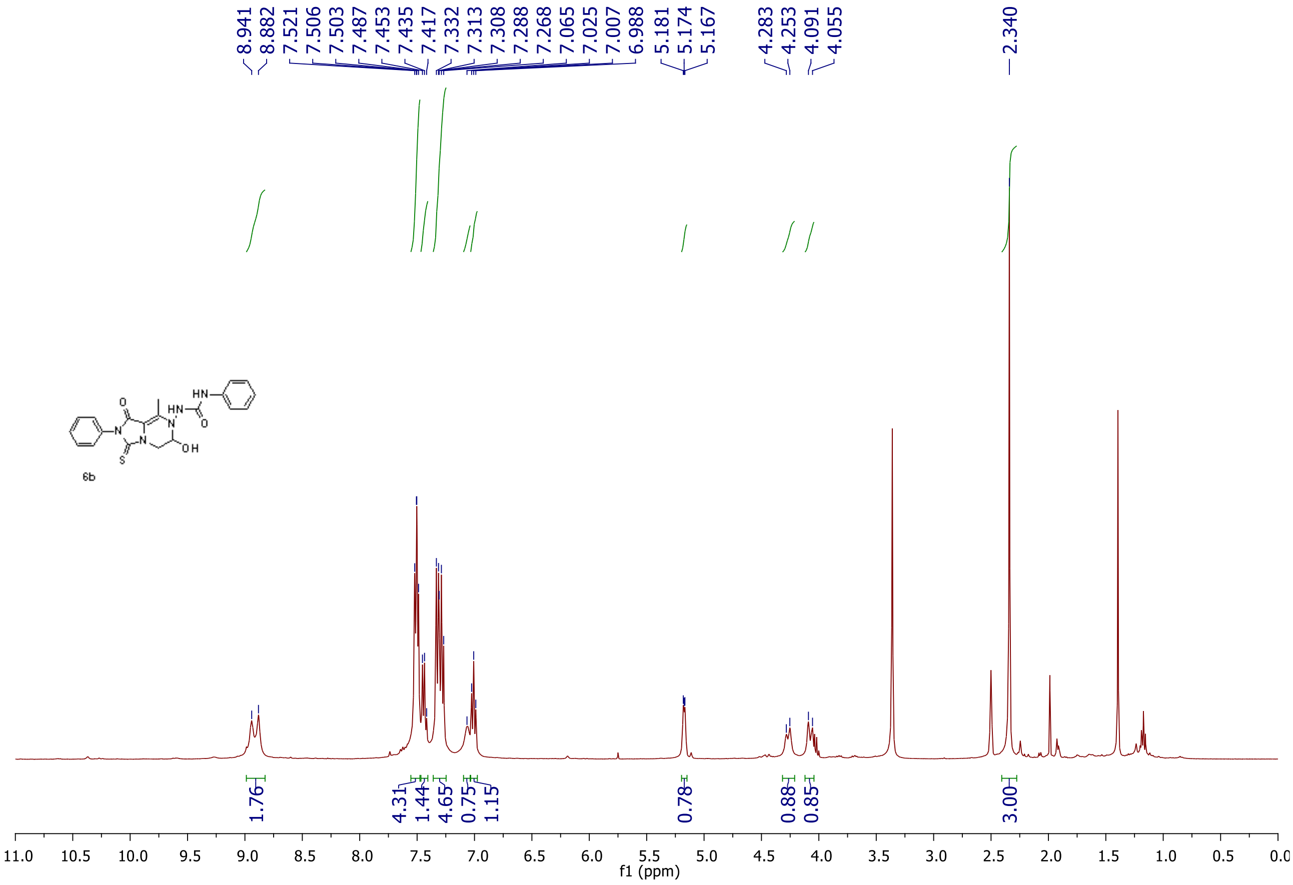
8.941
8.882
7.521
7.506
7.503
7.487
7.453
7.435
7.417
7.332
7.313
7.308
7.288
7.268
7.065
7.025
7.007
6.988
5.181
5.174
5.167
4.283
4.253
4.091
4.055
2.340
6b
1.76
4.31
1.44
4.65
0.75
1.15
0.78
0.88
0.85
3.00
f1 (ppm)

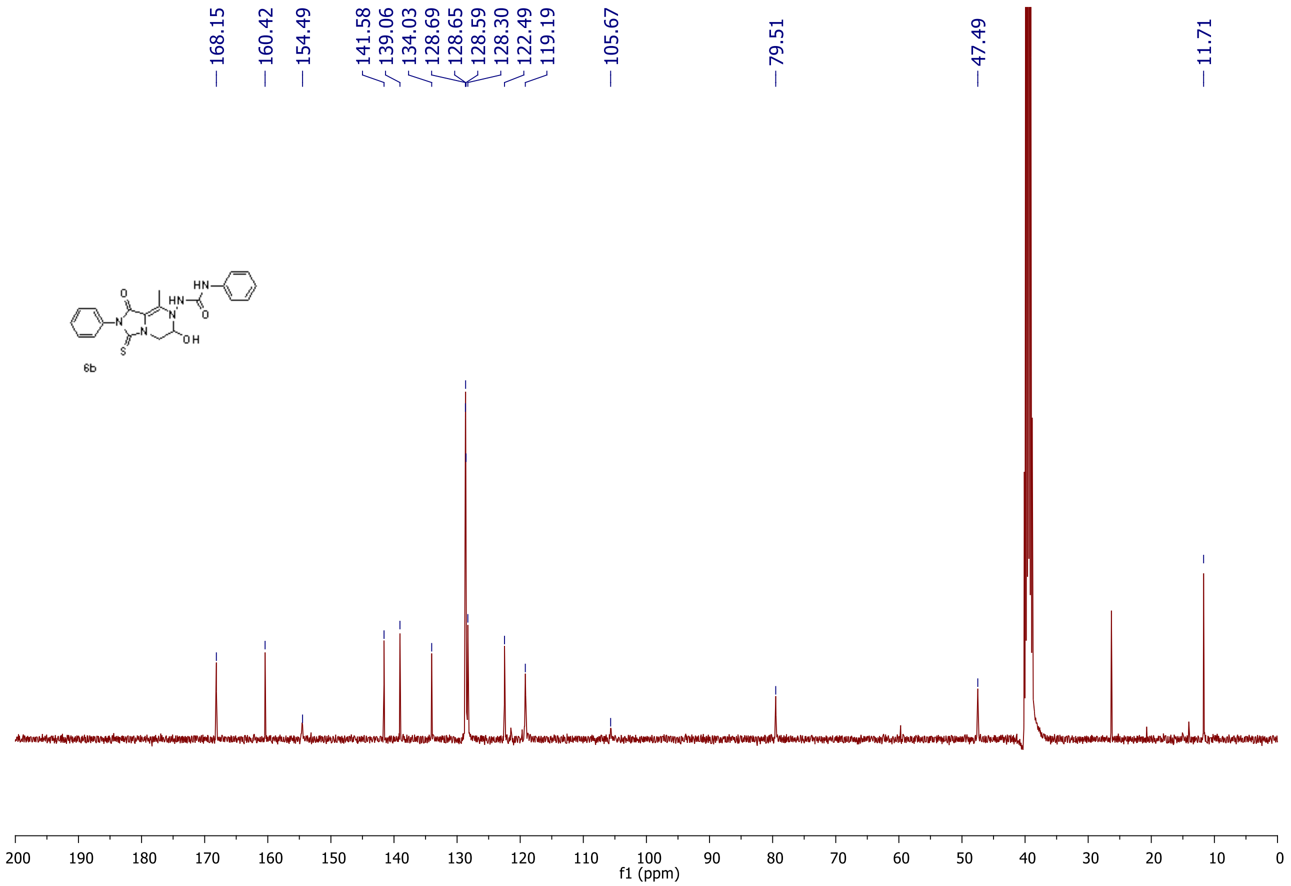
168.15
160.42
154.49
141.58
139.06
134.03
128.69
128.65
128.59
128.30
122.49
119.19
105.67
79.51
47.49
11.71
6b
f1 (ppm)

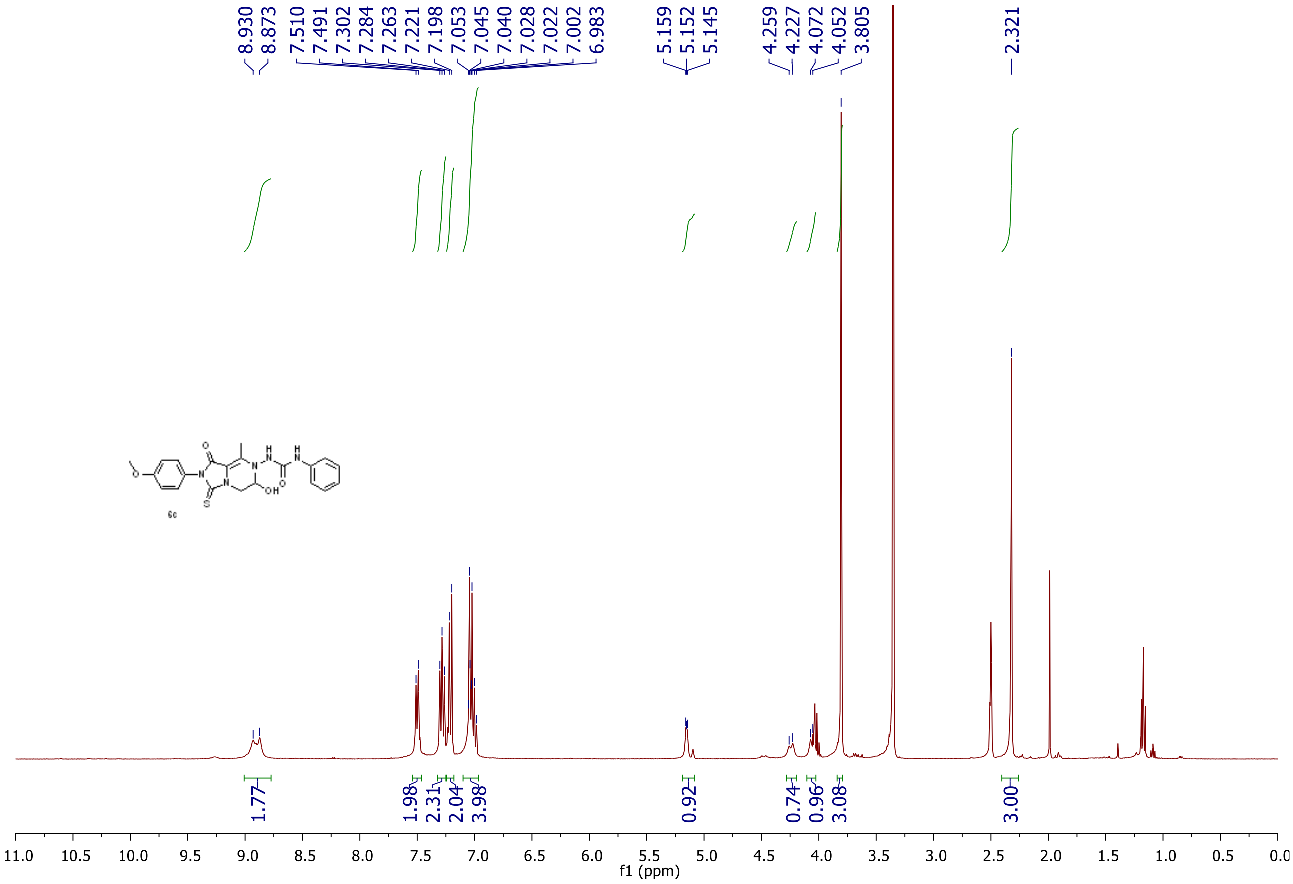
8.930
8.873
7.510
7.491
7.302
7.284
7.263
7.221
7.198
7.053
7.045
7.040
7.028
7.022
7.002
6.983
5.159
5.152
5.145
4.259
4.227
4.072
4.052
3.805
2.321
1.77
1.98
2.31
2.04
3.98
0.92
0.74
0.96
3.08
3.00
6c
f1 (ppm)

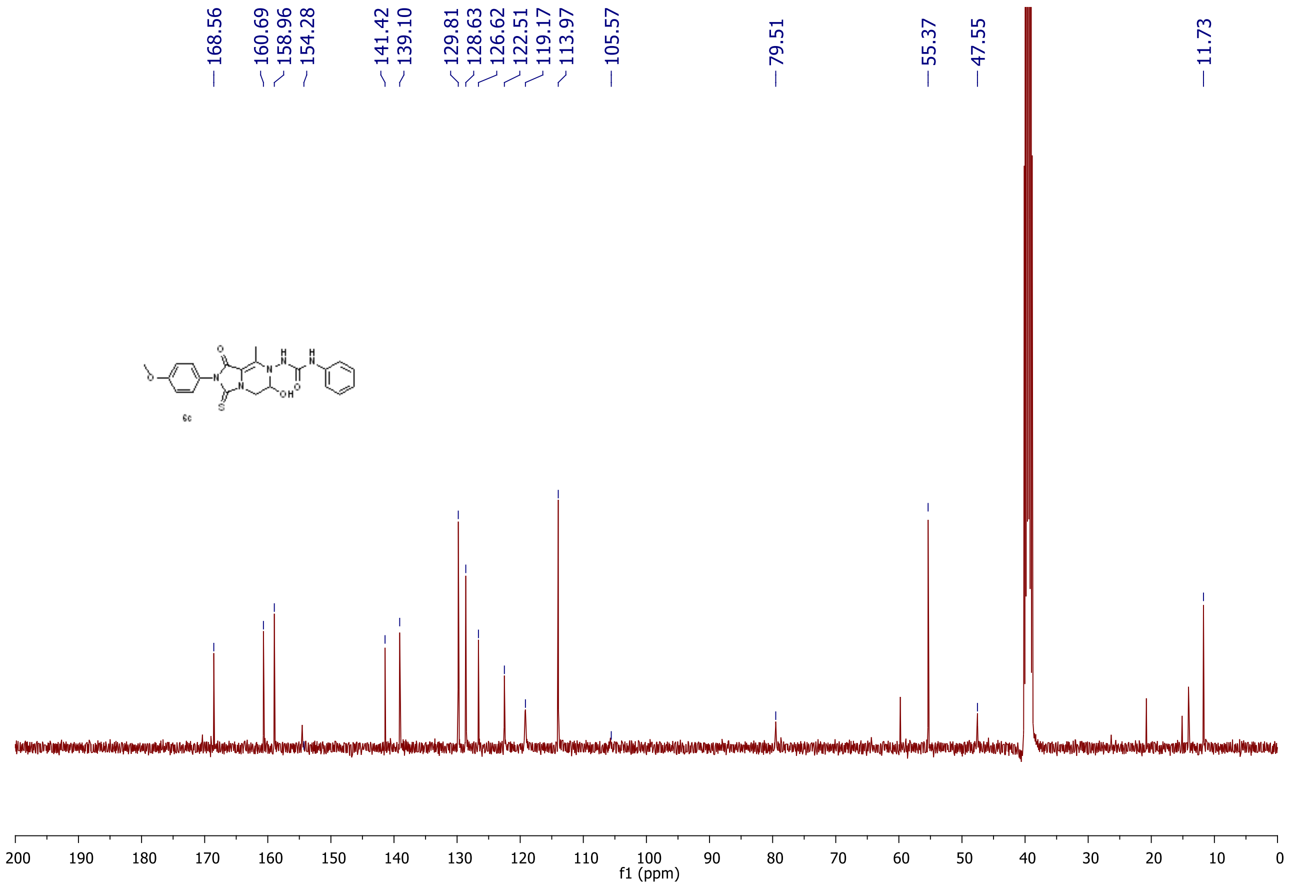
168.56
160.69
158.96
154.28
141.42
139.10
129.81
128.63
126.62
122.51
119.17
113.97
105.57
79.51
55.37
47.55
11.73
6c
f1 (ppm)

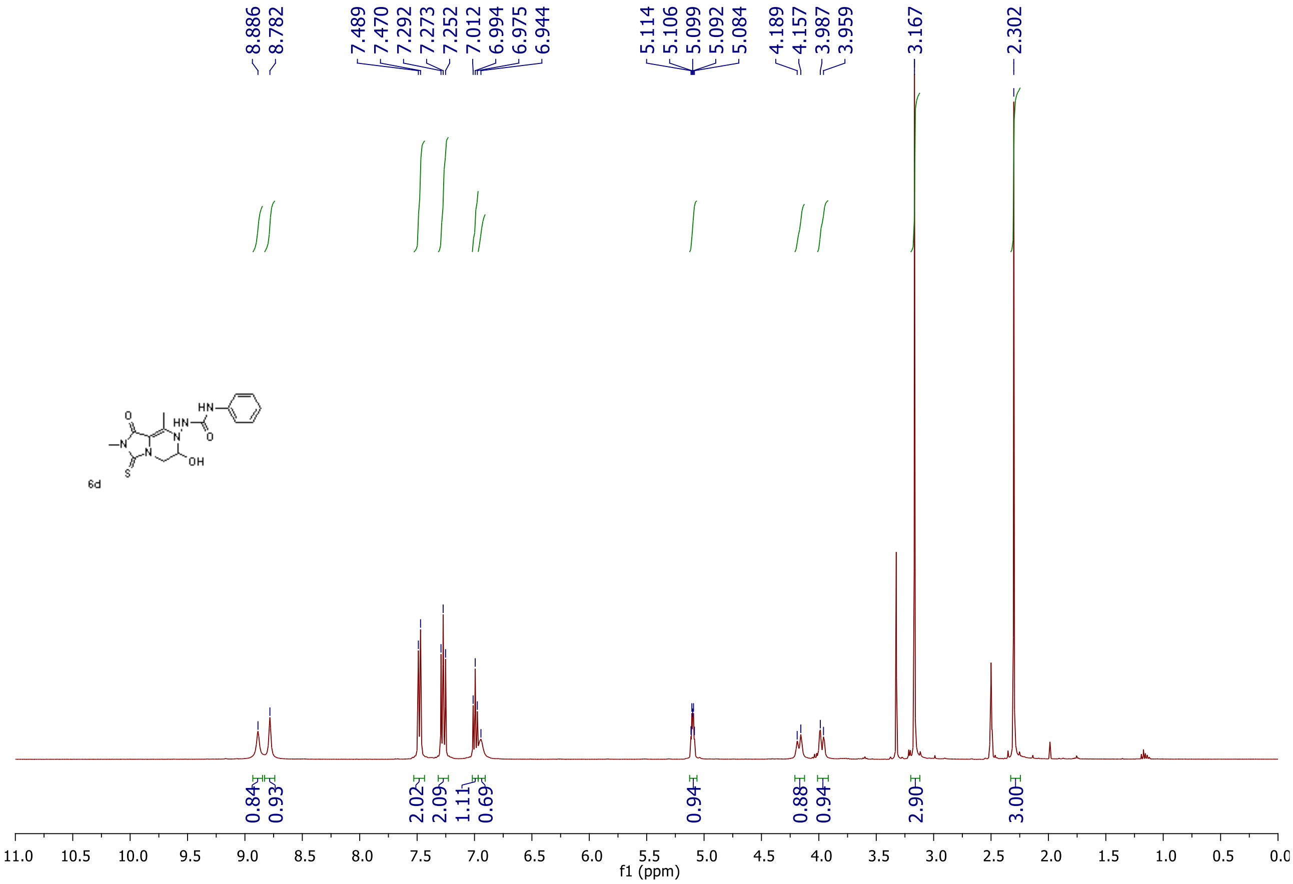
8.886
8.782
7.489
7.470
7.292
7.273
7.252
7.012
6.994
6.975
6.944
5.114
5.106
5.099
5.092
5.084
4.189
4.157
3.987
3.959
3.167
2.302
HN
HN
O
N
O
N
N
OH
S
6d
0.84
0.93
2.02
2.09
1.11
0.69
0.94
0.88
0.94
2.90
3.00
11.0 10.5 10.0 9.5 9.0 8.5 8.0 7.5 7.0 6.5 6.0 5.5 5.0 4.5 4.0 3.5 3.0 2.5 2.0 1.5 1.0 0.5 0.0
f1 (ppm)

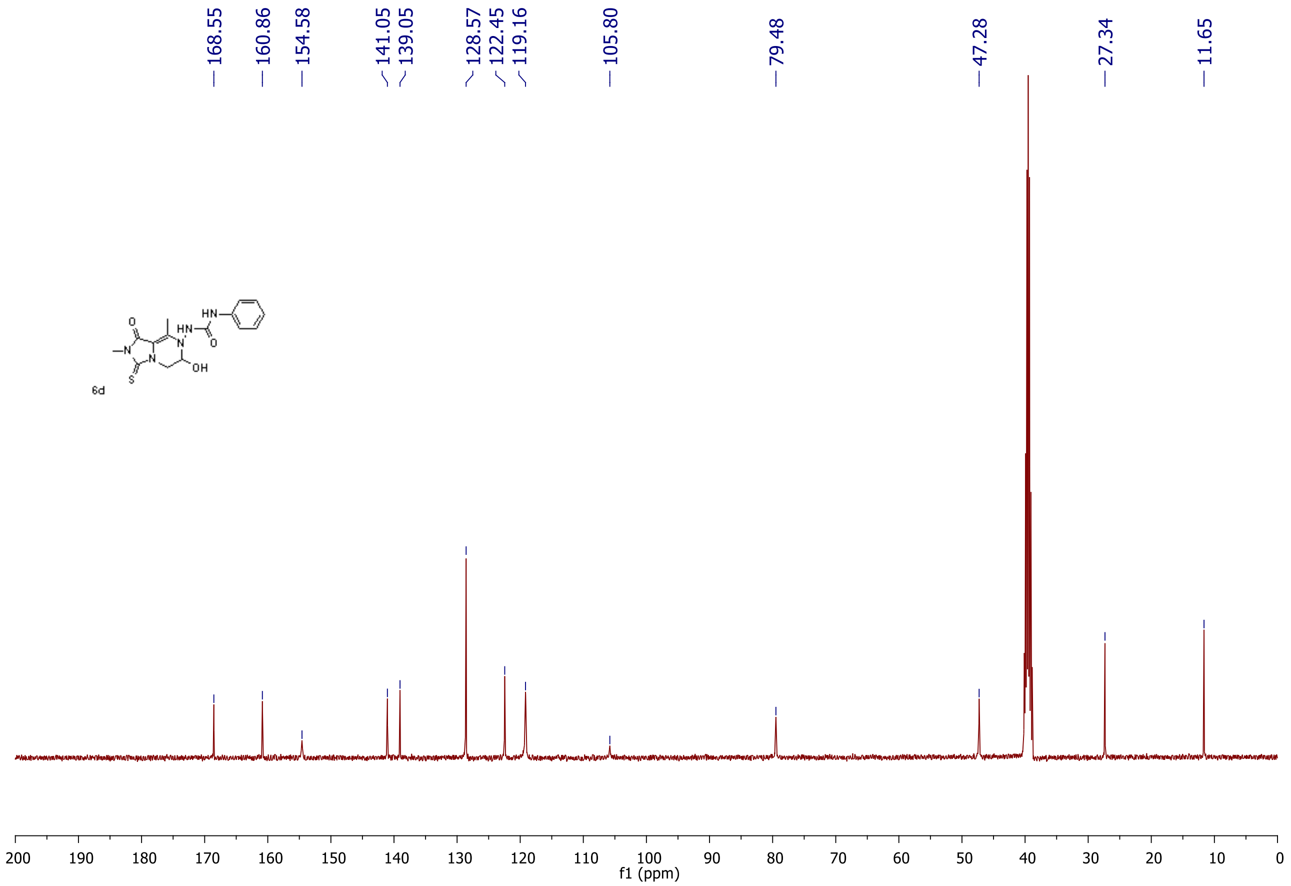
168.55
160.86
154.58
141.05
139.05
128.57
122.45
119.16
105.80
79.48
47.28
27.34
11.65
HN
HN
O
N
O
N
N
OH
S
6d
200 190 180 170 160 150 140 130 120 110 100 90 80 70 60 50 40 30 20 10 0
f1 (ppm)

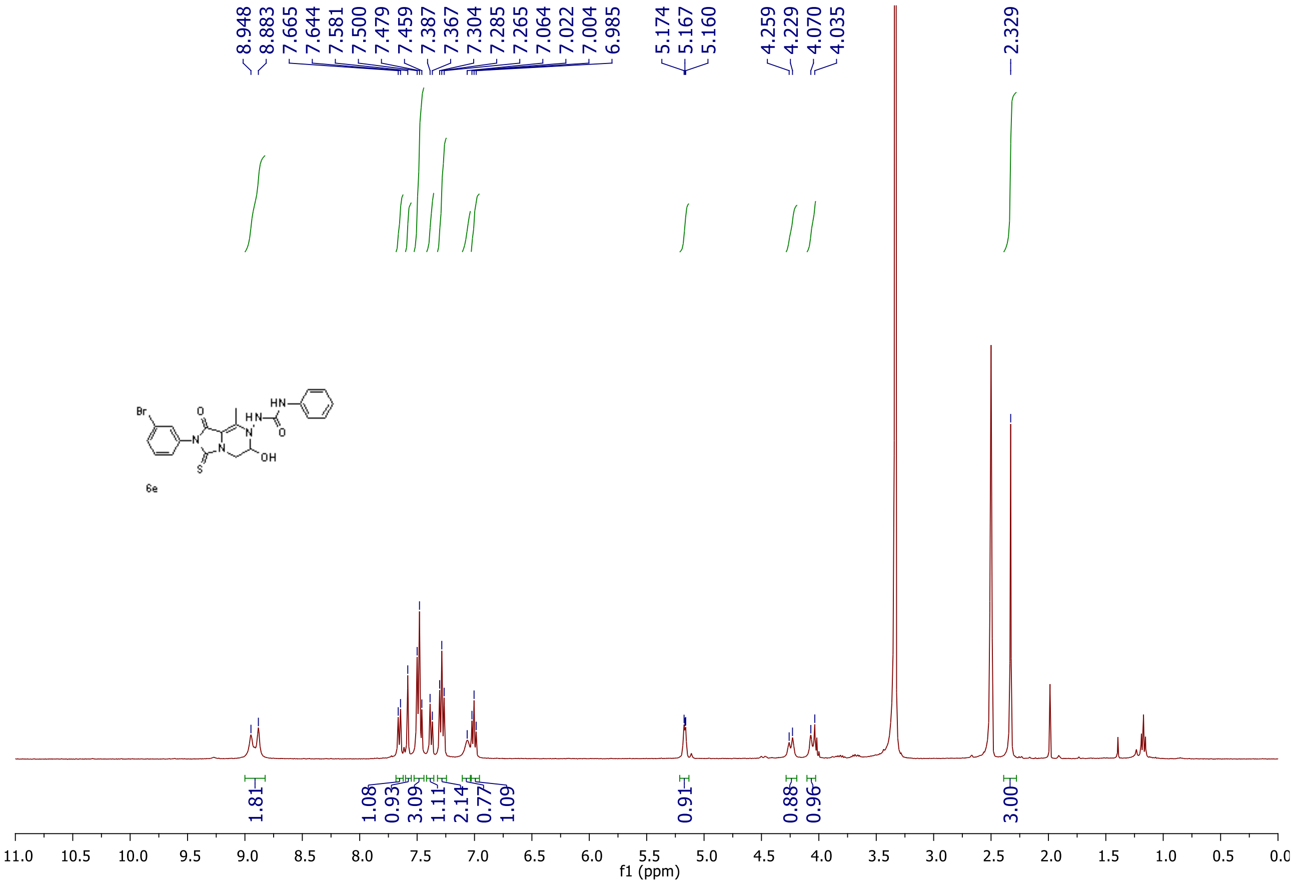
8.948
8.883
7.665
7.644
7.581
7.500
7.479
7.459
7.387
7.367
7.304
7.285
7.265
7.064
7.022
7.004
6.985
5.174
5.167
5.160
4.259
4.229
4.070
4.035
2.329
6e
1.81
1.08
0.93
3.09
1.11
2.14
0.77
1.09
0.91
0.88
0.96
3.00
f1 (ppm)

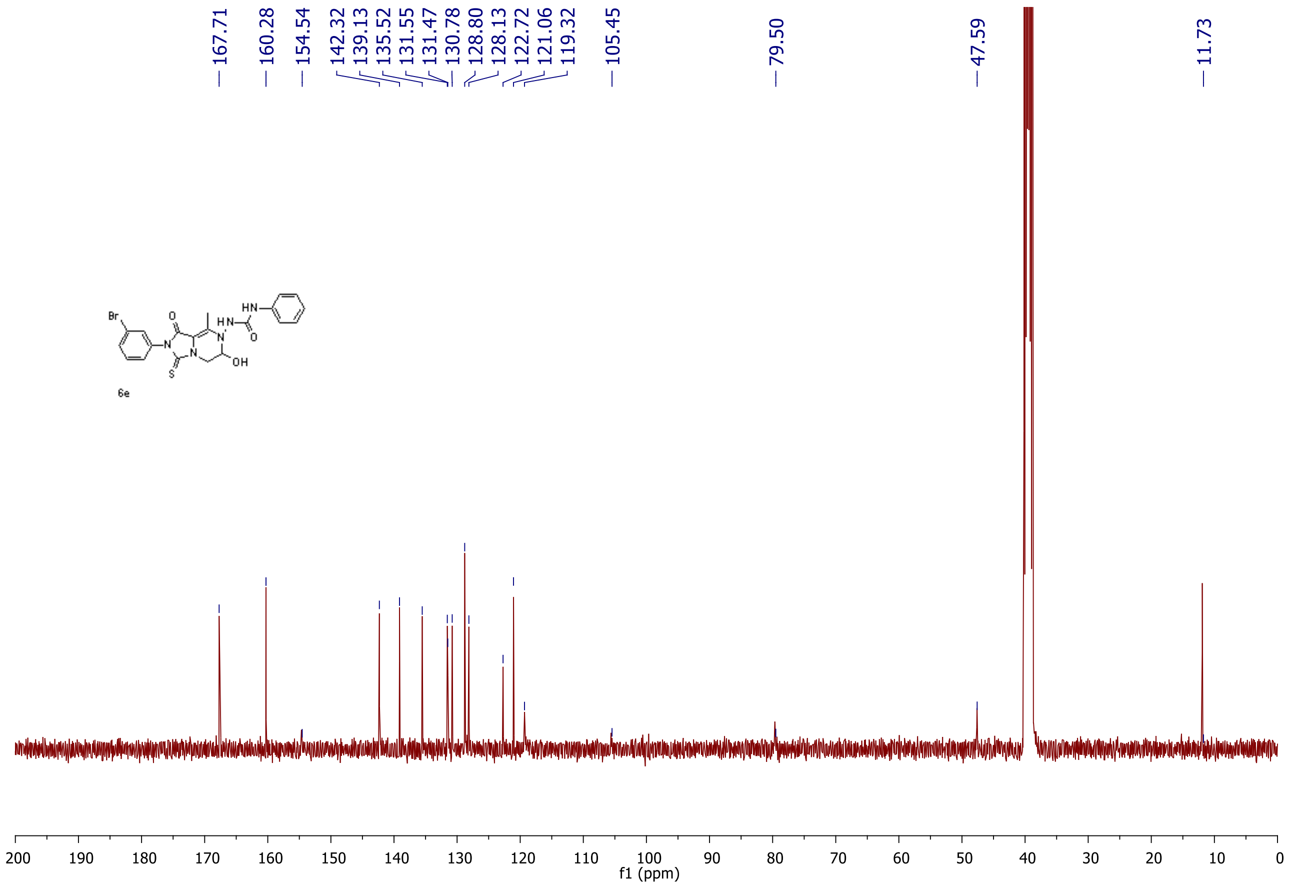
167.71
160.28
154.54
142.32
139.13
135.52
131.55
131.47
130.78
128.80
128.13
122.72
121.06
119.32
105.45
79.50
47.59
11.73
6e
f1 (ppm)

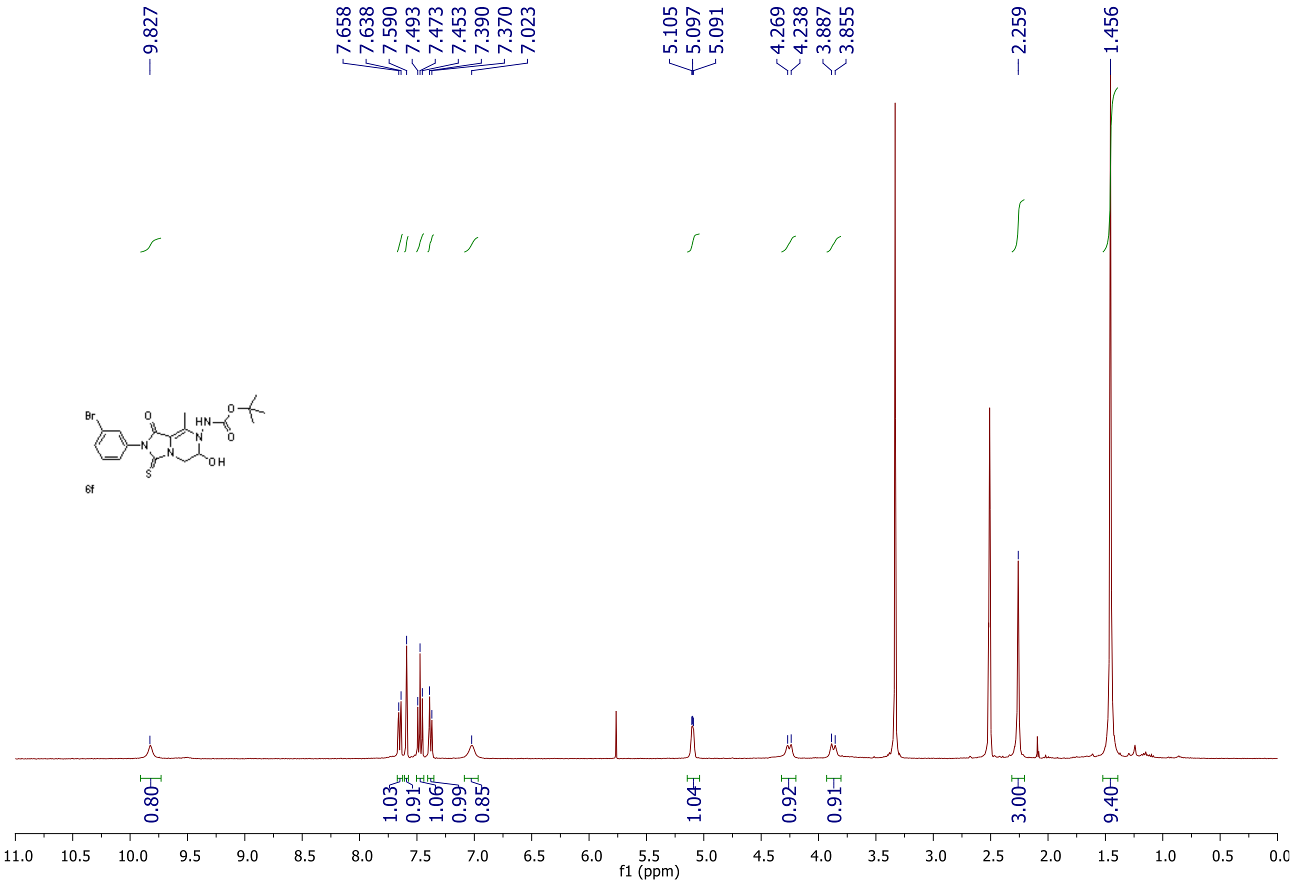
9.827
7.658 7.638 7.590 7.493 7.473 7.453 7.390 7.370 7.023
5.105 5.097 5.091
4.269 4.238 3.887 3.855
2.259
1.456
0.80
1.03 0.91 1.06 0.99 0.85
1.04
0.92
0.91
3.00
9.40
Br
HN
O
N
OH
S
6f
f1 (ppm)

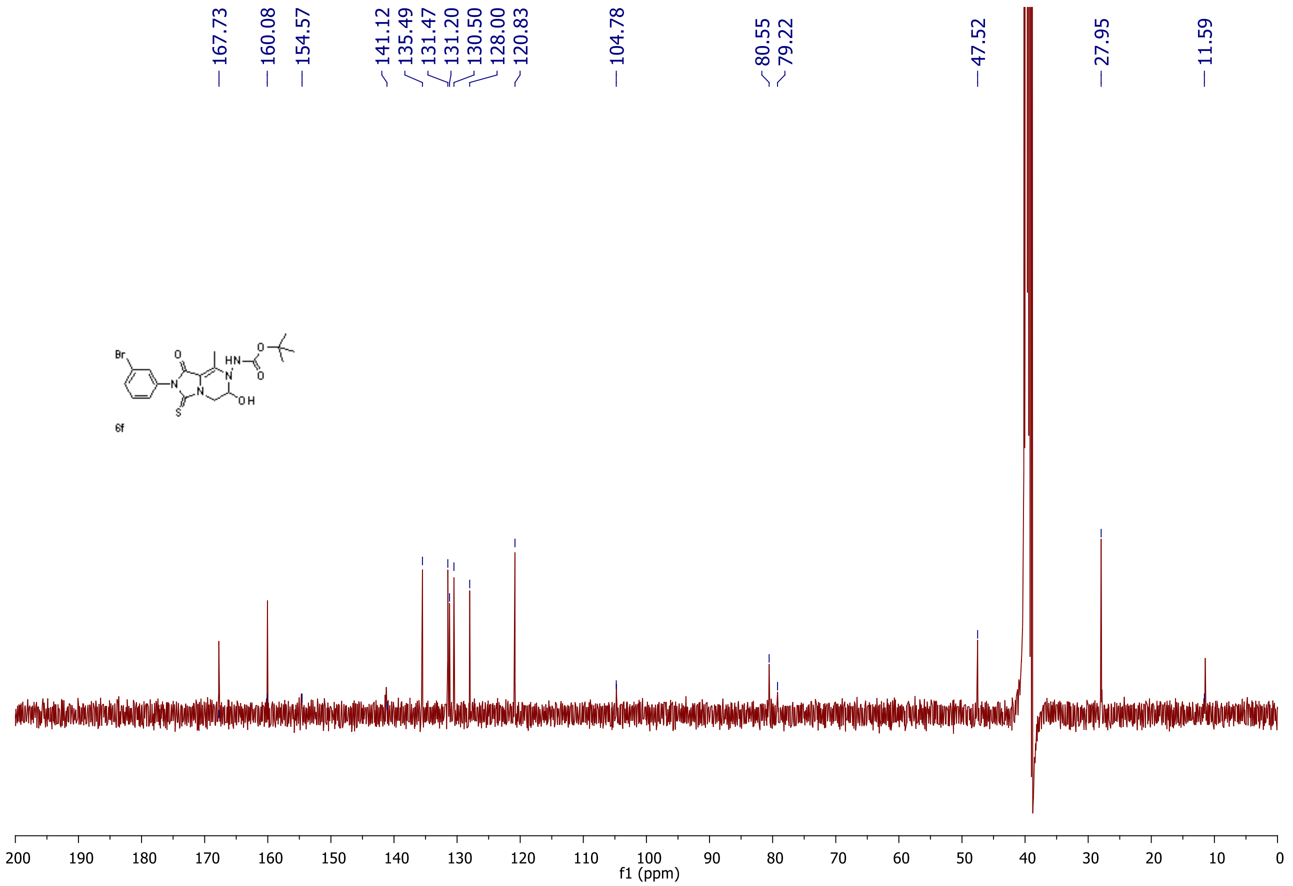
167.73
160.08
154.57
141.12 135.49 131.47 131.20 130.50 128.00 120.83
104.78
80.55 79.22
47.52
27.95
11.59
Br
HN
O
N
OH
S
6f
f1 (ppm)

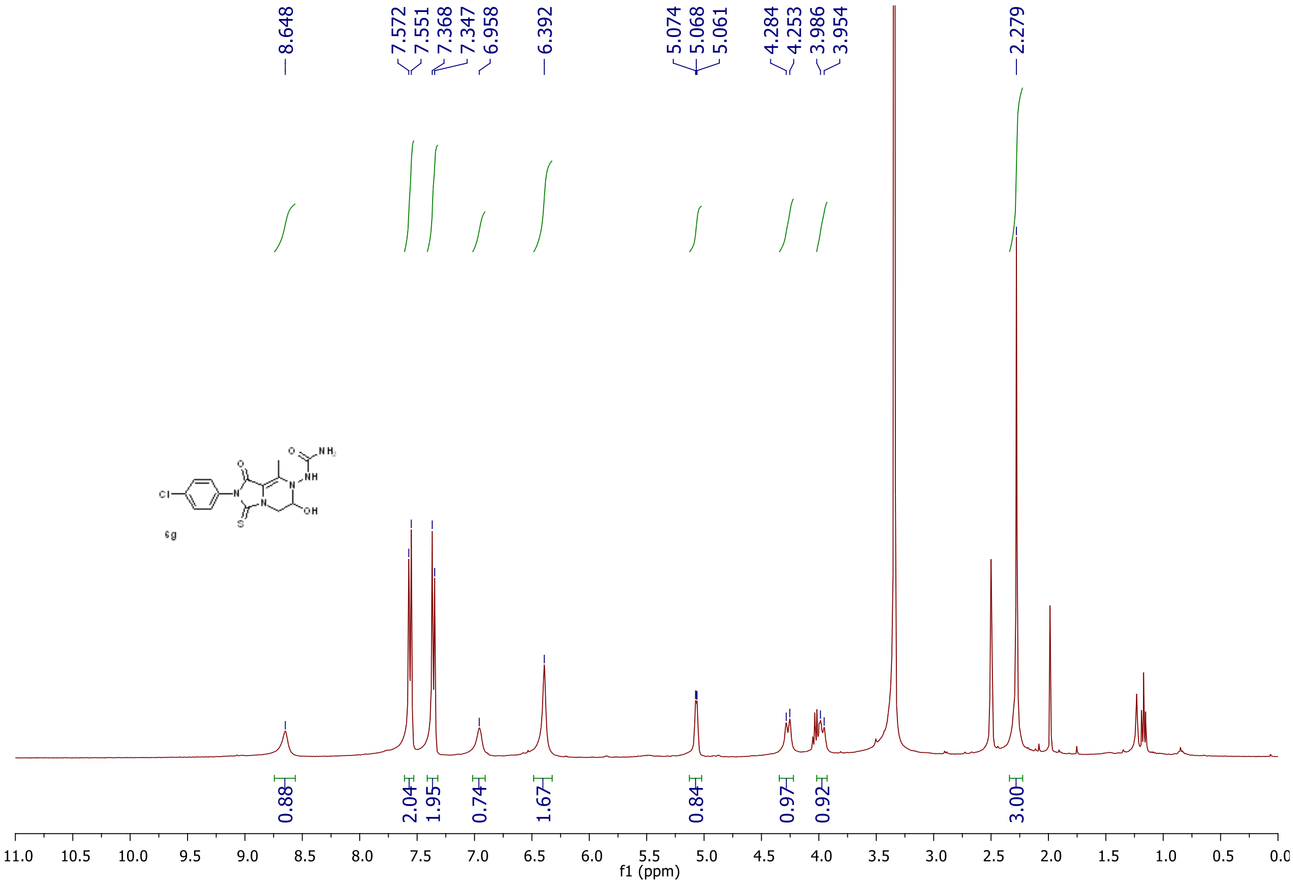
8.648
7.572
7.551
7.368
7.347
6.958
6.392
5.074
5.068
5.061
4.284
4.253
3.986
3.954
2.279
Cl
O
N
NH₂
NH
OH
S
6g
0.88
2.04
1.95
0.74
1.67
0.84
0.97
0.92
3.00
11.0 10.5 10.0 9.5 9.0 8.5 8.0 7.5 7.0 6.5 6.0 5.5 5.0 4.5 4.0 3.5 3.0 2.5 2.0 1.5 1.0 0.5 0.0
f1 (ppm)

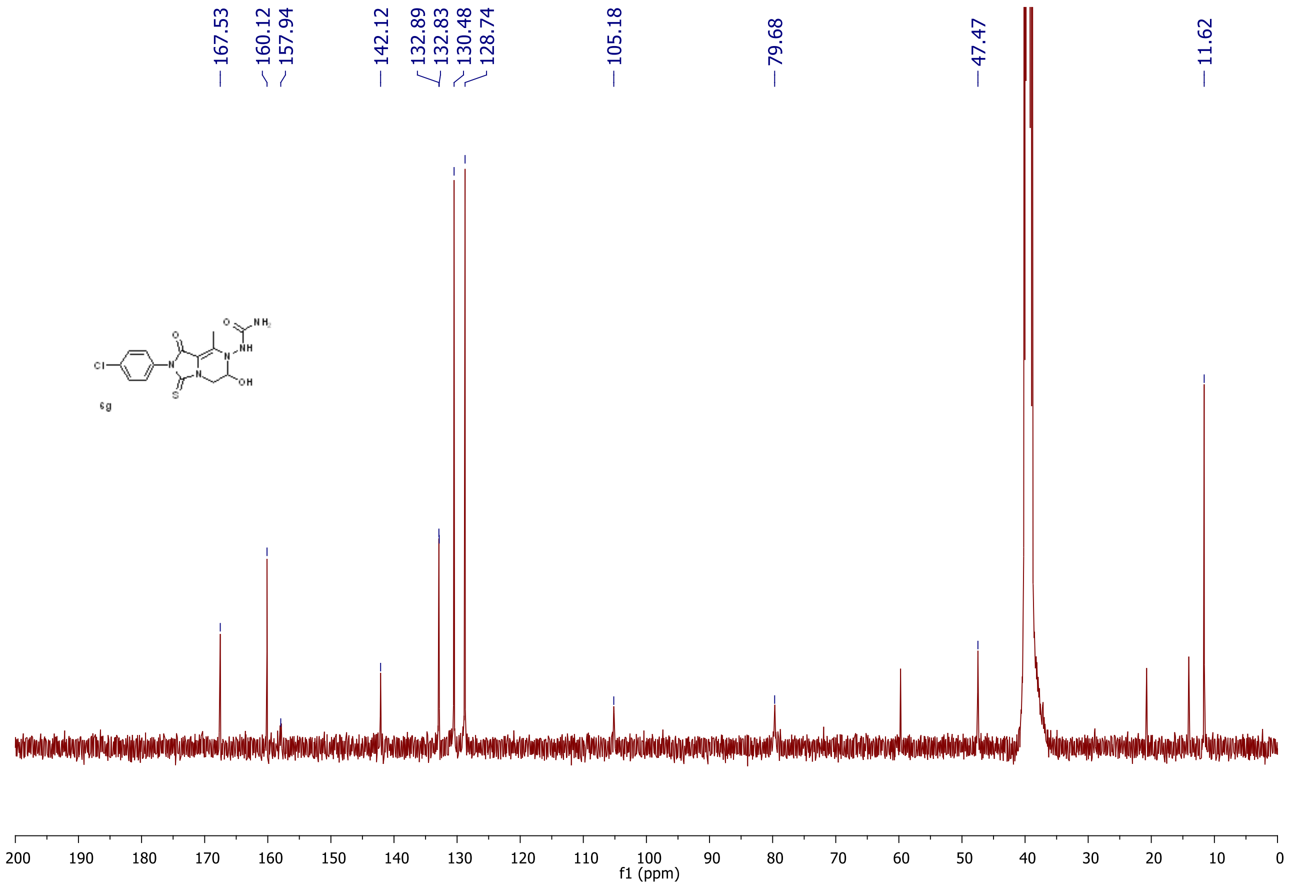
167.53
160.12
157.94
142.12
132.89
132.83
130.48
128.74
105.18
79.68
47.47
11.62
Cl
O
N
NH₂
NH
OH
S
6g
200 190 180 170 160 150 140 130 120 110 100 90 80 70 60 50 40 30 20 10 0
f1 (ppm)

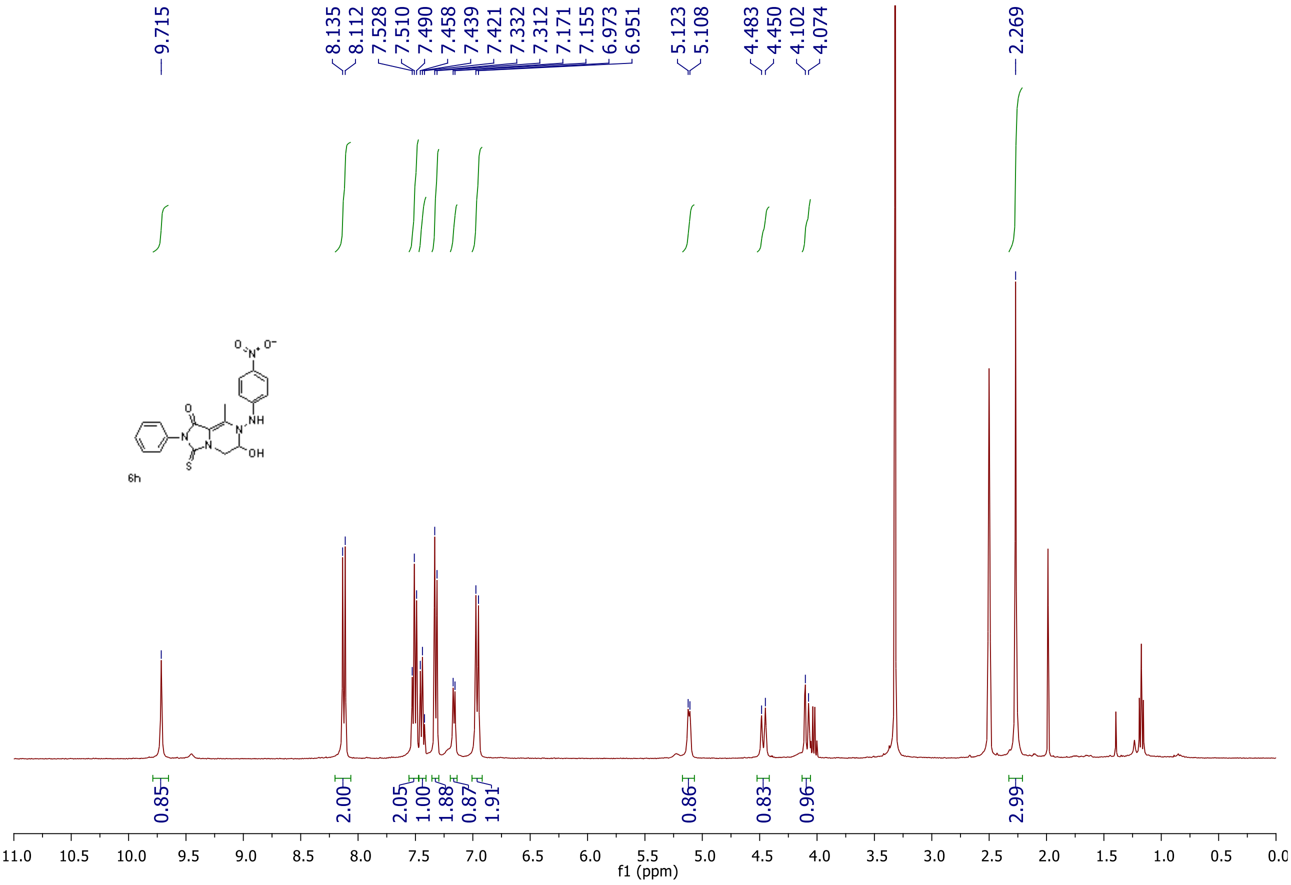
9.715
8.135
8.112
7.528
7.510
7.490
7.458
7.439
7.421
7.332
7.312
7.171
7.155
6.973
6.951
5.123
5.108
4.483
4.450
4.102
4.074
2.269
6h
0.85
2.00
2.05
1.00
1.88
0.87
1.91
0.86
0.83
0.96
2.99
f1 (ppm)

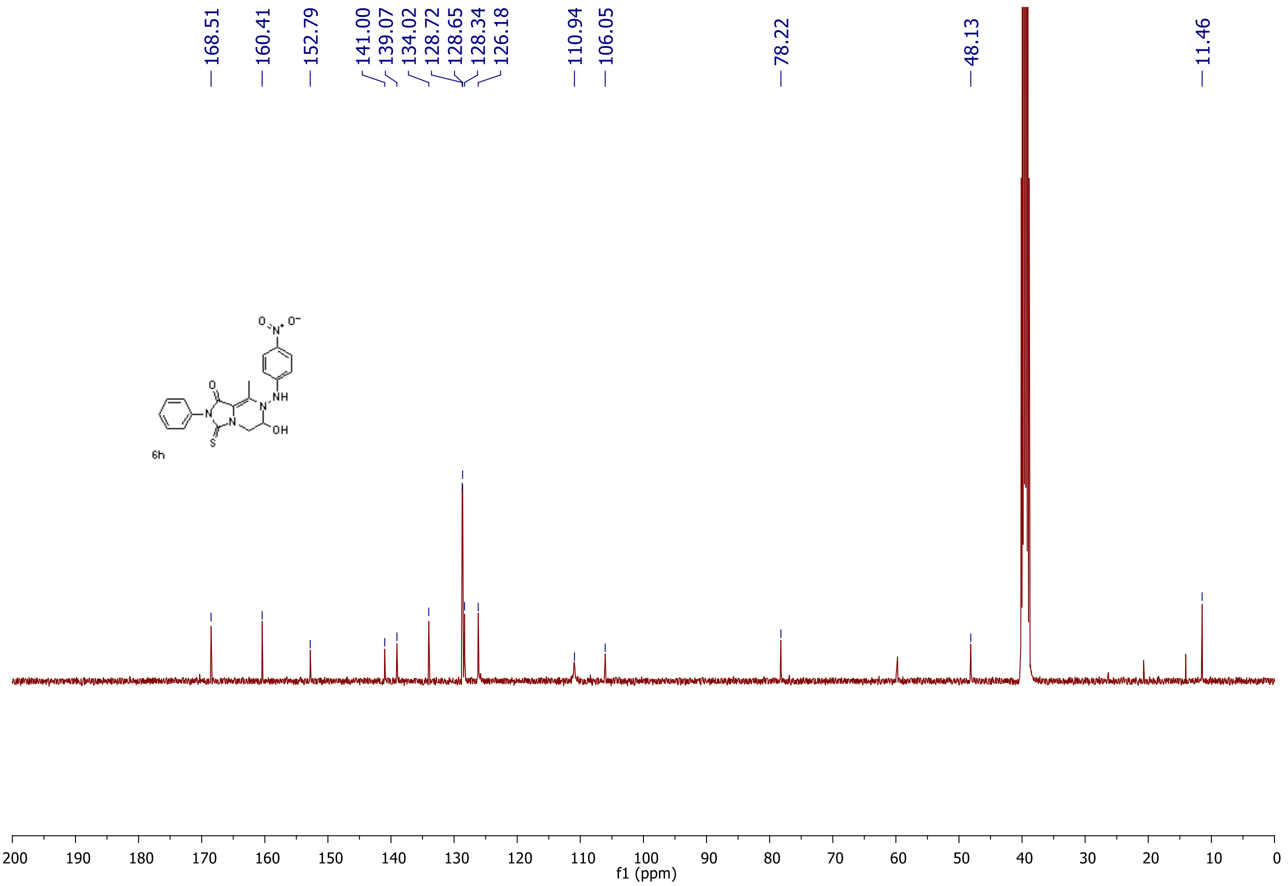
168.51
160.41
152.79
141.00
139.07
134.02
128.72
128.65
128.34
126.18
110.94
106.05
78.22
48.13
11.46
6h
f1 (ppm)

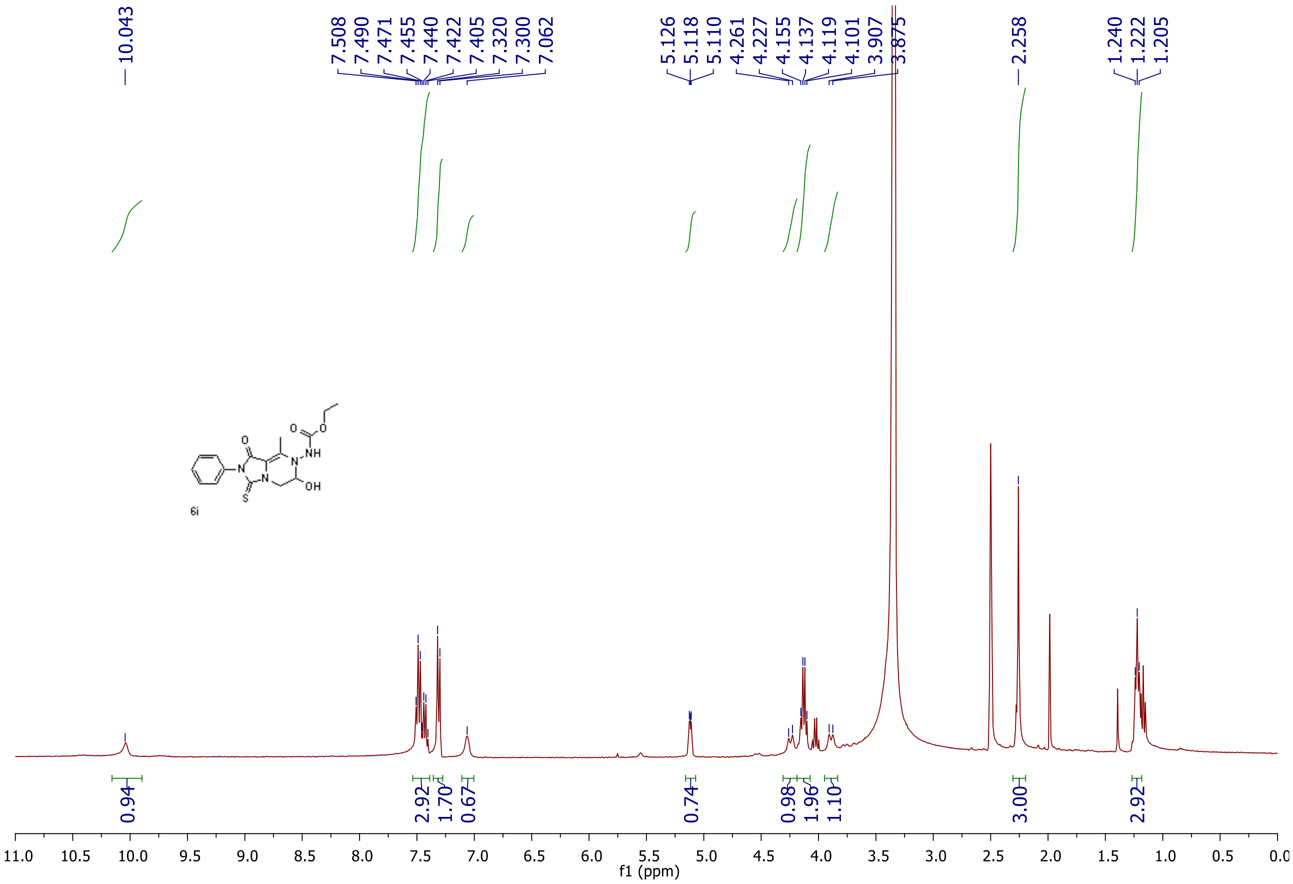
10.043
7.508
7.490
7.471
7.455
7.440
7.422
7.405
7.320
7.300
7.062
5.126
5.118
5.110
4.261
4.227
4.155
4.137
4.119
4.101
3.907
3.875
2.258
1.240
1.222
1.205
0.94
2.92
1.70
0.67
0.74
0.98
1.96
1.10
3.00
2.92
6i
f1 (ppm)

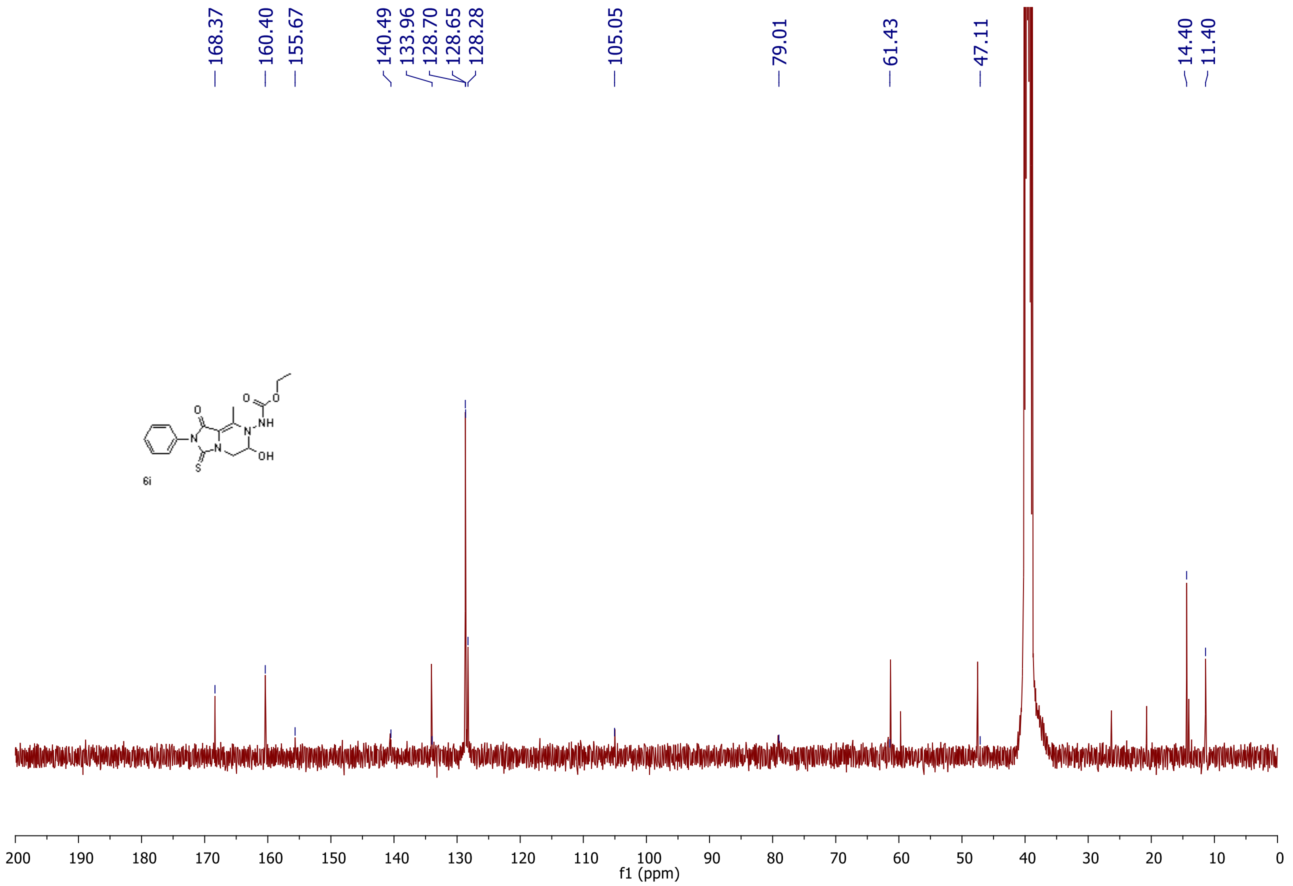
168.37
160.40
155.67
140.49
133.96
128.70
128.65
128.28
105.05
79.01
61.43
47.11
14.40
11.40
6i
f1 (ppm)

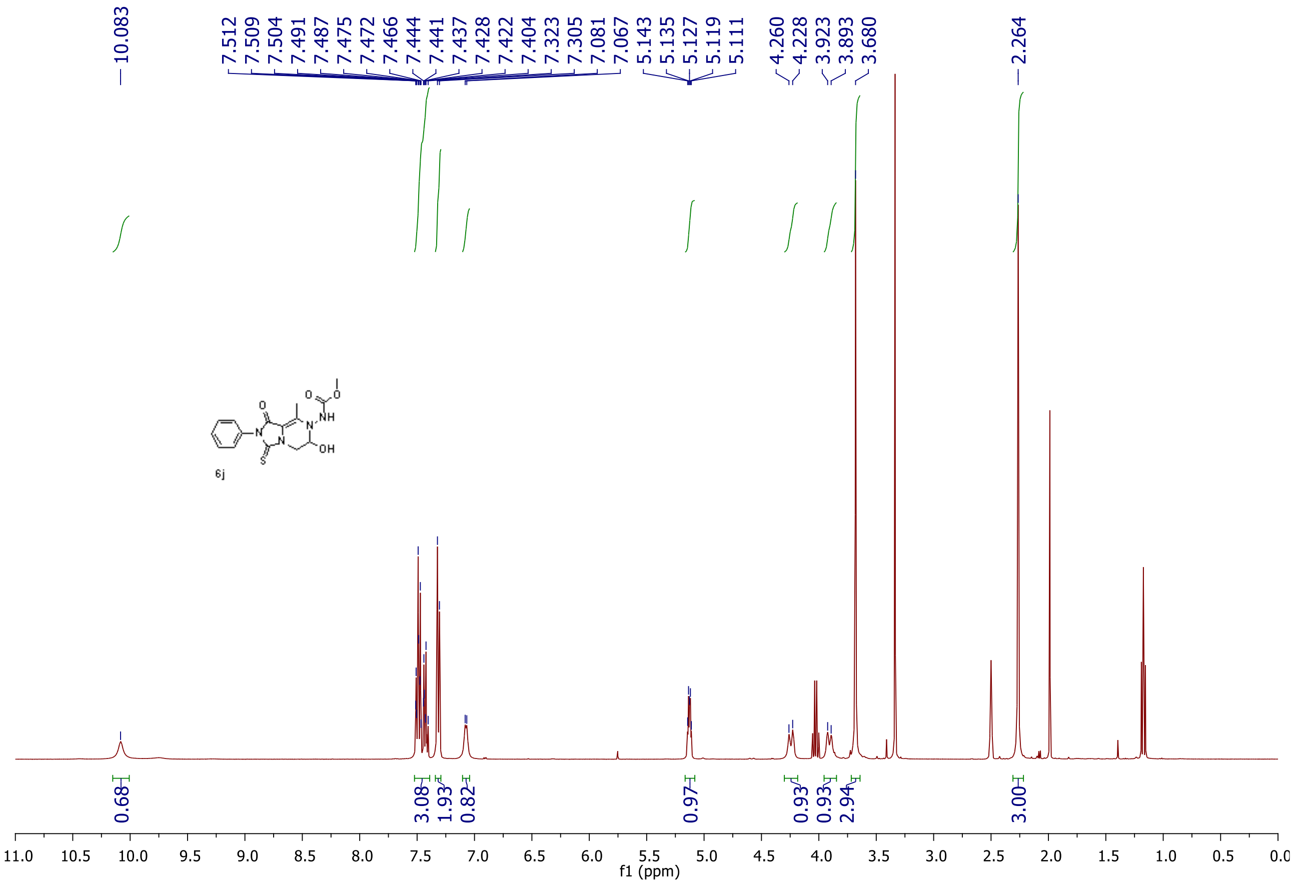
10.083
7.512
7.509
7.504
7.491
7.487
7.475
7.472
7.466
7.444
7.441
7.437
7.428
7.422
7.404
7.323
7.305
7.081
7.067
5.143
5.135
5.127
5.119
5.111
4.260
4.228
3.923
3.893
3.680
2.264
6j
0.68
3.08
1.93
0.82
0.97
0.93
0.93
2.94
3.00
f1 (ppm)

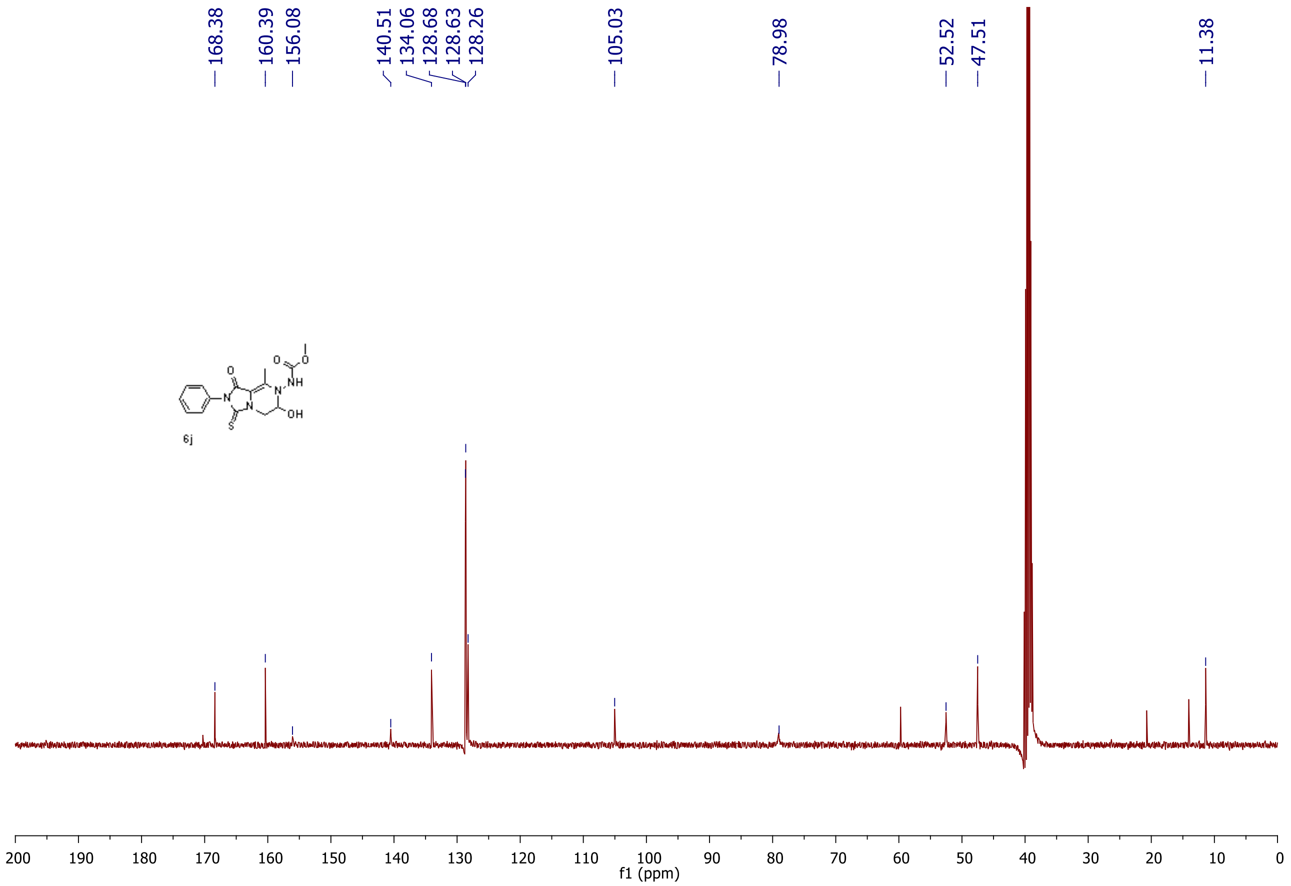
168.38
160.39
156.08
140.51
134.06
128.68
128.63
128.26
105.03
78.98
52.52
47.51
11.38
6j
f1 (ppm)

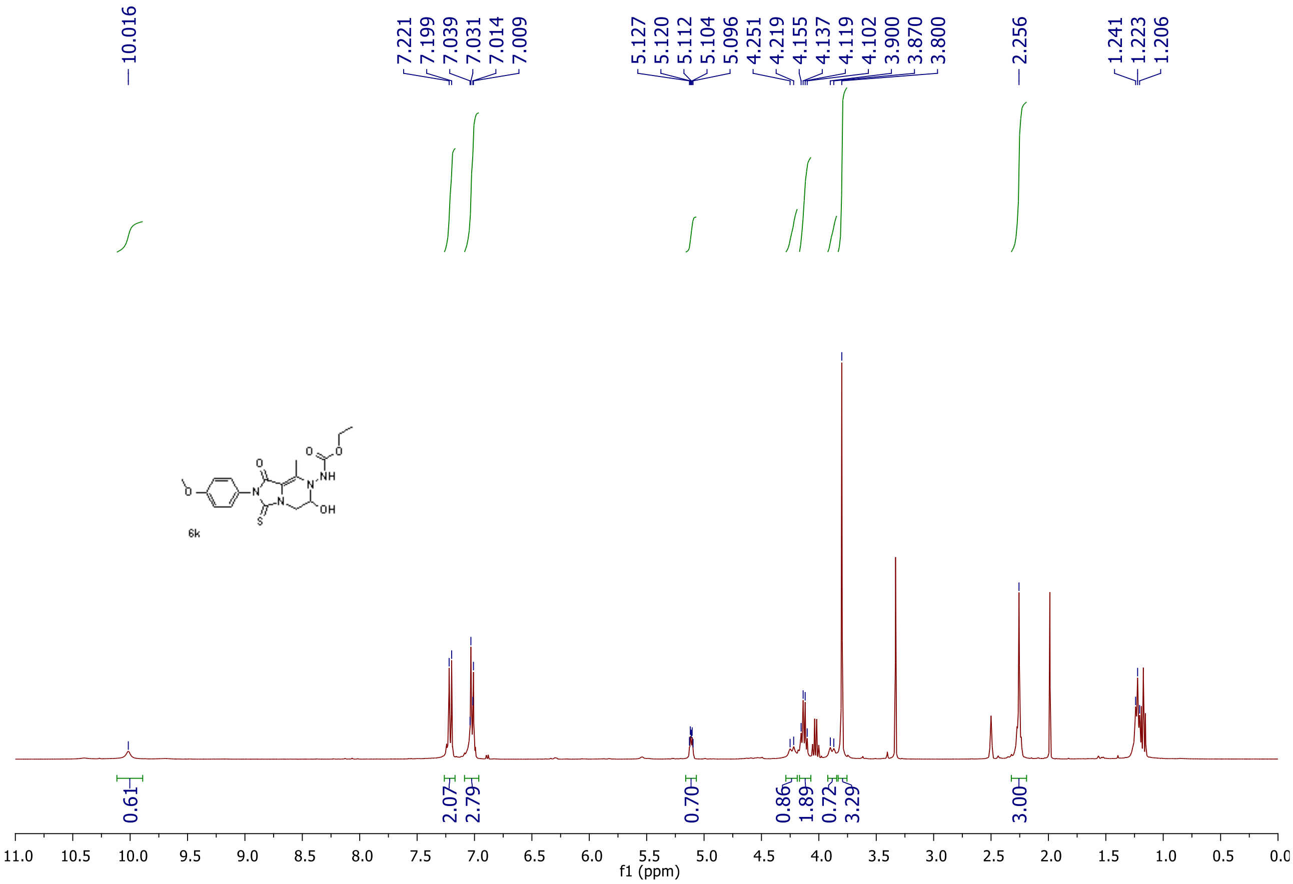
10.016
7.221
7.199
7.039
7.031
7.014
7.009
5.127
5.120
5.112
5.104
5.096
4.251
4.219
4.155
4.137
4.119
4.102
3.900
3.870
3.800
2.256
1.241
1.223
1.206
6k
0.61
2.07
2.79
0.70
0.86
1.89
0.72
3.29
3.00
f1 (ppm)

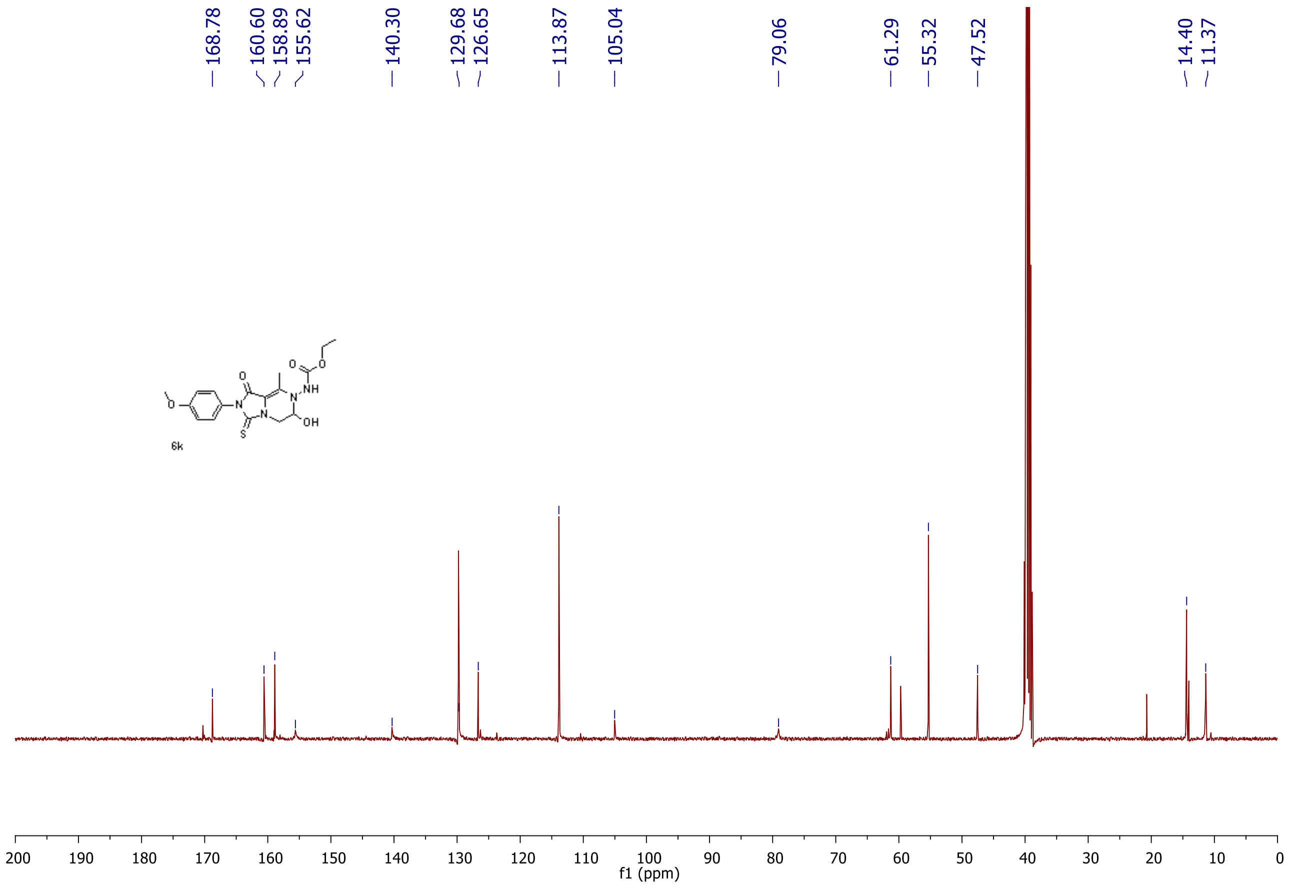
168.78
160.60
158.89
155.62
140.30
129.68
126.65
113.87
105.04
79.06
61.29
55.32
47.52
14.40
11.37
6k
f1 (ppm)

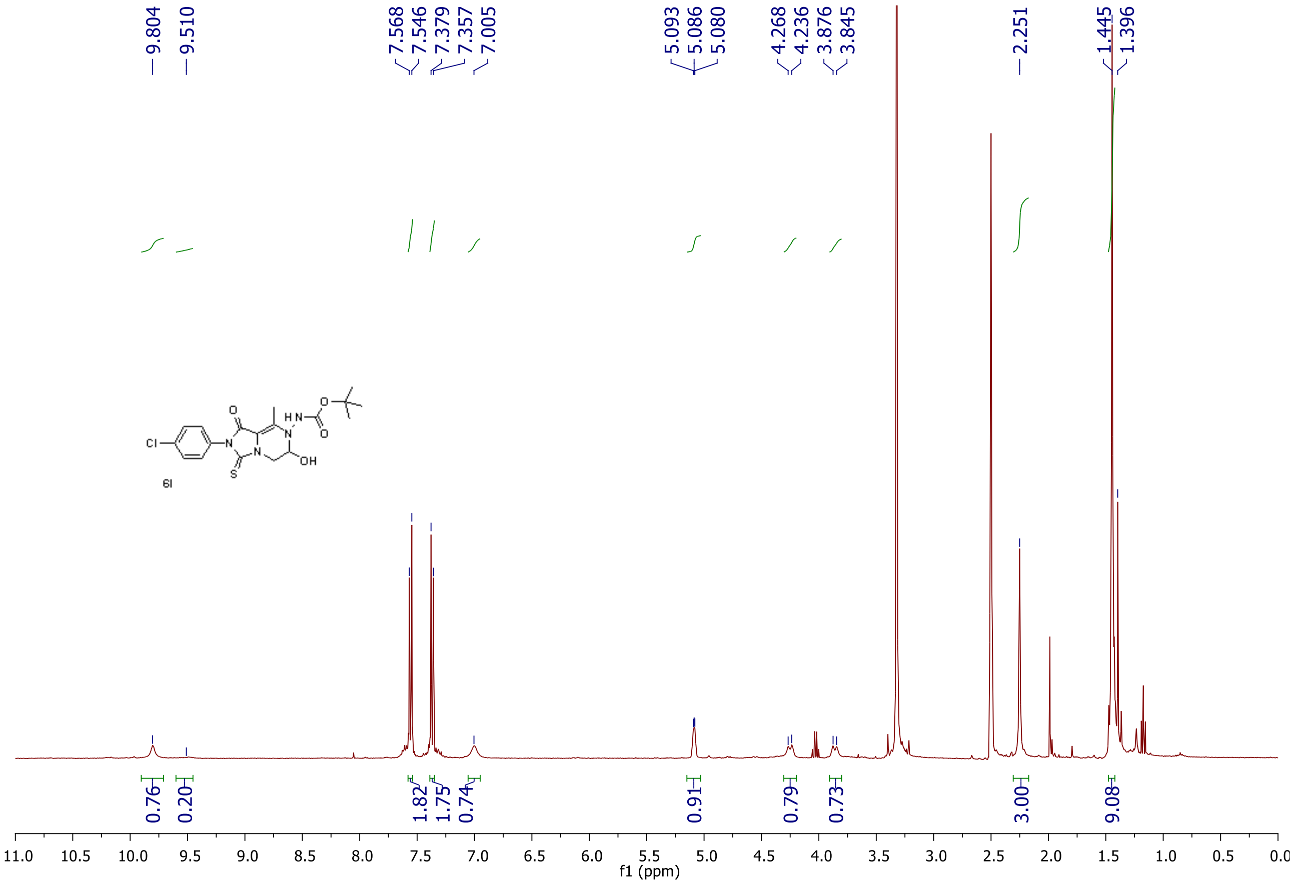
9.804
9.510
7.568
7.546
7.379
7.357
7.005
5.093
5.086
5.080
4.268
4.236
3.876
3.845
2.251
1.445
1.396
6l
0.76
0.20
1.82
1.75
0.74
0.91
0.79
0.73
3.00
9.08
11.0 10.5 10.0 9.5 9.0 8.5 8.0 7.5 7.0 6.5 6.0 5.5 5.0 4.5 4.0 3.5 3.0 2.5 2.0 1.5 1.0 0.5
f1 (ppm)

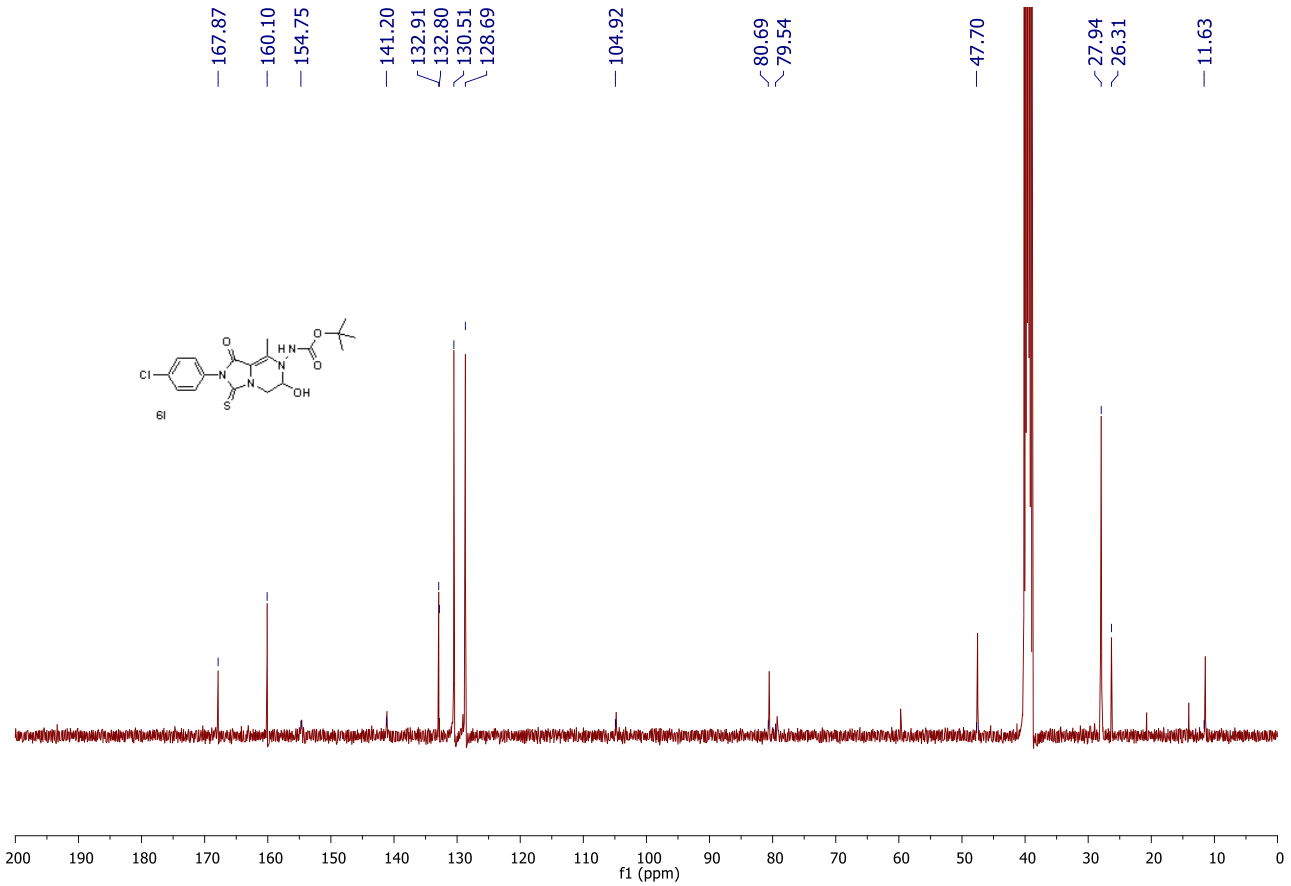
167.87
160.10
154.75
141.20
132.91
132.80
130.51
128.69
104.92
80.69
79.54
47.70
27.94
26.31
11.63
6l
200 190 180 170 160 150 140 130 120 110 100 90 80 70 60 50 40 30 20 10 0
f1 (ppm)

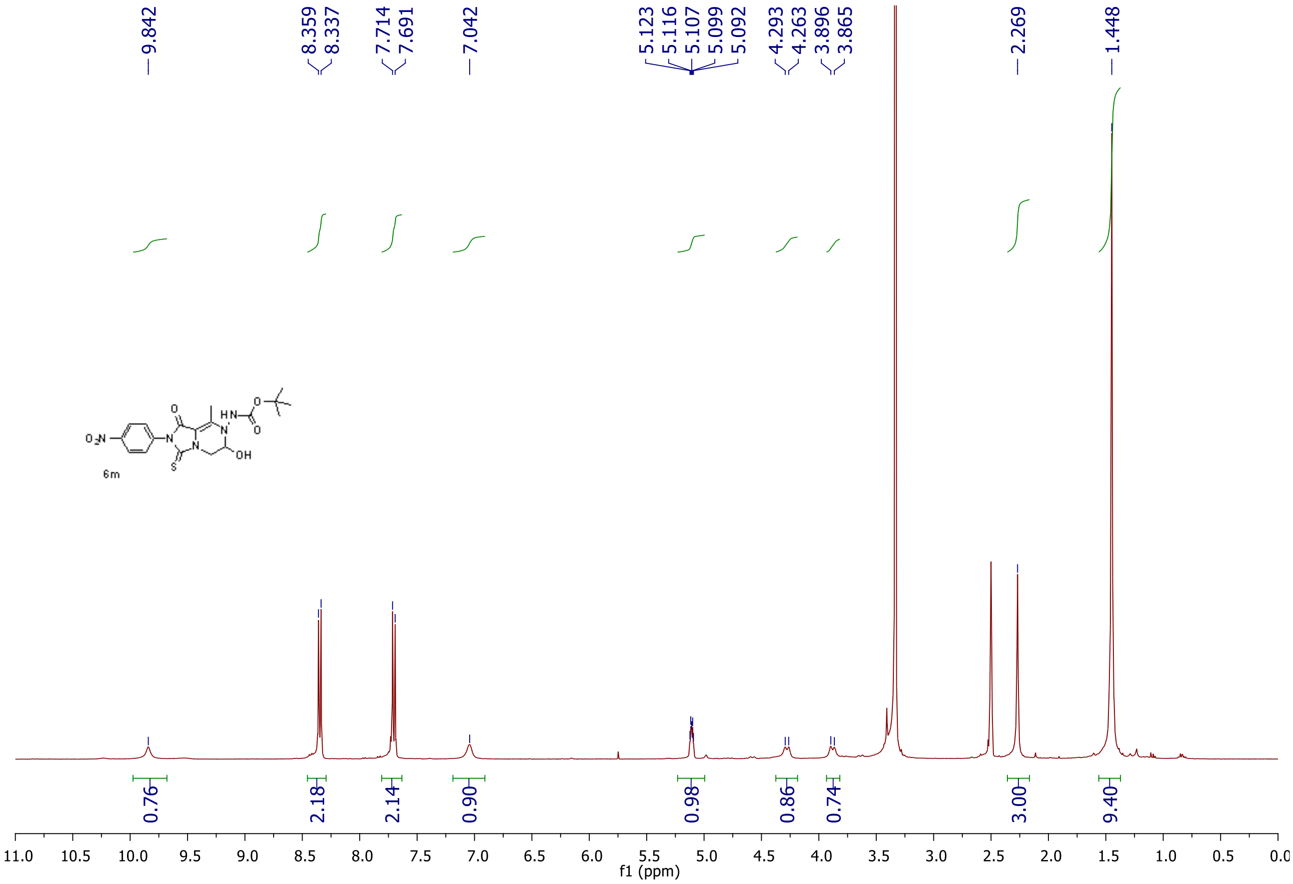
9.842
8.359
8.337
7.714
7.691
7.042
5.123
5.116
5.107
5.099
5.092
4.293
4.263
3.896
3.865
2.269
1.448
0.76
2.18
2.14
0.90
0.98
0.86
0.74
3.00
9.40
6m
f1 (ppm)

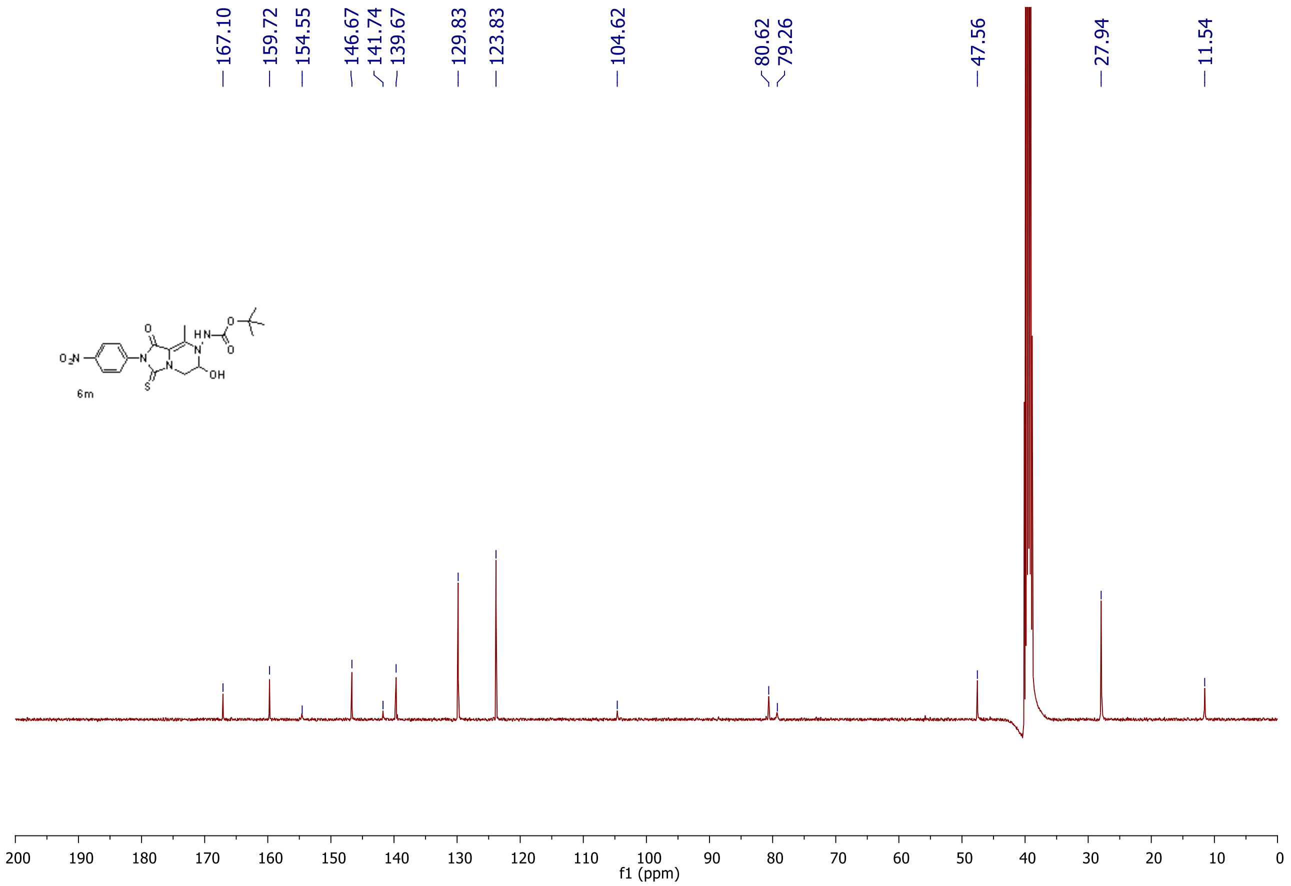
167.10
159.72
154.55
146.67
141.74
139.67
129.83
123.83
104.62
80.62
79.26
47.56
27.94
11.54
6m
f1 (ppm)

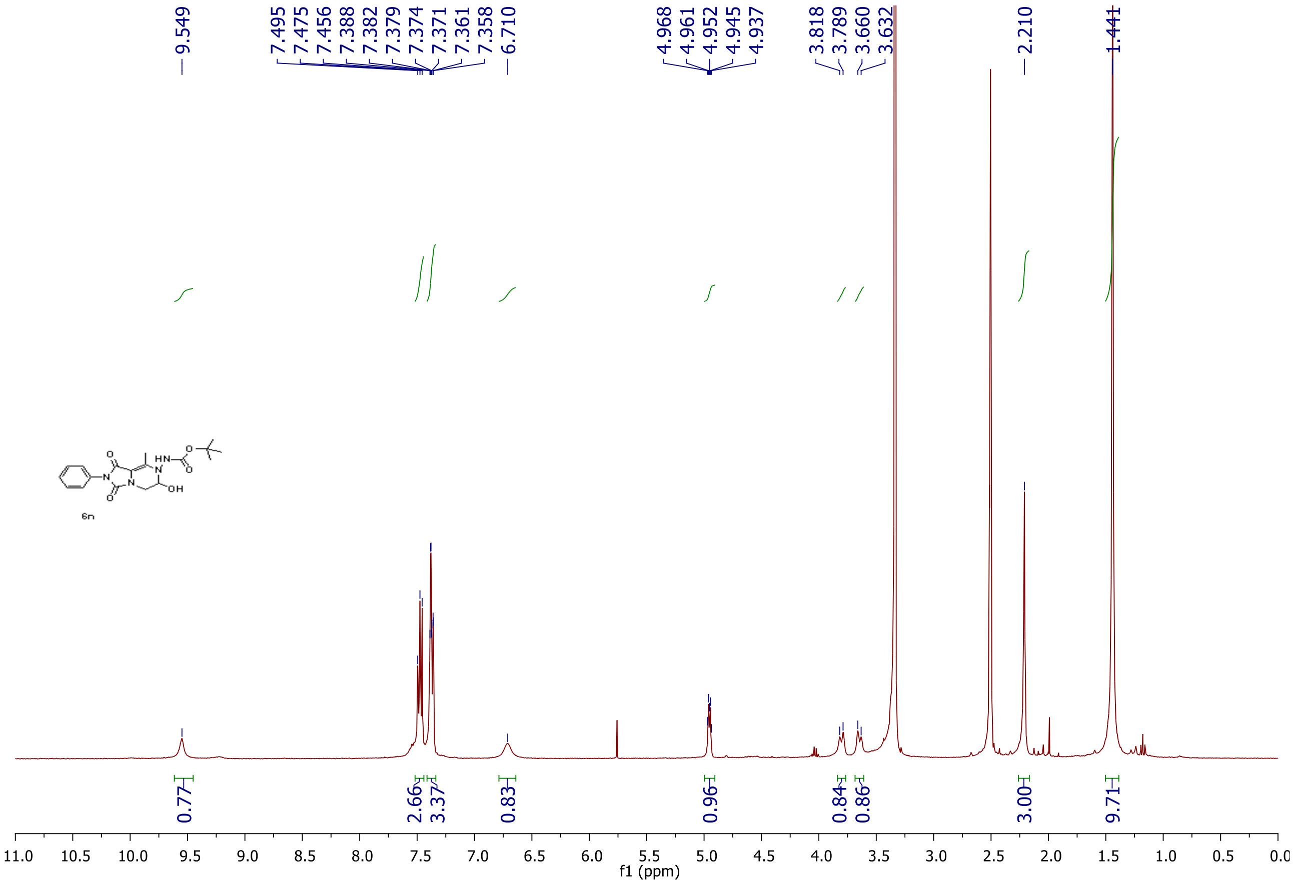
9.549
7.495
7.475
7.456
7.388
7.382
7.379
7.374
7.371
7.361
7.358
6.710
4.968
4.961
4.952
4.945
4.937
3.818
3.789
3.660
3.632
2.210
1.441
0.77
2.66
3.37
0.83
0.96
0.84
0.86
3.00
9.71
6n
f1 (ppm)

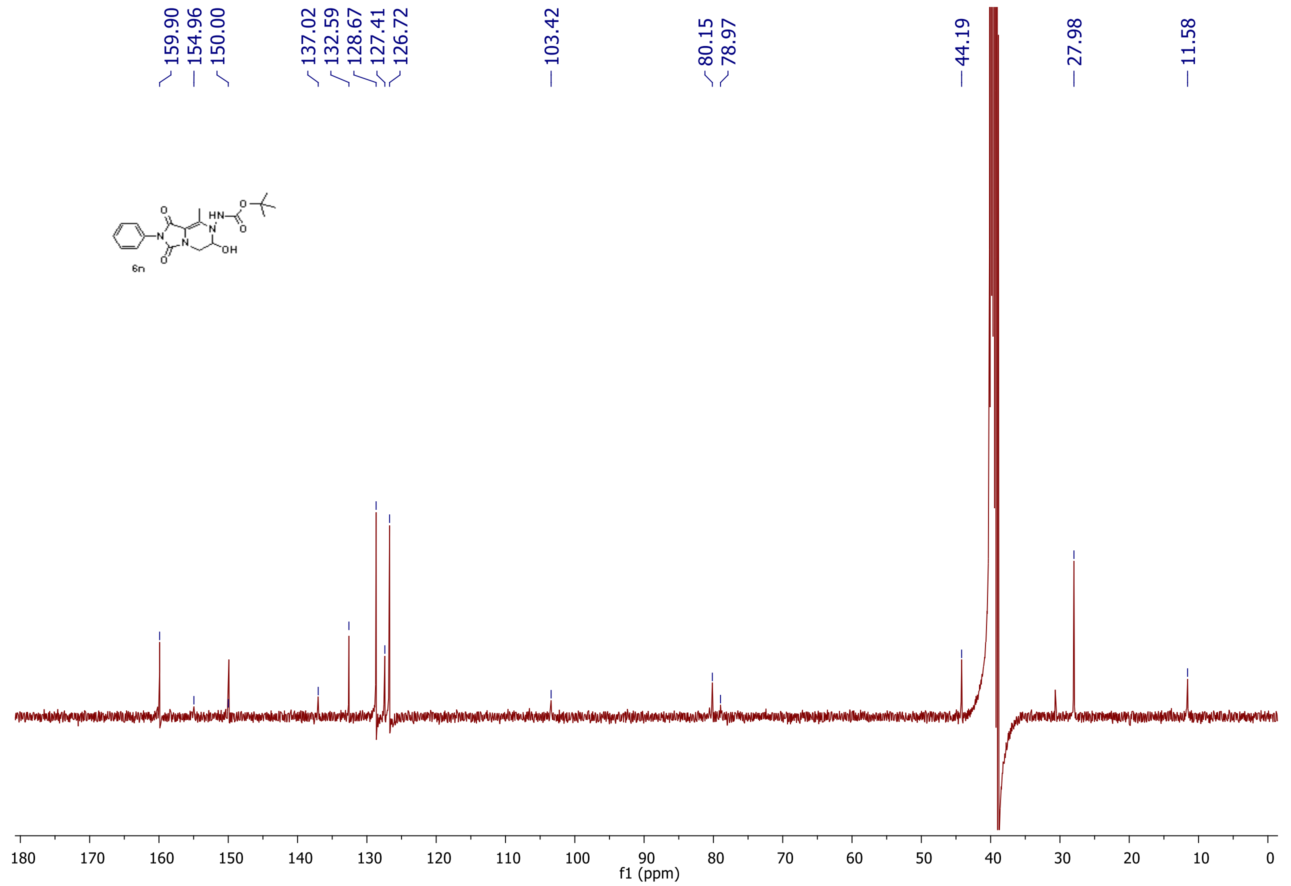
159.90
154.96
150.00
137.02
132.59
128.67
127.41
126.72
103.42
80.15
78.97
44.19
27.98
11.58
6n
f1 (ppm)

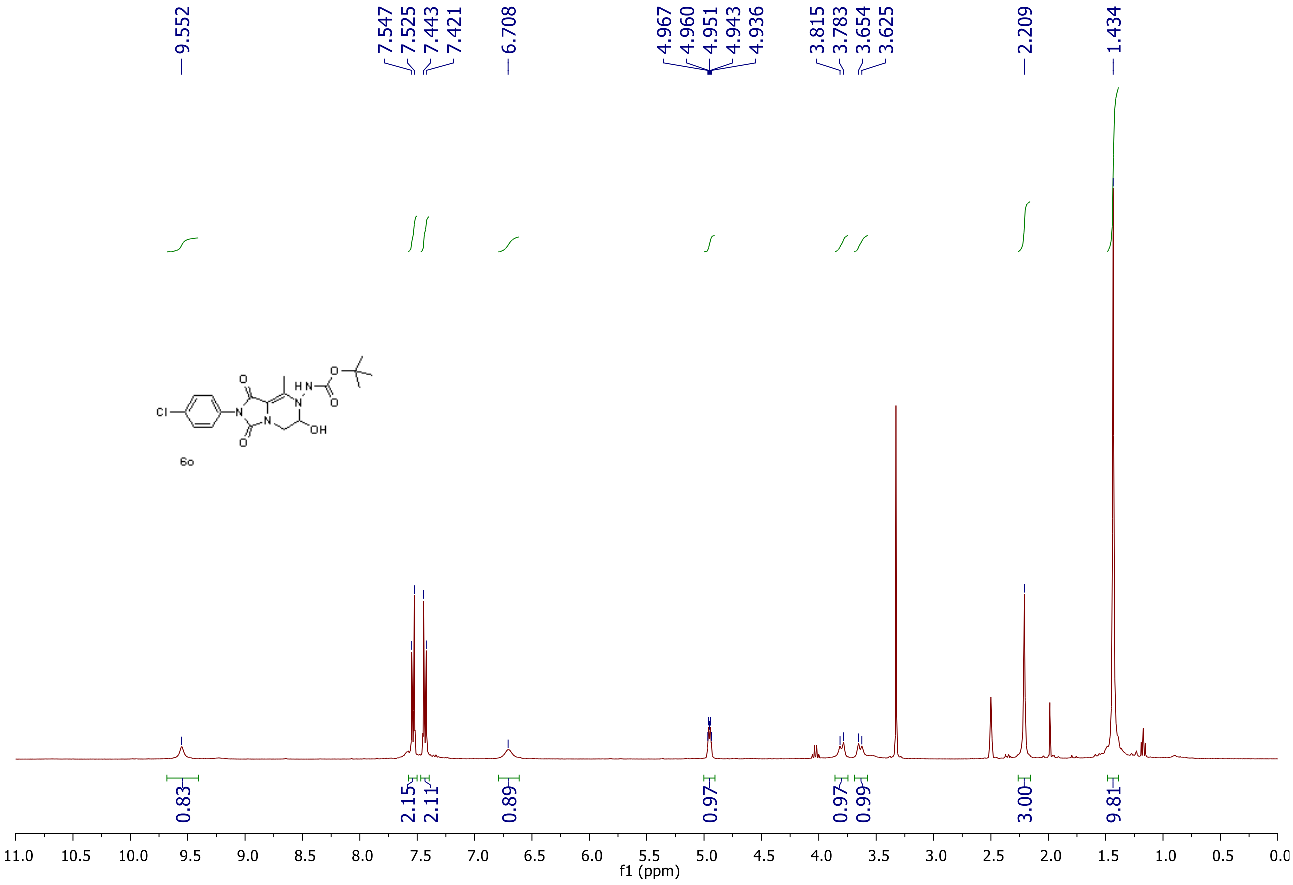
9.552
7.547
7.525
7.443
7.421
6.708
4.967
4.960
4.951
4.943
4.936
3.815
3.783
3.654
3.625
2.209
1.434
6o
0.83
2.15
2.11
0.89
0.97
0.97
0.99
3.00
9.81
f1 (ppm)

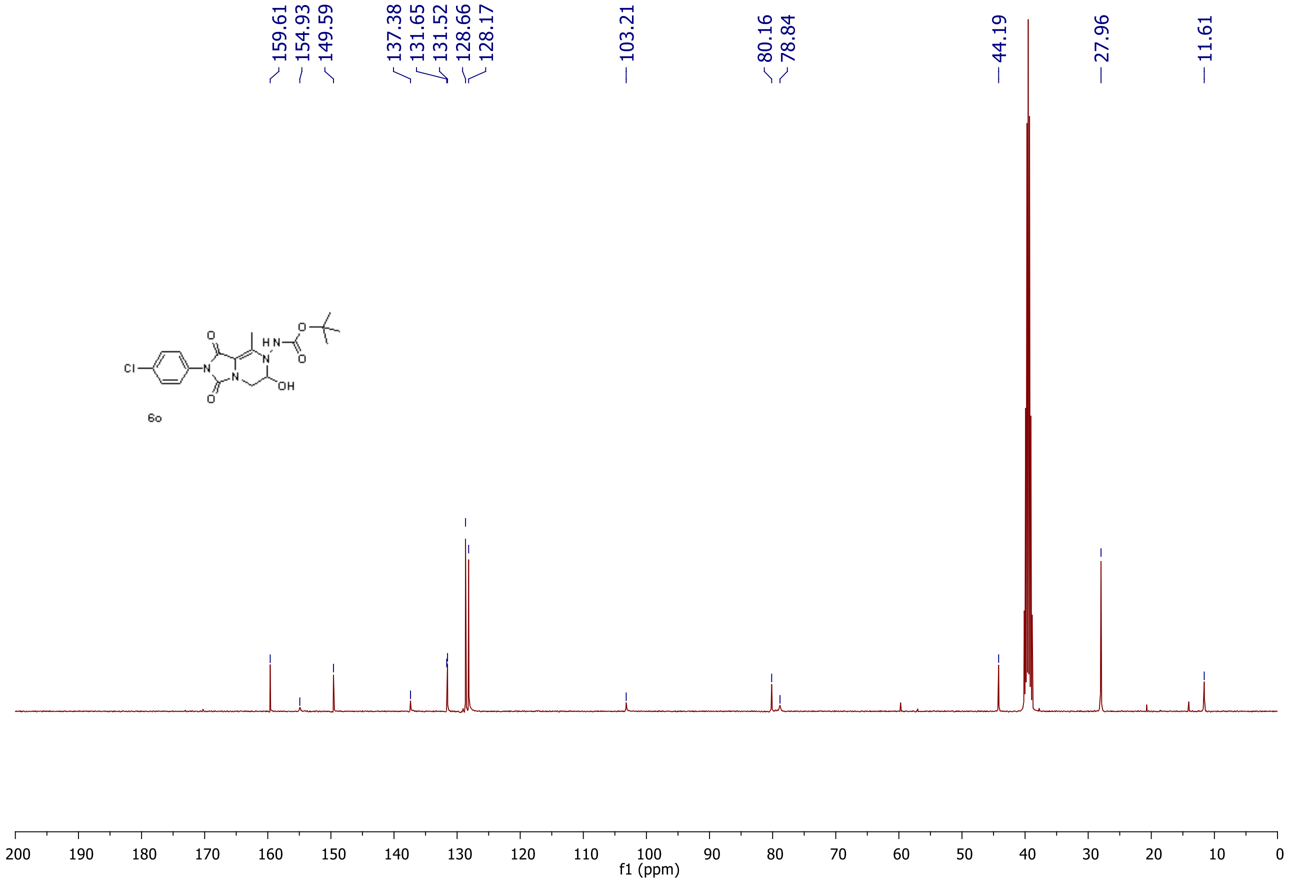
159.61
154.93
149.59
137.38
131.65
131.52
128.66
128.17
103.21
80.16
78.84
44.19
27.96
11.61
6o
f1 (ppm)

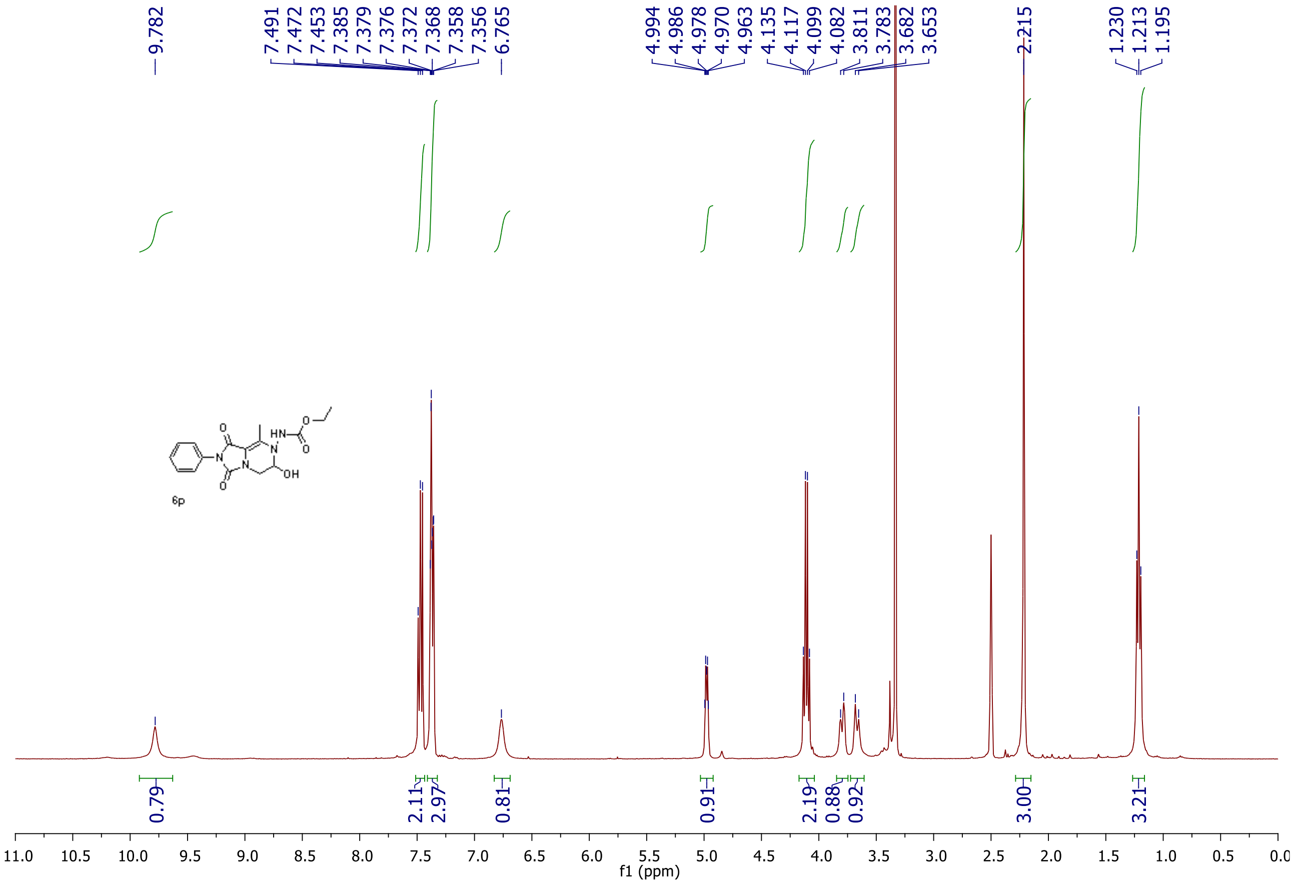
9.782
7.491
7.472
7.453
7.385
7.379
7.376
7.372
7.368
7.358
7.356
6.765
4.994
4.986
4.978
4.970
4.963
4.135
4.117
4.099
4.082
3.811
3.783
3.682
3.653
2.215
1.230
1.213
1.195
6p
0.79
2.11
2.97
0.81
0.91
2.19
0.88
0.92
3.00
3.21
f1 (ppm)

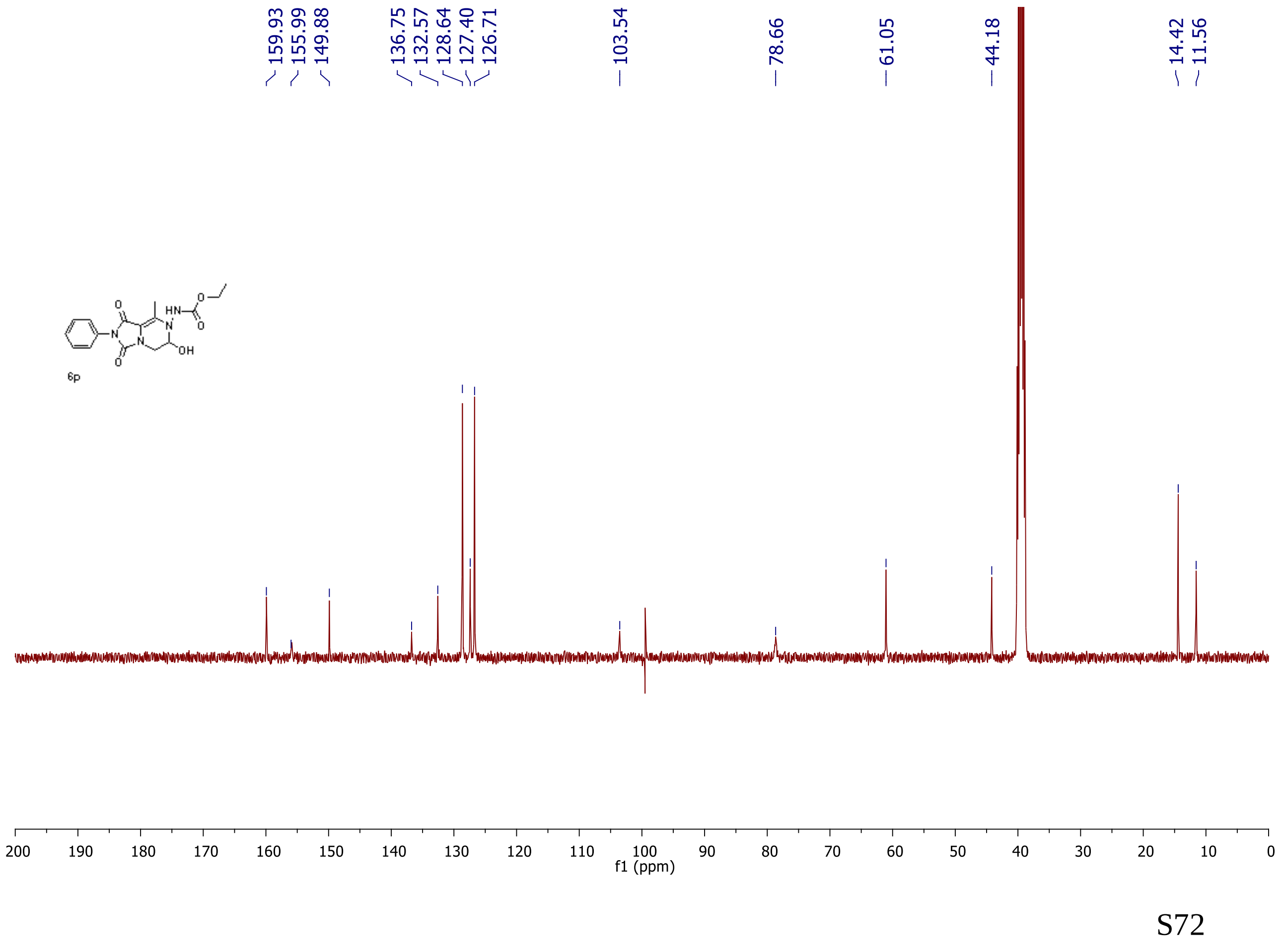
159.93
155.99
149.88
136.75
132.57
128.64
127.40
126.71
103.54
78.66
61.05
44.18
14.42
11.56
6p
f1 (ppm)

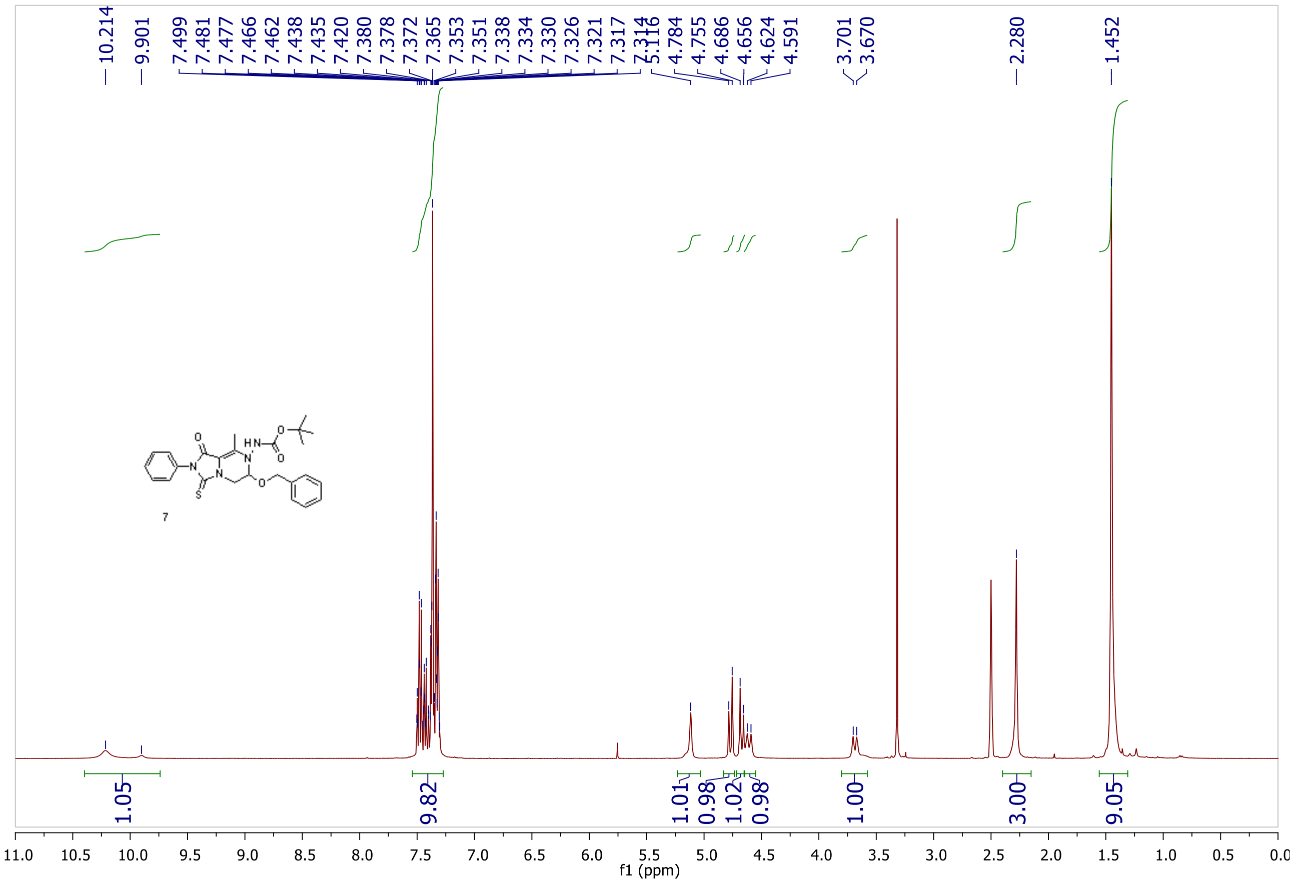
10.214
9.901
7.499
7.481
7.477
7.466
7.462
7.438
7.435
7.420
7.380
7.378
7.372
7.365
7.353
7.351
7.338
7.334
7.330
7.326
7.321
7.317
7.314
5.116
4.784
4.755
4.686
4.656
4.624
4.591
3.701
3.670
2.280
1.452
1.05
9.82
1.01
0.98
1.02
0.98
1.00
3.00
9.05
7
f1 (ppm)

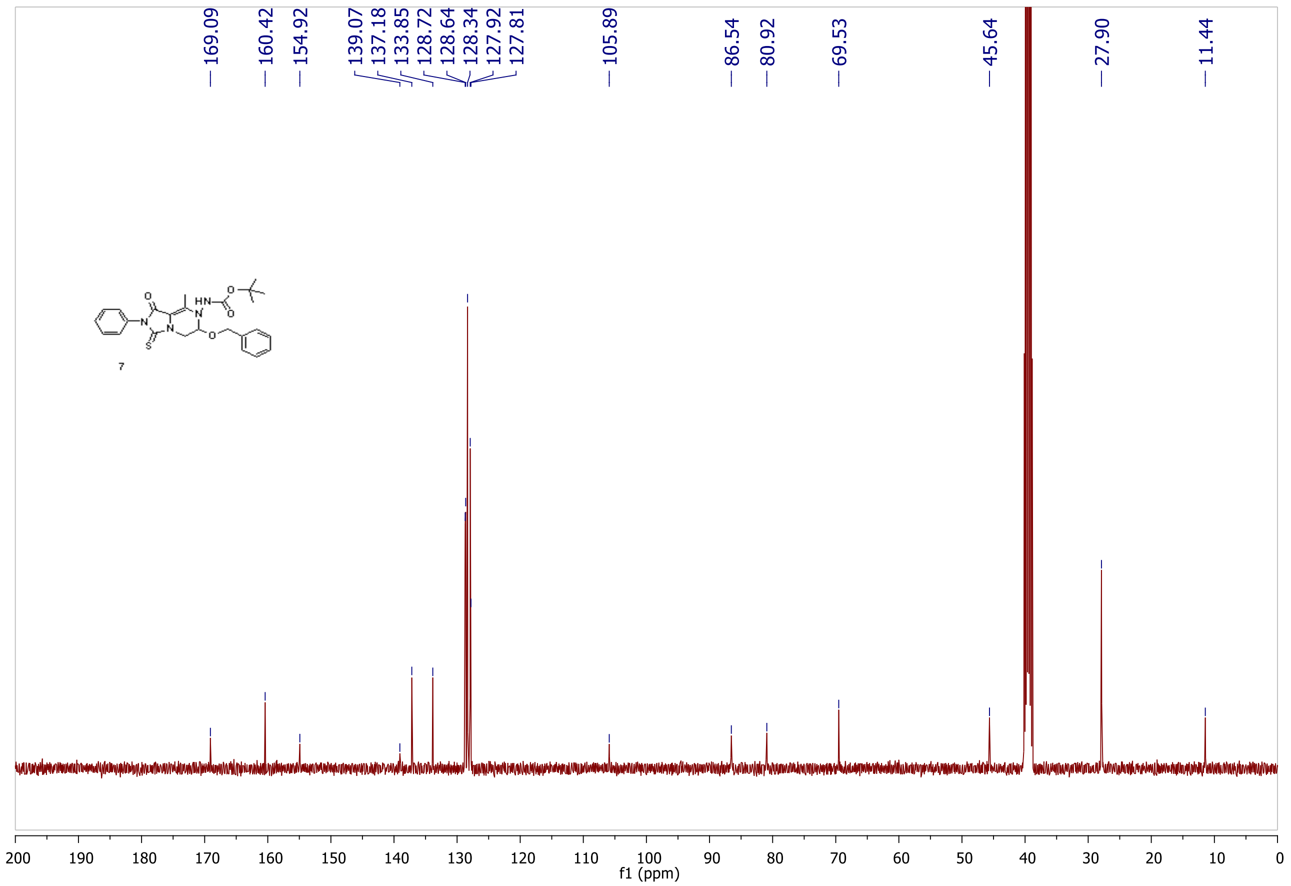
169.09
160.42
154.92
139.07
137.18
133.85
128.72
128.64
128.34
127.92
127.81
105.89
86.54
80.92
69.53
45.64
27.90
11.44
7
f1 (ppm)

8 References

[1] Preti, L.; Attanasi, O. A.; Caselli, E.; Favi, G.; Ori, C.; Davoli, P.; Felluga, F.; Prati, F. *Eur. J. Org. Chem.*, **2010**, 4312-4320. doi:10.1002/ejoc.201000434

[2] Attanasi, O. A.; De Crescentini, L.; Favi, G.; Filippone, Golobǐc, A.; Lillini, S.; Mantellini, F. *Synlett*, **2006**, 2735-2738. doi:10.1055/s-2006-950275;

[3] Mari, G.; De Crescentini, L.; Favi, G.; Mantellini, F.; Santeusanio, S. *Eur. J. Org. Chem.*, **2022**, *40*, 106-111.

[4] Notably, compounds **5f, 5j–r** at NMR analysis show two sets of peaks for the NH moiety. This observation is probably ascribable to the presence of a second axis along the N–N bond that determines the existence of *syn*/*anti*-rotamers of carbamates.

Barrett, K. T.; Metrano, A. J.; Rablen, P. R.; Miller, S. J. *Nature*, **2014**, *509*, 71–75. doi:10.1038/nature13189
